# Rumen metagenome and metatranscriptome analyses of low methane yield sheep reveals a *Sharpea*-enriched microbiome characterised by lactic acid formation and utilisation

**DOI:** 10.1186/s40168-016-0201-2

**Published:** 2016-10-19

**Authors:** Janine Kamke, Sandra Kittelmann, Priya Soni, Yang Li, Michael Tavendale, Siva Ganesh, Peter H. Janssen, Weibing Shi, Jeff Froula, Edward M. Rubin, Graeme T. Attwood

**Affiliations:** 1 AgResearch Limited, Grasslands Research Centre, Tennent Drive, Palmerston North, 4442 New Zealand; 2 Department of Energy, Joint Genome Institute, Walnut Creek, CA 94598 USA; 3 Genomic Division, Lawrence Berkeley National Laboratory, Berkeley, CA 94720 USA

**Keywords:** Bacteria, Metagenomics, Metatranscriptomics, Sheep rumen, Methane, Lactate production, Lactate utilisation

## Abstract

**Background:**

Enteric fermentation by farmed ruminant animals is a major source of methane and constitutes the second largest anthropogenic contributor to global warming. Reducing methane emissions from ruminants is needed to ensure sustainable animal production in the future. Methane yield varies naturally in sheep and is a heritable trait that can be used to select animals that yield less methane per unit of feed eaten. We previously demonstrated elevated expression of hydrogenotrophic methanogenesis pathway genes of methanogenic archaea in the rumens of high methane yield (HMY) sheep compared to their low methane yield (LMY) counterparts. Methane production in the rumen is strongly connected to microbial hydrogen production through fermentation processes. In this study, we investigate the contribution that rumen bacteria make to methane yield phenotypes in sheep.

**Results:**

Using deep sequence metagenome and metatranscriptome datasets in combination with 16S rRNA gene amplicon sequencing from HMY and LMY sheep, we show enrichment of lactate-producing *Sharpea* spp. in LMY sheep bacterial communities. Increased gene and transcript abundances for sugar import and utilisation and production of lactate, propionate and butyrate were also observed in LMY animals. *Sharpea azabuensis* and *Megasphaera* spp. act as important drivers of lactate production and utilisation according to phylogenetic analysis and read mappings.

**Conclusions:**

Our findings show that the rumen microbiome in LMY animals supports a rapid heterofermentative growth, leading to lactate production. We postulate that lactate is subsequently metabolised mainly to butyrate in LMY animals, producing 2 mol of hydrogen and 0.5 mol of methane per mol hexose, which represents 24 % less than the 0.66 mol of methane formed from the 2.66 mol of hydrogen produced if hexose fermentation was directly to acetate and butyrate. These findings are consistent with the theory that a smaller rumen size with a higher turnover rate, where rapid heterofermentative growth would be an advantage, results in lower hydrogen production and lower methane formation. Together with previous methanogen gene expression data, this builds a strong concept of how animal traits and microbial communities shape the methane phenotype in sheep.

**Electronic supplementary material:**

The online version of this article (doi:10.1186/s40168-016-0201-2) contains supplementary material, which is available to authorized users.

## Background

Methane is a particularly strong greenhouse gas with a global warming potential of 34× that of CO_2_ [1]. Approximately, a third of all methane emissions derived from human-related activities are from enteric fermentation in livestock [2] and are emitted mostly from ruminant animals. Animal breeding has been used for many years to select for desirable production traits in ruminant livestock, and breeding low methane emitting animals is being investigated [3–6]. This work has identified animals with methane emission yields (g methane/kg dry matter intake/day) consistently lower or higher than the average animal methane yield, and these animals have been used to breed LMY and HMY lines [5]. The methane yield trait is heritable, and feed particle retention time [7, 8] and rumen volume [3] are thought to contribute to the phenotype. In ruminants, most of the methane is produced in the reticulo-rumen by the action of methanogenic archaea. Previously, it was reported that the main difference between rumen methanogen communities from rumen samples of LMY and HMY sheep was the differentially higher expression of genes involved in the hydrogenotrophic methanogenesis pathway in HMY sheep [9].

There are several possible substrates for methanogenesis in the rumen that originate from bacterial fermentation, including hydrogen, carbon dioxide (CO_2_), formate, acetate and methyl compounds [10]. Among these, hydrogen and CO_2_ are the main substrate for methanogenesis in the rumen [11, 12], and the majority of hydrogen produced from microbial carbohydrate fermentation is used for methane production [12]. Thus, there is a strong connection between microbial fermentation processes, their hydrogen production and methane formation by the methanogenic archaea in the rumen [13], and it is likely that rumen bacterial communities and their activities contribute to the methane yield phenotype of the animal. Differences in the relative abundances of bacteria producing large amounts of hydrogen in the rumens of HMY and LMY animals also support this theory [14]. Here, we used 16S ribosomal ribonucleic acid (rRNA) gene amplicon sequencing and metagenomic and metatranscriptomic sequence analyses of rumen samples from naturally HMY and LMY cohorts of sheep to investigate the hypothesis that the differences in animal methane yield phenotype are linked to differences in bacterial gene abundance and/or transcriptional activity.

## Results

### Animal measurements

An overview of the analyses of methane emissions, pH, fermentation acids, 16S rRNA gene amplicon sequencing and metagenome/metatranscriptome sequencing is provided in Additional file 1: Table S1. The methane yield phenotypes of 23 cross-bred rams (selected from a larger cohort of 96 animals) fed a pelleted lucerne diet were determined previously [9] on two occasions. Animals were classified as LMY (mean 11.44 g methane/kg dry matter intake (DMI)), HMY (mean 15.85 g methane/kg DMI) or as falling between these extremes as “intermediate methane yield” (IMY, mean 13.89 g methane/kg DMI) animals, with a significantly different 28 % methane yield difference between the HMY and LMY groups (*P* = 0.0001) [9].

**Fig. 1:**
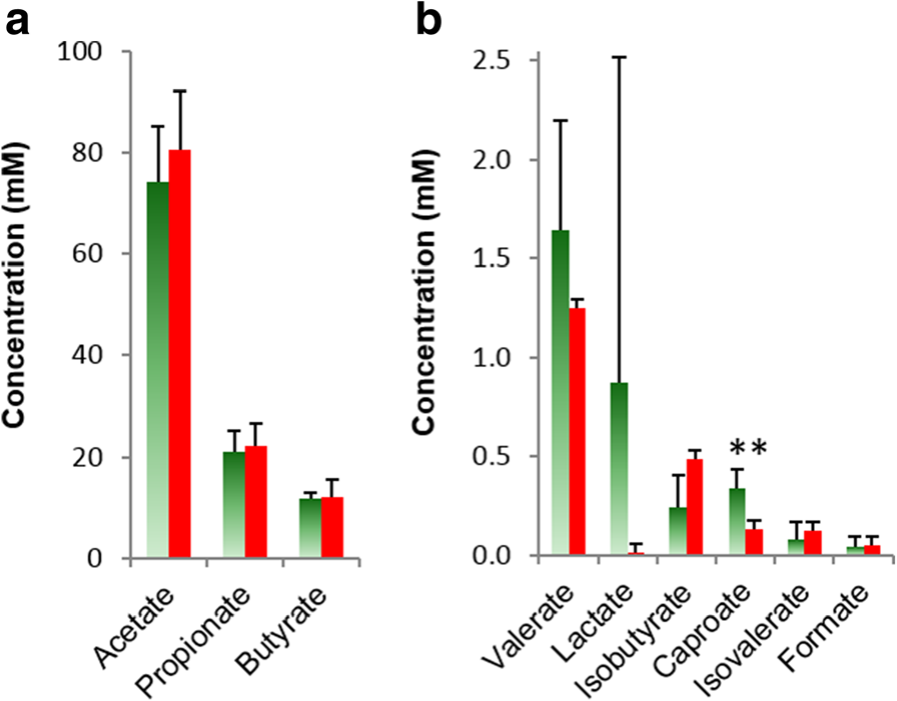
Concentrations of major (**a**) and minor (**b**) fermentation acids in rumen content samples from LMY and HMY sheep. Fermentation acids were determined by GC-MS after derivatisation and normalisation to an ethyl butyrate internal standard, and concentrations shown are in millimolar. **P < 0.01. *Green bars* represent LMY (*n* = 8), and *orange bars* HMY samples (*n* = 8)

Rumen content samples were taken from animals immediately after the last day of methane measurement and the pH, and fermentation products of all samples were measured. There were no differences in pH between HMY and LMY groups (*P* = 0.93). Volatile and non-volatile fatty acids in rumen samples were measured (Fig. 1), and acetate, propionate and butyrate were in the expected ranges [15] and did not differ significantly between the LMY and HMY sheep (Fig. 1a). The other fermentation products were present at low concentrations; formate, isobutyrate and isovalerate were <0.7 mM, valerate was 1 to 2 mM, while lactate was variable, being very low in HMY (mean 0.014 mM) and higher in LMY sheep (~0.9 mM) but with large variations between individual samples (Fig. 1b). Caproate was the only fermentation product that was significantly different (*P* = 0.003) between the two groups of sheep, being ~2.5× greater in the LMY sheep (Fig. 1b).

### Microbial community composition of HMY and LMY animals

**Fig. 2:**
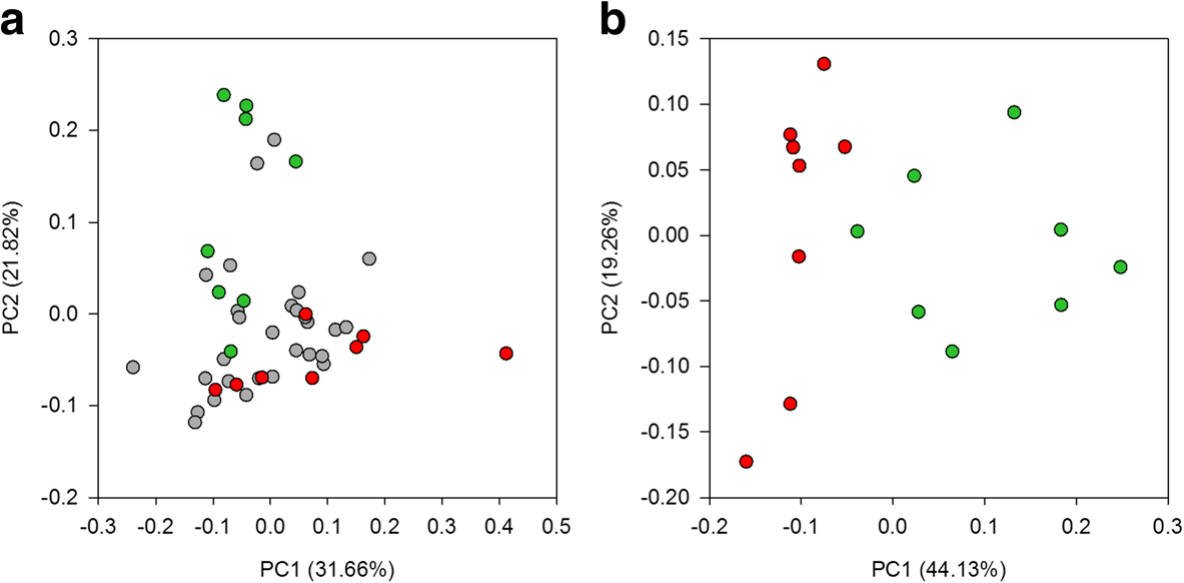
Principal coordinate analysis of rumen bacterial communities from HMY (*red*), LMY (*green*) or IMY (*grey*) sheep based on 16S rRNA gene amplicon sequence data (**a**) and 16S rRNA genes retrieved from the metagenome dataset (**b**). Percentage of data variation explained by the analysis is shown in *brackets*

**Fig. 3:**
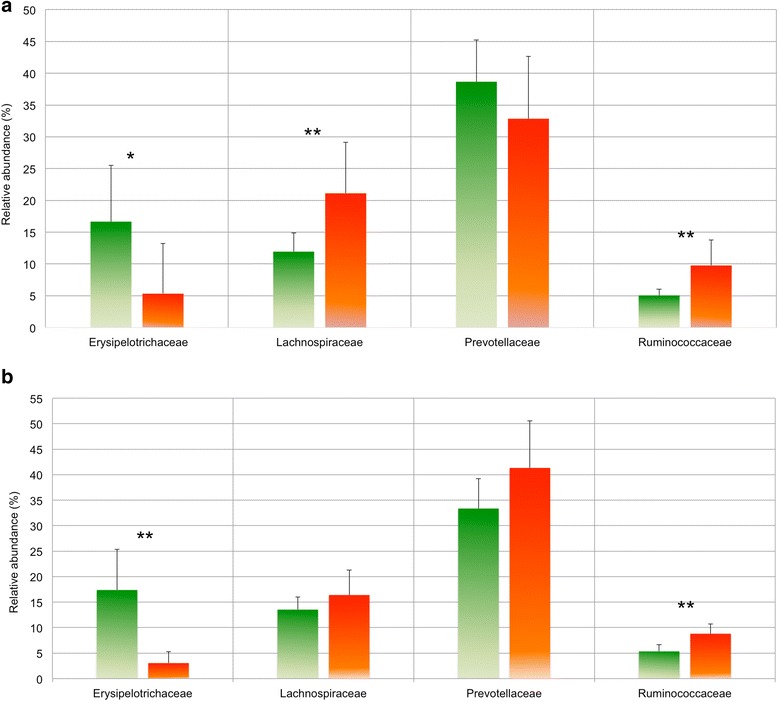
Relative abundance of the most highly represented bacterial families based on 16S rRNA gene amplicon sequencing data (**a**) and 16S rRNA genes retrieved from the metagenome dataset (**b**) from rumen content samples of LMY (*green*) and HMY (*orange*) sheep. **P < 0.01, *P < 0.05. *Error bars* denote one standard deviation

At a threshold >0.2 % relative abundance, 70 bacterial taxa (97 % sequence similarity) were recovered from the 16S rRNA gene amplicon sequences, while 67 taxa were retrieved from the metagenome-derived 16S rRNA genes. Differences in bacterial community composition were estimated using principal coordinate analysis based on the Bray-Curtis dissimilarity metric (PCoA, Fig. 2). HMY animal samples clustered separately from the LMY animals for both the amplicon and the metagenome datasets, while samples from IMY animals fell in between (Fig. 2). At the family level, four taxa made up appoximately 70 % of the 16S rRNA gene amplicon sequences and 16S rRNA genes derived from the metagenome data in all samples: Prevotellaceae, Lachnospiraceae, Ruminococcaceae and Erysipelotrichaceae (Fig. 3). While there was no significant difference (*P* ≤ 0.05) in the abundance of Prevotellaceae between HMY and LMY animals, all other taxa showed differential abundance, with Lachnospiraceae and Ruminococcaceae being more abundant in HMY samples and Erysipelotrichaceae more abundant in LMY samples based on amplicon sequencing data (Fig. 3a). The metagenomic 16S rRNA gene data confirmed the significantly higher abundance of Ruminococcaceae in HMY animals and Erysipelotrichaceae in LMY animals (Fig. 3b). At the genus- and species-level resolution, nine taxa showed significantly different relative abundances in HMY and LMY samples based on one-way ANOVA (*P* ≤ 0.05), four of which were more abundant in HMY animals and five were more abundant in LMY animals (Table 1). The most notable were *Sharpea* sp. and *S. azabuensis* (both family Erysipelotrichaceae), which were more abundant in LMY animals based on both amplicon and metagenome datasets, making up 6.3 and 7.5 % of the bacterial 16S rRNA gene reads from the LMY animals, respectively. *Megasphaera* spp. were also significantly more abundant in LMY samples in both datasets, with an average relative abundance of ~1 % in these animals. All three taxa were negatively and significantly correlated with methane yield in the Spearman’s rank correlation analysis (Table 1). In HMY animals, smaller differences in relative abundances were found at the genus and species level, with higher abundances of *Anaerostipes* sp. (~1 % in both amplicon and metagenome datasets) and Verrucomicrobia family RFP12 (mean 0.17 %).

**Table 1:** Bacterial taxa (97 % sequence similarity) with taxonomy assigned to highest possible resolution, differing in mean relative abundance (%) between HMY and LMY animals measured at two time points. Significances are based on one-way ANOVA and Bonferroni corrected *P* values

Taxon: order/family/genus	Methane yield group	Amplicon			Metagenome			Spearman	
		*P*	Low	High	*P*	Low	High	R	*P*
Clostridiales/Christensenellaceae	High	<0.01	0.05	0.42	NS	0.05	0.27	0.8	<0.01
Clostridiales/Lachnospiraceae/*Anaerostipes*	High	<0.01	0.12	1.2	NS	0.13	1.02	0.58	<0.01
Verrucomicrobia/Verruco-5/WCHB1/RFP12	High	<0.01	0.02	0.17	<0.01	0.03	0.16	0.67	<0.01
Bacteroidales/BS11	High	NS	0.03	0.35	<0.05	0.01	0.1	0.6	<0.01
Erysipelotrichales/Erysipelotrichaceae/*Sharpea*	Low	<0.05	6.43	0.49	<0.05	6.3	0.58	−0.52	<0.01
Coriobacteriales/Coriobacteriaceae/*Collinsella aerofaciens*	Low	<0.01	0.42	0.02	<0.05	0.62	0.01	−0.6	<0.01
Clostridiales/Eubacteriaceae/*Pseudoramibacter*	Low	<0.05	0.19	0.09	NS	0.25	0.1	NA	NA
Clostridiales/Veillonellaceae/*Megasphaera*	Low	<0.05	1.02	0.06	NS	1.41	0.07	−0.54	<0.01
Erysipelotrichales/Erysipelotrichaceae*/Sharpea azabuensis*	Low	<0.05	7.47	0.55	<0.05	7.83	0.68	−0.7	<0.01

Taxa with significant difference (*P* ≤ 0.05) in either 16S rRNA gene amplicon or 16S rRNA gene metagenome sequence abundance are shown. Spearman’s Rank Correlation based on amplicon sequencing data is included where −0.5 ≤ *R* ≥ 0.5 and *P* ≤ 0.01

*NS* not significant, *NA* not applicable

### Differentially abundant KEGG genes and transcripts

Three statistical analyses, Wilcoxon rank sum (WRS) test, sparse partial least squares (sPLS) regression and gene set enrichment analysis (GSEA), were used to identify differentially abundant KEGG genes in the metagenome and metatranscriptome datasets. Strong correlations between predictor genes/transcripts and animal methane yield were revealed by sPLS regression, with an adjusted *R* ^2^ of 0.962 (*P* = 1.57 × 10^−14^) and 0.987 (*P* = 2.2 × 10^−16^) for metagenome and metatranscriptome data, respectively (Additional file 2: Figure S1). These predictor genes overlapped well with genes and transcripts that were significantly differentially abundant in the WRS test (Additional file 3: Table S2) and resulted in a similar representation of gene categories as found by GSEA (Additional file 4: Table S3). We subsequently focused on selected subsets of genes/transcripts where correspondence to methane yield was supported by at least two of these analyses. Detailed results for each statistical analysis are elaborated in Additional file 5: Text S1, Additional file 3: Table S2. Similar to the microbial community composition data, the majority of differentially more abundant genes, transcripts and pathways identified were associated with the LMY animals. For the subsequent analyses, we focused on the most significant gene categories, including amino acid biosynthesis, phosphotransferase systems (PTS), galactose metabolism and short chain fatty acid metabolism.

### Biosynthesis of amino acids

GSEA identified amino acid biosynthesis pathways among the highest enrichment scores in our datasets (Additional file 4: Table S3). The most striking differences were found in genes related to the formation of intermediates of aromatic amino acid biosynthesis, including 3-dehydroquinate biosynthesis, chorismate biosynthesis via the shikimate pathway (K01626/*aroF*, K01735/*aroB*, K03785/*aroD*, K00800/*aroA*, K01736/*aroC*) and prephenate and 2-oxo-3-phenylpropanoate formation (K14170/*pheA*) (Additional file 6: Figure S2, Additional file 7: Table S4). These intermediates are substrates for a variety of reactions forming, for example, phenylalanine, tyrosine, tryptophan, vitamin E and ubiquinone. No significant differences were observed on a transcript per gene level. Several genes within the methionine biosynthesis pathway (K01739/*metB*, K01760/*metC*, K00549/*metE*) had higher gene and/or transcript abundance in LMY animals, and several of these genes were chosen predictor genes with a negative correlation to methane yield (Additional file 8: Figure S3, Additional file 7: Table S4). Genes involved in lysine (K00674/*dapD*), proline (K00286/*proC*), valine (K01687/*ilvD*) and arginine (K05830/*lysJ*) biosynthesis had greater gene or transcript abundances in LMY animals and were often negatively correlated to methane yield according to sPLS (Additional file 7: Table S4).

### PTS and galactose metabolism

GSEA identified PTS as a highly enriched pathway in the metagenome dataset from LMY animals (Additional file 4: Table S3). WRS and sPLS regression analyses with both metagenome and metatranscriptome data also supported higher abundance of PTS genes in LMY and a negative correlation to methane yield (Additional file 7: Table S4). Genes and/or transcripts involved in the transport of several sugars were significantly more abundant in LMY animals based on WRS, including *N*-acetyl-galactosamine, cellobiose, fructose, glucose, lactose, sorbose, beta-glucosides, galactitol and mannose. With the exception of lactose, and the addition of K02790 (*malX*) for maltose PTS, genes of all these PTSs were selected as predictors in the sPLS regression (with negative coefficients) for methane yield. PTS differences were not observed at the transcript per gene level (Additional file 7: Table S4).

Galactose metabolism was identified by GSEA as significantly enriched in LMY animals in both the metagenome and metatranscriptome datasets (Additional file 4: Table S3). The majority of the genes/transcripts significantly more abundant in LMY animals, or with correlation to methane by sPLS, belonged to PTS systems. However, K01220 (*lacG*) encoding 6-phospho-beta-galactosidase, K00917 (*lacC*) encoding tagatose 6-phosphate kinase and K08302 (*gatY*) encoding tagatose-1, 6-diphosphate aldolase also had significantly different gene and/or transcript abundances. These genes were also identified in sPLS regression analysis as predictor genes for methane yield with negative correlation coefficients (Additional file 7: Table S4). These genes have functions in the degradation of the imported sugars (e.g. lactose) to d-glyceraldehyde-3-phosphate that is subsequently processed via glycolysis (Additional file 9: Figure S4). Significant differences in transcript per gene between HMY and LMY animals were not found for any of the genes.

### Short chain fatty acid metabolism

**Fig. 4:**
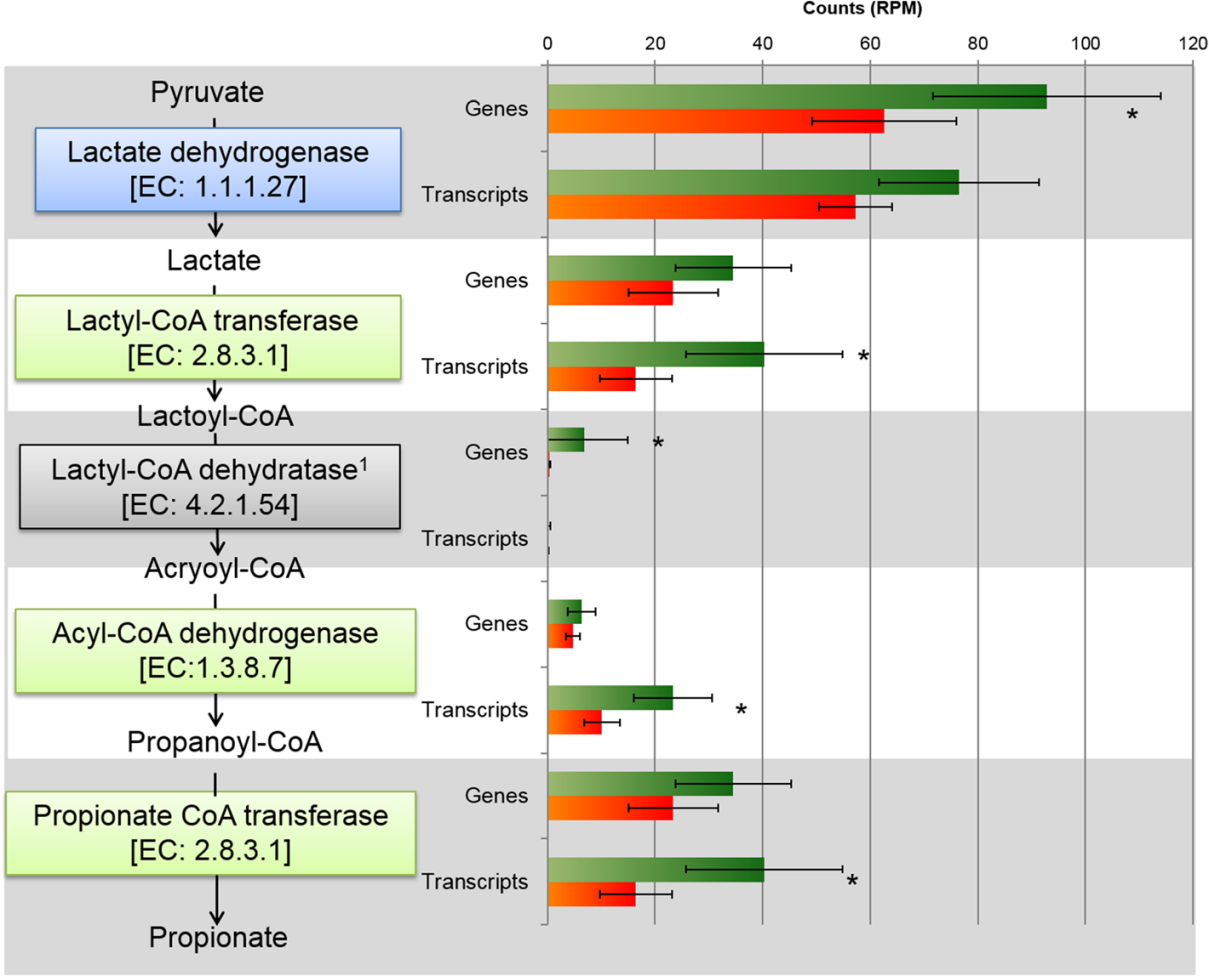
Functions involved in pyruvate fermentation to propionate via lactate production and utilisation via the acrylate pathway in relation to methane yield. *Coloured boxes* indicate that related genes were chosen predictors of methane yield with negative correlation based on sPLS analysis of metagenome (*blue*) or metatranscriptome (*green*) data. *Bar charts* show mean read counts (normalised to RPM) in HMY (*orange*) and LMY (*green*) metagenome (genes) and metatranscriptome (transcripts) datasets. ^1^No reference genes for this function were available in the KEGG database; read mappings were performed based on custom database. *P < 0.05 based on WRS. *Error bars* denote one standard deviation

Many genes involved in short chain fatty acid metabolism were differentially abundant in the metagenome and/or metatranscriptome datasets and were identified as predictor genes in sPLS (Additional file 7: Table S4), and some sub-pathways of special interest to rumen fatty acid metabolism appeared to be related to methane yield. These included genes encoding pyruvate fermentation to lactate and further through to propionate, including the acrylate pathway (Fig. 4, Additional file 7: Table S4). KEGG gene K00016 (*ldh*), which encodes NAD-dependent l (+)-lactate dehydrogenase (l-LDH, EC: 1.1.1.27), had a higher read abundance in LMY animals (*P* < 0.05) and was among the predictor genes with a negative coefficient (−0.0069) in sPLS regression of metagenome data. At the metatranscriptome level, genes involved in the degradation of lactate to propionate showed significantly higher read counts in the LMY animals (Fig. 4, Additional file 7: Table S4). These included genes K00249 (*acd*) encoding acyl-CoA dehydrogenase (EC: 1.3.8.7) and K01026 (*pct*) propionate CoA transferase (EC: 2.8.3.1), both of which were also identified as predictors of methane yield in sPLS based on metatranscriptome data with coefficients of −0.0239 and −0.0146, respectively. The KEGG gene database does not include representative genes for lactoyl-CoA-dehydratase (*lcdA*; EC: 4.2.1.54). Using criteria similar to those used for human gut microorganisms [16], we created a custom dataset based on *lcdA* gene sequences that encode the alpha subunit of lactoyl-CoA-dehydratase, which is considered an indicator gene for propionate production via the acrylate pathway [16]. Metagenome and metatranscriptome read mappings to this dataset revealed significant higher (*P* = 0.02) gene abundance in LMY animals (mean 6.75 reads per million (RPM)), compared to HMY animals (mean 0.37 RPM, Additional file 10: Table S5). Most of the reads mapped to *lcdA* from *Megasphaera* spp., which are known to produce propionate from lactate via the acrylate pathway in the rumen [17]. No differences in *lcdA* transcript or transcript/gene abundances were observed between LMY and HMY animals.

**Fig. 5:**
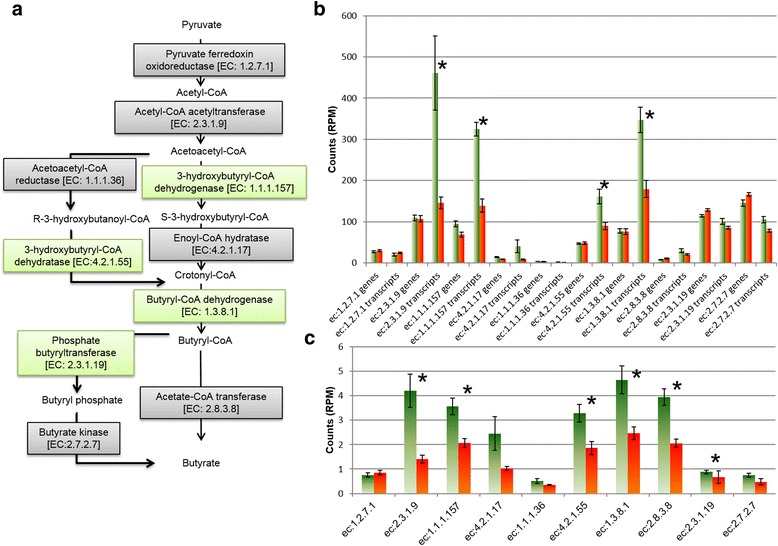
Functions involved in butyrate production from pyruvate or acetyl-coA in relation to methane yield. Schematic overview of functions involved in butyrate production (**a**). *Green boxes* indicate that related genes were chosen predictors of methane yield with negative correlation based on sPLS analysis of metatranscriptome data. *Bar chart* showing mean read counts (normalised to RPM) in high (*orange*) and low (*green*) metagenome (genes) and metatranscriptome (transcripts) data (**b**). Mean read count number (RPM) for butyrate production functions based on transcript per gene for low (*green*) and high (*orange*) methane yield samples (**c**). *P ≤ 0.05 based on WRS. *Error bars* denote standard deviations

Genes encoding the butyrate formation pathway also showed correlations to methane yield (Fig. 5, Additional file 7: Table S4). With the exception of genes K00023 (*phdB*) for acetoacetyl-CoA reductase (EC:1.1.1.36) and K00929 (*buk*) for butyrate kinase (EC:2.7.2.7), all genes encoding the conversion of pyruvate or acetyl-CoA to butyrate showed higher transcripts per gene levels in LMY animals. Many of these genes also had significantly higher transcript counts in LMY animals and were selected by sPLS as predictor genes with negative correlation to methane yield (Fig. 5, Additional file 7: Table S4). No significant differences were observed on the metagenome level, which indicates that differences related to butyrate formation may be directly related to differences in gene expression.

Our data also support a connection between methane yield and genes involved in the formation of butyrate from succinate (Additional file 4: Table S3), which were more abundant in LMY animals or negatively correlated to methane yield. These included genes K00043 (*gbd*) encoding 4-hydroxybutyrate dehydrogenase (EC: 1.1.1.61) and gene K14534 (*abfD*), which encodes the bifunctional enzyme 4-hydroxybutyryl-CoA dehydratase/vinylacetyl-CoA-delta-isomerase (EC: 4.2.1.120/5.3.3.3, Additional file 7: Table S4). The product of this bifunctional enzyme is crotonoyl CoA, which is subsequently transformed to butyrate (Fig. 5a).

### l (+)-lactate dehydrogenase

The elevated abundance of l-LDH genes (K00016, *ldh*, EC: 1.1.1.27) in LMY animals prompted us to investigate these in more detail. We reassembled *ldh*s from the combined metagenome and metatranscriptome data, which resulted in 198 genes with predicted protein lengths ≥310 aa, that were considered near full-length. These genes were analysed phylogenetically against known *ldh*s from rumen bacteria (Additional file 11: Figure S5) and were assigned to 11 rumen *ldh* clusters: *Butyrivibrio* (clusters 1 and 2), *Sarcina* (clusters 1 and 2), *Selenomonas*, Lachnospiraceae, *Olsenella/Clostridium*, *Sharpea/Kandleria*, *Treponema/Ruminococcus*, *Ruminococcus* and *Megasphaera* and one “metagenomic” cluster. Metagenome read mappings to the reassembled *ldh* genes showed differential abundances of 56 genes between HMY and LMY animals (38 more abundant in LMY, 18 more abundant in HMY, Additional file 12: Figure S6). The most differentially abundant *ldh* genes with higher reads counts in LMY animals were genes 2 and 3 (Additional file 12: Figure S6), which associated phylogenetically with *S. azabuensis* (Additional file 12: Figure S6) and gene 1, affiliated with *Kandleria vitulina*. Other *ldh* genes with significantly higher gene or transcript abundance in LMY animals showed phylogenetic affiliation with homologs from the genera *Selenomonas*, *Treponema*, *Ruminococcus*, *Megasphaera* and the *ldh* metagenome-cluster genes (Additional file 11: Figure S5 and Additional file 12: Figure S6). The majority of *ldh* genes with higher gene or transcript abundance in HMY animals showed phylogenetic affiliation with *Sarcina* spp., *Ruminococcus* spp. and *Selenomonas ruminantium*.

Mapping of the metatranscriptome reads showed 15 differently abundant *ldh* transcripts, (3 more abundant in HMY, 12 more abundant in LMY, Additional file 12: Figure S6), whose genes also showed significant differences at the metagenome level but not at a transcript per gene level. Noteworthy was the very high expression of *ldh* gene 7, associated with *Selenomonas ruminantium* (Additional file 11: Figure S5) in two samples from HMY animals (rank 33 and rank 45). These samples had approximately 477 and 276 RPM, respectively, compared to 0.05–6.35 RPM in the other HMY animals, but the reason for these high transcript levels is unknown.

### Mapping of metatranscriptome reads to *Sharpea azabuensis* and *Megasphaera elsdenii* genomes

As described above, 16S rRNA gene amplicon sequencing and general metagenome and metatranscriptome analysis indicated the association of *Sharpea* spp. and *Megasphaera* spp. with LMY animals. We selected two representative rumen isolate genomes for these organisms, *S. azabuensis* DSM20406 and *M. elsdenii* J1, and mapped the metatranscriptome reads from all HMY and LMY sheep back to these genomes to gain an overview of gene transcription in these key species.

For both genomes, significant (*P* ≤ 0.01) differences were observed between the total number of reads mapped to each genome between HMY and LMY animals (Additional file 13: Figure S7). An average of 937 RPM from HMY animals and 11372 RPM from LMY animals mapped to the *S. azabuensis* genome and 0.76 RPM from HMY animals and 2276 RPM from LMY animals mapped to the *M. elsdenii* genome.

From the genes in *S. azabuensis*, which showed gene expression of more than 1 RPM over all samples (2200 genes in total), all were more highly transcribed in LMY samples and for 1811 of these genes the difference was significant (*P* ≤ 0.05). The most highly expressed genes in LMY animals were associated with PTS components for lactose/cellobiose, mannose/fructose/sorbose, glucose, mannose/fructose/*N*-acetylgalactosamine, cellobiose and fructose transport as well as central sugar metabolism/fermentation genes such as glyceraldehyde 3-phosphate dehydrogenase (K00134), and fructose-1,6-bisphosphate aldolase (K01624, Additional file 14: Table S6). *S. azabuensis* lactate dehydrogenase genes were also significantly more highly expressed in LMY animals (*P* < 0.01, Additional file 14: Table S6). Interestingly, the d-lactate dehydrogenase gene was highly expressed with a 12-fold increase in expression in the LMY samples. l-lactate dehydrogenase was also significantly more highly expressed (*P* < 0.01) in LMY samples but at lower abundance, with an average of 0.99 RPM in LMY animals, compared to 0.12 RPM in HMY animals. This matches our results from the *ldh* gene reassembly (l-LDH gene 2) and metatranscriptome read mapping. Among the top ten most highly expressed genes were three genes potentially involved in energy storage through glycogen synthesis, such as glycogen synthase (K00703), and two subunits of glucose-1-phosphate adenylyltransferase (K00975, Additional file 14: Table S6). All of the shikimate pathway genes that were enriched in the LMY animals also had significantly more transcripts mapped to *S. azabuensis* (K01626, K01735, K03785, K00800, K01736, K14170, Additional file 14: Table S6), along with shikimate 5-dehydrogenase (EC:1.1.1.25, K00014) and shikimate kinase (EC:2.7.1.71, K00891). Transcripts from *S. azabuensis* genes encoding lysine (K00674), proline (K00286) and valine (K01687) biosynthesis were also more abundant in LMY animals, but the transcripts from methionine (K01739, K01760, K00549) and arginine (K05830) biosynthetic genes did not differ significantly in abundance between the LMY and HMY animals.

A total of 1046 *M. elsdenii* genes showed gene expression values of ≥1 RPM over all samples, all of which were more highly expressed in LMY animals, and 1012 (96.7 %) of which were not expressed at all in HMY animals. Out of these genes, 122 showed significantly different expression values between HMY and LMY samples (*P* ≤ 0.05) when considering uncorrected *P* values, but none after correction for false discovery rate. High variability of transcript counts between LMY samples was observed, with three LMY samples (ranks 1, 2 and 7) showing high transcript counts (average over all genes of 4–6 RPM) while the remaining LMY samples showed similar transcript read counts to the HMY samples (average over all genes 0.004–0.00007 RPM). On average, the most highly expressed genes with significantly higher abundance in LMY samples (Additional file 15: Table S7) included a lactate permease (IMG gene ID 2628045111, mean expression in LMY animals 11.64 RPM) and a potentially fused gene of a lactate-utilising protein with LutB domain and iron-sulphur binding protein (IMG gene ID 2628045659, mean expression in LMY animals 10.15 RPM). The l-lactate dehydrogenase gene (K00016) was also significantly more expressed in LMY samples at a level of 2.21 RPM. Several genes involved in lactate fermentation to butyrate, rather than to acetate, were highly expressed in LMY samples including genes encoding pyruvate-ferredoxin oxidoreductase, acetyl-CoA C-acetyltransferase, 3-hydroxybutyryl-CoA dehydrogenase, enoyl-CoA hydratase, butyryl-CoA dehydrogenase and acetate CoA transferase (Additional file 15: Table S7, Additional file 16: Figure S8). Metatranscriptome read counts of the indicator gene of the acrylate pathway, lactyl-CoA dehydrogenase (*lcdA*), from *M. elsdenii* (IMG gene ID 2628046107) were low in LMY (mean 0.04 RPM) and showed no significant differences to HMY samples, where no reads mapped to this gene. None of the genes encoding the shikimate pathway or biosynthesis of methionine, lysine, proline, valine or arginine had transcripts that were differentially abundant when mapped to the *M. elsdenii* genome.

## Discussion

Our analyses of the bacterial metagenome and metatranscriptome datasets in this study show clear differences between rumen bacterial communities of HMY and LMY sheep based on community composition, gene abundance and gene expression. The microbiomes of HMY and LMY sheep were previously shown to differ in expression of methanogen genes involved in the hydrogenotrophic methanogenesis pathway [9]. We hypothesised that this was a response of methanogens to the supply of hydrogen in the rumen, which is likely influenced by the fermentation processes of other rumen microbes, which in turn is governed by particle retention time and/or digesta passage rate in sheep [14]. The results from the detailed analysis of the bacterial metagenome and metatranscriptome datasets presented here support the hypothesis that the rumen bacterial fermentation in LMY sheep leads to lower hydrogen formation, thereby causing less methane production by the methanogenic archaea. We have also identified a set of microbial genes whose abundances and expression profiles show strong predictive ability in quantifying methane yield. Microbial DNA sequences correlated with methane yield have recently been reported in cattle [18–21], indicating that such genes could be generally useful as markers to predict methane yield phenotypes across ruminant species.

### Increased abundance of lactate producers in LMY rumen microbiomes

While the microbiomes of HMY animals showed an increased abundance of the families Lachnospiraceae and Ruminococcaceae, the microbiomes of LMY animals showed 10- to 13-fold enrichment of the genus *Sharpea* and accordingly, members of the family Erysipelotrichaceae to which *Sharpea* belongs*.* In LMY animals, 16S rRNA gene sequences assigned to *Sharpea* spp., from either amplicon or metagenome sequencing, represented ~14 % of total bacterial sequences. *S. azabuensis* is a Gram positive, heterofermentative anaerobe, capable of growth on a range of common sugars and produces lactate and CO_2_ from fermentation of glucose [22]. *Sharpea* spp. or family Erysipelotrichaceae 16S rRNA gene sequences have often been retrieved from ruminants. More recently, in an extensive survey of microbial communities in multiple cohorts of sheep with low and high methane yields, Kittelmann et al. [14] reported that the “S” LMY ruminotype was enriched with *S. azabuensis* sequences, among other lactate and succinate producers, representing on average 11.9 % of the bacterial 16S rRNA genes. It was postulated that the “S” ruminotype resulted in less hydrogen being formed from rumen fermentation, which in turn, supported smaller numbers of hydrogenotrophic methanogens and less methane formation. Our observations confirm that the microbial community in the LMY sheep analysed here belong to the “S” ruminotype. The reason for enrichment of *Sharpea* spp. in LMY sheep may be related to the observation that some animals appear to have naturally smaller rumen size, and a higher ruminal turnover rate [3, 23]. It has been hypothesised that a higher rumen turnover rate selects for microorganisms that are capable of fast, heterofermentative growth on soluble sugars, producing less hydrogen, which leads to less methane formation [14]. The definitive experiments linking microbial communities, rumen size and turnover rate directly with methane yield are yet to be conducted, but the association found between *Sharpea* spp. abundance and low methane yield from this study lend weight to this hypothesis. *Sharpea* spp. appear to fulfil this role in LMY sheep, but it is possible that other rumen microorganisms with similar growth and metabolic properties may dominate on other diets or within different ruminant hosts.

### Increased sugar transport and rapid fermentation leads to more lactate production

The proposed fermentation scheme of the “S” ruminotype community in the LMY animals is supported in our study by differences in the abundances of bacterial genes and transcripts encoding for PTSs and galactose metabolism. Individual components of PTSs are commonly found in rumen bacterial genomes, but our analysis of genomes sequenced via the Hungate1000 project (http://genome.jgi.doe.gov/TheHunmicrobiome/) show that the PTS genes most highly enriched in the LMY animals (cellobiose, fructose, glucose and lactose) are particularly abundant in species of *Sharpea, Clostridium*, *Enterococcus* and *Kandleria* and in Erysipelotrichaceae bacterium NK3D112 (Additional file 17: Table S8)*.* Furthermore, mapping the metatranscriptome read data to the *S. azabuensis* genome confirmed higher transcription of several PTS systems in LMY sheep from this organism. It also showed higher transcription level of genes involved in sugar fermentation such as glyceraldehyde-3-phosphate dehydrogenase and fructose-1,6-bisphosphate aldolase (Additional file 14: Table S6), further supporting the theory of rapid sugar processing by this organism. The enrichment of cellobiose- and glucose-specific PTS transporters is consistent with the degradation of the fibre component of the sheep lucerne diet, via the action of cellulases and cellobiases that generate cellobiose and glucose, respectively. The elevated level of fructose-specific PTS transporters also makes biological sense, as sucrose (a disaccharide of fructose and glucose) is a major component of the soluble carbohydrates found in lucerne [24]. However, the occurrence of lactose PTS transporters is surprising, as lactose is not a sugar produced by plants. It is known that genes annotated as lactose-specific PTS transporters in *Lactococcus lactis* [25] and *Streptococcus gordonii* [26] actually mediate galactose transport, thus it is probable that the lactose-specific PTS transporters (*lacEF*) identified in the rumen actually encode galactose PTS transporters. The elevated gene abundance of tagatose-6-phosphate pathway genes in LMY in our study that are involved in galactose metabolism supports this idea. Galactose is a significant sugar found in lucerne, making up ~1.5 and 1.4 % of the cell wall monomer composition of leaf and stem fractions, respectively [27].

Under conditions of rapid sugar fermentation in a high flux system, bacteria also need to synthesise more cellular components to keep pace with increased growth requirements. This necessitates the generation of more reducing potential to drive cellular reactions, such as fatty acid synthesis, and the production of more amino acids and nucleic acids to support increased bacterial growth. This scenario is consistent with the enrichment in the LMY animals of genes involved in the shikimate pathway which links carbohydrate metabolism to the synthesis of the aromatic amino acids, tyrosine, phenylalanine and tryptophan, as well as to the formation of co-enzymes and vitamins. In bacteria, this pathway is tightly regulated by direct metabolite feedback inhibition and/or by repression at the genetic level [28–30] to control the energetically expensive synthesis of aromatic compounds. Regulation of the pathway is also exerted via a metabolic control where the rate of enzyme synthesis is related to the growth rate of the cell [31]. Therefore, increased expression of shikimate pathway genes is a strong indicator that the LMY rumen microbiome is directing metabolism towards increased anabolic processes to support faster microbial growth. The mapping of shikimate pathway gene transcripts enriched in the LMY animals to the *S. azabuensis* genome is also an indication that these organisms are important contributors to this anabolic process.

Phylogenetic, as well as metagenomic and metatranscriptomic data, indicated an increased abundance and activity of *Sharpea* spp. Consequently, rapid fermentation of sugars released from lucerne in the LMY rumen would be expected and would result in the production of lactate and CO_2_. Lactate is formed as a means of quickly dumping reducing equivalents under conditions of rapid glycolytic flux [32]. Lactate is not a major product of rumen fermentation under normal conditions, but when animals are fed a carbohydrate-rich diet with high levels of soluble sugars, lactate can accumulate [33]. Our VFA data did not show a significant difference in lactate concentration between LMY and HMY animals but suggest a trend towards higher lactate concentration in LMY animals (Fig. 1), which supports the theory of rapid fermentation and lactate formation in these animals. Lactate can be formed via the action of two different forms of NAD-linked LDHs (nLDHs); one produces l (+)-lactate (LnLDH, EC 1.1.1.27) while the other (DnLDH, EC 1.1.1.28) produces d (−)-lactate. In the metagenome datasets, only the LnLDH genes were differentially abundant in the LMY animals, and phylogenetic analysis of their amino acid sequences showed that some of the most abundant LnLDH genes were associated with *Sharpea* spp*.* and with *Kandleria* spp., other potential lactate producers closely related to *Sharpea* [34]. Mapping the metatranscriptome reads to the *S. azabuensis* genome showed higher expression of the DnLDH gene in this organism which suggests increased production of lactate by *Sharpea* organisms in LMY animals comes from both LnLDH and DnLDH activity.

### Increased lactate conversion to propionate and butyrate in LMY animals

High lactate production in the rumen is known to reduce rumen pH and select for lactate-utilising organisms [35]. The observations of a 17-fold enrichment of 16S rRNA and *ldh* (l-LDH gene 28) genes, as well as a significant increase of transcripts from lactate-utilising *Megasphaera* spp. in LMY animals (Additional file 13: Figure S7), are consistent with this expectation. *M. elsdenii* is considered to be the main fermenter of lactate in the rumen, accounting for up to 74 % of the lactate fermentation in the rumen of dairy cattle [17]. Its relative abundance in the bacterial community is also known to increase under conditions of rapid sugar fermentation and lactic acidosis [36] and *Megasphaera* was one of the two genera found to be more abundant in low residual feed intake (efficient) dairy cows [37]. Lactate permease and a gene encoding a potential lactate utilisation protein were found among the most highly transcribed genes based on metatranscriptome read mappings to the *M. elsdenii* genome (Additional file 15: Table S7). Together with the l-lactate dehydrogenase, these genes appear to be involved in lactate uptake and processing to pyruvate. This metabolism of lactate by *Megasphaera* is likely to explain why there was no shift in the pH of the LMY rumen samples.

*M. elsdenii* produces propionate via the acrylate pathway, as well as producing acetate, butyrate, valerate, and traces of caproate from various reactions involving acetyl-CoA derived from lactate oxidation via pyruvate [17]. Genes encoding the acrylate pathway were reported to be enriched in the microbiomes of efficient dairy cows, and many of these genes were annotated as being from *M. elsdenii* [37]. In the total metagenome and metatranscriptome data from the current study, KEGG genes of the acrylate pathway showed significantly more abundance in LMY animals at both gene and transcript abundance levels (Fig. 4), as did genes involved in the transformation of pyruvate to butyrate on transcript and transcript per gene level (Fig. 5). However, fermentation to butyrate seems the more likely pathway used by *M. elsdenii* in the LMY animals, as the indicator gene for the acrylate pathway, lactyl-CoA dehydrogenase (*lcdA*), was only significantly more abundant in LMY animals on the metagenome level. Furthermore, direct mapping of the metatranscriptome data to the *M. elsdenii* genome showed that this gene was not highly transcribed. The remaining genes of the acrylate pathway are also involved in other pathways and are not useful for predicting propionate formation. Instead, fermentation to butyrate seems more likely, considering the high metatranscriptome read counts of genes involved in this pathway (Additional file 16: Figure S8). These results fit well with a current model for lactate utilisation by *M. elsdenii* under steady state conditions where lactate is converted predominantly to butyrate (54 %) with some acetate formation (12 %) and no propionate formation [38]. Several *ldh* genes with strong abundance and transcriptional activity in LMY animals fell into a separate phylogenetic cluster (“metagenomic cluster”) with no closely related reference *ldh* genes (Additional file 11: Figure S5 and Additional file 12: Figure S6). This indicates that some potentially important players involved in lactate production and utilisation in the rumen are not yet identified and their metabolic end products or impact on hydrogen and methane formation are not known.

Lactate conversion to butyrate, instead of to propionate, produces 2 mol of hydrogen per hexose, which could produce 0.5 mol of methane via the hydrogenotrophic pathway. A direct fermentation of hexoses to butyrate and acetate by, for example, members of the Ruminococcaceae would produce 2.66 mol of hydrogen and allow 0.66 mol of methane to be formed [13]. Thus, lower hydrogen production via the lactate to butyrate pathway would decrease methane production by 24 % and provides an explanation for the lower methane yield in animals with the “S”-type microbiome.

The preceding analyses indicate that the rumens of LMY sheep support a rapid fermentation by *Sharpea,* producing lactate, which is converted to mainly butyrate by *Megasphaera*. One might therefore expect increased lactate and butyrate concentrations in the rumen of LMY animals. Measurement of fermentation acids did not reveal any trend towards increased production of butyrate in the LMY animals, but a high degree of variability of lactate concentrations was observed between LMY samples, and the trend was towards higher lactate in the LMY samples. Our bacterial abundance and gene expression data predict that lactate production in the LMY rumen is balanced with lactate utilisation, such that significant differences in lactate concentrations compared to the HMY rumen are not observed. Butyrate is absorbed by the rumen epithelium where it is converted to β-hydroxybutyrate and acetoacetate and used as energy substrates for the epithelial cells. It is well known that butyrate stimulates rumen development [39] and that butyrate infused into the rumen causes papillary growth [40]. In dairy cattle, the rate of butyrate absorption from the rumen increases with increasing butyrate concentration, and a smaller rumen volume results in higher butyrate absorption [41]. Therefore, we propose that increased production of butyrate in LMY animals is balanced by greater absorption of butyrate across the rumen epithelium. Further experiments are required to examine the flux of both lactate and butyrate in the rumen of LMY animals to confirm these predictions.

## Conclusions

**Fig. 6:**
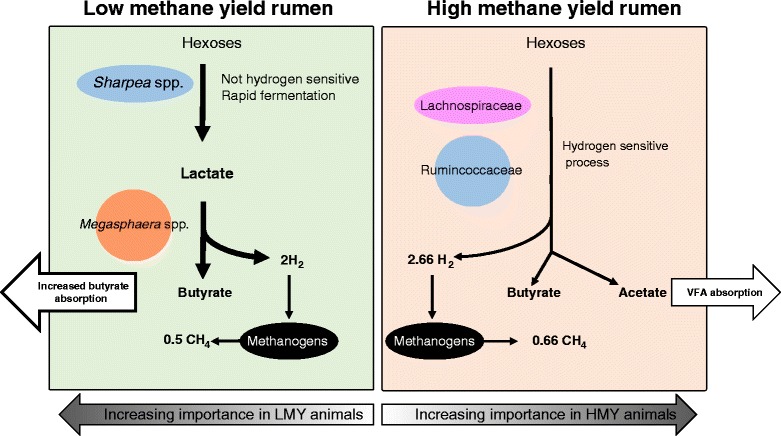
Schematic concept of bacterial processes influencing hydrogen and methane formation in low and high methane yield animals according to results of this study

The amplicon, metagenome and metatranscriptome data analysed in this study demonstrated strong evidence of a *Sharpea*-enriched, “S”-type bacterial community associated with LMY sheep. There is a clear pattern of gene and transcript abundance reflecting rapid heterofermentative growth in the rumen with lactate formation and subsequent metabolism to butyrate. These differences are consistent with a smaller rumen and a higher rate of digesta turnover in LMY animals, leading to a microbiome that produces less hydrogen and therefore less methane. In contrast, the HMY animals show less enrichment of specific bacterial taxa and maintain communities similar to those commonly found in other ruminants. Based on these results, we present a concept to explain the differences in bacterial communities and how they influence methane formation in the LMY and HMY animal cohorts (Fig. 6). In this concept, the community structure of the HMY rumen has higher abundance of members of the Ruminococcaceae and Lachnospiraceae, while the LMY community is enriched in Erysipelotrichaceae, especially *Sharpea* spp. We propose that this community shift is caused by physical differences in rumen size and turnover rate, where the smaller, faster turnover, LMY rumen selects for rapid bacterial fermenters, such as *Sharpea* spp. The higher abundance of *Sharpea* spp. is accompanied by increased lactate production and by a corresponding increase in conversion of lactate to butyrate by *Megasphaera* spp. These community differences result in a fermentation shift, from fermentation of hexoses to butyrate and acetate (mediated by organisms belonging to the family Ruminococcaceae in HMY animals), to fermentation of hexoses to butyrate only, via a two-step process (involving *Sharpea* spp. and *Megasphaera* spp., in LMY animals). The one-step HMY fermentation is predicted to generate 2.66 mol of hydrogen per mol hexose and 0.66 mol of methane, while the two-step fermentation in LMY animals gives 2 mol of hydrogen leading to 0.5 mol methane. Thus, it is predicted that the LMY rumen would produce approximately 24 % less methane. Of course, these pathways do not represent all of the fermentation occurring, and so, the overall methane difference is smaller. The demonstration of distinctly different rumen microbiomes between LMY and HMY animals supports the notion that the methane yield phenotype in sheep is a repeatable and heritable trait which can be selected in breeding programmes [42, 43]. Genetic and phenotypic correlations of methane outputs with various production traits in sheep (weaning weight, live weight at 8 months of age, dag score, muscle depth, and fleece weight at 12 months of age) have been measured [5] to establish whether selecting for LMY in sheep will be beneficial from an animal production point of view. Most of the correlations with production traits were weak and not significantly different from zero, but for fleece weight, the correlation estimates suggest a low economically favourable relationship. Therefore, in conjunction with methane yield measurements, rumen microbiome characterisation will be a helpful screening tool for selecting low methane emitting sheep.

## Methods

### Methane yield measurements and sampling

The sampling of rumen contents from sheep, data processing and analysis have been described previously [9]. Methane yield measurements (in g methane/kg dry matter intake DMI) were made on 23 animals, after adaptation to a pelleted lucerne diet, on two occasions in June 2011 using open circuit respiration chambers within the New Zealand Methane Measurement Centre at AgResearch Grasslands, Palmerston North. The animals were ranked based on methane yield and classified as HMY (4 animals), LMY (4 animals) or IMY (15 animals) methane yield animals (Additional file 1: Table S1). Rumen content samples were collected from each animal 4 h after feeding via stomach intubation on the morning following the completion of each methane measurement, except for one IMY animal (rank 19), for which rumen content samples could only be retrieved at one time point. Analysis of volatile fatty acids was conducted on all 45 samples. DNA and RNA were extracted from each sample and used for data generation as follows. DNA amplicon sequencing was conducted for all 45 samples, metagenomics and metatranscriptomic sequencing was conducted on the samples from 10 sheep at the two time points (20 samples total) including four LMY, four HMY and two IMY animals (Additional file 1: Table S1). The sequencing produced an average of 217 million metagenome jointed reads (51 Gb) per sample and 35 million metatranscriptome reads (7 Gb) per sample [9]. For detailed information on analysis of fermentation acids, 16S rRNA gene amplicon sequencing and analysis, processing of metagenome and metatranscriptome data and metagenome and metatranscriptome read based annotation, please refer to the supplementary materials (Additional file 5: Text S1). All read mapping analyses, to either the KEGG database, newly assembled genes in this study or reference genomes, were conducted using artefact and rRNA filtered, merged metagenome and metatranscriptome 2 × 150 bp paired end reads from metagenome and metatrancriptome data (Additional file 1: Table S1 and Additional file 5: Text S1).

### Statistical data analyses

We used three methods of statistical analysis to compare results from categorical (HMY, LMY) data based on individual genes (WRS) and pathways (GSEA) and direct correlations to methane yield via sPLS regression. All statistical analyses were conducted using RPM-normalised read count matrices. Differential gene abundance and expression on a pathway level was estimated by GSEA [44], using read count data from HMY or LMY animals only (*n* = 16) based on KEGG pathways. Using the desktop application GSEA-P [44], we pre-ranked genes by a signal-to-noise metric score and estimated normalised enrichment score (NES), nominal *P* values and false discovery rates (FDR) by permuting phenotypes 10,000 times. Initial gene selection of methane predictors were assessed using sPLS regression of methane yield on KEGG genes [45, 46], using the optimum sparsity tuning parameter (eta) and number of hidden components (K) predicted in the mean squared prediction error plot (MSPE) for each dataset. The 95 % confidence intervals of the coefficient for each selected KEGG gene were estimated and predictors with intervals shifting from positive to negative correlation or vice versa were excluded from the set of predictor KEGG genes. We then estimated the correlations between the chosen specific KEGG genes and methane yield. We also manually compared the selected KEGG gene sets of the sPLS regression analysis of each dataset with the results of categorical statistical analysis using WRS with 10,000 permutations [9] and the pathways enrichment scores from the GSEA. We verified whether the two different sampling time points had an influence on the gene or transcript abundance values and their association to the methane yield phenotype for the four HMY and LMY animals for our selected subsets of genes (Additional file 7: Table S4) and whether these difference were significant, using one-way ANOVA and WRS. Here, we used a “repeated measures ANOVA” via a general linear mixed model framework treating animals as random effects. Our results showed that for the majority (491 out of 496) of the KEGG genes these differences were not significant with *P* > 0.05 in either test and for the remaining five KEGG genes (K01220, K02787, K02796, K00158, and K00772) the differences between sampling days did not outweigh difference related to methane yield group (e.g. all genes remained more or less abundant in the respective methane yield phenotypes). We therefore focused all analyses on comparisons between methane yield groups or direct correlation to methane yield, only.

### Read extraction, assembly and phylogenetic analysis of l-lactate dehydrogenase (*ldh*) genes

For phylogenetic assignment, *ldh* genes were reassembled based on raw reads and contigs from existing assemblies, with hits to KEGG gene K00016 from both metagenome and metatranscriptome data (see Additional file 5: Text S1) and *ldh* genes with a protein length of ≥310 aa were considered near full-length and included into the phylogenetic analysis. Reference *ldh* sequences from rumen bacterial isolates with hits to K00016 were extracted from the IMG/M database in June 2015. Amino acid sequences were aligned using MUSCLE [47], and alignments were imported into ARB (v.6) [48] for manual refinement. Phylogenetic maximum likelihood bootstrap trees with 100 re-samplings were constructed using RAxML (v.7.7.2) [49], and the best scoring tree including bootstrap values was re-imported into ARB for cluster annotation.

### Read mapping to reference genomes from rumen isolates and reassembled *ldh* and *lcdA* database genes

Reference genome sequences and gene annotations from the rumen isolates *S. azabuensis* DSM20406 and *M. elsdenii* J1 were obtained from the Department of Energy Joint Genome Institute Genome portal [50]. Metatranscriptome reads of each HMY and LMY sample were mapped to the two reference genomes as well as metagenome and metatranscriptome reads to all reassembled *ldh* genes and all genes in the custom *lcdA* genes database (for information on database construction, see Additional file 5: text S1) using BBmap (http://sourceforge.net/projects/bbmap/) with an ID cut-off of 98 % sequence similarity for *ldh* genes and genome sequences, and 60 % sequence similarity for *lcdA* genes and counting ambiguous reads for all matching genes. Read counts were normalised to RPM, and statistical analysis of normalised read counts was conducted in R via the WRS test and Benjamini-Hochberg correction (for all genes in isolate genomes and *ldh* genes) to select genes or transcripts with significantly different abundances between the HMY and LMY animals.

### Functional comparison to the Hungate 1000 genomes

Functional identifiers of KEGG orthology genes from the metagenome dataset that showed significant correlation to methane yield in both the WRS test and sPLS analyses were uploaded into IMG/MER and used as screening IDs for all the bacterial genomes available from the Hungate 1000 project (http://genome.jgi.doe.gov/TheHunmicrobiome/) and all additionally available bacterial genomes derived from rumen habitats in June 2015 using the “functions versus genomes” tool in IMG/MER.

## Additional files

Additional file 1: Table S1. Overview of samples analysed in this study and methods of analysis conducted. (PDF 223 kb)
Additional file 2: Figure S1. Sparse partial least squares regression analysis (sPLS) of gene (A) or transcript (B) abundances correlated with animal methane yield plotting low (green), intermediate (blue) and high (red) methane yield animals based on gene abundance or expression values of selected predictor genes. (TIF 168 kb)
Additional file 3: Table S2. KEGG genes from metagenome (DNA) and metatranscriptome (RNA) data commonly identified in sparse partial least squares analysis (sPLS) and Wilcoxon rank sum test (WRS) correlated to methane yield/significantly differentially abundant between high and low methane yield sheep. (PDF 507 kb)
Additional file 4: Table S3. Gene set enrichment analysis of metagenome and metatranscriptome datasets. Pathways with genes differentially present or expressed based on nominal *P* value (NOM *P* val ≤0.05) are shown, ranked by normalised enrichment score (NES). Corrected *P* values for false discovery rate (FDR *q* val) and familywise-error rate (FEWER *P* val) are shown. (PDF 88 kb)
Additional file 5: Text S1. Detailed results for the statistical analysis and additional methods. (DOCX 40 kb)
Additional file 6: Figure S2. Functions involved in synthesis of aromatic amino acid precursors in relation to methane yield. Green boxes indicate that related genes were chosen as predictors of methane yield with negative correlation based on sPLS analysis of metatranscriptome data. The bar chart shows mean read counts (normalised to RPM) in high (orange) and low (green) metagenome (genes) and metatranscriptome (transcripts) data. **P* < 0.05 based on WRS. Error bars denote standard deviations. (TIF 268 kb)
Additional file 7: Table S4. Statistical data of KEGG genes related to low or high methane yield animals for Wilcoxon rank sum test (WRS) and sparse partial least squares analysis (sPLS). (PDF 1543 kb)
Additional file 8: Figure S3. Functions involved in methionine biosynthesis in relation to methane yield. Blue shaded functions indicate that related genes were chosen predictors of methane yield with negative correlation based on sPLS analysis of metagenome data. Bar chart shows mean read counts (normalised to RPM) in high (orange) and low (green) metagenome (genes) and metatranscriptome (transcripts) data. **P* < 0.05 based on the WRS test. Error bars denote standard deviations. (TIF 185 kb)
Additional file 9: Figure S4. Lactose degradation functions correlated with methane yield. Coloured boxes indicate that the corresponding genes were predictors of methane yield with negative correlation based on sPLS analysis of metagenome (blue) or metagenome and metatranscriptome (purple) data. Bar charts show mean read counts (normalised to RPM) in high (orange) and low (green) metagenome (genes) and metatranscriptome (transcripts) data. **P* < 0.05 based on WRS. Error bars denote standard deviations. (TIF 189 kb)
Additional file 10: Table S5. Read mapping statistics of metagenome and metatranscriptome data for lcdA genes. (PDF 83 kb)
Additional file 11: Figure S5. Phylogenetic maximum likelihood tree of lactate dehydrogenase genes based on a multiple sequence alignment including sequences >310aa from the metagenomic reassembly and reference sequences from selected rumen microorganisms. Bootstrap support of ≥75 % is illustrated by open and ≥90 % by filled circles. Colour shadings mark genes with significantly (*P* ≤ 0.05) more abundance in low metagenome (green), low metagenome and metatranscriptome (blue), high metagenome (orange) and high metagenome and metatranscriptome (red) samples. Reference sequences of lactate dehydrogenase genes from microbial isolates are shown with the relevant strain name and the 10-digit gene identifier in the IMG database. The out-group consisted of 14 lactate dehydrogenase amino acid sequences from protists. The scale bar represents 0.4 % sequence divergence. (TIF 1161 kb)
Additional file 12: Figure S6. Barplot showing metagenome (A) and metatranscriptome (B) read count numbers based on read mappings against reassembled rumen metagenome lactate dehydrogenase genes (K00016). Shown are genes with significantly different read count abundance (*P* ≤ 0.05) between low (green) and high (red) methane samples based on Wilcoxon rank sum tests and Benjamini-Hochberg correction. Metatranscriptome data for gene 7 was excluded due to unusually high expression values in high methane yield sheep (see the “Results” section). (TIF 72 kb)
Additional file 13: Figure S7. Total metatranscriptome reads mapped to *Sharpea azabuensis* DSM20406 and *Megasphaera elsdenii* J1 genomes in high (red) and low (green) methane yield sheep. Whiskers (error bars) above and below the box indicate the 90th and 10th percentiles; ** indicate significant difference (*P* ≤ 0.01, based on Wilcoxon rank sum test) between total RPM metatranscriptome reads mapped to the genome in high and low methane yield animals. (TIF 709 kb)
Additional file 14: Table S6. Differentially expressed genes of *Sharpea azabuensis* DSM20406 in low methane yield sheep according to metatranscriptome read mapping. (DOCX 25 kb)
Additional file 15: Table S7. Top 50 most highly and significant differentially expressed genes of *Megasphaera elsdenii* J1 in low methane yield sheep according to metatranscriptome read mapping. (DOCX 17 kb)
Additional file 16: Figure S8. Overview of pyruvate degradation functions correlated with methane yield in *Megasphaera elsdenii* J1 (A). Bar charts show mean read counts (normalised to RPM) in high (orange) and low (green) metatranscriptome (transcripts) data. **P* < 0.05 based on WRS (B). (TIF 191 kb)
Additional file 17: Table S8. KEGG functions from metagenome data commonly identified in Wilcoxon rank sum test and sparse partial least squares analysis as related to methane yield against their occurrence in Hungate 1000 and other rumen-derived genomes available in the IMG database in June 2015. (XLSX 204 kb)
