# Supplementary material for: Rumen metagenome and metatranscriptome analyses of low methane yield sheep reveals a Sharpea-enriched microbiome characterised by lactic acid formation and utilisation
Source: Microbiome. 2016 Oct 19;4:56. doi: 10.1186/s40168-016-0201-2 (PMC5069950; doi:10.1186/s40168-016-0201-2)
Supplement: Additional file 1: Table S1. — Overview of samples analysed in this study and methods of analysis conducted. (PDF 223 kb) [file 40168_2016_201_MOESM1_ESM.pdf]

Table S1. Overview of samples analysed in this study and methods of analysis conducted.

Rank CH_4	Sheep tag	Sampling date	Class	CH_4 yield	pH	VFA	16S	Meta genome	Meta transcriptome
1	S1494	13.06.11	low	10.03	6.41	●	●	●	●
2	S1283	13.06.11	low	10.37	6.41	●	●	●	●
3	S1133	28.06.11	intermediate	11.17	6.21	●	●		
4	S1435	28.06.11	low	11.38	6.75	●	●	●	●
5	S1494	28.06.11	low	11.48	6.07	●	●	●	●
6	S1265	13.06.11	low	11.78	6.69	●	●	●	●
7	S1435	13.06.11	low	11.91	6.43	●	●	●	●
8	S1283	28.06.11	low	12.23	6.01	●	●	●	●
9	S1636	28.06.11	intermediate	12.28	6.79	●	●		
10	S1265	28.06.11	low	12.35	6.25	●	●	●	●
11	S1142	28.06.11	intermediate	12.37	6.67	●	●		
12	S1363	28.06.11	intermediate	12.49	6.4	●	●	●	●
13	S1111	13.06.11	intermediate	12.56	6.35	●	●	●	●
14	S1638	28.06.11	intermediate	13.14	6.47	●	●		
15	S1253	13.06.11	intermediate	13.28	6.24	●	●		
16	S1248	28.06.11	intermediate	13.37	6.93	●	●		
17	S1142	13.06.11	intermediate	13.41	6.6	●	●		
18	S1277	13.06.11	intermediate	13.52	6.77	●	●		
20	S1248	13.06.11	intermediate	13.58	6.36	●	●		
21	S1243	28.06.11	intermediate	13.74	6.41	●	●		
22	S1636	13.06.11	intermediate	13.75	6.2	●	●		
23	S1133	13.06.11	intermediate	13.83	6.35	●	●		
24	S1423	28.06.11	intermediate	13.88	6.44	●	●		
25	S1448	28.06.11	intermediate	14.11	6.35	●	●		
26	S1243	13.06.11	intermediate	14.19	6.25	●	●		
27	S1172	28.06.11	intermediate	14.26	6.11	●	●		
28	S1638	13.06.11	intermediate	14.38	6.47	●	●		
29	S1448	13.06.11	intermediate	14.39	6.46	●	●		
30	S1277	28.06.11	intermediate	14.43	6.37	●	●		
31	S1423	13.06.11	intermediate	14.45	6.46	●	●		
32	S1679	28.06.11	intermediate	14.61	6.83	●	●		
33	S1234	28.06.11	high	14.71	6.25	●	●	●	●
34	S1111	28.06.11	intermediate	14.92	6.82	●	●	●	●
35	S1679	13.06.11	intermediate	15.05	6.61	●	●		
36	S1363	13.06.11	intermediate	15.11	6.25	●	●	●	●
37	S1172	13.06.11	intermediate	15.22	6.32	●	●		
38	S1174	13.06.11	high	15.55	6.08	●	●	●	●
39	S1234	13.06.11	high	15.62	6.47	●	●	●	●
40	S1266	28.06.11	intermediate	15.66	6.55	●	●		
41	S1266	13.06.11	intermediate	15.68	6.33	●	●		
42	S1586	28.06.11	high	15.72	6.44	●	●	●	●
43	S1586	13.06.11	high	15.84	6.4	●	●	●	●
44	S1333	13.06.11	high	16.14	6.35	●	●	●	●
45	S1174	28.06.11	high	16.32	6.5	●	●	●	●
46	S1333	28.06.11	high	16.91	6.45	●	●	●	●
