# Supplementary figures and images for: Rumen metagenome and metatranscriptome analyses of low methane yield sheep reveals a Sharpea-enriched microbiome characterised by lactic acid formation and utilisation
Source: Microbiome. 2016 Oct 19;4:56. doi: 10.1186/s40168-016-0201-2 (PMC5069950; doi:10.1186/s40168-016-0201-2)

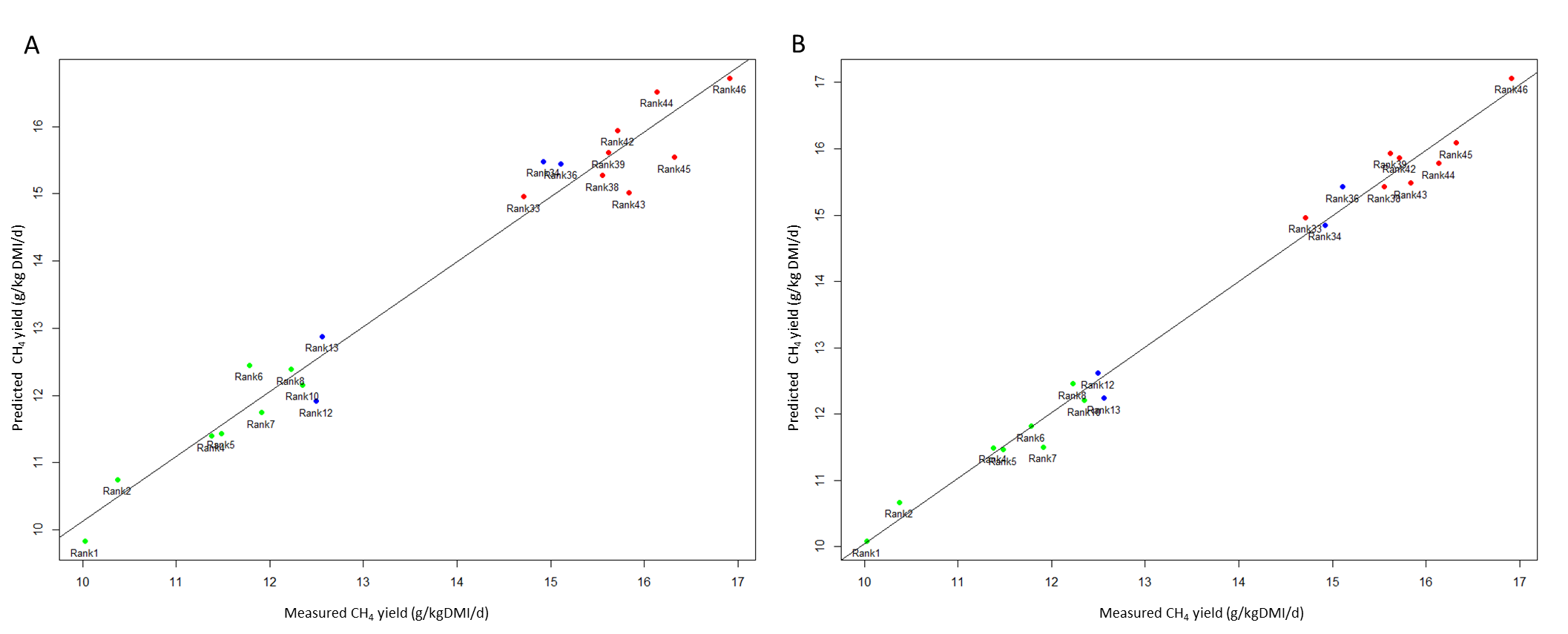

Supplement: Additional file 2: Figure S1. — Sparse partial least squares regression analysis (sPLS) of gene (A) or transcript (B) abundances correlated with animal methane yield plotting low (green), intermediate (blue) and high (red) methane yield animals based on gene abundance or expression values of selected predictor genes. (TIF 168 kb) [file 40168_2016_201_MOESM2_ESM.tif]

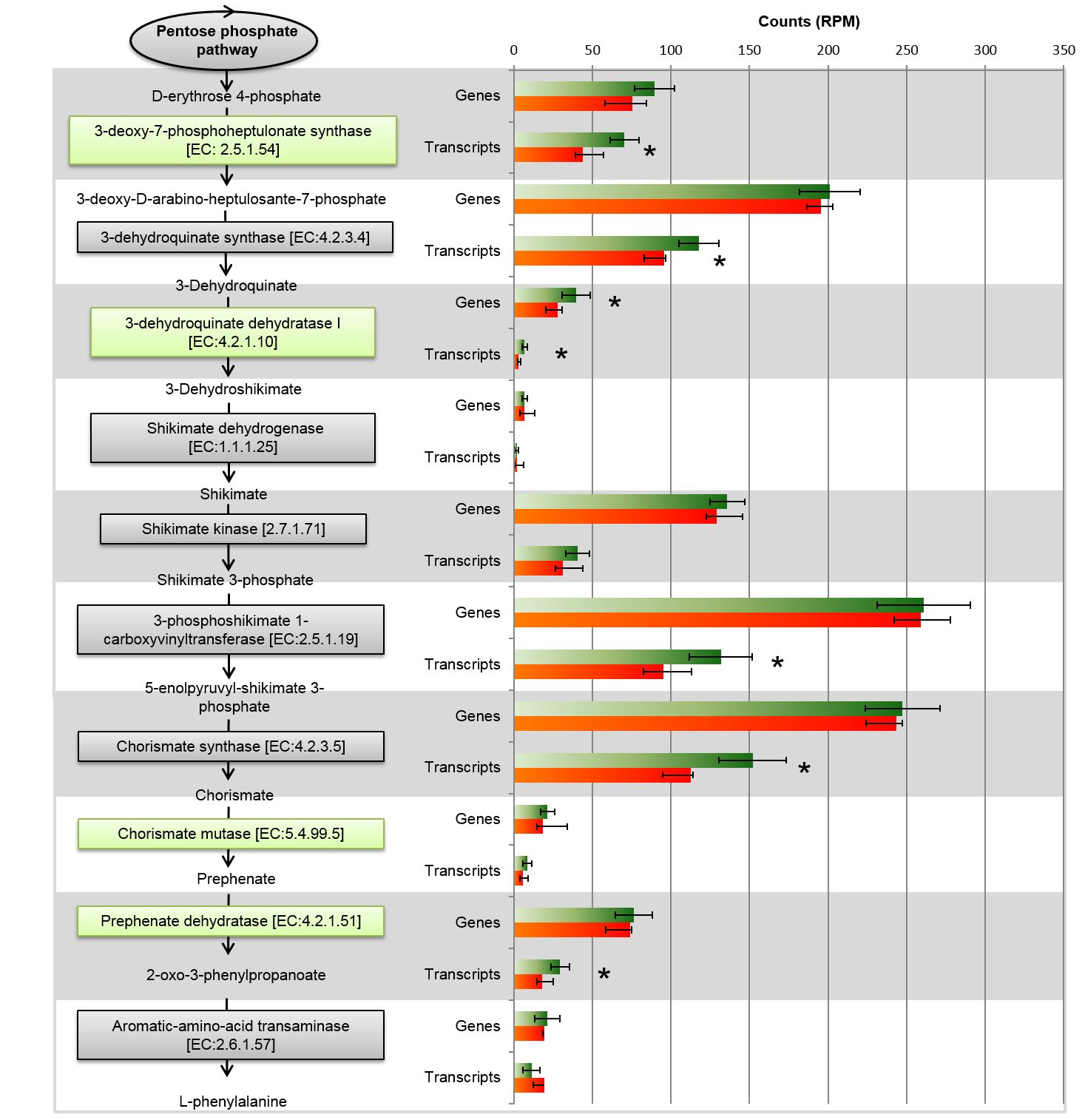

Supplement: Additional file 6: Figure S2. — Functions involved in synthesis of aromatic amino acid precursors in relation to methane yield. Green boxes indicate that related genes were chosen as predictors of methane yield with negative correlation based on sPLS analysis of metatranscriptome data. The bar chart shows mean read counts (normalised to RPM) in high (orange) and low (green) metagenome (genes) and metatranscriptome (transcripts) data. *P < 0.05 based on WRS. Error bars denote standard deviations. (TIF 268 kb) [file 40168_2016_201_MOESM6_ESM.tif]

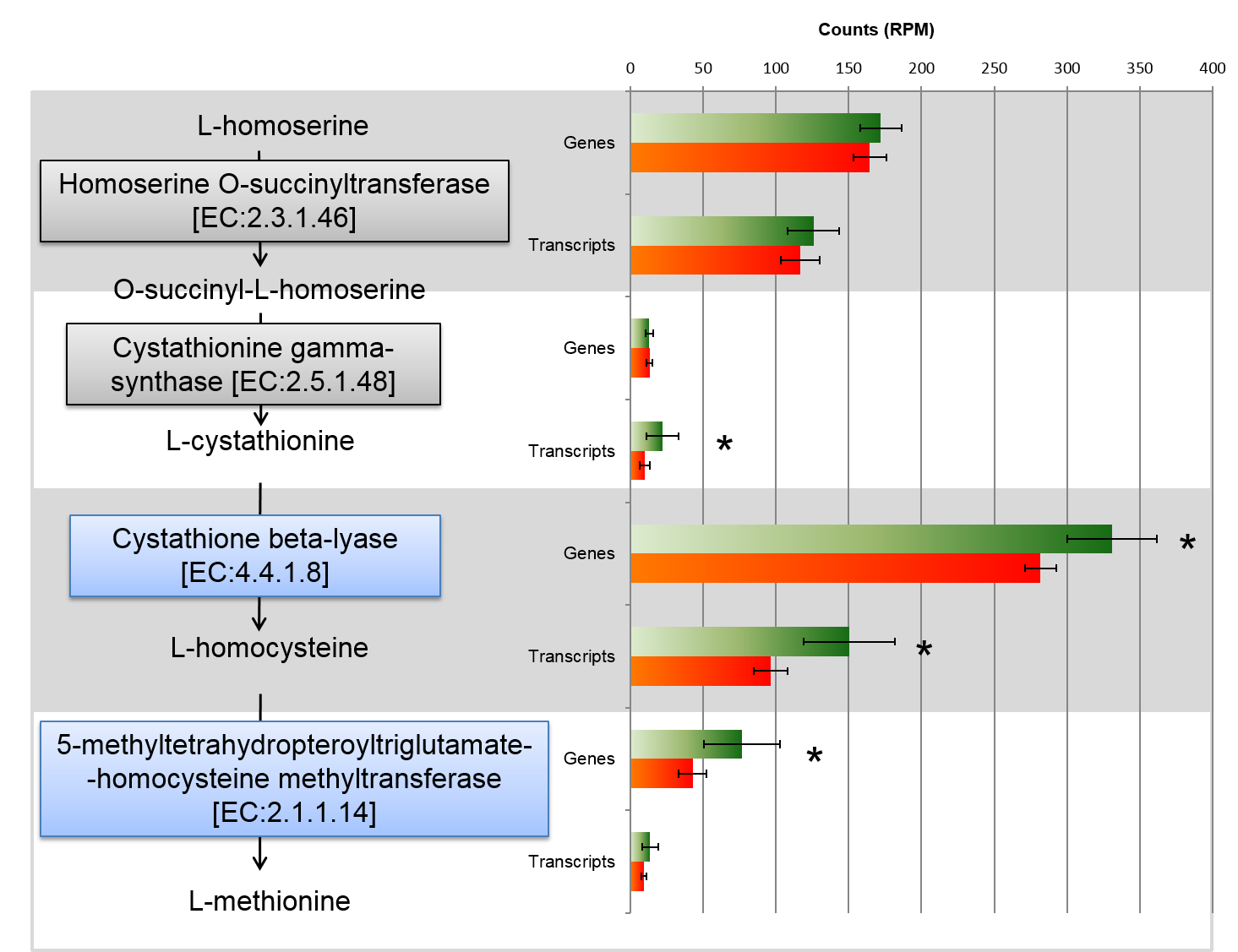

Supplement: Additional file 8: Figure S3. — Functions involved in methionine biosynthesis in relation to methane yield. Blue shaded functions indicate that related genes were chosen predictors of methane yield with negative correlation based on sPLS analysis of metagenome data. Bar chart shows mean read counts (normalised to RPM) in high (orange) and low (green) metagenome (genes) and metatranscriptome (transcripts) data. *P < 0.05 based on the WRS test. Error bars denote standard deviations. (TIF 185 kb) [file 40168_2016_201_MOESM8_ESM.tif]

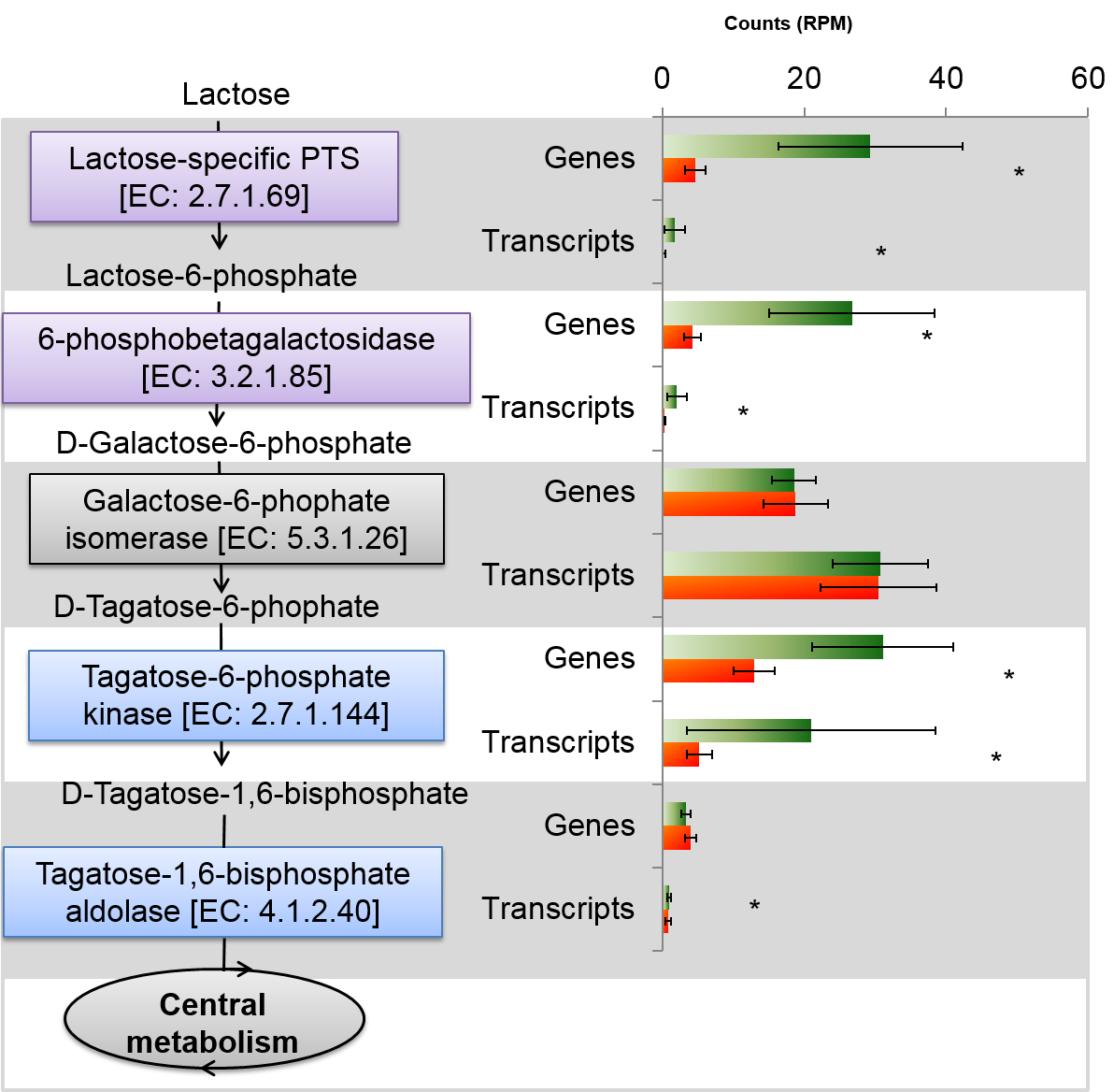

Supplement: Additional file 9: Figure S4. — Lactose degradation functions correlated with methane yield. Coloured boxes indicate that the corresponding genes were predictors of methane yield with negative correlation based on sPLS analysis of metagenome (blue) or metagenome and metatranscriptome (purple) data. Bar charts show mean read counts (normalised to RPM) in high (orange) and low (green) metagenome (genes) and metatranscriptome (transcripts) data. *P < 0.05 based on WRS. Error bars denote standard deviations. (TIF 189 kb) [file 40168_2016_201_MOESM9_ESM.tif]

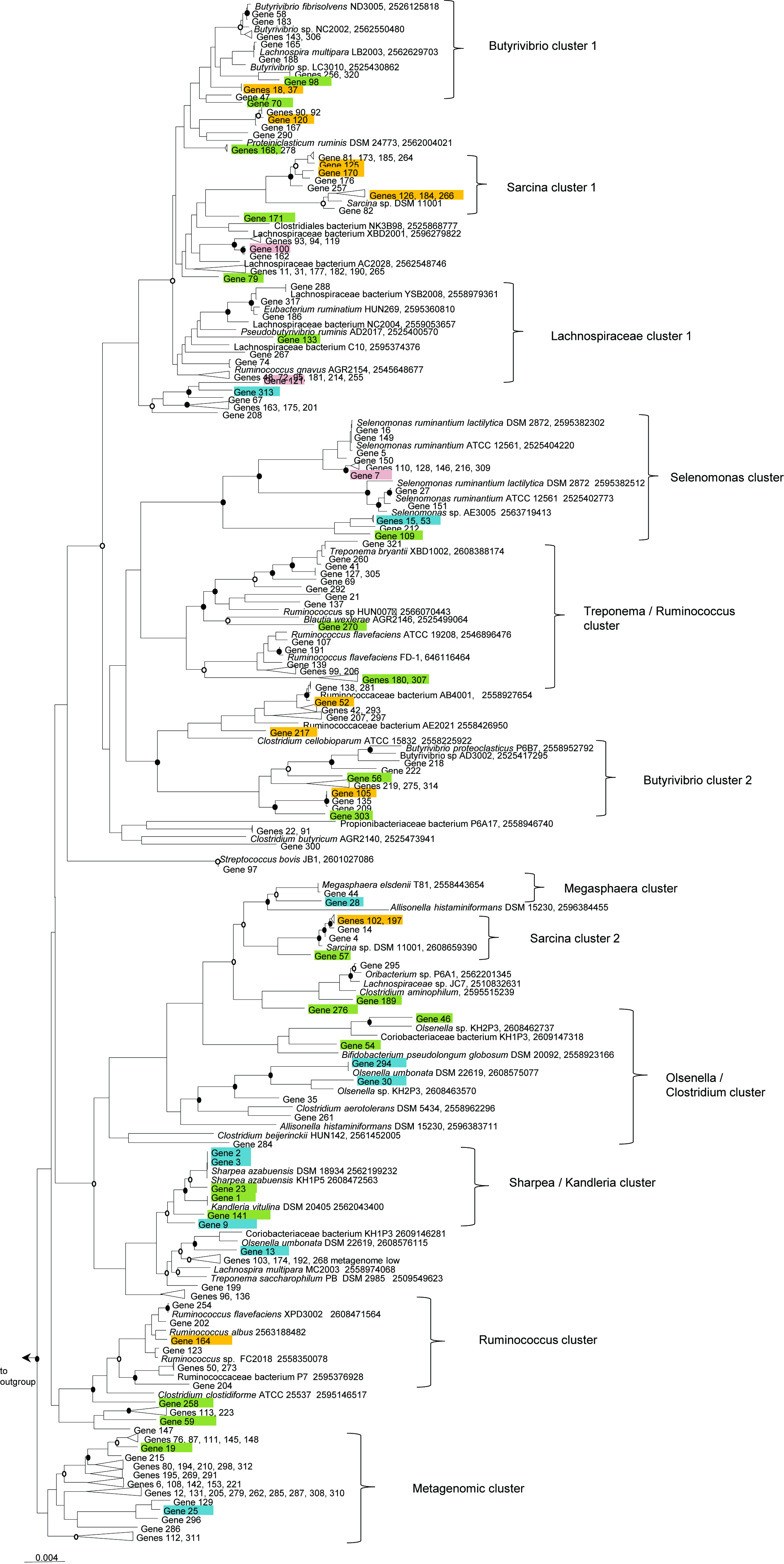

Supplement: Additional file 11: Figure S5. — Phylogenetic maximum likelihood tree of lactate dehydrogenase genes based on a multiple sequence alignment including sequences >310aa from the metagenomic reassembly and reference sequences from selected rumen microorganisms. Bootstrap support of ≥75 % is illustrated by open and ≥90 % by filled circles. Colour shadings mark genes with significantly (P ≤ 0.05) more abundance in low metagenome (green), low metagenome and metatranscriptome (blue), high metagenome (orange) and high metagenome and metatranscriptome (red) samples. Reference sequences of lactate dehydrogenase genes from microbial isolates are shown with the relevant strain name and the 10-digit gene identifier in the IMG database. The out-group consisted of 14 lactate dehydrogenase amino acid sequences from protists. The scale bar represents 0.4 % sequence divergence. (TIF 1161 kb) [file 40168_2016_201_MOESM11_ESM.tif]

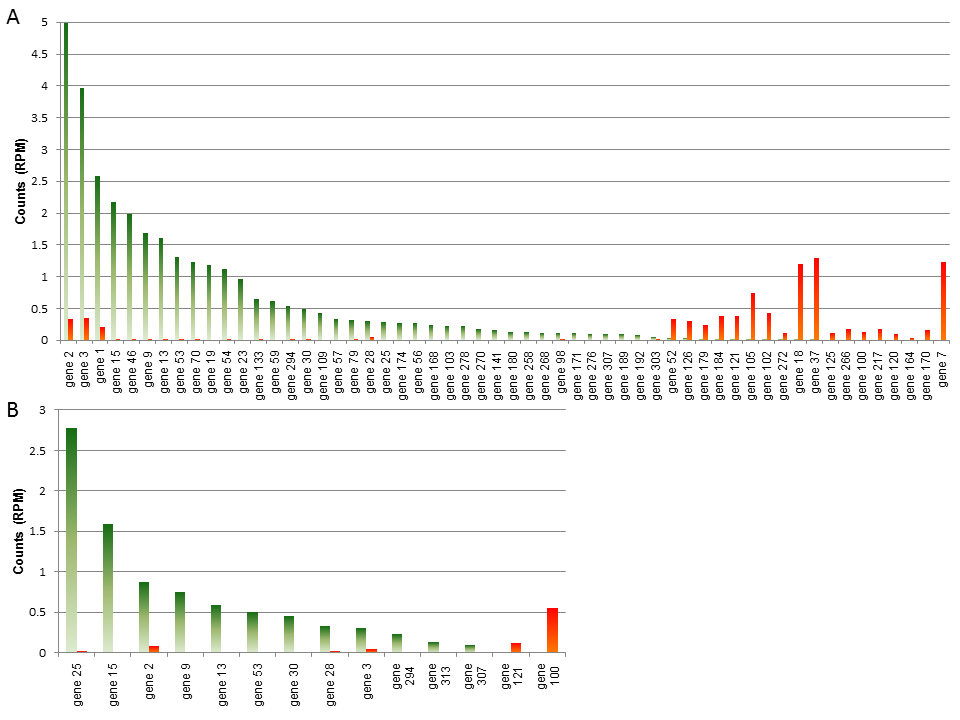

Supplement: Additional file 12: Figure S6. — Barplot showing metagenome (A) and metatranscriptome (B) read count numbers based on read mappings against reassembled rumen metagenome lactate dehydrogenase genes (K00016). Shown are genes with significantly different read count abundance (P ≤ 0.05) between low (green) and high (red) methane samples based on Wilcoxon rank sum tests and Benjamini-Hochberg correction. Metatranscriptome data for gene 7 was excluded due to unusually high expression values in high methane yield sheep (see the “Results” section). (TIF 72 kb) [file 40168_2016_201_MOESM12_ESM.tif]

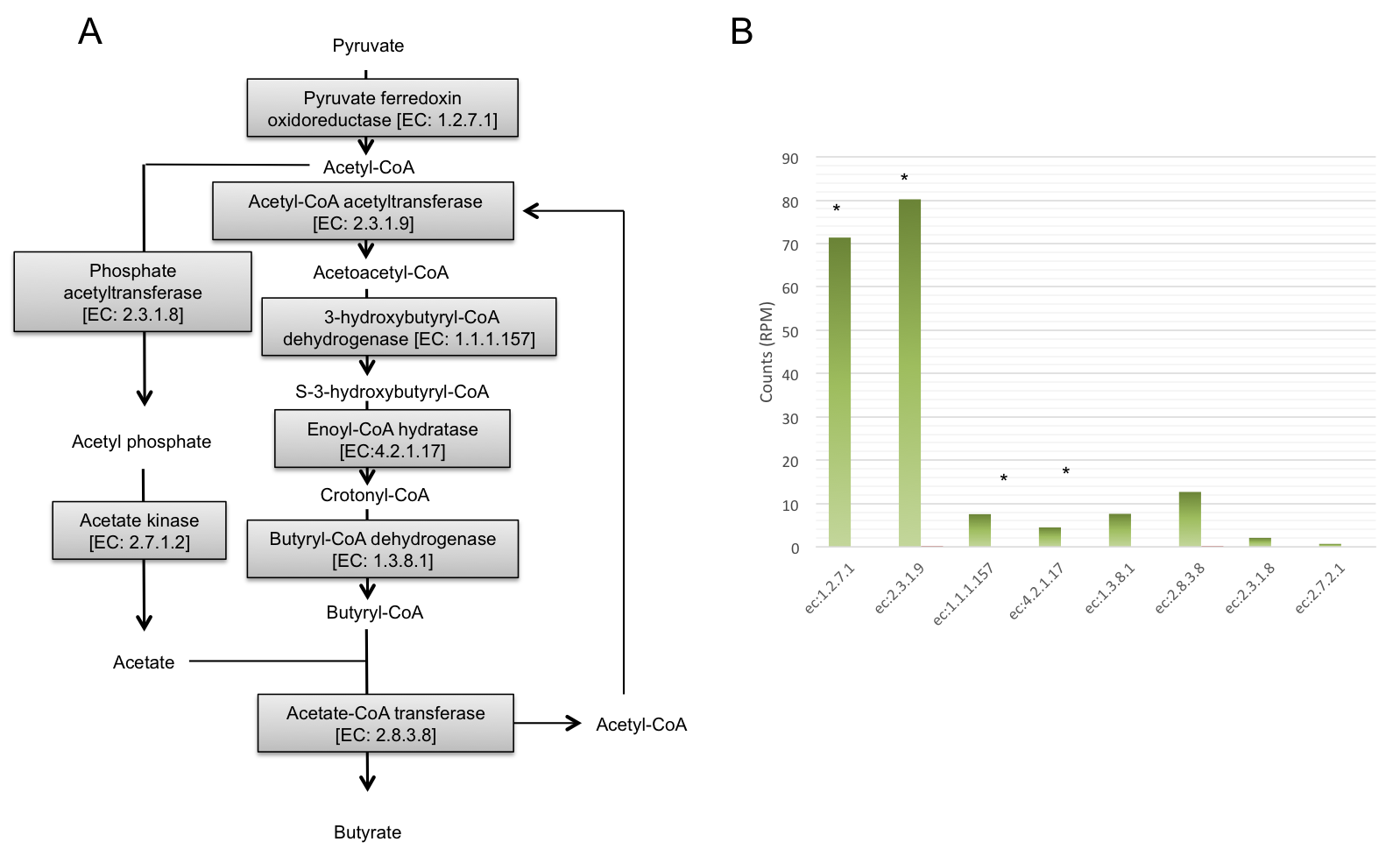

Supplement: Additional file 16: Figure S8. — Overview of pyruvate degradation functions correlated with methane yield in Megasphaera elsdenii J1 (A). Bar charts show mean read counts (normalised to RPM) in high (orange) and low (green) metatranscriptome (transcripts) data. *P < 0.05 based on WRS (B). (TIF 191 kb) [file 40168_2016_201_MOESM16_ESM.tif]
