# Supplementary material for: Rumen metagenome and metatranscriptome analyses of low methane yield sheep reveals a Sharpea-enriched microbiome characterised by lactic acid formation and utilisation
Source: Microbiome. 2016 Oct 19;4:56. doi: 10.1186/s40168-016-0201-2 (PMC5069950; doi:10.1186/s40168-016-0201-2)
Supplement: Additional file 3: Table S2. — KEGG genes from metagenome (DNA) and metatranscriptome (RNA) data commonly identified in sparse partial least squares analysis (sPLS) and Wilcoxon rank sum test (WRS) correlated to methane yield/significantly differentially abundant between high and low methane yield sheep. (PDF 507 kb) [file 40168_2016_201_MOESM3_ESM.pdf]

Table S2. KEGG genes from metagenome (DNA) and metatranscriptome (RNA) data commonly identified in sparse partial least squares analysis (sPLS) and Wilcoxon Rank Sum test (WRS) correlated to methane yield / significantly differentially abundant between high and low methane yield sheep.

**Data type**	**High/Low methane related**	**Mean low**	**Mean high**	**sPLS**	**WRS**		**KEGG gene**	**Definition**	**Pathways**
				**correlation coefficient**	**p.adj.BH**	**fold change**			
DNA	High	2.71	4.55	0.0159	0.0472	1.68	K00311	electron-transferring-flavoprotein dehydrogenase [EC:1.5.5.1]	-
DNA	High	0.08	0.28	0.0146	0.0145	3.59	K00539	oxidoreducatase E1.97.1.-	DDT degradation-Chlorocyclohexane and chlorobenzene degradation-Bisphenol degradation-Chloroalkane and chloroalkene degradation
DNA	High	170.53	236.29	0.0167	0.0086	1.39	K00666	fatty-acyl-CoA synthase [EC:6.2.1.-]	-
DNA	High	5.75	9.02	0.0128	0.0086	1.57	K00758	thymidine phosphorylase [EC:2.4.2.4]	Pyrimidine metabolism-Drug metabolism - other enzymes-Bladder cancer
DNA	High	0.02	0.05	0.0144	0.0145	2.24	K00827	alanine-glyoxylate transaminase / (R)-3-amino-2-methylpropionate-pyruvate transaminase [EC:2.6.1.44 2.6.1.40]	Alanine, aspartate and glutamate metabolism-Glycine, serine and threonine metabolism
DNA	High	0.05	0.14	0.0127	0.0145	2.47	K00953	FAD synthetase [EC:2.7.7.2]	Riboflavin metabolism
DNA	High	0.17	0.53	0.0129	0.019	3.21	K01160	crossover junction endodeoxyribonuclease RusA [EC:3.1.22.4]	-
DNA	High	1.84	3.87	0.0176	0.0086	2.11	K01342	subtilisin [EC:3.4.21.62]	-
DNA	High	0.49	0.78	0.0142	0.0086	1.6	K01631	2-dehydro-3-deoxyphosphogalactonate aldolase [EC:4.1.2.21]	Galactose metabolism
DNA	High	0.99	3.33	0.0147	0.0086	3.36	K01792	glucose-6-phosphate 1-epimerase [EC:5.1.3.15]	Glycolysis / Gluconeogenesis
DNA	High	4.26	13.27	0.0137	0.0086	3.12	K01846	methylaspartate mutase [EC:5.4.99.1]	C5-Branched dibasic acid metabolism
DNA	High	392.89	461.35	0.0147	0.0408	1.17	K01912	phenylacetate-CoA ligase [EC:6.2.1.30]	Phenylalanine metabolism
DNA	High	12.86	19.32	0.0186	0.0086	1.5	K01913	trans-2-methyl-5-isopropylhexa-2,5-dienoate-CoA ligase	Aminobenzoate degradation-Limonene and pinene degradation-Caprolactam degradation-Tropane, piperidine and pyridine alkaloid biosynthesis
DNA	High	0.79	1.87	0.0142	0.0472	2.36	K02674	type IV pilus assembly protein PilY1	-
DNA	High	19.75	37.58	0.0129	0.0408	1.9	K02856	L-rhamnose-H+ transport protein	-
DNA	High	1.22	3.71	0.0159	0.0233	3.03	K03219	type III secretion protein SctC	Bacterial secretion system
DNA	High	0.80	2.52	0.0164	0.0233	3.13	K03222	type III secretion protein SctJ	Bacterial secretion system
DNA	High	0.46	1.31	0.0153	0.0086	2.83	K03223	type III secretion protein SctL	Bacterial secretion system
DNA	High	2.47	8.32	0.0153	0.0086	3.37	K03224	ATP synthase in type III secretion protein SctN [EC:3.6.3.14]	Bacterial secretion system
DNA	High	1.23	3.78	0.0141	0.0086	3.06	K03226	type III secretion protein SctR	Bacterial secretion system
DNA	High	0.46	1.54	0.016	0.0086	3.33	K03227	type III secretion protein SctS	Bacterial secretion system
DNA	High	1.11	3.57	0.0149	0.0086	3.21	K03228	type III secretion protein SctT	Bacterial secretion system
DNA	High	1.60	5.05	0.0151	0.0086	3.16	K03229	type III secretion protein SctU	Bacterial secretion system
DNA	High	3.68	12.22	0.0152	0.0086	3.32	K03230	type III secretion protein SctV	Bacterial secretion system
DNA	High	32.74	51.71	0.0149	0.0472	1.58	K03579	ATP-dependent helicase HrpB [EC:3.6.4.13]	-
DNA	High	0.17	0.4	0.0162	0.0484	2.33	K04058	type III secretion protein SctW	Bacterial secretion system
DNA	High	0.76	1.4	0.0187	0.0086	1.85	K04874	potassium voltage-gated channel Shaker-related subfamily A member 1	-
DNA	High	0.06	0.13	0.0134	0.0086	2	K05830	acetylornithine/LysW-gamma-L-lysine aminotransferase [EC:2.6.1.11 2.6.1.-]	Lysine biosynthesis-Arginine and proline metabolism-2-Oxocarboxylic acid metabolism-Biosynthesis of amino acids

DNA	High	0.06	0.13	0.0168	0.0086	2.32	K07249	retinal dehydrogenase [EC:1.2.1.36]	Retinol metabolism
DNA	High	0.01	0.05	0.0119	0.0086	5.82	K07283	putative salt-induced outer membrane protein	-
DNA	High	1.01	3.32	0.0154	0.0086	3.3	K07796	Cu(I)/Ag(I) efflux system outer membrane protein CusC/SilC	Two-component system
DNA	High	0.00	0.03	0.0157	0.0453	6.92	K08605	coccolysin [EC:3.4.24.30]	-
DNA	High	0.17	0.42	0.0165	0.0086	2.5	K08635	membrane metallo-endopeptidase-like 1	-
DNA	High	0.03	0.14	0.014	0.019	4.4	K08636	phosphate-regulating neutral endopeptidase [EC:3.4.24.-]	-
DNA	High	130.14	167.09	0.0143	0.0384	1.28	K08641	D-alanyl-D-alanine dipeptidase [EC:3.4.13.22]	-
DNA	High	0.07	0.35	0.0149	0.0305	5.01	K09610	endothelin-converting enzyme-like 1 [EC:3.4.24.-]	-
DNA	High	0.01	0.07	0.0159	0.0145	8.6	K09711	hypothetical protein	-
DNA	High	0.36	0.7	0.0109	0.0472	1.94	K09716	hypothetical protein	-
DNA	High	0.02	0.09	0.0119	0.019	4.25	K09744	hypothetical protein	-
DNA	High	0.11	0.27	0.0177	0.019	2.43	K10060	C-type lectin domain family 4 member F	-
DNA	High	1.40	2.18	0.0172	0.0086	1.56	K10243	-	-
DNA	High	0.03	0.08	0.0176	0.0086	3.13	K10639	E3 ubiquitin-protein ligase CCNP1IP1 [EC:6.3.2.19]	-
DNA	High	0.10	0.29	0.0145	0.0145	2.86	K11382	MFS transporter, OPA family, phosphoglycerate transporter protein	Two-component system
DNA	High	0.53	1.53	0.0152	0.019	2.87	K11900	type VI secretion system protein ImpC	-
DNA	High	0.02	0.06	0.0125	0.0086	2.81	K12204	defect in organelle trafficking protein DotC	-
DNA	High	0.11	0.23	0.0135	0.019	2.14	K12206	intracellular multiplication protein IcmB	-
DNA	High	0.00	0.01	0.0173	0.0086	21.48	K12221	intracellular multiplication protein IcmS	-
DNA	High	0.04	0.11	0.0113	0.0086	2.66	K12434	polyketide synthase 7	-
DNA	High	0.01	0.05	0.0148	0.019	4.43	K12448	UDP-arabinose 4-epimerase [EC:5.1.3.5]	Amino sugar and nucleotide sugar metabolism
DNA	High	0.08	0.23	0.0151	0.0268	3.09	K13085	phosphatidylinositol-4,5-bisphosphate 4-phosphatase [EC:3.1.3.78]	Bacterial invasion of epithelial cells-Shigellosis-Salmonella infection
DNA	High	0.23	0.86	0.0161	0.0086	3.7	K13600	chlorophyllide a oxygenase [EC:1.14.13.122]	Porphyrin and chlorophyll metabolism
DNA	High	0.71	1.76	0.0168	0.0086	2.47	K13670	putative glycosyltransferase [EC:2.4.-.-]	-
DNA	High	2.00	5.82	0.014	0.0453	2.9	K13893	microcin C transport system substrate-binding protein	ABC transporters
DNA	High	0.13	0.39	0.0155	0.0086	2.91	K14275	D-xylonate dehydratase	Pentose and glucuronate interconversions
DNA	High	0.01	0.07	0.0184	0.019	6.63	K14324	histone deacetylase complex subunit SAP18	RNA transport-mRNA surveillance pathway
DNA	High	0.75	2.81	0.0171	0.0086	3.73	K14333	2,3-dihydroxybenzoate decarboxylase [EC:4.1.1.46]	Benzoate degradation-Aminobenzoate degradation
DNA	High	1.70	3.94	0.0174	0.0086	2.31	K14414	transcriptional regulatory protein RtcR	-
DNA	High	0.03	0.09	0.0146	0.0086	3.13	K14426	solute carrier family 12 (sodium/chloride transporter), member 3	-
DNA	High	0.16	0.34	0.018	0.033	2.11	K14429	solute carrier family 12 (potassium/chloride transporters), member 9	-
DNA	High	0.08	0.23	0.0152	0.0086	2.95	K12217	intracellular multiplication protein IcmO	-
DNA	Low	50.87	15.04	-0.0097	0.0086	3.38	K00005	glycerol dehydrogenase [EC:1.1.1.6]	Glycerolipid metabolism

DNA	Low	92.78	62.57	-0.0069	0.0359	1.48	K00016	L-lactate dehydrogenase [EC:1.1.1.27]	Glycolysis / Gluconeogenesis-Cysteine and methionine metabolism-Pyruvate metabolism-Propanoate metabolism
DNA	Low	0.28	0.08	-0.0124	0.0145	3.45	K00119		Phosphonate and phosphinate metabolism
DNA	Low	108.76	74.54	-0.0073	0.0484	1.46	K00128	aldehyde dehydrogenase (NAD+) [EC:1.2.1.3]	Glycolysis / Gluconeogenesis-Pentose and glucuronate interconversions-Ascorbate and aldarate metabolism-Fatty acid degradation-Valine, leucine and isoleucine degradation-Lysine degradation-Arginine and proline metabolism-Histidine metabolism-Tryptophan metabolism-beta-Alanine metabolism-Glycerolipid metabolism-Pyruvate metabolism-Chloroalkane and chloroalkene degradation-Propanoate metabolism-Limonene and pinene degradation
DNA	Low	8.97	5.65	-0.0065	0.0233	1.59	K00224		DDT degradation-Chlorocyclohexane and chlorobenzene degradation-Polycyclic aromatic hydrocarbon degradation
DNA	Low	3.41	2.32	-0.0071	0.0408	1.47	K00251	3-oxo-5-beta-steroid 4-dehydrogenase [EC:1.3.1.3]	Primary bile acid biosynthesis-Steroid hormone biosynthesis
DNA	Low	7.65	0.48	-0.0097	0.0086	15.93	K00366	ferredoxin-nitrite reductase [EC:1.7.7.1]	Nitrogen metabolism
DNA	Low	29.20	10.38	-0.0058	0.0145	2.81	K00383	glutathione reductase (NADPH) [EC:1.8.1.7]	Glutathione metabolism-Thyroid hormone synthesis
DNA	Low	29.87	11.99	-0.008	0.0086	2.49	K00394	adenylylsulfate reductase, subunit A [EC:1.8.99.2]	Sulfur metabolism
DNA	Low	30.05	14.8	-0.0062	0.0268	2.03	K00459	nitronate monooxygenase [EC:1.13.12.16]	Nitrogen metabolism
DNA	Low	2.89	1.27	-0.0072	0.033	2.27	K00462	biphenyl-2,3-diol 1,2-dioxygenase [EC:1.13.11.39]	Chlorocyclohexane and chlorobenzene degradation-Dioxin degradation-Degradation of aromatic compounds
DNA	Low	14.36	1.29	-0.0082	0.0086	11.16	K00526	ribonucleoside-diphosphate reductase beta chain [EC:1.17.4.1]	Purine metabolism-Pyrimidine metabolism
DNA	Low	37.82	17.78	-0.0065	0.0384	2.13	K00547	homocysteine S-methyltransferase [EC:2.1.1.10]	Cysteine and methionine metabolism
DNA	Low	76.62	42.74	-0.008	0.0086	1.79	K00549	5-methyltetrahydropteroyltriglutamate--homocysteine methyltransferase [EC:2.1.1.14]	Cysteine and methionine metabolism-Selenocompound metabolism-Biosynthesis of amino acids
DNA	Low	21.25	11.85	-0.0066	0.0305	1.79	K00563	23S rRNA (guanine745-N1)-methyltransferase [EC:2.1.1.187]	-
DNA	Low	2.42	1.24	-0.0049	0.0484	1.95	K00614		-
DNA	Low	5.09	1.38	-0.0068	0.0086	3.68	K00687	penicillin-binding protein 2B [EC:2.3.2.-]	beta-Lactam resistance-Peptidoglycan biosynthesis
DNA	Low	14.23	3.45	-0.0074	0.0086	4.13	K00904	deoxyguanosine kinase [EC:2.7.1.113]	Purine metabolism
DNA	Low	31.05	12.96	-0.007	0.0086	2.4	K00917	tagatose 6-phosphate kinase [EC:2.7.1.144]	Galactose metabolism
DNA	Low	26.73	4.25	-0.008	0.0086	6.29	K01220	6-phospho-beta-galactosidase [EC:3.2.1.85]	Galactose metabolism
DNA	Low	24.12	1.64	-0.0085	0.0086	14.73	K01232	maltose-6'-phosphate glucosidase [EC:3.2.1.122]	Starch and sucrose metabolism
DNA	Low	3.36	1.48	-0.0092	0.0086	2.26	K01249	DNA glycosylase [EC:3.2.2.-]	-
DNA	Low	87.03	66.91	-0.0044	0.0453	1.3	K01271	Xaa-Pro dipeptidase [EC:3.4.13.9]	-
DNA	Low	28.74	14.08	-0.0101	0.0086	2.04	K01420	CRP/FNR family transcriptional regulator, anaerobic regulatory protein	-
DNA	Low	9.07	2.03	-0.0048	0.0086	4.46	K01444	N4-(beta-N-acetylglucosaminyl)-L-asparaginase [EC:3.5.1.26]	Other glycan degradation-Lysosome
DNA	Low	5.64	1.82	-0.0142	0.0086	3.1	K01455	formamidase [EC:3.5.1.49]	Glyoxylate and dicarboxylate metabolism-Nitrogen metabolism-Carbon metabolism
DNA	Low	19.51	8.74	-0.0086	0.0086	2.23	K01632	fructose-6-phosphate phosphoketolase [EC:4.1.2.22]	Carbon fixation in photosynthetic organisms
DNA	Low	48.66	29.28	-0.0128	0.0408	1.66	K01750	ornithine cyclodeaminase [EC:4.3.1.12]	Arginine and proline metabolism-Biosynthesis of amino acids

DNA	Low	21.74	11.83	-0.0088	0.0408	1.84	K01788	N-acylglucosamine-6-phosphate 2-epimerase [EC:5.1.3.9]	Amino sugar and nucleotide sugar metabolism
DNA	Low	73.43	50.49	-0.008	0.0425	1.45	K02036	phosphate transport system ATP-binding protein [EC:3.6.3.27]	ABC transporters
DNA	Low	67.44	47.15	-0.0074	0.0425	1.43	K02038	phosphate transport system permease protein	ABC transporters
DNA	Low	12.67	6.75	-0.0054	0.0359	1.88	K02047	sulfate transport system permease protein	Sulfur metabolism-ABC transporters
DNA	Low	22.51	6.95	-0.0094	0.0145	3.24	K02082	tagatose-6-phosphate ketose/aldose isomerase [EC:5.-.-.-]	-
DNA	Low	24.83	12.88	-0.0123	0.0086	1.93	K02217	ferritin [EC:1.16.3.1]	Porphyrin and chlorophyll metabolism
DNA	Low	6.56	1.15	-0.0058	0.0086	5.71	K02243	competence protein ComGA	-
DNA	Low	3.44	0.55	-0.0069	0.0086	6.27	K02245	competence protein ComGC	-
DNA	Low	2.52	0.96	-0.0055	0.0384	2.62	K02430	DeoR family transcriptional regulator, L-fucose operon activator	-
DNA	Low	2.33	0.64	-0.0071	0.0086	3.64	K02436	DeoR family transcriptional regulator, galactitol utilization operon repressor	-
DNA	Low	0.30	0.05	-0.0152	0.0233	5.82	K02511	MFS transporter, ACS family, 4-hydroxyphenylacetate permease	-
DNA	Low	6.73	2.31	-0.0117	0.0268	2.91	K02526	2-keto-3-deoxygluconate permease	-
DNA	Low	3.43	1.53	-0.0081	0.0086	2.24	K02530	DeoR family transcriptional regulator, lactose phosphotransferase system repressor	-
DNA	Low	18.90	7.81	-0.0067	0.0086	2.42	K02598	nitrite transporter NirC	-
DNA	Low	18.69	7.54	-0.0046	0.0359	2.48	K02744	PTS system, N-acetylgalactosamine-specific IIA component [EC:2.7.1.69]	Galactose metabolism-Phosphotransferase system (PTS)
DNA	Low	8.49	0.59	-0.0072	0.0086	14.44	K02745	PTS system, N-acetylgalactosamine-specific IIB component [EC:2.7.1.69]	Galactose metabolism-Phosphotransferase system (PTS)
DNA	Low	10.24	0.77	-0.0079	0.0086	13.32	K02746	PTS system, N-acetylgalactosamine-specific IIC component	Galactose metabolism-Phosphotransferase system (PTS)
DNA	Low	12.13	0.85	-0.0079	0.0086	14.33	K02747	PTS system, N-acetylgalactosamine-specific IID component	Galactose metabolism-Phosphotransferase system (PTS)
DNA	Low	14.61	2.96	-0.0102	0.0086	4.94	K02759	PTS system, cellobiose-specific IIA component [EC:2.7.1.69]	Phosphotransferase system (PTS)
DNA	Low	34.88	6.18	-0.0074	0.0086	5.64	K02760	PTS system, cellobiose-specific IIB component [EC:2.7.1.69]	Phosphotransferase system (PTS)
DNA	Low	97.79	27.78	-0.008	0.0145	3.52	K02761	PTS system, cellobiose-specific IIC component	Phosphotransferase system (PTS)
DNA	Low	7.48	1.5	-0.0088	0.033	4.98	K02769	PTS system, fructose-specific IIB component [EC:2.7.1.69]	Fructose and mannose metabolism-Phosphotransferase system (PTS)
DNA	Low	13.39	1.44	-0.0072	0.0145	9.31	K02770	PTS system, fructose-specific IIC component	Fructose and mannose metabolism-Phosphotransferase system (PTS)
DNA	Low	28.9	7.75	-0.0064	0.0086	3.73	K02777	PTS system, glucose-specific IIA component [EC:2.7.1.69]	Glycolysis / Gluconeogenesis-Starch and sucrose metabolism-Amino sugar and nucleotide sugar metabolism-Phosphotransferase system (PTS)
DNA	Low	19.69	2.96	-0.0067	0.0086	6.66	K02778	PTS system, glucose-specific IIB component [EC:2.7.1.69]	Glycolysis / Gluconeogenesis-Amino sugar and nucleotide sugar metabolism-Phosphotransferase system (PTS)
DNA	Low	5.64	0.92	-0.0076	0.0086	6.14	K02786	PTS system, lactose-specific IIA component [EC:2.7.1.69]	Galactose metabolism-Phosphotransferase system (PTS)
DNA	Low	29.33	4.7	-0.008	0.0086	6.24	K02787	PTS system, lactose-specific IIB component [EC:2.7.1.69]	Galactose metabolism-Phosphotransferase system (PTS)

DNA	Low	0.16	0.04	-0.0116	0.0408	3.85	K02815	PTS system, sorbose-specific IID component	Fructose and mannose metabolism-Phosphotransferase system (PTS)
DNA	Low	2.72	0.95	-0.0098	0.0268	2.87	K03189	urease accessory protein	-
DNA	Low	37.88	15.12	-0.0089	0.0086	2.5	K03311	branched-chain amino acid:cation transporter, LIVCS family	-
DNA	Low	29.81	10.27	-0.0099	0.019	2.9	K03325	arsenite transporter, ACR3 family	-
DNA	Low	0.45	0.15	-0.0117	0.0268	2.99	K03445	MFS transporter, DHA1 family, purine ribonucleoside efflux pump	-
DNA	Low	15.24	5.91	-0.0052	0.0359	2.58	K03478	hypothetical protein	-
DNA	Low	11.35	2.32	-0.0088	0.0086	4.89	K03481	RpiR family transcriptional regulator, glv operon transcriptional regulator	-
DNA	Low	21.25	8.36	-0.0108	0.0384	2.54	K03488	beta-glucoside operon transcriptional antiterminator	-
DNA	Low	12.54	2.11	-0.009	0.0086	5.95	K03492	GntR family transcriptional regulator	-
DNA	Low	2.10	0.51	-0.0071	0.0145	4.14	K03647	protein involved in ribonucleotide reduction	-
DNA	Low	33.98	21.44	-0.004	0.0425	1.58	K03712	MarR family transcriptional regulator, multiple antibiotic resistance protein MarR	-
DNA	Low	9.03	3.76	-0.0082	0.019	2.4	K03741	arsenate reductase [EC:1.20.4.1]	-
DNA	Low	2.83	0.4	-0.0078	0.0086	7.12	K04047	starvation-inducible DNA-binding protein	-
DNA	Low	5.57	1.94	-0.0051	0.033	2.86	K05299	formate dehydrogenase alpha subunit [EC:1.2.1.43]	Methane metabolism-Carbon fixation pathways in prokaryotes-Carbon metabolism
DNA	Low	10.86	6.26	-0.0071	0.0086	1.73	K05337	ferredoxin	-
DNA	Low	2.51	0.64	-0.0064	0.0086	3.93	K05362	UDP-N-acetylmuramoyl-L-alanyl-D-glutamate-L-lysine ligase [EC:6.3.2.7]	Peptidoglycan biosynthesis
DNA	Low	0.43	0.08	-0.0071	0.0145	5.24	K05522	endonuclease VIII [EC:3.2.2.- 4.2.99.18]	Base excision repair
DNA	Low	0.93	0.16	-0.0063	0.0086	5.83	K05556	ketoreductase RED1 [EC:1.1.1.-]	Biosynthesis of type II polyketide products
DNA	Low	16.72	5.66	-0.0046	0.0145	2.95	K05820	MFS transporter, PPP family, 3-phenylpropionic acid transporter	-
DNA	Low	3.57	1.74	-0.0072	0.0268	2.05	K05823	N-acetyldiaminopimelate deacetylase [EC:3.5.1.47]	Lysine biosynthesis-Biosynthesis of amino acids
DNA	Low	28.56	6.39	-0.0077	0.0145	4.47	K05845	osmoprotectant transport system substrate-binding protein	ABC transporters
DNA	Low	17.58	6.83	-0.0061	0.0233	2.57	K05847	osmoprotectant transport system ATP-binding protein	ABC transporters
DNA	Low	1.29	0.38	-0.0085	0.0086	3.43	K05894	12-oxophytodienoic acid reductase [EC:1.3.1.42]	alpha-Linolenic acid metabolism
DNA	Low	4.19	0.34	-0.0092	0.0086	12.2	K05915		Bisphenol degradation-Naphthalene degradation
DNA	Low	20.29	12.59	-0.0102	0.033	1.61	K06199	CrcB protein	-
DNA	Low	5.89	2.77	-0.0081	0.0086	2.13	K06286	septation ring formation regulator	-
DNA	Low	2.76	0.32	-0.0053	0.0086	8.61	K06929		-
DNA	Low	28.73	13.99	-0.0058	0.0268	2.05	K06956		-
DNA	Low	13.42	2.14	-0.0083	0.0086	6.27	K07396	putative protein-disulfide isomerase	-
DNA	Low	21.92	13.76	-0.0097	0.0408	1.59	K07483	transposase	-
DNA	Low	629.65	328.36	-0.006	0.0359	1.92	K07496	putative transposase	-
DNA	Low	463.63	367.06	-0.0179	0.0086	1.26	K07497	putative transposase	-
DNA	Low	0.54	0.19	-0.0069	0.0425	2.82	K07542	phosphatidylinositol glycan, class V [EC:2.4.1.-]	Glycosylphosphatidylinositol(GPI)-anchor biosynthesis

DNA	Low	6.81	3.46	-0.0062	0.033	1.97	K07570	general stress protein 13	-
DNA	Low	1.26	0.2	-0.006	0.0086	6.19	K07757	sugar-phosphatase [EC:3.1.3.23]	-
DNA	Low	20.81	9.01	-0.0066	0.0425	2.31	K07770	two-component system, OmpR family, response regulator CssR	Two-component system
DNA	Low	5.09	1.55	-0.0068	0.0268	3.3	K08153	MFS transporter, DHA1 family, multidrug resistance protein	-
DNA	Low	5.31	0.99	-0.0099	0.0086	5.35	K08159	MFS transporter, DHA1 family, L-arabinose/isopropyl-beta-D-thiogalactopyranoside export protein	-
DNA	Low	28.35	7.8	-0.0085	0.0086	3.63	K09155	hypothetical protein	-
DNA	Low	12.71	3.24	-0.0063	0.0086	3.93	K09693	teichoic acid transport system ATP-binding protein [EC:3.6.3.40]	ABC transporters
DNA	Low	1.32	0.09	-0.007	0.0086	14.05	K09813	hemin transport system permease protein	ABC transporters
DNA	Low	24.58	9.3	-0.012	0.0086	2.64	K09825	Fur family transcriptional regulator, peroxide stress response regulator	-
DNA	Low	36.91	11.22	-0.009	0.0233	3.29	K09963	hypothetical protein	-
DNA	Low	7.87	3.19	-0.0079	0.0086	2.46	K09972	general L-amino acid transport system ATP-binding protein [EC:3.6.3.-]	ABC transporters
DNA	Low	0.51	0.13	-0.0068	0.0086	3.83	K09996	arginine transport system substrate-binding protein	ABC transporters
DNA	Low	14.88	2.33	-0.0072	0.0086	6.38	K10120	fructooligosaccharide transport system substrate-binding protein	-
DNA	Low	12.30	2.84	-0.0071	0.0086	4.33	K10121	fructooligosaccharide transport system permease protein	-
DNA	Low	10.11	1.07	-0.0069	0.0086	9.44	K10122	fructooligosaccharide transport system permease protein	-
DNA	Low	24.45	2.16	-0.0066	0.0086	11.31	K10254	myosin-crossreactive antigen	-
DNA	Low	0.47	0.1	-0.0088	0.019	4.57	K10815	hydrogen cyanide synthase HcnB [EC:1.4.99.5]	Cyanoamino acid metabolism
DNA	Low	1.11	0.26	-0.0097	0.0086	4.25	K11202	PTS system, fructose-specific IIB-like component [EC:2.7.1.69]	-
DNA	Low	6.90	3.39	-0.0059	0.0086	2.04	K11630	two-component system, OmpR family, bacitracin resistance response regulator BceR	Two-component system
DNA	Low	1.02	0.11	-0.0079	0.019	9.15	K11633	two-component system, OmpR family, sensor histidine kinase YxdK [EC:2.7.13.3]	Two-component system
DNA	Low	1.20	0.6	-0.0091	0.0359	2	K11892	type VI secretion system protein ImpK	Bacterial secretion system
DNA	Low	6.23	1.6	-0.0084	0.0268	3.89	K12268	accessory secretory protein Asp1	-
DNA	Low	7.55	2.21	-0.0085	0.0268	3.41	K12269	accessory secretory protein Asp2	-
DNA	Low	1.48	0.48	-0.0079	0.0268	3.05	K12270	accessory secretory protein Asp3	-
DNA	Low	0.05	0.01	-0.0088	0.0086	7.06	K12301	MFS transporter, ACS family, solute carrier family 17 (sodium-dependent inorganic phosphate cotransporter), member 5	Lysosome
DNA	Low	3.48	0.46	-0.0061	0.0086	7.55	K12556	penicillin-binding protein 2X [EC:2.3.2.-]	beta-Lactam resistance-Peptidoglycan biosynthesis
DNA	Low	7.13	1.5	-0.0135	0.0484	4.75	K13021	MFS transporter, ACS family, tartrate transporter	-
DNA	Low	2.04	0.88	-0.0063	0.0384	2.33	K13403	methylenetetrahydrofolate dehydrogenase(NAD+) / 5,10-	One carbon pool by folate

								methenyltetrahydrofolate cyclohydrolase [EC:1.5.1.15 3.5.4.9]	
DNA	Low	0.63	0.15	-0.0068	0.0086	4.12	K13639	MerR family transcriptional regulator, redox-sensitive transcriptional activator SoxR	-
DNA	Low	5.93	1.53	-0.0112	0.0233	3.87	K13727	phenolic acid decarboxylase [EC:4.1.1.-]	-
DNA	Low	0.19	0.01	-0.0061	0.0145	18.94	K13732	fibronectin-binding protein A	Bacterial invasion of epithelial cells
DNA	Low	13.99	9.9	-0.0077	0.0233	1.41	K13770	TetR/AcrR family transcriptional regulator, fatty acid metabolism regulator protein	-
DNA	Low	330.85	281.8	-0.0107	0.033	1.17	K14155	cystathione beta-lyase [EC:4.4.1.8]	Cysteine and methionine metabolism-Selenocompound metabolism-Biosynthesis of amino acids
DNA	Low	0.15	0.06	-0.0093	0.0453	2.74	K14286	ethanolamine-phosphate phospho-lyase [EC:4.2.3.2]	Glycerophospholipid metabolism
DNA	Low	0.07	0.01	-0.0101	0.033	8.72	K14309	nuclear pore complex protein Nup93	RNA transport
DNA	Low	2.20	0.57	-0.0057	0.0086	3.84	K15016	enoyl-CoA hydratase / 3-hydroxyacyl-CoA dehydrogenase [EC:4.2.1.17 1.1.1.35]	Carbon fixation pathways in prokaryotes-Carbon metabolism
DNA	Low	0.34	0.06	-0.0045	0.0086	5.88	K15054	(S)-mandelate dehydrogenase [EC:1.1.99.31]	Aminobenzoate degradation
RNA	High	0.02	0.14	0.0259	0.0212	5.82	K00323	NAD(P) transhydrogenase [EC:1.6.1.2]	Nicotinate and nicotinamide metabolism
RNA	High	45.15	121.23	0.0122	0.0067	2.68	K00399	methyl-coenzyme M reductase alpha subunit [EC:2.8.4.1]	Methane metabolism-Carbon metabolism
RNA	High	29.13	89.94	0.0134	0.0067	3.09	K00401	methyl-coenzyme M reductase beta subunit [EC:2.8.4.1]	Methane metabolism-Carbon metabolism
RNA	High	2.15	4.77	0.0287	0.0252	2.21	K00814	alanine transaminase [EC:2.6.1.2]	Alanine, aspartate and glutamate metabolism-Carbon fixation in photosynthetic organisms-Carbon metabolism-2-Oxocarboxylic acid metabolism-Biosynthesis of amino acids
RNA	High	0.12	0.52	0.0291	0.0067	4.38	K01479	formiminoglutamase [EC:3.5.3.8]	Histidine metabolism
RNA	High	0.32	1.15	0.022	0.0067	3.56	K02319	DNA polymerase I [EC:2.7.7.7]	Purine metabolism-Pyrimidine metabolism
RNA	High	0.16	0.49	0.0226	0.0067	3.07	K02683	DNA primase [EC:2.7.7.-]	-
RNA	High	0.70	3.21	0.0154	0.0326	4.61	K03045	DNA-directed RNA polymerase subunit B" [EC:2.7.7.6]	Purine metabolism-Pyrimidine metabolism-RNA polymerase
RNA	High	0.18	0.85	0.0252	0.0067	4.66	K03053	DNA-directed RNA polymerase subunit H [EC:2.7.7.6]	Purine metabolism-Pyrimidine metabolism-RNA polymerase
RNA	High	1.02	4.4	0.0236	0.0297	4.3	K03124	transcription initiation factor TFIIB	Basal transcription factors-Epstein-Barr virus infection-Viral carcinogenesis
RNA	High	0.98	2.65	0.0136	0.0191	2.71	K03167	DNA topoisomerase VI subunit B [EC:5.99.1.3]	-
RNA	High	0.18	0.62	0.0295	0.0429	3.41	K03226	type III secretion protein SctR	Bacterial secretion system
RNA	High	0.07	0.33	0.0169	0.0364	4.47	K04795	fibrillarin-like pre-rRNA processing protein	-
RNA	High	0.29	0.98	0.0179	0.0067	3.41	K04801	replication factor C small subunit	-
RNA	High	0.00	0.04	0.0337	0.0191	14.93	K04857	voltage-dependent calcium channel L type alpha-1S	MAPK signaling pathway-Calcium signaling pathway-Cardiac muscle contraction-Adrenergic signaling in cardiomyocytes-Vascular smooth muscle contraction-Retrograde endocannabinoid signaling-Cholinergic synapse-Serotonergic synapse-GABAergic synapse-Insulin secretion-GnRH signaling pathway-Oxytocin signaling pathway-Alzheimer's disease-Hypertrophic cardiomyopathy (HCM)-Arrhythmogenic right ventricular cardiomyopathy (ARVC)-Dilated cardiomyopathy
RNA	High	2.59	6.43	0.0102	0.0067	2.49	K06863	5-formaminoimidazole-4-carboxamide-1-(beta)-D-ribofuranosyl 5'-monophosphate synthetase [EC:6.3.4.23]	Purine metabolism

RNA	High	28.38	53.21	0.0279	0.0285	1.87	K06907	Phage tail sheath protein FI	-
RNA	High	0.02	0.21	0.0219	0.0212	10.7	K06913	-	-
RNA	High	0.09	0.78	0.017	0.0067	9.01	K06927	diphthine-ammonia ligase [EC:6.3.1.14]	-
RNA	High	0.63	2.02	0.0204	0.0191	3.22	K07041		-
RNA	High	0.07	0.35	0.0212	0.0067	4.88	K07135	-	-
RNA	High	0.13	0.47	0.0168	0.0364	3.65	K07142	-	-
RNA	High	1.25	2.27	0.0312	0.0494	1.82	K07318	adenine-specific DNA-methyltransferase [EC:2.1.1.72]	-
RNA	High	0.85	1.93	0.0167	0.0212	2.27	K07463	archaea-specific RecJ-like exonuclease	-
RNA	High	0.19	0.76	0.0106	0.0067	3.9	K07558	tRNA nucleotidyltransferase (CCA-adding enzyme) [EC:2.7.7.72]	-
RNA	High	0.13	0.7	0.0189	0.0212	5.26	K07569	RNA-binding protein	-
RNA	High	0.44	1.36	0.0215	0.0067	3.08	K07582	-	-
RNA	High	0.03	0.21	0.0262	0.0252	7.42	K07732	riboflavin kinase, archaea type [EC:2.7.1.161]	Riboflavin metabolism
RNA	High	0.71	3.19	0.0202	0.0067	4.52	K09482	glutamyl-tRNA(Gln) amidotransferase subunit D [EC:6.3.5.7]	Aminoacyl-tRNA biosynthesis
RNA	High	2.70	5.23	0.0191	0.0067	1.93	K09935	hypothetical protein	-
RNA	High	0.48	1.9	0.0165	0.0067	3.97	K10725	archaeal cell division control protein 6	-
RNA	High	0.34	1.17	0.0223	0.0126	3.42	K10896	fanconi anemia group M protein	Fanconi anemia pathway
RNA	High	0.02	0.09	0.0266	0.0438	5.79	K11404	histone deacetylase 3 [EC:3.5.1.98]	Thyroid hormone signaling pathway-Alcoholism-Viral carcinogenesis
RNA	High	0.01	0.13	0.0239	0.0191	12.66	K12739	peptidyl-prolyl cis-trans isomerase-like 6 [EC:5.2.1.8]	-
RNA	High	0.07	0.36	0.0289	0.0067	5.03	K13886	coronin-1B	-
RNA	High	0.02	0.13	0.0239	0.0297	5.23	K14995	solute carrier family 38 (sodium-coupled neutral amino acid transporter), member 9	-
RNA	Low	2.72	0.93	-0.0158	0.0326	2.91	K00004	(R,R)-butanediol dehydrogenase / meso-butanediol dehydrogenase / diacetyl reductase [EC:1.1.1.4 1.1.1.- 1.1.1.303]	Butanoate metabolism
RNA	Low	324.93	139.22	-0.024	0.0067	2.33	K00074	3-hydroxybutyryl-CoA dehydrogenase [EC:1.1.1.157]	Phenylalanine metabolism-Benzoate degradation-Butanoate metabolism
RNA	Low	528.65	408.92	-0.0276	0.0164	1.29	K00100	Oxidoreductase	Fructose and mannose metabolism-Bisphenol degradation-Linoleic acid metabolism-Chloroalkane and chloroalkene degradation-Butanoate metabolism
RNA	Low	172.02	58.11	-0.025	0.0067	2.96	K00104	glycolate oxidase [EC:1.1.3.15]	Glyoxylate and dicarboxylate metabolism
RNA	Low	24.88	4.44	-0.0143	0.0067	5.6	K00158	pyruvate oxidase [EC:1.2.3.3]	Pyruvate metabolism
RNA	Low	16.77	6.55	-0.0211	0.0067	2.56	K00194	acetyl-CoA decarbonylase/synthase complex subunit delta [EC:2.1.1.245]	Methane metabolism-Carbon fixation pathways in prokaryotes-Carbon metabolism
RNA	Low	23.86	9.93	-0.022	0.0067	2.4	K00197	acetyl-CoA decarbonylase/synthase complex subunit gamma [EC:2.1.1.245]	Methane metabolism-Carbon fixation pathways in prokaryotes-Carbon metabolism
RNA	Low	43.30	17.71	-0.0147	0.0067	2.44	K00198	carbon-monoxide dehydrogenase catalytic subunit [EC:1.2.99.2 1.2.7.4]	Nitrotoluene degradation-Methane metabolism-Carbon fixation pathways in prokaryotes-Carbon metabolism
RNA	Low	347.43	179.21	-0.0117	0.0374	1.94	K00248	butyryl-CoA dehydrogenase [EC:1.3.8.1]	Fatty acid degradation-Valine, leucine and isoleucine degradation-Butanoate metabolism-Carbon metabolism-Fatty acid metabolism
RNA	Low	23.31	10.1	-0.0239	0.0067	2.31	K00249	acyl-CoA dehydrogenase [EC:1.3.8.7]	Fatty acid degradation-Valine, leucine and isoleucine degradation-beta-Alanine metabolism-Propanoate metabolism-Carbon metabolism-Fatty acid metabolism-PPAR signaling pathway

RNA	Low	85.50	57.63	-0.0135	0.0164	1.48	K00286	pyrroline-5-carboxylate reductase [EC:1.5.1.2]	Arginine and proline metabolism-Biosynthesis of amino acids
RNA	Low	14.59	6.76	-0.0131	0.0191	2.16	K00375	GntR family transcriptional regulator / MocR family aminotransferase	-
RNA	Low	47.17	14.81	-0.0186	0.0067	3.18	K00385	anaerobic sulfite reductase subunit C	Sulfur metabolism
RNA	Low	168.04	110.66	-0.0148	0.0381	1.52	K00528	ferredoxin--NADP+ reductase [EC:1.18.1.2]	-
RNA	Low	216.09	176.8	-0.0164	0.0067	1.22	K00540	Oxidoreductase	-
RNA	Low	6.71	2.93	-0.0092	0.0126	2.29	K00547	homocysteine S-methyltransferase [EC:2.1.1.10]	Cysteine and methionine metabolism
RNA	Low	173.29	127.1	-0.0176	0.0067	1.36	K00609	aspartate carbamoyltransferase catalytic subunit [EC:2.1.3.2]	Pyrimidine metabolism-Alanine, aspartate and glutamate metabolism
RNA	Low	461.33	145.59	-0.018	0.0191	3.17	K00626	acetyl-CoA C-acetyltransferase [EC:2.3.1.9]	Fatty acid degradation-Synthesis and degradation of ketone bodies-Valine, leucine and isoleucine degradation-Lysine degradation-Benzoate degradation-Tryptophan metabolism-Pyruvate metabolism-Glyoxylate and dicarboxylate metabolism-Propanoate metabolism-Butanoate metabolism-Carbon fixation pathways in prokaryotes-Terpenoid backbone biosynthesis-Carbon metabolism-Fatty acid metabolism-Two-component system
RNA	Low	6.07	1.75	-0.0212	0.0374	3.48	K00632	acetyl-CoA acyltransferase [EC:2.3.1.16]	Fatty acid degradation-Valine, leucine and isoleucine degradation-Geraniol degradation-Benzoate degradation-alpha-Linolenic acid metabolism-Ethylbenzene degradation-Fatty acid metabolism
RNA	Low	12.50	6.18	-0.016	0.0067	2.02	K00674	2,3,4,5-tetrahydropyridine-2-carboxylate N-succinyltransferase [EC:2.3.1.117]	Lysine biosynthesis-Biosynthesis of amino acids
RNA	Low	4.28	2.14	-0.0219	0.0067	2	K00842	aminotransferase [EC:2.6.1.-]	-
RNA	Low	0.54	0.1	-0.0147	0.0212	5.65	K00883	2-dehydro-3-deoxygalactonokinase [EC:2.7.1.58]	Galactose metabolism
RNA	Low	6.96	3.58	-0.0174	0.0438	1.94	K00901	diacylglycerol kinase (ATP dependent) [EC:2.7.1.107]	Glycerolipid metabolism-Glycerophospholipid metabolism-Phosphatidylinositol signaling system
RNA	Low	40.32	16.44	-0.0146	0.0326	2.45	K01026	propionate CoA-transferase [EC:2.8.3.1]	Pyruvate metabolism-Propanoate metabolism-Styrene degradation
RNA	Low	4.46	2.37	-0.0192	0.0212	1.88	K01076	Thioester hydrolase	Limonene and pinene degradation-Biosynthesis of unsaturated fatty acids
RNA	Low	2.08	0.32	-0.0151	0.0067	6.4	K01220	6-phospho-beta-galactosidase [EC:3.2.1.85]	Galactose metabolism
RNA	Low	1.71	0.22	-0.0188	0.0067	7.7	K01232	maltose-6'-phosphate glucosidase [EC:3.2.1.122]	Starch and sucrose metabolism
RNA	Low	46.56	10.51	-0.0164	0.0067	4.43	K01442	choloylglycine hydrolase [EC:3.5.1.24]	Primary bile acid biosynthesis-Secondary bile acid biosynthesis
RNA	Low	57.79	29.93	-0.0191	0.0067	1.93	K01443	N-acetylglucosamine-6-phosphate deacetylase [EC:3.5.1.25]	Amino sugar and nucleotide sugar metabolism
RNA	Low	12.80	6.76	-0.0153	0.0212	1.89	K01451	hippurate hydrolase [EC:3.5.1.32]	Phenylalanine metabolism
RNA	Low	3.20	1.44	-0.0239	0.0067	2.23	K01455	formamidase [EC:3.5.1.49]	Glyoxylate and dicarboxylate metabolism-Nitrogen metabolism-Carbon metabolism
RNA	Low	5.47	2.4	-0.0213	0.0067	2.28	K01501	nitrilase [EC:3.5.5.1]	Tryptophan metabolism-Cyanoamino acid metabolism-Aminobenzoate degradation-Styrene degradation-Nitrogen metabolism
RNA	Low	2.53	0.09	-0.0271	0.0191	27.35	K01577	oxalyl-CoA decarboxylase [EC:4.1.1.8]	Glyoxylate and dicarboxylate metabolism
RNA	Low	198.49	132.57	-0.0158	0.0394	1.5	K01615	glutaconyl-CoA decarboxylase [EC:4.1.1.70]	Benzoate degradation-Butanoate metabolism
RNA	Low	70.43	44.11	-0.0202	0.0067	1.6	K01626	3-deoxy-7-phosphoheptulonate synthase [EC:2.5.1.54]	Phenylalanine, tyrosine and tryptophan biosynthesis-Biosynthesis of amino acids
RNA	Low	453.43	303.59	-0.0114	0.0269	1.49	K01687	dihydroxy-acid dehydratase [EC:4.2.1.9]	Valine, leucine and isoleucine biosynthesis-Pantothenate and CoA biosynthesis-2-Oxocarboxylic acid metabolism-Biosynthesis of amino acids

RNA	Low	19.07	3.81	-0.0269	0.0067	5	K01788	N-acylglucosamine-6-phosphate 2-epimerase [EC:5.1.3.9]	Amino sugar and nucleotide sugar metabolism
RNA	Low	435.07	250.49	-0.0202	0.0164	1.74	K01848	methylmalonyl-CoA mutase, N-terminal domain [EC:5.4.99.2]	Valine, leucine and isoleucine degradation-Propanoate metabolism-Carbon fixation pathways in prokaryotes-Carbon metabolism
RNA	Low	90.04	52.8	-0.0185	0.0164	1.71	K01849	methylmalonyl-CoA mutase, C-terminal domain [EC:5.4.99.2]	Valine, leucine and isoleucine degradation-Propanoate metabolism-Carbon fixation pathways in prokaryotes-Carbon metabolism
RNA	Low	121.21	49.39	-0.0202	0.0067	2.45	K01989	putative ABC transport system substrate-binding protein	-
RNA	Low	19.88	8.39	-0.0253	0.0067	2.37	K02007	cobalt/nickel transport system permease protein	ABC transporters
RNA	Low	156.96	98.04	-0.0164	0.0164	1.6	K02028	polar amino acid transport system ATP-binding protein [EC:3.6.3.21]	-
RNA	Low	199.63	129.86	-0.0146	0.0212	1.54	K02029	polar amino acid transport system permease protein	-
RNA	Low	43.48	2.28	-0.0239	0.0067	19.07	K02746	PTS system, N-acetylgalactosamine-specific IIC component	Galactose metabolism-Phosphotransferase system (PTS)
RNA	Low	53.67	2.93	-0.0198	0.0067	18.3	K02747	PTS system, N-acetylgalactosamine-specific IID component	Galactose metabolism-Phosphotransferase system (PTS)
RNA	Low	1.79	0.73	-0.0154	0.0413	2.46	K02756	PTS system, beta-glucosides-specific IIB component [EC:2.7.1.69]	Phosphotransferase system (PTS)
RNA	Low	132.00	29.88	-0.0109	0.0067	4.42	K02760	PTS system, cellobiose-specific IIB component [EC:2.7.1.69]	Phosphotransferase system (PTS)
RNA	Low	268.84	72.75	-0.0098	0.0191	3.7	K02761	PTS system, cellobiose-specific IIC component	Phosphotransferase system (PTS)
RNA	Low	1.90	0.52	-0.0205	0.0067	3.68	K02774	PTS system, galactitol-specific IIB component [EC:2.7.1.69]	Galactose metabolism-Phosphotransferase system (PTS)
RNA	Low	484.38	212.72	-0.0186	0.0164	2.28	K02793	PTS system, mannose-specific IIA component [EC:2.7.1.69]	Fructose and mannose metabolism-Amino sugar and nucleotide sugar metabolism-Phosphotransferase system (PTS)
RNA	Low	17.99	11.85	-0.0184	0.0269	1.52	K02825	pyrimidine operon attenuation protein / uracil phosphoribosyltransferase [EC:2.4.2.9]	Pyrimidine metabolism
RNA	Low	1.79	0.52	-0.0154	0.0067	3.43	K03478	hypothetical protein	-
RNA	Low	8.65	4.03	-0.0206	0.0067	2.15	K03484	LacI family transcriptional regulator, sucrose operon repressor	-
RNA	Low	2.37	0.26	-0.0161	0.0067	8.97	K03492	GntR family transcriptional regulator	-
RNA	Low	426.67	242.32	-0.0163	0.0191	1.76	K03521	electron transfer flavoprotein beta subunit	-
RNA	Low	514.93	321.65	-0.0133	0.0191	1.6	K03522	electron transfer flavoprotein alpha subunit	-
RNA	Low	77.4	19.49	-0.0164	0.0067	3.97	K03688	ubiquinone biosynthesis protein	Ubiquinone and other terpenoid-quinone biosynthesis
RNA	Low	9.39	4.74	-0.0178	0.0269	1.98	K03722	ATP-dependent DNA helicase DinG [EC:3.6.4.12]	-
RNA	Low	2.70	0.3	-0.0249	0.0067	8.94	K03741	arsenate reductase [EC:1.20.4.1]	-
RNA	Low	6.89	3.13	-0.0172	0.0067	2.2	K03785	3-dehydroquinate dehydratase I [EC:4.2.1.10]	Phenylalanine, tyrosine and tryptophan biosynthesis-Biosynthesis of amino acids
RNA	Low	2.19	1.02	-0.0244	0.0067	2.13	K04014	protein NrfC	-
RNA	Low	1.27	0.46	-0.0134	0.0252	2.75	K05339	holin-like protein LrgB	Two-component system
RNA	Low	2.61	1.46	-0.0248	0.0297	1.79	K05587	bidirectional [NiFe] hydrogenase diaphorase subunit [EC:1.6.5.3]	Oxidative phosphorylation
RNA	Low	1.56	0.51	-0.0211	0.0126	3.05	K05817	LysR family transcriptional regulator, hca operon transcriptional activator	-

RNA	Low	62.45	22.12	-0.0182	0.0067	2.82	K05832	putative ABC transport system permease protein	-
RNA	Low	2.83	1.09	-0.0163	0.0067	2.59	K05847	osmoprotectant transport system ATP-binding protein	ABC transporters
RNA	Low	220.98	69.23	-0.0188	0.0067	3.19	K05919	superoxide reductase [EC:1.15.1.2]	-
RNA	Low	2.29	1.14	-0.0089	0.0381	2.01	K06518	holin-like protein	-
RNA	Low	29.95	9.78	-0.0098	0.0212	3.06	K06902	MFS transporter, UMF1 family	-
RNA	Low	88.09	59.89	-0.0197	0.0067	1.47	K07024	unknown hydrolase	-
RNA	Low	16.75	10.5	-0.0095	0.0347	1.6	K07050	misacylated tRNA(Ala) deacylase [EC:3.1.1.-]	-
RNA	Low	0.90	0.34	-0.0316	0.0067	2.63	K07309	Tat-targeted selenate reductase subunit YnfE [EC:1.97.1.9]	Selenocompound metabolism
RNA	Low	19.54	13.81	-0.0148	0.0454	1.41	K07473	DNA-damage-inducible protein J	-
RNA	Low	0.54	0.14	-0.0178	0.0067	3.93	K07508	acetyl-CoA acyltransferase 2 [EC:2.3.1.16]	Fatty acid elongation-Fatty acid degradation-Valine, leucine and isoleucine degradation-Benzoate degradation-Fatty acid metabolism
RNA	Low	15.69	5.38	-0.0267	0.0067	2.92	K07749	formyl-CoA transferase [EC:2.8.3.16]	-
RNA	Low	0.81	0.17	-0.0159	0.0067	4.73	K07811	trimethylamine-N-oxide reductase (cytochrome c) [EC:1.7.2.3]	Two-component system
RNA	Low	6.66	3.84	-0.0218	0.0067	1.74	K08302	tagatose 1,6-diphosphate aldolase GatY/KbaY [EC:4.1.2.40]	Galactose metabolism
RNA	Low	41.63	26.18	-0.0151	0.0191	1.59	K08978	bacterial/archaeal transporter family protein	-
RNA	Low	123.19	87.11	-0.0099	0.0232	1.41	K09157	hypothetical protein	-
RNA	Low	0.12	0.00	-0.0114	0.0381	37.79	K09611	probable aminopeptidase NPEPL1 [EC:3.4.11.-]	-
RNA	Low	89.44	14.69	-0.0143	0.0067	6.09	K09825	Fur family transcriptional regulator, peroxide stress response regulator	-
RNA	Low	11.88	6.84	-0.0226	0.0232	1.74	K10010	cystine transport system ATP-binding protein [EC:3.6.3.-]	ABC transporters
RNA	Low	16.68	8.53	-0.0206	0.0191	1.96	K10036	glutamine transport system substrate-binding protein	ABC transporters
RNA	Low	9.77	5.68	-0.0186	0.0252	1.72	K10118	raffinose/stachyose/melibiose transport system permease protein	ABC transporters
RNA	Low	44.22	19.6	-0.0237	0.0413	2.26	K10192	oligogalacturonide transport system substrate-binding protein	ABC transporters
RNA	Low	0.22	0.03	-0.0327	0.0067	7.46	K12261	2-hydroxyacyl-CoA lyase 1 [EC:4.1.-.-]	Peroxisome
RNA	Low	2.81	1.19	-0.0255	0.0067	2.36	K12297	23S rRNA (guanine2445-N2)-methyltransferase / 23S rRNA (guanine2069-N7)-methyltransferase [EC:2.1.1.173 2.1.1.264]	-
RNA	Low	0.33	0.08	-0.0128	0.0316	3.9	K12554	alanine adding enzyme [EC:2.3.2.-]	Peptidoglycan biosynthesis
RNA	Low	0.26	0.07	-0.0285	0.047	3.87	K13794	LysR family transcriptional regulator, regulatory protein for tcuABC	-
RNA	Low	1.83	0.95	-0.0261	0.047	1.91	K13921	1-propanol dehydrogenase	Glycerolipid metabolism
RNA	Low	44.14	19.7	-0.0206	0.0164	2.24	K14138	acetyl-CoA synthase [EC:2.3.1.169]	Carbon fixation pathways in prokaryotes-Carbon metabolism
RNA	Low	29.5	18.05	-0.0218	0.0252	1.63	K14170	chorismate mutase / prephenate dehydratase [EC:5.4.99.5 4.2.1.51]	Phenylalanine, tyrosine and tryptophan biosynthesis-Biosynthesis of amino acids
RNA	Low	2.26	0.97	-0.0227	0.0285	2.33	K15023	5-methyltetrahydrofolate corrinoid/iron sulfur protein methyltransferase [EC:2.1.1.258]	Carbon fixation pathways in prokaryotes-Carbon metabolism
