# Supplementary material for: Rumen metagenome and metatranscriptome analyses of low methane yield sheep reveals a Sharpea-enriched microbiome characterised by lactic acid formation and utilisation
Source: Microbiome. 2016 Oct 19;4:56. doi: 10.1186/s40168-016-0201-2 (PMC5069950; doi:10.1186/s40168-016-0201-2)
Supplement: Additional file 4: Table S3. — Gene set enrichment analysis of metagenome and metatranscriptome datasets. Pathways with genes differentially present or expressed based on nominal P value (NOM P val ≤0.05) are shown, ranked by normalised enrichment score (NES). Corrected P values for false discovery rate (FDR q val) and familywise-error rate (FEWER P val) are shown. (PDF 88 kb) [file 40168_2016_201_MOESM4_ESM.pdf]

Table S3. Gene set enrichment analysis of metagenome and metatranscriptome datasets. Pathways with genes differentially present or expressed based on nominal *P* value (NOM *P*-val ≤ 0.05) are shown, ranked by normalised enrichment score (NES). Corrected *P* values for false discovery rate (FDR q-val) and familywise-error rate (FEWER *P*-val) are shown.

Methane yield group	KEGG Pathway	NES	NOM *P*-val	FDR q-val	FWER *P*-val
Metagenome					
Low	ko00052-Galactose metabolism	-1.97	0.003	0.017	0.015
Low	ko02060-Phosphotransferase systems (PTS)	-1.82	0.015	0.092	0.120
Low	ko00440-Phosphonate and phosphinate metabolism	-1.62	0.012	0.734	0.602
Low	ko00561- Glycerolipid metabolism	-1.58	0.047	0.544	0.701
Low	ko00290- Valine, leucine, isoleucine biosynthesis	-1.57	0.036	0.496	0.716
Low	ko00791- Atrazine degradation	-1.53	0.040	0.608	0.801
Low	ko01212- Fatty acid metabolism	-1.45	0.043	0.786	0.909
High	ko03070-Bacterial secretion system	1.70	0.019	0.470	0.386
High	ko00983- Drug metabolism and other enzymes	1.55	0.039	0.688	0.773
High	ko00250- Alanine, asparate and glutamate metabolism	1.55	0.036	0.538	0.782
Metatranscriptome					
Low	ko00052-Galactose metabolism	-1.72	0.010	0.511	0.270
Low	ko00630-Gyloxylate and dicarboxylate metabolism	-1.69	0.005	0.362	0.348
Low	ko00720-Carbon fixation pathways in prokaryotes	-1.64	0.010	0.427	0.507
Low	ko01230-Biosynthesis of amino acids	-1.61	0.035	0.393	0.570
Low	ko00550-Peptidoglycan biosynthesis	-1.60	0.020	0.365	0.612
Low	ko00300-Lysine biosynthesis	-1.58	0.030	0.316	0.669
Low	ko00290-Valine, leucine, isoleucine biosynthesis	-1.55	0.040	0.311	0.736
Low	ko00450-Selenocompund metabolism	-1.52	0.033	0.311	0.792
Low	ko00330-Arginine and proline metabolism	-1.48	0.038	0.373	0.870
