# Supplementary material for: Rumen metagenome and metatranscriptome analyses of low methane yield sheep reveals a Sharpea-enriched microbiome characterised by lactic acid formation and utilisation
Source: Microbiome. 2016 Oct 19;4:56. doi: 10.1186/s40168-016-0201-2 (PMC5069950; doi:10.1186/s40168-016-0201-2)
Supplement: Additional file 5: Text S1. — Detailed results for the statistical analysis and additional methods. (DOCX 40 kb) [file 40168_2016_201_MOESM5_ESM.docx]

**Supplementary Text S1**

KEGG genes consistently identified in multiple statistical analyses

The metagenome sequence reads mapped to 10,272 KEGG genes while the metatranscriptome reads mapped to 6,626 KEGG genes. Read count matrix datasets based on these gene and transcript mappings were used for statistical analyses. We previously identified 297 KEGG genes in the metagenome dataset and 350 KEGG genes in the metatranscriptome dataset ([Shi *et al.*, 2014](#_ENREF_11)) that were significantly different between HMY and LMY animal categories using Wilcoxon Rank Sum test (WRS). Subsequently, we applied sparse partial least squares regression analysis (sPLS) to identify correlations between gene or transcript abundances and animal methane yield values. This identified 308 KEGG genes from the metagenome data and 162 from metatranscriptome data that were significantly correlated with methane yield.

Comparison of the WRS and sPLS statistical analyses for the metagenome data identified 185 genes that showed significant correlation with methane yield in both analyses; 60 genes correlated with HMY, and 125 with LMY. Many of the genes positively correlated with methane yield were related to bacterial secretion systems, especially Type III Secretion Systems (T3SSs, Table S3). Genes negatively correlated with methane yield were mainly associated with phosphotransferase systems (PTS) and galactose metabolism (Table S3). Additionally, some ABC transporters for phosphate and osmoprotectant, and genes related to amino acid metabolism (aromatic amino acids, cysteine and methionine) were also correlated to LMY. In the metatranscriptome data, 129 gene transcripts were related to methane yield (36 correlated to HMY, 93 to LMY). Among the transcripts associated with HMY animals, the main transcripts identified were related to DNA polymerase genes and subunits of the methyl-coenzyme M reductase enzyme (Table S3) which catalyses the final step in the methanogenesis pathway. Transcripts associated with LMY fell into the categories of galactose metabolism and PTS as well as amino acid biosynthesis and short chain fatty acid metabolism (Table S3).

Gene set enrichment analysis

Gene set enrichment analysis (GSEA) resulted in a similar representation of gene categories as was found in the WRS tests and sPLS regressions. For the HMY animals, the Bacterial secretion system gene set (ko03070) showed particular enrichment while Drug metabolism (ko00983) and Alanine, Asparate and Glutamate metabolism (ko00250) were also significantly enriched (Table S2). For the LMY animals, Galactose metabolism (ko00052) and PTS (ko02060), showed significant enrichment with a false discovery rate of <0.25% and *P* values < 0.01% and <0.05%, respectively. Five additional gene sets were also significantly enriched; Phosphonate and phosphinate metabolism (ko00440), Glycerolipid metabolism (ko00561), Valine, leucine and isoleucine biosynthesis (ko00290), Atrazine degradation (ko00791) and Fatty acid metabolism (ko01212) (Table S2).

In the metatranscriptome dataset, no significant up-regulation was detected in any of the gene sets that were associated with a HMY phenotype. For the LMY animals, two gene sets were particularly enriched; Galactose metabolism (ko00052) and glyoxylate and dicarboxylate metabolism (ko00630), while a further eight gene sets showed significant enrichment including several pathways related to amino acid metabolism (Table S2).

**Methodological details:**

Analysis of fermentation acids

Analysis of fermentation acids from rumen samples was performed on a Shimadzu 5050a GC-MS (Shimadzu, Kyoto, Japan) equipped with a ZB-5 MS (30 m x 0.25 mm ID x 0.25 µm film thickness, Phenomenex, USA) capillary column.  Acids were extracted from the acidified samples with ether and derivatised to their *t*-butyl-dimethylsilyl esters following the method described by Richardson et al. (1989), which detects both volatile and non-volatile fatty acid acids.  Selected ion acquisition was performed to monitor the following ions: formic, (103 m/z); acetic, (117 m/z); propionic, (131 m/z); butyric and isobutyric, (145 m/z); valeric, isovaleric and 2-methylcaproic, (159 m/z); caproic and 2-ethylbutyric, (163 m/z); lactic, (243 m/z); succinic (263 m/z) and fumaric, (291 m/z).  A calibration of the detector responses for each individual volatile fatty acid relative to 2-ethylbutryric acid was determined with each batch of samples analysed using a solution containing known quantities of organic acids. Statistical analysis was conducted using non-parametric testing via two-group Wilcoxon Rank Sum test in R to test for differences in volatile fatty acid concentration between HMY and LMY samples.

16S rRNA gene amplicon sequencing and data analysis

Amplicon sequence data of bacterial 16S rRNA genes from DNA extracted from all 44 rumen samples were generated analogous to amplicon sequencing data of archaeal 16S rRNA genes ([Shi *et al.* (2014](#_ENREF_11)) and analysed using the QIIME pipeline ([Caporaso *et al.*, 2010](#_ENREF_1)). Briefly, 454 Titanium pyrosequencing reads were size and quality filtered using default parameters in QIIME and mapped back to the original sample by using the 12-bp error-correcting Golay barcodes. Reads were clustered into operational taxonomic units (OTUs) with 97% sequence similarity cut-off using uclust ([Edgar, 2010](#_ENREF_4)). Phylogenetic affiliations were assigned to each OTU based on the Greengenes database (version gg_13_5) ([McDonald *et al.*, 2012](#_ENREF_8)). Relative abundance tables were generated at the species level, and statistical analysis including all taxa with a relative abundance of at least 0.2% in one or more samples was conducted. We performed one-way analysis of variance (ANOVA) in QIIME to identify differences between bacterial communities of high and low methane yielding animals. Relative abundances were also used to estimate the correlation of individual species-level taxa with methane yield using Spearman’s Rank. A value of p ≤ 0.001 was regarded as statistically significant. Principal coordinate analysis was conducted in QIIME to identify community-wide differences between high, low and intermediate samples, and clustering of samples was visualised using Sigmaplot version 13.0b (Systat Software Inc., San Jose, CA, USA).

Processing of metagenome and metatranscriptome data

Metagenomic datasets analysed in this study include 20 tight insert (250 bp) libraries from selected high, low and intermediate methane yield samples (Table S1) that were sequenced from both ends (2x150bp) using Illumina HiSeq2000 technology. Additionally two 8kb insert libraries from combined samples of either all low or high methane yield samples were sequenced using Illumina HiSeq 2000 technology. Sequence reads were quality filtered, screened and filtered for contaminating host DNA analyses as described previously ([Shi *et al.*, 2014](#_ENREF_11)). Sequencing reads that matched small subunit ribosomal RNA (ssRNA) genes based on screening against SILVA ([Pruesse *et al.*, 2007](#_ENREF_9)), Greengenes ([DeSantis *et al.*, 2006](#_ENREF_3)) and RDP ([Cole *et al.*, 2009](#_ENREF_2)) databases were filtered out of the dataset and processed analogous to the ssRNA gene amplicon data described above. Thus filtered paired end reads were merged using FLASH (Magoc and Salzberg, 2011) and used for readmapping analyses. Metatranscriptomic sequencing was conducted for the same 20 samples as in the metagenomes datasets (Table S1) by mRNA enrichment, transcription into double-stranded cDNA, Illumina TruSeq library preparation and sequencing (2x150bp) on the Illumina HiSeq 2000 platform as previously described ([Shi *et al.*, 2014](#_ENREF_11)). Datasets were quality and artifact filtered and paired end read merged using FLASH and used for read count based comparative analysis as described previously ([Shi *et al.*, 2014](#_ENREF_11)).

Metagenome and metatranscriptome read-based annotation

Quality and rRNA filtered, FLASH merged read data from both the tight insert library metagenome and metatranscriptome datasets were screened against the KEGG database, version 58.1 ([Kanehisa & Goto, 2000](#_ENREF_6)) using USEARCH 6.0 ([Edgar, 2010](#_ENREF_4)) at an E-value cutoff of 1×10^-5^  and read count matrices were constructed and normalised to reads per million (RPM).

*Ldh* gene re-assemblies

Individual assemblies based on the 20 metagenome and 20 metatranscriptome datasets were constructed as previously described ([Shi *et al.*, 2014](#_ENREF_11)). Assemblies were combined, and duplicated contigs, or smaller contigs covered by larger ones, were removed using the clustering function in Vmatch (<http://www.vmatch.de>). The resulting contigs were submitted to IMG/Mer ([Markowitz *et al.*, 2014](#_ENREF_7)) for gene-calling and automatic annotation. All assembled genes with hits to the relevant K00016 were exported and combined with the corresponding raw-reads with hits to K00016 genes from metagenome and metatranscriptome data. Using these reads, contigs were extended using the Distributed Nucleating Assembler function in Kmernator (<https://github.com/JGI-Bioinformatics/Kmernator>). Genes on the resulting contigs were predicted using MetaGeneMark ([Zhu *et al.*, 2010](#_ENREF_12)).

Lactoyl-CoA dehydrogenase subunit alpha gene (*lcdA*) database

For *lcdA* genes a custom database for screening purposes was created based on reference sequences of *Clostridium propionicum* (*lcdA* accession: AEM62994) which is known to use the acrylate pathway for propionate formation ([Hetzel *et al.*, 2003](#_ENREF_5)). BlastP searches using the *lcdA* sequence against the IMG database were conducted using a 30% aa sequence identity cut-off. The gene neighbourhoods of all hits were checked for the presence of propionate CoA-transferase genes two genes upstream of *lcdA* and the presence of *lcdB* and *lcdC* genes to ensure identity of lactoyl-CoA dehydrogenase genes as described previously ([Reichardt *et al.*, 2014](#_ENREF_10)). Genes not fulfilling these criteria were excluded. Nucleotide sequences of all remaining hits were exported from IMG and combined with all *lcdA* sequences from uncultured organisms of the human gut retrieved by Reichardt *et al*. ([2014](#_ENREF_10)) creating a database of 120 *lcdA* gene sequences.

**References**

Caporaso, J. G., Kuczynski, J., Stombaugh, J., Bittinger, K., Bushman, F. D., Costello, E. K.*, et al.* (2010). QIIME allows analysis of high-throughput community sequencing data. *Nat Methods* **7,** 335-6.

Cole, J. R., Wang, Q., Cardenas, E., Fish, J., Chai, B., Farris, R. J.*, et al.* (2009). The Ribosomal Database Project: improved alignments and new tools for rRNA analysis. *Nucleic Acids Res* **37,** D141-D145.

DeSantis, T. Z., Hugenholtz, P., Larsen, N., Rojas, M., Brodie, E. L., Keller, K.*, et al.* (2006). Greengenes, a chimera-checked 16S rRNA gene database and workbench compatible with ARB. *Appl Environ Microbiol* **72,** 5069-72.

Edgar, R. C. (2010). Search and clustering orders of magnitude faster than BLAST. *Bioinformatics* **26,** 2460-2461.

Hetzel, M., Brock, M., Selmer, T., Pierik, A. J., Golding, B. T. &Buckel, W. (2003). Acryloyl-CoA reductase from *Clostridium propionicum*. An enzyme complex of propionyl-CoA dehydrogenase and electron-transferring flavoprotein. *Eur J Biochem* **270,** 902-10.

Kanehisa, M.Goto, S. (2000). KEGG: kyoto encyclopedia of genes and genomes. *Nucleic Acids Res* **28,** 27-30.

Magoc T, Salzberg SL. 2011. FLASH: fast length adjustment of short reads to improve genome assemblies. *Bioinformatics* **27**(21): 2957-2963.

Markowitz, V. M., Chen, I.-M. A., Chu, K., Szeto, E., Palaniappan, K., Pillay, M.*, et al.* (2014). IMG/M 4 version of the integrated metagenome comparative analysis system. *Nucleic Acids Res* **42,** D568-D573.

McDonald, D., Price, M. N., Goodrich, J., Nawrocki, E. P., DeSantis, T. Z., Probst, A.*, et al.* (2012). An improved Greengenes taxonomy with explicit ranks for ecological and evolutionary analyses of bacteria and archaea. *ISME J* **6,** 610-8.

Pruesse, E., Quast, C., Knittel, K., Fuchs, B. M., Ludwig, W., Peplies, J.*, et al.* (2007). SILVA: a comprehensive online resource for quality checked and aligned ribosomal RNA sequence data compatible with ARB. *Nucleic Acids Res* **35,** 7188-96.

Reichardt, N., Duncan, S. H., Young, P., Belenguer, A., McWilliam Leitch, C., Scott, K. P.*, et al.* (2014). Phylogenetic distribution of three pathways for propionate production within the human gut microbiota. *ISME J* **8,** 1323-35.

Shi, W., Moon, C. D., Leahy, S. C., Kang, D., Froula, J., Kittelmann, S.*, et al.* (2014). Methane yield phenotypes linked to differential gene expression in the sheep rumen microbiome. *Genome Res* **24,** 1517-25.

Zhu, W., Lomsadze, A. &Borodovsky, M. (2010). Ab initio gene identification in metagenomic sequences. *Nucleic Acids Res* **38,** e132.
