# Supplementary material for: Rumen metagenome and metatranscriptome analyses of low methane yield sheep reveals a Sharpea-enriched microbiome characterised by lactic acid formation and utilisation
Source: Microbiome. 2016 Oct 19;4:56. doi: 10.1186/s40168-016-0201-2 (PMC5069950; doi:10.1186/s40168-016-0201-2)
Supplement: Additional file 7: Table S4. — Statistical data of KEGG genes related to low or high methane yield animals for Wilcoxon rank sum test (WRS) and sparse partial least squares analysis (sPLS). (PDF 1543 kb) [file 40168_2016_201_MOESM7_ESM.pdf]

Table S4. Statistical data of KEGG genes related to low or high methane yield animals for Wilcoxon Rank Sum test (WRS) and sparse partial least squares analysis (sPLS).

					Mean		Standard dev		WRS		sPLS		
**Subset**	**Data type**	**KEGG Gene**	**Gene name**	**DEFINITION**	**low**	**high**	**low**	**high**	**p.adj.BH**	**Fold change**	**Range2.5**	**Range97.5**	**coefficient**
Aminoacid biosynthesis	DNA	K00013	hisD	histidinol dehydrogenase [EC:1.1.1.23]	239	231	27.7	20.7	1	1.03	NA	NA	NA
Aminoacid biosynthesis	RNA	K00013	hisD	histidinol dehydrogenase [EC:1.1.1.23]	173	140	24.6	13	0.126	1.24	NA	NA	NA
Aminoacid biosynthesis	Transcripts/gene	NA	hisD	histidinol dehydrogenase [EC:1.1.1.23]	0.738	0.611	0.153	0.0773	0.274	1.21	NA	NA	NA
Aminoacid biosynthesis	DNA	K09484	qa-3	quinate dehydrogenase [EC:1.1.1.24]	0.00453	0.0589	0.00627	0.0878	0.468	13	NA	NA	NA
Aminoacid biosynthesis	DNA	K00014	aroE	shikimate dehydrogenase [EC:1.1.1.25]	175	164	19.1	11.3	0.938	1.07	NA	NA	NA
Aminoacid biosynthesis	RNA	K00014	aroE	shikimate dehydrogenase [EC:1.1.1.25]	83.1	69.2	10.3	9.37	0.142	1.2	NA	NA	NA
Aminoacid biosynthesis	Transcripts/gene	NA	aroE	3-dehydroquinate dehydratase / shikimate dehydrogenase [EC:4.2.1.10 1.1.1.25]	0.473	0.418	0.088	0.0628	0.354	1.13	NA	NA	NA
Aminoacid biosynthesis	DNA	K00016	ldh	L-lactate dehydrogenase [EC:1.1.1.27]	92.8	62.6	21.2	13.4	0.0359	1.48	-0.0102	-0.00375	-0.00691
Aminoacid biosynthesis	RNA	K00016	ldh	L-lactate dehydrogenase [EC:1.1.1.27]	76.6	193	14.9	259	0.519	2.52	NA	NA	NA
Aminoacid biosynthesis	Transcripts/gene	NA	ldh	L-lactate dehydrogenase [EC:1.1.1.27]	0.842	2.71	0.162	3.15	0.315	3.23	NA	NA	NA
Aminoacid biosynthesis	DNA	K05887	ydiB	quinate/shikimate dehydrogenase [EC:1.1.1.282]	0.548	0.232	0.289	0.103	0.0536	2.37	NA	NA	NA
Aminoacid biosynthesis	RNA	K05887	ydiB	quinate/shikimate dehydrogenase [EC:1.1.1.282]	0.138	0.0444	0.104	0.0323	0.0794	3.1	NA	NA	NA
Aminoacid biosynthesis	Transcripts/gene	NA	ydiB	quinate/shikimate dehydrogenase [EC:1.1.1.282]	0.279	0.214	0.199	0.137	0.713	1.3	NA	NA	NA
Aminoacid biosynthesis	RNA	K00003	NA	homoserine dehydrogenase [EC:1.1.1.3]	73.8	63.2	13.1	5.93	0.283	1.17	NA	NA	NA
Aminoacid biosynthesis	Transcripts/gene	NA	NA	homoserine dehydrogenase [EC:1.1.1.3]	1.02	0.855	0.172	0.109	0.136	1.2	NA	NA	NA
Aminoacid biosynthesis	DNA	K00030	IDH3	isocitrate dehydrogenase (NAD+) [EC:1.1.1.41]	22.7	23.5	6.36	6.62	1	1.04	NA	NA	NA
Aminoacid biosynthesis	RNA	K00030	IDH3	isocitrate dehydrogenase (NAD+) [EC:1.1.1.41]	29.9	13.5	33.6	3.36	0.187	2.21	NA	NA	NA
Aminoacid biosynthesis	Transcripts/gene	NA	IDH3	isocitrate dehydrogenase (NAD+) [EC:1.1.1.41]	1.22	0.584	0.935	0.0806	0.125	2.09	NA	NA	NA
Aminoacid biosynthesis	DNA	K00031	icd	isocitrate dehydrogenase [EC:1.1.1.42]	128	123	22.8	22.7	1	1.04	NA	NA	NA
Aminoacid biosynthesis	RNA	K00031	icd	isocitrate dehydrogenase [EC:1.1.1.42]	149	135	36.3	29.9	0.623	1.1	NA	NA	NA
Aminoacid biosynthesis	Transcripts/gene	NA	icd	isocitrate dehydrogenase [EC:1.1.1.42]	1.2	1.12	0.4	0.24	0.838	1.08	NA	NA	NA
Aminoacid biosynthesis	DNA	K00052	leuB	3-isopropylmalate dehydrogenase [EC:1.1.1.85]	194	172	21.7	12.7	0.45	1.12	NA	NA	NA
Aminoacid biosynthesis	RNA	K00052	leuB	3-isopropylmalate dehydrogenase [EC:1.1.1.85]	217	173	51	28.8	0.255	1.25	NA	NA	NA
Aminoacid biosynthesis	Transcripts/gene	NA	leuB	3-isopropylmalate dehydrogenase [EC:1.1.1.85]	1.13	1	0.262	0.155	0.426	1.12	NA	NA	NA
Aminoacid biosynthesis	DNA	K00053	ilvC	ketol-acid reductoisomerase [EC:1.1.1.86]	200	180	24.3	11.8	0.394	1.11	NA	NA	NA
Aminoacid biosynthesis	RNA	K00053	ilvC	ketol-acid reductoisomerase [EC:1.1.1.86]	893	670	190	95.3	0.116	1.33	NA	NA	NA
Aminoacid biosynthesis	Transcripts/gene	NA	ilvC	ketol-acid reductoisomerase [EC:1.1.1.86]	4.5	3.72	0.937	0.422	0.125	1.21	NA	NA	NA
Aminoacid biosynthesis	DNA	K05824	LYS12	homoisocitrate dehydrogenase [EC:1.1.1.87]	0.13	0.0835	0.0623	0.0341	0.731	1.55	NA	NA	NA
Aminoacid biosynthesis	RNA	K05824	LYS12	homoisocitrate dehydrogenase [EC:1.1.1.87]	0.269	0.241	0.144	0.128	0.944	1.12	NA	NA	NA
Aminoacid biosynthesis	DNA	K10978	aksF	methanogen homoisocitrate dehydrogenase [EC:1.1.1.87 1.1.1.-]	0.045	0.108	0.0235	0.0515	0.132	2.41	0.00577	0.0192	0.0139
Aminoacid biosynthesis	RNA	K10978	aksF	methanogen homoisocitrate dehydrogenase [EC:1.1.1.87 1.1.1.-]	0.0653	0.091	0.0538	0.0973	0.989	1.39	NA	NA	NA
Aminoacid biosynthesis	Transcripts/gene	NA	aksF	methanogen homoisocitrate dehydrogenase [EC:1.1.1.87 1.1.1.-]	1.94	1.85	0.663	0.959	0.785	1.05	NA	NA	NA
Aminoacid biosynthesis	DNA	K00058	serA	D-3-phosphoglycerate dehydrogenase [EC:1.1.1.95]	328	330	33.4	41.9	1	1.01	NA	NA	NA
Aminoacid biosynthesis	RNA	K00058	serA	D-3-phosphoglycerate dehydrogenase [EC:1.1.1.95]	551	488	90.6	46.1	0.206	1.13	NA	NA	NA
Aminoacid biosynthesis	Transcripts/gene	NA	serA	D-3-phosphoglycerate dehydrogenase [EC:1.1.1.95]	1.7	1.5	0.366	0.225	0.31	1.14	NA	NA	NA
Aminoacid biosynthesis	DNA	K05358	quiA	quinate dehydrogenase (quinone) [EC:1.1.5.8]	0.0137	0.0116	0.00915	0.0082	1	1.18	NA	NA	NA
Aminoacid biosynthesis	Transcripts/gene	NA	NA	1,2-dihydroxy-3-keto-5-methylthiopentene dioxygenase [EC:1.13.11.53 1.13.11.54]	8.33	5.93	14.1	9.51	0.942	1.41	NA	NA	NA
Aminoacid biosynthesis	DNA	K08967	mtnD, mtnZ, ADI1	1,2-dihydroxy-3-keto-5-methylthiopentene dioxygenase [EC:1.13.11.53 1.13.11.54]	0.0126	0.0048	0.0115	0.0052	0.687	2.66	NA	NA	NA
Aminoacid biosynthesis	RNA	K08967	mtnD, mtnZ, ADI1	1,2-dihydroxy-3-keto-5-methylthiopentene dioxygenase [EC:1.13.11.53 1.13.11.54]	0.0722	0.0495	0.0723	0.0378	0.788	1.46	NA	NA	NA
Aminoacid biosynthesis	Transcripts/gene	NA	NA	1,2-dihydroxy-3-keto-5-methylthiopentene dioxygenase [EC:1.13.11.53 1.13.11.54]	8.33	5.93	14.1	9.51	0.942	1.41	NA	NA	NA
Aminoacid biosynthesis	DNA	K00500	phhA	phenylalanine-4-hydroxylase [EC:1.14.16.1]	0.0236	0.0148	0.0152	0.0106	0.795	1.6	NA	NA	NA
Aminoacid biosynthesis	DNA	K05933	NA	aminocyclopropanecarboxylate oxidase [EC:1.14.17.4]	0.00975	0.0119	0.0072	0.0099	1	1.22	NA	NA	NA
Aminoacid biosynthesis	RNA	K05933	NA	aminocyclopropanecarboxylate oxidase [EC:1.14.17.4]	0.128	0.15	0.0746	0.138	0.944	1.17	NA	NA	NA
Aminoacid biosynthesis	Transcripts/gene	NA	NA	aminocyclopropanecarboxylate oxidase [EC:1.14.17.4]	12.7	23.6	9.54	31.2	0.902	1.86	NA	NA	NA
Aminoacid biosynthesis	DNA	K00215	dapB	4-hydroxy-tetrahydrodipicolinate reductase [EC:1.17.1.8]	220	193	23	8.02	0.132	1.14	NA	NA	NA
Aminoacid biosynthesis	RNA	K00215	dapB	4-hydroxy-tetrahydrodipicolinate reductase [EC:1.17.1.8]	159	142	18.6	16	0.312	1.12	NA	NA	NA
Aminoacid biosynthesis	Transcripts/gene	NA	dapB	4-hydroxy-tetrahydrodipicolinate reductase [EC:1.17.1.8]	0.729	0.737	0.117	0.0707	0.895	1.01	NA	NA	NA
Aminoacid biosynthesis	DNA	K00133	asd	aspartate-semialdehyde dehydrogenase [EC:1.2.1.11]	278	276	24.3	15.9	1	1.01	NA	NA	NA
Aminoacid biosynthesis	RNA	K00133	asd	aspartate-semialdehyde dehydrogenase [EC:1.2.1.11]	310	248	55.1	30	0.144	1.25	NA	NA	NA
Aminoacid biosynthesis	Transcripts/gene	NA	asd	aspartate-semialdehyde dehydrogenase [EC:1.2.1.11]	1.12	0.9	0.221	0.106	0.125	1.25	NA	NA	NA
Aminoacid biosynthesis	DNA	K00134	gapA	glyceraldehyde 3-phosphate dehydrogenase [EC:1.2.1.12]	302	302	25.9	16.5	1	1	NA	NA	NA
Aminoacid biosynthesis	RNA	K00134	gapA	glyceraldehyde 3-phosphate dehydrogenase [EC:1.2.1.12]	6860	6040	1910	1500	0.519	1.14	NA	NA	NA
Aminoacid biosynthesis	Transcripts/gene	NA	gapA	glyceraldehyde 3-phosphate dehydrogenase [EC:1.2.1.12]	22.8	20.1	6	5.28	0.594	1.13	NA	NA	NA
Aminoacid biosynthesis	Transcripts/gene	NA	ALDH3A2	aldehyde dehydrogenase family 7 member A1 [EC:1.2.1.31 1.2.1.8 1.2.1.3]	0.671	0.548	0.391	0.147	0.838	1.22	NA	NA	NA
Aminoacid biosynthesis	DNA	K00143	LYS2	L-aminoadipate-semialdehyde dehydrogenase [EC:1.2.1.31]	1.78	1.63	0.471	0.32	1	1.09	NA	NA	NA
Aminoacid biosynthesis	RNA	K00143	LYS2	L-aminoadipate-semialdehyde dehydrogenase [EC:1.2.1.31]	0.251	0.391	0.0892	0.231	0.424	1.56	NA	NA	NA
Aminoacid biosynthesis	Transcripts/gene	NA	ALDH7A1	aldehyde dehydrogenase family 7 member A1 [EC:1.2.1.31 1.2.1.8 1.2.1.3]	0.229	0.34	0.143	0.147	0.274	1.48	NA	NA	NA
Aminoacid biosynthesis	RNA	K14085	ALDH7A1	aldehyde dehydrogenase family 7 member A1 [EC:1.2.1.31 1.2.1.8 1.2.1.3]	0.0592	0.0875	0.0591	0.0726	0.853	1.48	NA	NA	NA
Aminoacid biosynthesis	DNA	K00145	argC	N-acetyl-gamma-glutamyl-phosphate reductase [EC:1.2.1.38]	218	212	23.2	15.5	1	1.03	NA	NA	NA
Aminoacid biosynthesis	RNA	K00145	argC	N-acetyl-gamma-glutamyl-phosphate reductase [EC:1.2.1.38]	131	96	28.1	13	0.0736	1.37	NA	NA	NA
Aminoacid biosynthesis	Transcripts/gene	NA	ARG56	N-acetyl-gamma-glutamyl-phosphate reductase / acetylglutamate kinase [EC:1.2.1.38 2.7.2.8]	0.608	0.456	0.133	0.0649	0.125	1.33	NA	NA	NA
Aminoacid biosynthesis	DNA	K12659	ARG56	N-acetyl-gamma-glutamyl-phosphate reductase / acetylglutamate kinase [EC:1.2.1.38 2.7.2.8]	0.0028	0.0066	0.0033	0.0057	0.45	2.34	NA	NA	NA
Aminoacid biosynthesis	RNA	K12659	ARG56	N-acetyl-gamma-glutamyl-phosphate reductase / acetylglutamate kinase [EC:1.2.1.38 2.7.2.8]	0.0652	0.232	0.0637	0.126	0.0853	3.56	NA	NA	NA
Aminoacid biosynthesis	DNA	K00147	proA	glutamate-5-semialdehyde dehydrogenase [EC:1.2.1.41]	251	249	30.2	20.3	1	1.01	NA	NA	NA
Aminoacid biosynthesis	RNA	K00147	proA	glutamate-5-semialdehyde dehydrogenase [EC:1.2.1.41]	167	132	25.7	25.8	0.0822	1.26	NA	NA	NA
Aminoacid biosynthesis	Transcripts/gene	NA	ALDH18A1	delta-1-pyrroline-5-carboxylate synthetase [EC:2.7.2.11 1.2.1.41]	0.672	0.531	0.118	0.0841	0.136	1.27	NA	NA	NA
Aminoacid biosynthesis	DNA	K00150	gap2	glyceraldehyde-3-phosphate dehydrogenase (NAD(P)) [EC:1.2.1.59]	11.3	13	2.44	3.75	0.62	1.15	NA	NA	NA
Aminoacid biosynthesis	RNA	K00150	gap2	glyceraldehyde-3-phosphate dehydrogenase (NAD(P)) [EC:1.2.1.59]	13.7	13.9	7.35	3.91	0.623	1.01	NA	NA	NA
Aminoacid biosynthesis	Transcripts/gene	NA	gap2	glyceraldehyde-3-phosphate dehydrogenase (NAD(P)) [EC:1.2.1.59]	1.32	1.17	0.9	0.529	0.895	1.13	NA	NA	NA
Aminoacid biosynthesis	Transcripts/gene	NA	ALDH7A1	aldehyde dehydrogenase family 7 member A1 [EC:1.2.1.31 1.2.1.8 1.2.1.3]	1.76	1.46	1.86	0.498	0.785	1.2	NA	NA	NA
Aminoacid biosynthesis	DNA	K00210	NA	prephenate dehydrogenase [EC:1.3.1.12]	66.4	67	7.57	13	1	1.01	NA	NA	NA
Aminoacid biosynthesis	RNA	K00210	NA	prephenate dehydrogenase [EC:1.3.1.12]	91.7	57.7	26.1	26.7	0.185	1.59	NA	NA	NA
Aminoacid biosynthesis	DNA	K04517	tyrA2	prephenate dehydrogenase [EC:1.3.1.12]	97.3	93.8	14.7	19.6	1	1.04	NA	NA	NA
Aminoacid biosynthesis	RNA	K04517	tyrA2	prephenate dehydrogenase [EC:1.3.1.12]	32.9	26.1	5.56	7.09	0.229	1.26	NA	NA	NA
Aminoacid biosynthesis	Transcripts/gene	NA	tyrA	chorismate mutase / prephenate dehydrogenase [EC:5.4.99.5 1.3.1.12]	0.771	0.519	0.211	0.145	0.136	1.49	NA	NA	NA
Aminoacid biosynthesis	Transcripts/gene	NA	tyrC	cyclohexadieny/prephenate dehydrogenase [EC:1.3.1.43 1.3.1.12]	0.817	0.326	0.933	0.299	0.491	2.51	NA	NA	NA
Aminoacid biosynthesis	DNA	K00220	tyrC	cyclohexadieny/prephenate dehydrogenase [EC:1.3.1.43 1.3.1.12]	0.195	0.52	0.0799	0.407	0.252	2.66	NA	NA	NA
Aminoacid biosynthesis	RNA	K00220	tyrC	cyclohexadieny/prephenate dehydrogenase [EC:1.3.1.43 1.3.1.12]	0.142	0.161	0.165	0.163	0.788	1.13	NA	NA	NA
Aminoacid biosynthesis	DNA	K15226	tyrAa	arogenate dehydrogenase (NADP+) [EC:1.3.1.78]	0.195	0.357	0.262	0.229	0.297	1.83	NA	NA	NA
Aminoacid biosynthesis	DNA	K15227	TYRAAT	arogenate dehydrogenase (NADP+), plant [EC:1.3.1.78]	0.0186	0.0018	0.0214	0.0037	0.193	10.1	NA	NA	NA
Aminoacid biosynthesis	RNA	K00265	gltB	glutamate synthase (NADPH/NADH) large chain [EC:1.4.1.13 1.4.1.14]	43.2	21.6	33.9	17.7	0.188	2	NA	NA	NA

Aminoacid biosynthesis	Transcripts/gene	NA	gltB	glutamate synthase (NADPH/NADH) small chain [EC:1.4.1.13 1.4.1.14]	0.715	0.542	0.132	0.0644	0.125	1.32	NA	NA	NA
Aminoacid biosynthesis	DNA	K00264	GLT1	glutamate synthase (NADPH/NADH) [EC:1.4.1.13 1.4.1.14]	4.11	4.11	0.724	0.986	1	1	NA	NA	NA
Aminoacid biosynthesis	RNA	K00264	GLT1	glutamate synthase (NADPH/NADH) [EC:1.4.1.13 1.4.1.14]	1.13	0.777	0.198	0.335	0.121	1.45	NA	NA	NA
Aminoacid biosynthesis	DNA	K00265	gltB	glutamate synthase (NADPH/NADH) large chain [EC:1.4.1.13 1.4.1.14]	426	396	69.4	70.4	0.968	1.07	NA	NA	NA
Aminoacid biosynthesis	DNA	K00266	gltD	glutamate synthase (NADPH/NADH) small chain [EC:1.4.1.13 1.4.1.14]	770	738	80.6	41.9	0.968	1.04	NA	NA	NA
Aminoacid biosynthesis	RNA	K00266	gltD	glutamate synthase (NADPH/NADH) small chain [EC:1.4.1.13 1.4.1.14]	802	595	98.2	64.7	0.0406	1.35	NA	NA	NA
Aminoacid biosynthesis	Transcripts/gene	NA	gltD	glutamate synthase (NADPH/NADH) small chain [EC:1.4.1.13 1.4.1.14]	0.715	0.542	0.132	0.0644	0.125	1.32	NA	NA	NA
Aminoacid biosynthesis	DNA	K03340	dapdh	diaminopimelate dehydrogenase [EC:1.4.1.16]	172	184	16.8	11.1	0.62	1.07	NA	NA	NA
Aminoacid biosynthesis	RNA	K03340	dapdh	diaminopimelate dehydrogenase [EC:1.4.1.16]	231	206	86.3	60.1	0.623	1.12	NA	NA	NA
Aminoacid biosynthesis	Transcripts/gene	NA	dapdh	diaminopimelate dehydrogenase [EC:1.4.1.16]	1.37	1.11	0.553	0.254	0.234	1.23	NA	NA	NA
Aminoacid biosynthesis	DNA	K11646	NA	3-dehydroquinate synthase II [EC:1.4.1.24]	7.04	7.89	1.53	2.67	0.731	1.12	NA	NA	NA
Aminoacid biosynthesis	RNA	K11646	NA	3-dehydroquinate synthase II [EC:1.4.1.24]	1.16	1.56	0.389	0.771	0.623	1.35	NA	NA	NA
Aminoacid biosynthesis	Transcripts/gene	NA	NA	3-dehydroquinate synthase II [EC:1.4.1.24]	0.173	0.279	0.079	0.343	0.713	1.61	NA	NA	NA
Aminoacid biosynthesis	DNA	K03334	IL4I1	L-amino-acid oxidase [EC:1.4.3.2]	0.0159	0.0095	0.0137	0.0073	0.875	1.68	NA	NA	NA
Aminoacid biosynthesis	RNA	K00293	LYS9	saccharopine dehydrogenase (NADP+, L-glutamate forming) [EC:1.5.1.10]	1.1	0.867	0.474	0.621	0.471	1.27	NA	NA	NA
Aminoacid biosynthesis	DNA	K00286	proC	pyrroline-5-carboxylate reductase [EC:1.5.1.2]	158	153	19.3	10.7	1	1.03	NA	NA	NA
Aminoacid biosynthesis	RNA	K00286	proC	pyrroline-5-carboxylate reductase [EC:1.5.1.2]	85.5	57.6	13.4	7.68	0.0164	1.48	-0.0256	-0.0057	-0.0135
Aminoacid biosynthesis	Transcripts/gene	NA	proC	pyrroline-5-carboxylate reductase [EC:1.5.1.2]	0.547	0.378	0.102	0.0585	0.097	1.45	NA	NA	NA
Aminoacid biosynthesis	DNA	K00290	LYS1	saccharopine dehydrogenase (NAD+, L-lysine forming) [EC:1.5.1.7]	182	175	21	18.9	0.915	1.04	NA	NA	NA
Aminoacid biosynthesis	RNA	K00290	LYS1	saccharopine dehydrogenase (NAD+, L-lysine forming) [EC:1.5.1.7]	183	170	20.1	33.5	0.626	1.08	NA	NA	NA
Aminoacid biosynthesis	Transcripts/gene	NA	LYS1	saccharopine dehydrogenase (NAD+, L-lysine forming) [EC:1.5.1.7]	1.02	0.964	0.161	0.117	0.594	1.05	NA	NA	NA
Aminoacid biosynthesis	DNA	K00547	mmuM	homocysteine S-methyltransferase [EC:2.1.1.10]	37.8	17.8	11.7	6.24	0.0384	2.13	-0.00952	-0.00444	-0.00647
Aminoacid biosynthesis	RNA	K00547	mmuM	homocysteine S-methyltransferase [EC:2.1.1.10]	6.71	2.93	2.25	1	0.0126	2.29	-0.0202	-0.00373	-0.00917
Aminoacid biosynthesis	Transcripts/gene	NA	mmuM	homocysteine S-methyltransferase [EC:2.1.1.10]	0.178	0.176	0.0242	0.0563	0.983	1.01	NA	NA	NA
Aminoacid biosynthesis	DNA	K00548	metH	5-methyltetrahydrofolate--homocysteine methyltransferase [EC:2.1.1.13]	596	558	51.9	34.3	0.732	1.07	NA	NA	NA
Aminoacid biosynthesis	RNA	K00548	metH	5-methyltetrahydrofolate--homocysteine methyltransferase [EC:2.1.1.13]	333	252	58.4	29.3	0.0566	1.32	NA	NA	NA
Aminoacid biosynthesis	Transcripts/gene	NA	metH	5-methyltetrahydrofolate--homocysteine methyltransferase [EC:2.1.1.13]	0.566	0.452	0.13	0.0544	0.218	1.25	NA	NA	NA
Aminoacid biosynthesis	DNA	K00549	metE	5-methyltetrahydropteroyltriglutamate--homocysteine methyltransferase [EC:2.1.1.14]	76.6	42.7	26.3	9.64	0.00856	1.79	-0.0129	-0.00218	-0.00796
Aminoacid biosynthesis	RNA	K00549	metE	5-methyltetrahydropteroyltriglutamate--homocysteine methyltransferase [EC:2.1.1.14]	13.6	9.16	5.64	1.87	0.283	1.48	NA	NA	NA
Aminoacid biosynthesis	Transcripts/gene	NA	metE	5-methyltetrahydropteroyltriglutamate--homocysteine methyltransferase [EC:2.1.1.14]	0.183	0.222	0.0759	0.0518	0.234	1.21	NA	NA	NA
Aminoacid biosynthesis	DNA	K00558	dcm	DNA (cytosine-5)-methyltransferase 1 [EC:2.1.1.37]	525	552	60.8	46.5	0.911	1.05	NA	NA	NA
Aminoacid biosynthesis	RNA	K00558	dcm	DNA (cytosine-5)-methyltransferase 1 [EC:2.1.1.37]	93.9	111	16.4	9.73	0.13	1.18	NA	NA	NA
Aminoacid biosynthesis	Transcripts/gene	NA	dcm	DNA (cytosine-5)-methyltransferase 1 [EC:2.1.1.37]	0.183	0.201	0.0553	0.0127	0.209	1.1	NA	NA	NA
Aminoacid biosynthesis	DNA	K00544	BHMT	betaine-homocysteine S-methyltransferase [EC:2.1.1.5]	0.00584	0.0042	0.00827	0.0032	1	1.39	NA	NA	NA
Aminoacid biosynthesis	DNA	K00600	glyA	glycine hydroxymethyltransferase [EC:2.1.2.1]	304	318	24.7	19.7	0.875	1.05	NA	NA	NA
Aminoacid biosynthesis	RNA	K00600	glyA	glycine hydroxymethyltransferase [EC:2.1.2.1]	552	444	70.5	48.1	0.0698	1.24	NA	NA	NA
Aminoacid biosynthesis	Transcripts/gene	NA	glyA	glycine hydroxymethyltransferase [EC:2.1.2.1]	1.83	1.4	0.317	0.15	0.097	1.31	NA	NA	NA
Aminoacid biosynthesis	DNA	K00611	argF, argI	ornithine carbamoyltransferase [EC:2.1.3.3]	151	159	17	30.7	0.675	1.05	NA	NA	NA
Aminoacid biosynthesis	RNA	K00611	argF, argI	ornithine carbamoyltransferase [EC:2.1.3.3]	166	160	39	25.4	0.989	1.03	NA	NA	NA
Aminoacid biosynthesis	Transcripts/gene	NA	argF, argI	ornithine carbamoyltransferase [EC:2.1.3.3]	1.12	1.03	0.315	0.149	0.958	1.09	NA	NA	NA
Aminoacid biosynthesis	DNA	K00615	tktA, tktB	transketolase [EC:2.2.1.1]	695	682	66	68.6	0.939	1.02	NA	NA	NA
Aminoacid biosynthesis	RNA	K00615	tktA, tktB	transketolase [EC:2.2.1.1]	1430	1120	159	193	0.0658	1.28	NA	NA	NA
Aminoacid biosynthesis	Transcripts/gene	NA	tktA, tktB	transketolase [EC:2.2.1.1]	2.07	1.67	0.303	0.383	0.136	1.24	NA	NA	NA
Aminoacid biosynthesis	DNA	K00616	talA, talB	transaldolase [EC:2.2.1.2]	39.1	29.1	6.57	6.97	0.158	1.34	NA	NA	NA
Aminoacid biosynthesis	RNA	K00616	talA, talB	transaldolase [EC:2.2.1.2]	62	37.5	41.2	8.46	0.256	1.66	NA	NA	NA
Aminoacid biosynthesis	Transcripts/gene	NA	talA, talB	transaldolase / glucose-6-phosphate isomerase [EC:2.2.1.2 5.3.1.9]	1.61	1.31	1.12	0.3	0.958	1.23	NA	NA	NA
Aminoacid biosynthesis	DNA	K13810	tal-pgi	transaldolase / glucose-6-phosphate isomerase [EC:2.2.1.2 5.3.1.9]	0.248	0.415	0.0443	0.161	0.301	1.68	NA	NA	NA
Aminoacid biosynthesis	RNA	K13810	tal-pgi	transaldolase / glucose-6-phosphate isomerase [EC:2.2.1.2 5.3.1.9]	0.183	0.212	0.0718	0.154	0.895	1.16	NA	NA	NA
Aminoacid biosynthesis	DNA	K01652	ilvB, ilvG, ilvI	acetolactate synthase I/II/III large subunit [EC:2.2.1.6]	454	445	58.7	51.5	1	1.02	NA	NA	NA
Aminoacid biosynthesis	RNA	K01652	ilvB, ilvG, ilvI	acetolactate synthase I/II/III large subunit [EC:2.2.1.6]	394	304	53.6	35.6	0.0526	1.3	NA	NA	NA
Aminoacid biosynthesis	DNA	K01653	ilvH, ilvN	acetolactate synthase I/III small subunit [EC:2.2.1.6]	116	109	11.5	8.93	0.779	1.07	NA	NA	NA
Aminoacid biosynthesis	RNA	K01653	ilvH, ilvN	acetolactate synthase I/III small subunit [EC:2.2.1.6]	101	83.3	19.7	8.28	0.233	1.21	NA	NA	NA
Aminoacid biosynthesis	Transcripts/gene	NA	ilvH, ilvN	acetolactate synthase I/III small subunit [EC:2.2.1.6]	0.88	0.706	0.164	0.102	0.136	1.25	NA	NA	NA
Aminoacid biosynthesis	DNA	K00619	argA	amino-acid N-acetyltransferase [EC:2.3.1.1]	2.61	4.97	1.34	3.98	0.567	1.91	NA	NA	NA
Aminoacid biosynthesis	RNA	K00619	argA	amino-acid N-acetyltransferase [EC:2.3.1.1]	0.7	6.11	0.467	9.43	0.186	8.74	NA	NA	NA
Aminoacid biosynthesis	DNA	K14682	argAB	amino-acid N-acetyltransferase [EC:2.3.1.1]	15.4	10.6	7.42	3.48	0.564	1.46	NA	NA	NA
Aminoacid biosynthesis	RNA	K14682	argAB	amino-acid N-acetyltransferase [EC:2.3.1.1]	3.95	2.54	1.87	0.869	0.382	1.56	NA	NA	NA
Aminoacid biosynthesis	Transcripts/gene	NA	argAB	amino-acid N-acetyltransferase [EC:2.3.1.1]	0.473	0.435	0.114	0.0591	0.838	1.09	NA	NA	NA
Aminoacid biosynthesis	DNA	K00674	dapD	2,3,4,5-tetrahydropyridine-2-carboxylate N-succinyltransferase [EC:2.3.1.117]	24.3	15.8	4.42	3.36	0.0962	1.54	-0.0177	-0.0016	-0.0102
Aminoacid biosynthesis	RNA	K00674	dapD	2,3,4,5-tetrahydropyridine-2-carboxylate N-succinyltransferase [EC:2.3.1.117]	12.5	6.18	3.31	1.05	0.00669	2.02	-0.0271	-0.00751	-0.016
Aminoacid biosynthesis	Transcripts/gene	NA	dapD	2,3,4,5-tetrahydropyridine-2-carboxylate N-succinyltransferase [EC:2.3.1.117]	0.518	0.4	0.102	0.0671	0.136	1.29	NA	NA	NA
Aminoacid biosynthesis	DNA	K09011	cimA	D-citramalate synthase [EC:2.3.1.182]	127	130	13.5	18.4	1	1.02	NA	NA	NA
Aminoacid biosynthesis	RNA	K09011	cimA	D-citramalate synthase [EC:2.3.1.182]	123	113	37.1	50.6	0.895	1.08	NA	NA	NA
Aminoacid biosynthesis	Transcripts/gene	NA	cimA	D-citramalate synthase [EC:2.3.1.182]	0.984	0.846	0.339	0.296	0.636	1.16	NA	NA	NA
Aminoacid biosynthesis	DNA	K00640	cysE	serine O-acetyltransferase [EC:2.3.1.30]	232	211	13.3	13.4	0.176	1.1	NA	NA	NA
Aminoacid biosynthesis	RNA	K00640	cysE	serine O-acetyltransferase [EC:2.3.1.30]	117	85.3	25	15	0.119	1.37	NA	NA	NA
Aminoacid biosynthesis	Transcripts/gene	NA	cysE	serine O-acetyltransferase [EC:2.3.1.30]	0.506	0.403	0.119	0.0498	0.136	1.25	NA	NA	NA
Aminoacid biosynthesis	DNA	K00641	metX	homoserine O-acetyltransferase [EC:2.3.1.31]	33.5	38.5	8.22	7.16	0.675	1.15	NA	NA	NA
Aminoacid biosynthesis	RNA	K00641	metX	homoserine O-acetyltransferase [EC:2.3.1.31]	6.44	7.95	1.16	2.07	0.382	1.23	NA	NA	NA
Aminoacid biosynthesis	Transcripts/gene	NA	metX	homoserine O-acetyltransferase [EC:2.3.1.31]	0.201	0.214	0.0584	0.0765	0.942	1.06	NA	NA	NA
Aminoacid biosynthesis	Transcripts/gene	NA	argJ	glutamate N-acetyltransferase / amino-acid N-acetyltransferase [EC:2.3.1.35 2.3.1.1]	0.502	0.428	0.132	0.0386	0.491	1.17	NA	NA	NA
Aminoacid biosynthesis	DNA	K00620	argJ	glutamate N-acetyltransferase / amino-acid N-acetyltransferase [EC:2.3.1.35 2.3.1.1]	136	132	24	30.5	1	1.03	NA	NA	NA
Aminoacid biosynthesis	RNA	K00620	argJ	glutamate N-acetyltransferase / amino-acid N-acetyltransferase [EC:2.3.1.35 2.3.1.1]	67.1	56.3	15.1	13.3	0.519	1.19	NA	NA	NA
Aminoacid biosynthesis	DNA	K00651	metA	homoserine O-succinyltransferase [EC:2.3.1.46]	172	165	14.3	11.5	0.675	1.04	NA	NA	NA
Aminoacid biosynthesis	RNA	K00651	metA	homoserine O-succinyltransferase [EC:2.3.1.46]	126	117	17.7	13.4	0.519	1.08	NA	NA	NA
Aminoacid biosynthesis	Transcripts/gene	NA	metA	homoserine O-succinyltransferase [EC:2.3.1.46]	0.732	0.71	0.0864	0.0621	0.731	1.03	NA	NA	NA
Aminoacid biosynthesis	DNA	K05822	dapH, dapD	tetrahydrodipicolinate N-acetyltransferase [EC:2.3.1.89]	5.26	2.91	2.05	0.404	0.299	1.81	NA	NA	NA
Aminoacid biosynthesis	RNA	K05822	dapH, dapD	tetrahydrodipicolinate N-acetyltransferase [EC:2.3.1.89]	2.36	1.2	1.12	0.362	0.0536	1.96	NA	NA	NA
Aminoacid biosynthesis	Transcripts/gene	NA	dapH, dapD	tetrahydrodipicolinate N-acetyltransferase [EC:2.3.1.89]	0.467	0.412	0.14	0.105	0.594	1.13	NA	NA	NA
Aminoacid biosynthesis	DNA	K01647	gltA	citrate synthase [EC:2.3.3.1]	153	139	27.9	29.4	1	1.1	NA	NA	NA
Aminoacid biosynthesis	RNA	K01647	gltA	citrate synthase [EC:2.3.3.1]	99.6	83.4	11.1	24.3	0.283	1.19	NA	NA	NA
Aminoacid biosynthesis	Transcripts/gene	NA	gltA	citrate synthase [EC:2.3.3.1]	0.664	0.614	0.11	0.167	0.594	1.08	NA	NA	NA
Aminoacid biosynthesis	DNA	K01649	leuA	2-isopropylmalate synthase [EC:2.3.3.13]	329	326	38.2	34.1	1	1.01	NA	NA	NA
Aminoacid biosynthesis	RNA	K01649	leuA	2-isopropylmalate synthase [EC:2.3.3.13]	304	249	58	48.9	0.382	1.22	NA	NA	NA

Aminoacid biosynthesis	Transcripts/gene	NA	leuA	2-isopropylmalate synthase [EC:2.3.3.13]	0.933	0.779	0.209	0.206	0.31	1.2	NA	NA	NA
Aminoacid biosynthesis	DNA	K01655	LYS20, LYS21	homocitrate synthase [EC:2.3.3.14]	5.92	8.01	2.44	1.71	0.504	1.35	NA	NA	NA
Aminoacid biosynthesis	RNA	K01655	LYS20, LYS21	homocitrate synthase [EC:2.3.3.14]	1.06	1.13	0.27	0.244	0.788	1.06	NA	NA	NA
Aminoacid biosynthesis	DNA	K10977	aksA	methanogen homocitrate synthase [EC:2.3.3.14 2.3.3.-]	6.15	7.03	1.46	2.68	0.83	1.14	NA	NA	NA
Aminoacid biosynthesis	RNA	K10977	aksA	methanogen homocitrate synthase [EC:2.3.3.14 2.3.3.-]	0.28	0.884	0.151	0.593	0.121	3.16	NA	NA	NA
Aminoacid biosynthesis	Transcripts/gene	NA	aksA	methanogen homocitrate synthase [EC:2.3.3.14 2.3.3.-]	0.112	0.155	0.0191	0.105	0.636	1.38	NA	NA	NA
Aminoacid biosynthesis	DNA	K00765	hisG	ATP phosphoribosyltransferase [EC:2.4.2.17]	153	150	14.5	7.32	0.989	1.02	NA	NA	NA
Aminoacid biosynthesis	RNA	K00765	hisG	ATP phosphoribosyltransferase [EC:2.4.2.17]	110	93	17.9	7.41	0.208	1.18	NA	NA	NA
Aminoacid biosynthesis	Transcripts/gene	NA	hisG	ATP phosphoribosyltransferase [EC:2.4.2.17]	0.726	0.621	0.151	0.0557	0.31	1.17	NA	NA	NA
Aminoacid biosynthesis	DNA	K00766	trpD	anthranilate phosphoribosyltransferase [EC:2.4.2.18]	168	159	18.1	9.63	0.911	1.05	NA	NA	NA
Aminoacid biosynthesis	RNA	K00766	trpD	anthranilate phosphoribosyltransferase [EC:2.4.2.18]	138	122	44.8	50.7	0.73	1.13	NA	NA	NA
Aminoacid biosynthesis	Transcripts/gene	NA	trpD	anthranilate synthase/phosphoribosyltransferase [EC:4.1.3.27 2.4.2.18]	0.845	0.771	0.299	0.262	0.671	1.1	NA	NA	NA
Aminoacid biosynthesis	DNA	K00772	mtaP	5'-methylthioadenosine phosphorylase [EC:2.4.2.28]	13.7	15.5	3.87	6.71	1	1.13	NA	NA	NA
Aminoacid biosynthesis	RNA	K00772	mtaP	5'-methylthioadenosine phosphorylase [EC:2.4.2.28]	3.07	7.64	0.836	5.98	0.0406	2.49	NA	NA	NA
Aminoacid biosynthesis	Transcripts/gene	NA	mtaP	5'-methylthioadenosine phosphorylase [EC:2.4.2.28]	0.232	0.498	0.0695	0.375	0.16	2.15	NA	NA	NA
Aminoacid biosynthesis	DNA	K00797	speE	spermidine synthase [EC:2.5.1.16]	40.1	36.5	10.1	11	0.83	1.1	NA	NA	NA
Aminoacid biosynthesis	RNA	K00797	speE	spermidine synthase [EC:2.5.1.16]	17.7	21	6.06	5.92	0.572	1.18	NA	NA	NA
Aminoacid biosynthesis	Transcripts/gene	NA	speE	spermidine synthase [EC:2.5.1.16]	0.458	0.589	0.174	0.141	0.315	1.29	NA	NA	NA
Aminoacid biosynthesis	DNA	K00800	aroA	3-phosphoshikimate 1-carboxyvinyltransferase [EC:2.5.1.19]	261	259	29.4	16.6	1	1.01	NA	NA	NA
Aminoacid biosynthesis	RNA	K00800	aroA	3-phosphoshikimate 1-carboxyvinyltransferase [EC:2.5.1.19]	132	95.4	19.9	12.7	0.0394	1.38	NA	NA	NA
Aminoacid biosynthesis	Transcripts/gene	NA	aroA	pentafunctional AROM polypeptide [EC:4.2.3.4 4.2.1.10 1.1.1.25 2.7.1.71 2.5.1.19]	0.514	0.37	0.112	0.0515	0.125	1.39	NA	NA	NA
Aminoacid biosynthesis	DNA	K00802	SMS	spermine synthase [EC:2.5.1.22]	0.00398	0.0023	0.00384	0.0025	1	1.72	NA	NA	NA
Aminoacid biosynthesis	RNA	K00802	SMS	spermine synthase [EC:2.5.1.22]	0.0153	0.0666	0.0331	0.0483	0.228	4.34	NA	NA	NA
Aminoacid biosynthesis	Transcripts/gene	NA	SMS	spermine synthase [EC:2.5.1.22]	2.03	3.91	3.85	7.25	0.942	1.93	NA	NA	NA
Aminoacid biosynthesis	DNA	K01738	cysK	cysteine synthase A [EC:2.5.1.47]	346	358	48.2	39.5	0.989	1.04	NA	NA	NA
Aminoacid biosynthesis	RNA	K01738	cysK	cysteine synthase A [EC:2.5.1.47]	525	157	766	19.7	0.344	3.35	NA	NA	NA
Aminoacid biosynthesis	DNA	K12339	cysM	cysteine synthase B [EC:2.5.1.47]	1.68	1.78	0.65	0.566	0.989	1.06	NA	NA	NA
Aminoacid biosynthesis	RNA	K12339	cysM	cysteine synthase B [EC:2.5.1.47]	0.804	0.294	0.585	0.158	0.14	2.73	NA	NA	NA
Aminoacid biosynthesis	Transcripts/gene	NA	cysM	cysteine synthase B [EC:2.5.1.47]	1.64	0.439	2.64	0.0588	0.234	3.74	NA	NA	NA
Aminoacid biosynthesis	DNA	K13034	ATCYSC1	L-3-cyanoalanine synthase/ cysteine synthase [EC:2.5.1.47 4.4.1.9]	0.00811	0.0234	0.0119	0.0188	0.45	2.89	NA	NA	NA
Aminoacid biosynthesis	DNA	K01739	metB	cystathionine gamma-synthase [EC:2.5.1.48]	13	13.2	2.71	2.12	1	1.01	NA	NA	NA
Aminoacid biosynthesis	RNA	K01739	metB	cystathionine gamma-synthase [EC:2.5.1.48]	22.2	9.93	10.9	3.38	0.0252	2.23	NA	NA	NA
Aminoacid biosynthesis	Transcripts/gene	NA	metB	cystathionine gamma-synthase [EC:2.5.1.48]	1.77	0.767	1.04	0.275	0.097	2.31	NA	NA	NA
Aminoacid biosynthesis	DNA	K01740	metY	O-acetylhomoserine (thiol)-lyase [EC:2.5.1.49]	582	580	72	39.8	1	1	NA	NA	NA
Aminoacid biosynthesis	RNA	K01740	metY	O-acetylhomoserine (thiol)-lyase [EC:2.5.1.49]	609	241	789	31.3	0.253	2.53	NA	NA	NA
Aminoacid biosynthesis	Transcripts/gene	NA	metY	O-acetylhomoserine (thiol)-lyase [EC:2.5.1.49]	1.12	0.415	1.59	0.037	0.263	2.7	NA	NA	NA
Aminoacid biosynthesis	DNA	K01626	aroF, aroG, aroH	3-deoxy-7-phosphoheptulonate synthase [EC:2.5.1.54]	89.6	75.6	12.7	17.5	0.62	1.18	NA	NA	NA
Aminoacid biosynthesis	RNA	K01626	aroF, aroG, aroH	3-deoxy-7-phosphoheptulonate synthase [EC:2.5.1.54]	70.4	44.1	9.22	5.12	0.00669	1.6	-0.0275	-0.0135	-0.0202
Aminoacid biosynthesis	DNA	K03856	aroA	3-deoxy-7-phosphoheptulonate synthase [EC:2.5.1.54]	53.7	68.6	11.1	20.5	0.676	1.28	NA	NA	NA
Aminoacid biosynthesis	RNA	K03856	aroA	3-deoxy-7-phosphoheptulonate synthase [EC:2.5.1.54]	50.7	38.6	17.2	10.1	0.253	1.31	NA	NA	NA
Aminoacid biosynthesis	Transcripts/gene	NA	aroA	3-deoxy-7-phosphoheptulonate synthase / chorismate mutase [EC:2.5.1.54 5.4.99.5]	0.853	0.598	0.145	0.134	0.097	1.43	NA	NA	NA
Aminoacid biosynthesis	DNA	K13853	aroG, aroA	3-deoxy-7-phosphoheptulonate synthase / chorismate mutase [EC:2.5.1.54 5.4.99.5]	0.135	0.381	0.036	0.39	0.033	2.82	NA	NA	NA
Aminoacid biosynthesis	RNA	K13853	aroG, aroA	3-deoxy-7-phosphoheptulonate synthase / chorismate mutase [EC:2.5.1.54 5.4.99.5]	0.0766	0.443	0.0546	0.58	0.185	5.79	NA	NA	NA
Aminoacid biosynthesis	DNA	K00789	metK	S-adenosylmethionine synthetase [EC:2.5.1.6]	376	379	25.4	16.6	1	1.01	NA	NA	NA
Aminoacid biosynthesis	RNA	K00789	metK	S-adenosylmethionine synthetase [EC:2.5.1.6]	334	324	35	31.2	0.676	1.03	NA	NA	NA
Aminoacid biosynthesis	Transcripts/gene	NA	metK	S-adenosylmethionine synthetase [EC:2.5.1.6]	0.89	0.854	0.0871	0.0858	0.636	1.04	NA	NA	NA
Aminoacid biosynthesis	DNA	K00811	ASP5	aspartate aminotransferase, chloroplastic [EC:2.6.1.1]	160	157	33.4	15.5	0.989	1.02	NA	NA	NA
Aminoacid biosynthesis	RNA	K00811	ASP5	aspartate aminotransferase, chloroplastic [EC:2.6.1.1]	171	146	28	21	0.256	1.17	NA	NA	NA
Aminoacid biosynthesis	DNA	K00812	aspB	aspartate aminotransferase [EC:2.6.1.1]	186	183	16.8	9.68	1	1.02	NA	NA	NA
Aminoacid biosynthesis	RNA	K00812	aspB	aspartate aminotransferase [EC:2.6.1.1]	232	189	52.2	40.7	0.312	1.22	NA	NA	NA
Aminoacid biosynthesis	DNA	K00813	aspC	aspartate aminotransferase [EC:2.6.1.1]	8.41	9.27	2.49	3.42	1	1.1	NA	NA	NA
Aminoacid biosynthesis	RNA	K00813	aspC	aspartate aminotransferase [EC:2.6.1.1]	2.26	2.11	0.602	0.588	0.944	1.07	NA	NA	NA
Aminoacid biosynthesis	DNA	K11358	yhdR	aspartate aminotransferase [EC:2.6.1.1]	103	120	5.57	24	0.213	1.17	NA	NA	NA
Aminoacid biosynthesis	RNA	K11358	yhdR	aspartate aminotransferase [EC:2.6.1.1]	59.9	67.3	10.3	14.8	0.427	1.12	NA	NA	NA
Aminoacid biosynthesis	DNA	K14454	GOT1	aspartate aminotransferase, cytoplasmic [EC:2.6.1.1]	1.28	1.12	1.03	0.364	1	1.14	NA	NA	NA
Aminoacid biosynthesis	RNA	K14454	GOT1	aspartate aminotransferase, cytoplasmic [EC:2.6.1.1]	0.928	1.26	0.499	0.188	0.312	1.35	NA	NA	NA
Aminoacid biosynthesis	DNA	K14455	GOT2	aspartate aminotransferase, mitochondrial [EC:2.6.1.1]	4.86	3.9	3.4	1.3	1	1.25	NA	NA	NA
Aminoacid biosynthesis	RNA	K14455	GOT2	aspartate aminotransferase, mitochondrial [EC:2.6.1.1]	2.89	3.32	1.9	0.635	0.472	1.15	NA	NA	NA
Aminoacid biosynthesis	Transcripts/gene	NA	GOT2	aspartate aminotransferase, mitochondrial [EC:2.6.1.1]	1.02	0.864	0.206	0.101	0.31	1.18	NA	NA	NA
Aminoacid biosynthesis	DNA	K00818	argD	acetylornithine aminotransferase [EC:2.6.1.11]	133	131	17.1	15.9	1	1.02	NA	NA	NA
Aminoacid biosynthesis	RNA	K00818	argD	acetylornithine aminotransferase [EC:2.6.1.11]	131	102	35.3	19.4	0.228	1.29	NA	NA	NA
Aminoacid biosynthesis	DNA	K05830	lysJ	acetylornithine/LysW-gamma-L-lysine aminotransferase [EC:2.6.1.11 2.6.1.-]	0.0647	0.129	0.0175	0.039	0.00856	2	0.00529	0.0199	0.0134
Aminoacid biosynthesis	RNA	K05830	lysJ	acetylornithine/LysW-gamma-L-lysine aminotransferase [EC:2.6.1.11 2.6.1.-]	0.0348	0.0374	0.0632	0.0615	0.944	1.07	NA	NA	NA
Aminoacid biosynthesis	Transcripts/gene	NA	lysJ	acetylornithine/LysW-gamma-L-lysine aminotransferase [EC:2.6.1.11 2.6.1.-]	0.888	0.801	0.199	0.082	0.671	1.11	NA	NA	NA
Aminoacid biosynthesis	DNA	K00821	argD	acetylornithine/N-succinyldiaminopimelate aminotransferase [EC:2.6.1.11 2.6.1.17]	122	113	20.4	26.9	1	1.08	NA	NA	NA
Aminoacid biosynthesis	RNA	K00821	argD	acetylornithine/N-succinyldiaminopimelate aminotransferase [EC:2.6.1.11 2.6.1.17]	91.5	92.7	21.6	18.1	0.895	1.01	NA	NA	NA
Aminoacid biosynthesis	DNA	K14267	dapC	N-succinyldiaminopimelate aminotransferase [EC:2.6.1.17]	1.02	0.782	0.249	0.162	0.348	1.3	NA	NA	NA
Aminoacid biosynthesis	RNA	K14267	dapC	N-succinyldiaminopimelate aminotransferase [EC:2.6.1.17]	0.485	0.268	0.171	0.102	0.0788	1.81	NA	NA	NA
Aminoacid biosynthesis	Transcripts/gene	NA	dapC	N-succinyldiaminopimelate aminotransferase [EC:2.6.1.17]	0.779	0.843	0.283	0.174	0.554	1.08	NA	NA	NA
Aminoacid biosynthesis	DNA	K00814	GPT	alanine transaminase [EC:2.6.1.2]	5.34	4.21	4.68	0.956	0.989	1.27	NA	NA	NA
Aminoacid biosynthesis	RNA	K00814	GPT	alanine transaminase [EC:2.6.1.2]	2.15	4.77	0.772	1.25	0.0252	2.21	0.0153	0.0333	0.0287
Aminoacid biosynthesis	Transcripts/gene	NA	alaA	alanine-synthesizing transaminase [EC:2.6.1.66 2.6.1.2]	0.81	0.89	0.277	0.275	0.636	1.1	NA	NA	NA
Aminoacid biosynthesis	Transcripts/gene	NA	ARO8	aromatic amino acid aminotransferase I [EC:2.6.1.57 2.6.1.27 2.6.1.5]	3.17	1.03	2.24	0.555	0.125	3.08	NA	NA	NA
Aminoacid biosynthesis	Transcripts/gene	NA	ARO9	aromatic amino acid aminotransferase II [EC:2.6.1.58 2.6.1.28]	57.2	10.8	45.3	18.9	0.0769	5.28	NA	NA	NA
Aminoacid biosynthesis	Transcripts/gene	NA	AADAT	kynurenine/2-aminoadipate aminotransferase [EC:2.6.1.7 2.6.1.39]	1.34	1.2	1.06	0.608	0.895	1.12	NA	NA	NA
Aminoacid biosynthesis	DNA	K00826	ilvE	branched-chain amino acid aminotransferase [EC:2.6.1.42]	259	256	21.4	14.8	1	1.01	NA	NA	NA
Aminoacid biosynthesis	RNA	K00826	ilvE	branched-chain amino acid aminotransferase [EC:2.6.1.42]	522	460	125	35.9	0.382	1.13	NA	NA	NA
Aminoacid biosynthesis	Transcripts/gene	NA	ilvE	branched-chain amino acid aminotransferase [EC:2.6.1.42]	2.02	1.8	0.501	0.108	0.234	1.12	NA	NA	NA
Aminoacid biosynthesis	DNA	K00815	TAT	tyrosine aminotransferase [EC:2.6.1.5]	0.109	0.101	0.0593	0.0372	1	1.07	NA	NA	NA
Aminoacid biosynthesis	RNA	K00815	TAT	tyrosine aminotransferase [EC:2.6.1.5]	0.0652	0.0577	0.0733	0.0592	0.944	1.13	NA	NA	NA
Aminoacid biosynthesis	Transcripts/gene	NA	ARO8	aromatic amino acid aminotransferase I [EC:2.6.1.57 2.6.1.27 2.6.1.5]	1.35	0.769	0.855	0.739	0.354	1.76	NA	NA	NA
Aminoacid biosynthesis	DNA	K00831	serC	phosphoserine aminotransferase [EC:2.6.1.52]	258	260	24.5	14.6	1	1.01	NA	NA	NA
Aminoacid biosynthesis	RNA	K00831	serC	phosphoserine aminotransferase [EC:2.6.1.52]	632	577	123	55.2	0.312	1.1	NA	NA	NA
Aminoacid biosynthesis	Transcripts/gene	NA	serC	phosphoserine aminotransferase [EC:2.6.1.52]	2.47	2.22	0.509	0.176	0.234	1.11	NA	NA	NA

Aminoacid biosynthesis	DNA	K00832	tyrB	aromatic-amino-acid transaminase [EC:2.6.1.57]	21.3	19.4	8.07	11	0.968	1.09	NA	NA	NA
Aminoacid biosynthesis	RNA	K00832	tyrB	aromatic-amino-acid transaminase [EC:2.6.1.57]	11.3	19.5	5.46	26.9	0.424	1.72	NA	NA	NA
Aminoacid biosynthesis	Transcripts/gene	NA	tyrB	aromatic amino acid aminotransferase I [EC:2.6.1.57 2.6.1.27 2.6.1.5]	0.548	0.816	0.178	0.805	0.671	1.49	NA	NA	NA
Aminoacid biosynthesis	DNA	K00838	ARO8	aromatic amino acid aminotransferase I [EC:2.6.1.57 2.6.1.27 2.6.1.5]	0.0432	0.0398	0.018	0.0214	1	1.09	NA	NA	NA
Aminoacid biosynthesis	RNA	K00838	ARO8	aromatic amino acid aminotransferase I [EC:2.6.1.57 2.6.1.27 2.6.1.5]	0.126	0.0459	0.116	0.0325	0.142	2.76	NA	NA	NA
Aminoacid biosynthesis	DNA	K05821	ARO9	aromatic amino acid aminotransferase II [EC:2.6.1.58 2.6.1.28]	0.00686	0.003	0.00566	0.0052	0.563	2.29	NA	NA	NA
Aminoacid biosynthesis	RNA	K05821	ARO9	aromatic amino acid aminotransferase II [EC:2.6.1.58 2.6.1.28]	0.39	0.194	0.352	0.0979	0.311	2.02	NA	NA	NA
Aminoacid biosynthesis	Transcripts/gene	NA	alaA	alanine-synthesizing transaminase [EC:2.6.1.66 2.6.1.2]	0.873	0.859	0.291	0.357	0.895	1.02	NA	NA	NA
Aminoacid biosynthesis	DNA	K14260	alaA	alanine-synthesizing transaminase [EC:2.6.1.66 2.6.1.2]	33.2	28.4	7.69	8.82	0.779	1.17	NA	NA	NA
Aminoacid biosynthesis	RNA	K14260	alaA	alanine-synthesizing transaminase [EC:2.6.1.66 2.6.1.2]	28.3	22.9	9.13	7.18	0.572	1.24	NA	NA	NA
Aminoacid biosynthesis	Transcripts/gene	NA	AADAT	kynurenine/2-aminoadipate aminotransferase [EC:2.6.1.7 2.6.1.39]	0.91	0.842	0.513	0.344	0.958	1.08	NA	NA	NA
Aminoacid biosynthesis	DNA	K00825	AADAT	kynurenine/2-aminoadipate aminotransferase [EC:2.6.1.7 2.6.1.39]	0.168	0.184	0.0784	0.0572	0.911	1.09	NA	NA	NA
Aminoacid biosynthesis	RNA	K00825	AADAT	kynurenine/2-aminoadipate aminotransferase [EC:2.6.1.7 2.6.1.39]	0.191	0.219	0.106	0.14	0.944	1.14	NA	NA	NA
Aminoacid biosynthesis	DNA	K00836	ectB	diaminobutyrate-2-oxoglutarate transaminase [EC:2.6.1.76]	11.1	10.6	8.94	2	0.394	1.05	NA	NA	NA
Aminoacid biosynthesis	RNA	K00836	ectB	diaminobutyrate-2-oxoglutarate transaminase [EC:2.6.1.76]	10.1	7.08	10.7	1.64	0.895	1.43	NA	NA	NA
Aminoacid biosynthesis	Transcripts/gene	NA	ectB	diaminobutyrate-2-oxoglutarate transaminase [EC:2.6.1.76]	0.85	0.679	0.229	0.144	0.426	1.25	NA	NA	NA
Aminoacid biosynthesis	DNA	K10206		LL-diaminopimelate aminotransferase [EC:2.6.1.83]	242	238	23	21.2	1	1.02	NA	NA	NA
Aminoacid biosynthesis	RNA	K10206		LL-diaminopimelate aminotransferase [EC:2.6.1.83]	67.7	69.7	17.8	43.6	0.424	1.03	NA	NA	NA
Aminoacid biosynthesis	Transcripts/gene	NA	NA	LL-diaminopimelate aminotransferase [EC:2.6.1.83]	0.279	0.286	0.0588	0.161	0.426	1.02	NA	NA	NA
Aminoacid biosynthesis	DNA	K00817	hisC	histidinol-phosphate aminotransferase [EC:2.6.1.9]	296	291	46.5	18.1	0.968	1.02	NA	NA	NA
Aminoacid biosynthesis	RNA	K00817	hisC	histidinol-phosphate aminotransferase [EC:2.6.1.9]	167	132	27.2	20	0.144	1.26	NA	NA	NA
Aminoacid biosynthesis	Transcripts/gene	NA	hisC	histidinol-phosphate aminotransferase [EC:2.6.1.9]	0.578	0.452	0.147	0.0523	0.155	1.28	NA	NA	NA
Aminoacid biosynthesis	DNA	K00899	mtnK	5-methylthioribose kinase [EC:2.7.1.100]	11.2	7.71	3.08	3.24	0.399	1.45	NA	NA	NA
Aminoacid biosynthesis	RNA	K00899	mtnK	5-methylthioribose kinase [EC:2.7.1.100]	4.52	4.37	0.942	1.65	0.895	1.04	NA	NA	NA
Aminoacid biosynthesis	Transcripts/gene	NA	mtnK	5-methylthioribose kinase [EC:2.7.1.100]	0.426	0.625	0.129	0.25	0.382	1.47	NA	NA	NA
Aminoacid biosynthesis	DNA	K00850	pfkA	6-phosphofructokinase 1 [EC:2.7.1.11]	453	429	49.9	55.8	0.83	1.06	NA	NA	NA
Aminoacid biosynthesis	RNA	K00850	pfkA	6-phosphofructokinase 1 [EC:2.7.1.11]	807	861	194	263	0.97	1.07	NA	NA	NA
Aminoacid biosynthesis	Transcripts/gene	NA	pfkA	6-phosphofructokinase 1 [EC:2.7.1.11]	1.81	1.99	0.515	0.458	0.636	1.1	NA	NA	NA
Aminoacid biosynthesis	DNA	K00872	thrB1	homoserine kinase [EC:2.7.1.39]	35.6	22	11.7	9.4	0.267	1.62	NA	NA	NA
Aminoacid biosynthesis	RNA	K00872	thrB1	homoserine kinase [EC:2.7.1.39]	15.2	9.51	5.77	6.38	0.283	1.6	NA	NA	NA
Aminoacid biosynthesis	DNA	K02204	thrB2	homoserine kinase type II [EC:2.7.1.39]	0.279	0.449	0.0863	0.174	0.395	1.61	NA	NA	NA
Aminoacid biosynthesis	RNA	K02204	thrB2	homoserine kinase type II [EC:2.7.1.39]	0.0342	0.0874	0.0465	0.0861	0.355	2.56	NA	NA	NA
Aminoacid biosynthesis	Transcripts/gene	NA	thrB2	homoserine kinase type II [EC:2.7.1.39]	0.748	0.646	0.166	0.116	0.31	1.16	NA	NA	NA
Aminoacid biosynthesis	DNA	K00873	pyk	pyruvate kinase [EC:2.7.1.40]	214	208	31.7	31.1	1	1.03	NA	NA	NA
Aminoacid biosynthesis	RNA	K00873	pyk	pyruvate kinase [EC:2.7.1.40]	234	170	69.4	42.9	0.139	1.38	NA	NA	NA
Aminoacid biosynthesis	DNA	K12406	PKLR	pyruvate kinase isozymes R/L [EC:2.7.1.40]	0.0851	0.0891	0.0491	0.0503	1	1.05	NA	NA	NA
Aminoacid biosynthesis	Transcripts/gene	NA	pyk	pyruvate kinase [EC:2.7.1.40]	1.09	0.813	0.203	0.141	0.125	1.33	NA	NA	NA
Aminoacid biosynthesis	DNA	K00891	aroK, aroL	shikimate kinase [EC:2.7.1.71]	136	129	11	6.58	0.83	1.05	NA	NA	NA
Aminoacid biosynthesis	RNA	K00891	aroK, aroL	shikimate kinase [EC:2.7.1.71]	40.5	31.3	7.68	4.65	0.0998	1.3	NA	NA	NA
Aminoacid biosynthesis	Transcripts/gene	NA	aroK, aroL	pentafunctional AROM polypeptide [EC:4.2.3.4 4.2.1.10 1.1.1.25 2.7.1.71 2.5.1.19]	0.307	0.248	0.0606	0.0411	0.155	1.23	NA	NA	NA
Aminoacid biosynthesis	DNA	K13829	aroKB	shikimate kinase / 3-dehydroquinate synthase [EC:2.7.1.71 4.2.3.4]	12	9.94	6.76	1.72	1	1.21	NA	NA	NA
Aminoacid biosynthesis	RNA	K13829	aroKB	shikimate kinase / 3-dehydroquinate synthase [EC:2.7.1.71 4.2.3.4]	4.18	3.04	1.7	1.09	0.471	1.38	NA	NA	NA
Aminoacid biosynthesis	DNA	K00931	proB	glutamate 5-kinase [EC:2.7.2.11]	161	154	21.5	14	1	1.05	NA	NA	NA
Aminoacid biosynthesis	RNA	K00931	proB	glutamate 5-kinase [EC:2.7.2.11]	109	83.8	18.1	18.6	0.081	1.3	NA	NA	NA
Aminoacid biosynthesis	Transcripts/gene	NA	proB	delta-1-pyrroline-5-carboxylate synthetase [EC:2.7.2.11 1.2.1.41]	0.693	0.547	0.143	0.0955	0.177	1.27	NA	NA	NA
Aminoacid biosynthesis	DNA	K12657	ALDH18A1	delta-1-pyrroline-5-carboxylate synthetase [EC:2.7.2.11 1.2.1.41]	0.164	0.182	0.0799	0.0385	0.62	1.11	NA	NA	NA
Aminoacid biosynthesis	RNA	K12657	ALDH18A1	delta-1-pyrroline-5-carboxylate synthetase [EC:2.7.2.11 1.2.1.41]	0.597	0.413	0.309	0.16	0.344	1.44	NA	NA	NA
Aminoacid biosynthesis	DNA	K00927	pgk	phosphoglycerate kinase [EC:2.7.2.3]	349	359	33.1	22.4	0.989	1.03	NA	NA	NA
Aminoacid biosynthesis	RNA	K00927	pgk	phosphoglycerate kinase [EC:2.7.2.3]	1670	1470	277	171	0.256	1.14	NA	NA	NA
Aminoacid biosynthesis	Transcripts/gene	NA	pgk	phosphoglycerate kinase [EC:2.7.2.3]	4.82	4.1	0.86	0.509	0.177	1.18	NA	NA	NA
Aminoacid biosynthesis	DNA	K00928	lysC	aspartate kinase [EC:2.7.2.4]	333	321	38.2	22.1	1	1.04	NA	NA	NA
Aminoacid biosynthesis	RNA	K00928	lysC	aspartate kinase [EC:2.7.2.4]	282	236	41.9	25.9	0.188	1.2	NA	NA	NA
Aminoacid biosynthesis	Transcripts/gene	NA	lysC	bifunctional diaminopimelate decarboxylase / aspartate kinase [EC:4.1.1.20 2.7.2.4]	1.02	0.865	0.169	0.0832	0.136	1.18	NA	NA	NA
Aminoacid biosynthesis	DNA	K12524	thrA	bifunctional aspartokinase / homoserine dehydrogenase 1 [EC:2.7.2.4 1.1.1.3]	225	229	26.4	32.3	1	1.02	NA	NA	NA
Aminoacid biosynthesis	RNA	K12524	thrA	bifunctional aspartokinase / homoserine dehydrogenase 1 [EC:2.7.2.4 1.1.1.3]	284	240	47.7	41.4	0.283	1.18	NA	NA	NA
Aminoacid biosynthesis	DNA	K12525	metL	bifunctional aspartokinase / homoserine dehydrogenase 2 [EC:2.7.2.4 1.1.1.3]	0.538	0.381	0.586	0.15	1	1.41	NA	NA	NA
Aminoacid biosynthesis	RNA	K12525	metL	bifunctional aspartokinase / homoserine dehydrogenase 2 [EC:2.7.2.4 1.1.1.3]	0.1	0.0481	0.108	0.0458	0.681	2.08	NA	NA	NA
Aminoacid biosynthesis	DNA	K00930	argB	acetylglutamate kinase [EC:2.7.2.8]	183	177	22.1	13.2	1	1.03	NA	NA	NA
Aminoacid biosynthesis	RNA	K00930	argB	acetylglutamate kinase [EC:2.7.2.8]	90.9	70.6	15.5	12.2	0.189	1.29	NA	NA	NA
Aminoacid biosynthesis	Transcripts/gene	NA	argB	N-acetyl-gamma-glutamyl-phosphate reductase / acetylglutamate kinase [EC:1.2.1.38 2.7.2.8]	0.505	0.4	0.113	0.062	0.136	1.26	NA	NA	NA
Aminoacid biosynthesis	DNA	K00948	prsA	ribose-phosphate pyrophosphokinase [EC:2.7.6.1]	286	292	25.3	24.4	0.938	1.02	NA	NA	NA
Aminoacid biosynthesis	RNA	K00948	prsA	ribose-phosphate pyrophosphokinase [EC:2.7.6.1]	417	368	65.8	15.2	0.188	1.13	NA	NA	NA
Aminoacid biosynthesis	Transcripts/gene	NA	prsA	ribose-phosphate pyrophosphokinase [EC:2.7.6.1]	1.46	1.27	0.249	0.104	0.177	1.15	NA	NA	NA
Aminoacid biosynthesis	Transcripts/gene	NA	sseA	thiosulfate/3-mercaptopyruvate sulfurtransferase [EC:2.8.1.1 2.8.1.2]	0.0986	0.136	0.108	0.0951	0.525	1.38	NA	NA	NA
Aminoacid biosynthesis	DNA	K01011	sseA	thiosulfate/3-mercaptopyruvate sulfurtransferase [EC:2.8.1.1 2.8.1.2]	3.3	2.6	0.803	0.43	0.563	1.27	NA	NA	NA
Aminoacid biosynthesis	RNA	K01011	sseA	thiosulfate/3-mercaptopyruvate sulfurtransferase [EC:2.8.1.1 2.8.1.2]	0.282	0.348	0.273	0.227	0.519	1.24	NA	NA	NA
Aminoacid biosynthesis	Transcripts/gene	NA	sseA	thiosulfate/3-mercaptopyruvate sulfurtransferase [EC:2.8.1.1 2.8.1.2]	0.101	0.14	0.111	0.0981	0.525	1.39	NA	NA	NA
Aminoacid biosynthesis	Transcripts/gene	NA	NA	fructose 1,6-bisphosphate aldolase/phosphatase [EC:4.1.2.13 3.1.3.11]	0.736	0.498	0.125	0.0858	0.097	1.48	NA	NA	NA
Aminoacid biosynthesis	DNA	K04486	NA	histidinol-phosphatase (PHP family) [EC:3.1.3.15]	118	103	18.9	27	0.875	1.14	NA	NA	NA
Aminoacid biosynthesis	RNA	K04486	NA	histidinol-phosphatase (PHP family) [EC:3.1.3.15]	26.7	20.9	5.93	6.49	0.256	1.27	NA	NA	NA
Aminoacid biosynthesis	Transcripts/gene	NA	NA	histidinol-phosphatase (PHP family) [EC:3.1.3.15]	0.449	0.392	0.0715	0.058	0.274	1.15	NA	NA	NA
Aminoacid biosynthesis	DNA	K01079	serB	phosphoserine phosphatase [EC:3.1.3.3]	131	118	19	18.8	0.731	1.11	NA	NA	NA
Aminoacid biosynthesis	RNA	K01079	serB	phosphoserine phosphatase [EC:3.1.3.3]	86.5	74.9	14.7	25.7	0.519	1.15	NA	NA	NA
Aminoacid biosynthesis	Transcripts/gene	NA	serB	phosphoserine / homoserine phosphotransferase [EC:3.1.3.3 2.7.1.39]	0.751	0.656	0.163	0.126	0.354	1.14	NA	NA	NA
Aminoacid biosynthesis	DNA	K02203	thrH	phosphoserine / homoserine phosphotransferase [EC:3.1.3.3 2.7.1.39]	47.8	57.2	6.66	12.7	0.293	1.2	NA	NA	NA
Aminoacid biosynthesis	RNA	K02203	thrH	phosphoserine / homoserine phosphotransferase [EC:3.1.3.3 2.7.1.39]	45.9	40.2	11.8	7.2	0.519	1.14	NA	NA	NA
Aminoacid biosynthesis	DNA	K09880	mtnC	enolase-phosphatase E1 [EC:3.1.3.77]	0.0287	0.0395	0.0188	0.0193	0.779	1.37	NA	NA	NA
Aminoacid biosynthesis	DNA	K08966	mtnX	2-hydroxy-3-keto-5-methylthiopentenyl-1-phosphate phosphatase [EC:3.1.3.87]	0.0276	0.0547	0.0124	0.0287	0.348	1.98	NA	NA	NA
Aminoacid biosynthesis	DNA	K01244	MTN	5'-methylthioadenosine nucleosidase [EC:3.2.2.16]	1.97	3	1.47	1.58	0.731	1.52	NA	NA	NA
Aminoacid biosynthesis	RNA	K01244	MTN	5'-methylthioadenosine nucleosidase [EC:3.2.2.16]	0.707	0.37	0.652	0.351	0.283	1.91	NA	NA	NA
Aminoacid biosynthesis	Transcripts/gene	NA	MTN	5'-methylthioadenosine nucleosidase [EC:3.2.2.16]	0.39	0.126	0.186	0.0867	0.16	3.09	NA	NA	NA
Aminoacid biosynthesis	DNA	K01243	mtnN, mtn, pfs	S-adenosylhomocysteine/5'-methylthioadenosine nucleosidase [EC:3.2.2.9]	124	117	13.9	11.8	0.779	1.06	NA	NA	NA
Aminoacid biosynthesis	RNA	K01243	mtnN, mtn, pfs	S-adenosylhomocysteine/5'-methylthioadenosine nucleosidase [EC:3.2.2.9]	53.7	47.5	7.55	8.25	0.283	1.13	NA	NA	NA
Aminoacid biosynthesis	Transcripts/gene	NA	mtnN, mtn, pfs	S-adenosylhomocysteine/5'-methylthioadenosine nucleosidase [EC:3.2.2.9]	0.436	0.406	0.0712	0.0464	0.671	1.07	NA	NA	NA

Aminoacid biosynthesis	DNA	K01251	ahcY	adenosylhomocysteinase [EC:3.3.1.1]	85.9	105	16.8	26.2	0.563	1.22	NA	NA	NA
Aminoacid biosynthesis	RNA	K01251	ahcY	adenosylhomocysteinase [EC:3.3.1.1]	48.6	59.5	11.2	9.38	0.129	1.22	NA	NA	NA
Aminoacid biosynthesis	Transcripts/gene	NA	ahcY	adenosylhomocysteinase [EC:3.3.1.1]	0.581	0.59	0.146	0.136	1	1.02	NA	NA	NA
Aminoacid biosynthesis	DNA	K14677	ACY1	aminoacylase [EC:3.5.1.14]	0.0271	0.0182	0.0138	0.0135	0.83	1.49	NA	NA	NA
Aminoacid biosynthesis	DNA	K01438	argE	acetylornithine deacetylase [EC:3.5.1.16]	114	117	13.1	15.1	1	1.03	NA	NA	NA
Aminoacid biosynthesis	RNA	K01438	argE	acetylornithine deacetylase [EC:3.5.1.16]	54.1	48	5.12	10.3	0.424	1.13	NA	NA	NA
Aminoacid biosynthesis	Transcripts/gene	NA	argE	acetylornithine deacetylase [EC:3.5.1.16]	0.482	0.409	0.0726	0.0597	0.234	1.18	NA	NA	NA
Aminoacid biosynthesis	DNA	K01439	dapE	succinyl-diaminopimelate desuccinylase [EC:3.5.1.18]	91.3	70.8	19.9	14.9	0.398	1.29	NA	NA	NA
Aminoacid biosynthesis	RNA	K01439	dapE	succinyl-diaminopimelate desuccinylase [EC:3.5.1.18]	30.4	23.1	5.42	5.27	0.16	1.32	NA	NA	NA
Aminoacid biosynthesis	Transcripts/gene	NA	dapE	succinyl-diaminopimelate desuccinylase [EC:3.5.1.18]	0.347	0.335	0.0967	0.0772	0.838	1.03	NA	NA	NA
Aminoacid biosynthesis	DNA	K01450	def	formylmethionine deformylase [EC:3.5.1.31]	0.0251	0.0535	0.018	0.0427	0.62	2.13	NA	NA	NA
Aminoacid biosynthesis	DNA	K05823	NA	N-acetyldiaminopimelate deacetylase [EC:3.5.1.47]	3.57	1.74	1	0.655	0.0268	2.05	-0.00998	-0.00406	-0.0072
Aminoacid biosynthesis	RNA	K05823	NA	N-acetyldiaminopimelate deacetylase [EC:3.5.1.47]	0.659	0.371	0.263	0.135	0.112	1.78	NA	NA	NA
Aminoacid biosynthesis	Transcripts/gene	NA	NA	N-acetyldiaminopimelate deacetylase [EC:3.5.1.47]	0.205	0.243	0.111	0.142	0.671	1.19	NA	NA	NA
Aminoacid biosynthesis	DNA	K01476	rocF, arg	arginase [EC:3.5.3.1]	13.6	13.3	2.07	2.29	1	1.02	NA	NA	NA
Aminoacid biosynthesis	RNA	K01476	rocF, arg	arginase [EC:3.5.3.1]	3.67	3.77	2.32	1.14	0.676	1.03	NA	NA	NA
Aminoacid biosynthesis	Transcripts/gene	NA	rocF, arg	arginase [EC:3.5.3.1]	0.278	0.285	0.171	0.085	0.426	1.03	NA	NA	NA
Aminoacid biosynthesis	DNA	K01496	hisI	phosphoribosyl-AMP cyclohydrolase [EC:3.5.4.19]	12.4	9.54	3.58	2.13	0.62	1.3	NA	NA	NA
Aminoacid biosynthesis	RNA	K01496	hisI	phosphoribosyl-AMP cyclohydrolase [EC:3.5.4.19]	4.12	3.57	0.924	1.06	0.383	1.16	NA	NA	NA
Aminoacid biosynthesis	Transcripts/gene	NA	hisI	phosphoribosyl-ATP pyrophosphohydrolase / phosphoribosyl-AMP cyclohydrolase / histidinol dehydrogenase [EC:3.6.1.31 3.5.4.19 1.1.1.23]	0.638	0.584	0.161	0.152	0.594	1.09	NA	NA	NA
Aminoacid biosynthesis	DNA	K01523	hisE	phosphoribosyl-ATP pyrophosphohydrolase [EC:3.6.1.31]	8.13	6.66	2.23	1.64	0.968	1.22	NA	NA	NA
Aminoacid biosynthesis	RNA	K01523	hisE	phosphoribosyl-ATP pyrophosphohydrolase [EC:3.6.1.31]	4.64	4.42	1.21	0.77	0.788	1.05	NA	NA	NA
Aminoacid biosynthesis	Transcripts/gene	NA	hisE	phosphoribosyl-ATP pyrophosphohydrolase / phosphoribosyl-AMP cyclohydrolase / histidinol dehydrogenase [EC:3.6.1.31 3.5.4.19 1.1.1.23]	0.667	0.604	0.175	0.163	0.594	1.1	NA	NA	NA
Aminoacid biosynthesis	DNA	K11755	hisIE	phosphoribosyl-ATP pyrophosphohydrolase / phosphoribosyl-AMP cyclohydrolase [EC:3.6.1.31 3.5.4.19]	107	115	7.87	6.96	0.511	1.08	NA	NA	NA
Aminoacid biosynthesis	RNA	K11755	hisIE	phosphoribosyl-ATP pyrophosphohydrolase / phosphoribosyl-AMP cyclohydrolase [EC:3.6.1.31 3.5.4.19]	70.9	69.3	15.1	21.1	0.731	1.02	NA	NA	NA
Aminoacid biosynthesis	DNA	K14152	HIS4	phosphoribosyl-ATP pyrophosphohydrolase / phosphoribosyl-AMP cyclohydrolase / histidinol dehydrogenase [EC:3.6.1.31 3.5.4.19 1.1.1.23]	0.0601	0.0554	0.0364	0.0288	1	1.09	NA	NA	NA
Aminoacid biosynthesis	RNA	K14152	HIS4	phosphoribosyl-ATP pyrophosphohydrolase / phosphoribosyl-AMP cyclohydrolase / histidinol dehydrogenase [EC:3.6.1.31 3.5.4.19 1.1.1.23]	0.195	0.239	0.162	0.123	0.471	1.22	NA	NA	NA
Aminoacid biosynthesis	DNA	K09758	asdA	aspartate 4-decarboxylase [EC:4.1.1.12]	3.99	5.87	1.54	1.98	0.383	1.47	NA	NA	NA
Aminoacid biosynthesis	RNA	K09758	asdA	aspartate 4-decarboxylase [EC:4.1.1.12]	0.897	1.37	0.183	0.426	0.207	1.52	NA	NA	NA
Aminoacid biosynthesis	Transcripts/gene	NA	asdA	aspartate 4-decarboxylase [EC:4.1.1.12]	0.253	0.257	0.0979	0.125	0.838	1.02	NA	NA	NA
Aminoacid biosynthesis	DNA	K01586	lysA	diaminopimelate decarboxylase [EC:4.1.1.20]	324	320	23.1	20.9	1	1.01	NA	NA	NA
Aminoacid biosynthesis	RNA	K01586	lysA	diaminopimelate decarboxylase [EC:4.1.1.20]	231	172	40.8	34.9	0.124	1.34	NA	NA	NA
Aminoacid biosynthesis	Transcripts/gene	NA	lysA	bifunctional diaminopimelate decarboxylase / aspartate kinase [EC:4.1.1.20 2.7.2.4]	0.716	0.537	0.137	0.0945	0.125	1.33	NA	NA	NA
Aminoacid biosynthesis	DNA	K12526	lysAC	bifunctional diaminopimelate decarboxylase / aspartate kinase [EC:4.1.1.20 2.7.2.4]	0.0507	0.0816	0.0303	0.0347	0.45	1.61	NA	NA	NA
Aminoacid biosynthesis	RNA	K12526	lysAC	bifunctional diaminopimelate decarboxylase / aspartate kinase [EC:4.1.1.20 2.7.2.4]	0.0611	0.0699	0.0634	0.0519	0.895	1.14	NA	NA	NA
Aminoacid biosynthesis	DNA	K01609	trpC	indole-3-glycerol phosphate synthase [EC:4.1.1.48]	115	110	10.8	6.07	0.989	1.04	NA	NA	NA
Aminoacid biosynthesis	RNA	K01609	trpC	indole-3-glycerol phosphate synthase [EC:4.1.1.48]	70.9	66.1	18.4	22.1	0.73	1.07	NA	NA	NA
Aminoacid biosynthesis	Transcripts/gene	NA	trpC	anthranilate synthase / indole-3-glycerol phosphate synthase / phosphoribosylanthranilate isomerase [EC:4.1.3.27 4.1.1.48 5.3.1.24]	0.594	0.576	0.18	0.161	0.785	1.03	NA	NA	NA
Aminoacid biosynthesis	DNA	K13498	trpCF	indole-3-glycerol phosphate synthase / phosphoribosylanthranilate isomerase [EC:4.1.1.48 5.3.1.24]	10	7.4	5.07	2.03	0.83	1.36	NA	NA	NA
Aminoacid biosynthesis	RNA	K13498	trpCF	indole-3-glycerol phosphate synthase / phosphoribosylanthranilate isomerase [EC:4.1.1.48 5.3.1.24]	2.04	2.09	1.32	0.404	0.572	1.03	NA	NA	NA
Aminoacid biosynthesis	DNA	K01611	speD	S-adenosylmethionine decarboxylase [EC:4.1.1.50]	9	7.16	6.1	2.61	1	1.26	NA	NA	NA
Aminoacid biosynthesis	RNA	K01611	speD	S-adenosylmethionine decarboxylase [EC:4.1.1.50]	8.39	7.17	4.49	2.81	0.895	1.17	NA	NA	NA
Aminoacid biosynthesis	Transcripts/gene	NA	speD	S-adenosylmethionine decarboxylase [EC:4.1.1.50]	1.12	1.01	0.621	0.24	0.983	1.11	NA	NA	NA
Aminoacid biosynthesis	DNA	K01623	ALDO	fructose-bisphosphate aldolase, class I [EC:4.1.2.13]	17.5	16.8	2.77	4.46	1	1.04	NA	NA	NA
Aminoacid biosynthesis	RNA	K01623	ALDO	fructose-bisphosphate aldolase, class I [EC:4.1.2.13]	15.7	13.4	3.91	3.99	0.519	1.18	NA	NA	NA
Aminoacid biosynthesis	DNA	K01624	fbaA	fructose-bisphosphate aldolase, class II [EC:4.1.2.13]	340	329	40.4	21.4	1	1.03	NA	NA	NA
Aminoacid biosynthesis	RNA	K01624	fbaA	fructose-bisphosphate aldolase, class II [EC:4.1.2.13]	2890	2050	780	356	0.141	1.41	NA	NA	NA
Aminoacid biosynthesis	Transcripts/gene	NA	fbaA	fructose-bisphosphate aldolase, class II [EC:4.1.2.13]	7.99	5.85	1.83	0.836	0.125	1.37	NA	NA	NA
Aminoacid biosynthesis	DNA	K01622	NA	fructose 1,6-bisphosphate aldolase/phosphatase [EC:4.1.2.13 3.1.3.11]	7.49	8.4	1.66	2.82	0.875	1.12	NA	NA	NA
Aminoacid biosynthesis	RNA	K01622	NA	fructose 1,6-bisphosphate aldolase/phosphatase [EC:4.1.2.13 3.1.3.11]	4.52	11.3	2.91	4.82	0.101	2.51	NA	NA	NA
Aminoacid biosynthesis	DNA	K08093	hxlA	3-hexulose-6-phosphate synthase [EC:4.1.2.43]	6.43	5.88	1.73	2.4	1	1.09	NA	NA	NA
Aminoacid biosynthesis	DNA	K08093	hxlA	bifunctional enzyme Fae/Hps [EC:4.3.-.- 4.1.2.43]	9.57	9.9	2.39	4.05	1	1.03	NA	NA	NA
Aminoacid biosynthesis	RNA	K08093	hxlA	3-hexulose-6-phosphate synthase [EC:4.1.2.43]	0.777	0.761	0.653	0.258	0.944	1.02	NA	NA	NA
Aminoacid biosynthesis	RNA	K13812	fae-hps	bifunctional enzyme Fae/Hps [EC:4.3.-.- 4.1.2.43]	1.73	5.79	1.47	4.44	0.0729	3.36	NA	NA	NA
Aminoacid biosynthesis	Transcripts/gene	NA	hps-phi	3-hexulose-6-phosphate synthase / 6-phospho-3-hexuloisomerase [EC:4.1.2.43 5.3.1.27]	0.164	0.571	0.0838	0.848	0.136	3.49	NA	NA	NA
Aminoacid biosynthesis	DNA	K13831	hps-phi	3-hexulose-6-phosphate synthase / 6-phospho-3-hexuloisomerase [EC:4.1.2.43 5.3.1.27]	2.04	3.16	0.482	0.562	0.019	1.55	NA	NA	NA
Aminoacid biosynthesis	RNA	K13831	hps-phi	3-hexulose-6-phosphate synthase / 6-phospho-3-hexuloisomerase [EC:4.1.2.43 5.3.1.27]	0.308	0.44	0.128	0.158	0.228	1.43	NA	NA	NA
Aminoacid biosynthesis	DNA	K01620	ltaE	threonine aldolase [EC:4.1.2.5]	197	182	17.3	14.2	0.402	1.08	NA	NA	NA
Aminoacid biosynthesis	RNA	K01620	ltaE	threonine aldolase [EC:4.1.2.5]	93.5	74.6	7.08	13.5	0.068	1.25	NA	NA	NA
Aminoacid biosynthesis	Transcripts/gene	NA	ltaE	threonine aldolase [EC:4.1.2.5]	0.477	0.415	0.0456	0.102	0.408	1.15	NA	NA	NA
Aminoacid biosynthesis	DNA	K01657	trpE	anthranilate synthase component I [EC:4.1.3.27]	216	197	24.8	15.6	0.45	1.1	NA	NA	NA
Aminoacid biosynthesis	RNA	K01657	trpE	anthranilate synthase component I [EC:4.1.3.27]	141	147	42	62.9	0.944	1.04	NA	NA	NA
Aminoacid biosynthesis	DNA	K01658	trpG	anthranilate synthase component II [EC:4.1.3.27]	89	81.4	9.69	6.45	0.504	1.09	NA	NA	NA
Aminoacid biosynthesis	RNA	K01658	trpG	anthranilate synthase component II [EC:4.1.3.27]	50.2	52.4	16.4	22.1	1	1.04	NA	NA	NA
Aminoacid biosynthesis	DNA	K13503	trpEG	anthranilate synthase [EC:4.1.3.27]	0.0166	0.0263	0.00744	0.009	0.46	1.59	NA	NA	NA
Aminoacid biosynthesis	Transcripts/gene	NA	TRP1	anthranilate synthase / indole-3-glycerol phosphate synthase / phosphoribosylanthranilate isomerase [EC:4.1.3.27 4.1.1.48 5.3.1.24]	0.656	0.717	0.223	0.235	0.785	1.09	NA	NA	NA
Aminoacid biosynthesis	DNA	K13497	trpGD	anthranilate synthase/phosphoribosyltransferase [EC:4.1.3.27 2.4.2.18]	15.2	11	7.57	3.14	0.938	1.38	NA	NA	NA
Aminoacid biosynthesis	RNA	K13497	trpGD	anthranilate synthase/phosphoribosyltransferase [EC:4.1.3.27 2.4.2.18]	14.5	10.7	10.9	3.28	1	1.36	NA	NA	NA
Aminoacid biosynthesis	DNA	K01656	TRP3	anthranilate synthase / indole-3-glycerol phosphate synthase [EC:4.1.3.27 4.1.1.48]	0.12	0.124	0.048	0.0847	0.989	1.03	NA	NA	NA
Aminoacid biosynthesis	RNA	K01656	TRP3	anthranilate synthase / indole-3-glycerol phosphate synthase [EC:4.1.3.27 4.1.1.48]	0.0505	0.0637	0.0361	0.0601	0.841	1.26	NA	NA	NA
Aminoacid biosynthesis	DNA	K13501	TRP1	anthranilate synthase / indole-3-glycerol phosphate synthase / phosphoribosylanthranilate isomerase [EC:4.1.3.27 4.1.1.48 5.3.1.24]	0.0918	0.0748	0.0315	0.0228	0.732	1.23	NA	NA	NA
Aminoacid biosynthesis	RNA	K13501	TRP1	anthranilate synthase / indole-3-glycerol phosphate synthase / phosphoribosylanthranilate isomerase [EC:4.1.3.27 4.1.1.48 5.3.1.24]	0.148	0.219	0.04	0.135	0.731	1.48	NA	NA	NA
Aminoacid biosynthesis	DNA	K03785	aroD	3-dehydroquinate dehydratase I [EC:4.2.1.10]	39.8	27.9	8.98	7.7	0.0484	1.43	NA	NA	NA
Aminoacid biosynthesis	RNA	K03785	aroD	3-dehydroquinate dehydratase I [EC:4.2.1.10]	6.89	3.13	1.49	0.625	0.00669	2.2	-0.0248	-0.0108	-0.0172
Aminoacid biosynthesis	DNA	K03786	aroQ, qutE	3-dehydroquinate dehydratase II [EC:4.2.1.10]	84.3	88.2	8.06	5.8	0.911	1.05	NA	NA	NA
Aminoacid biosynthesis	RNA	K03786	aroQ, qutE	3-dehydroquinate dehydratase II [EC:4.2.1.10]	55.1	46.7	8.36	4.72	0.121	1.18	NA	NA	NA
Aminoacid biosynthesis	Transcripts/gene	NA	aroQ, qutE	3-dehydroquinate dehydratase / shikimate dehydrogenase [EC:4.2.1.10 1.1.1.25]	0.49	0.423	0.0616	0.0509	0.136	1.16	NA	NA	NA
Aminoacid biosynthesis	DNA	K13832	aroDE, DHQ-SDH	3-dehydroquinate dehydratase / shikimate dehydrogenase [EC:4.2.1.10 1.1.1.25]	6.8	6.56	1.69	2.78	0.938	1.04	NA	NA	NA
Aminoacid biosynthesis	RNA	K13832	aroDE, DHQ-SDH	3-dehydroquinate dehydratase / shikimate dehydrogenase [EC:4.2.1.10 1.1.1.25]	1.83	1.81	1.05	1.15	1	1.01	NA	NA	NA
Aminoacid biosynthesis	DNA	K08964	mtnB	methylthioribulose-1-phosphate dehydratase [EC:4.2.1.109]	0.0466	0.0698	0.033	0.0268	0.779	1.5	NA	NA	NA
Aminoacid biosynthesis	DNA	K01689	eno	enolase [EC:4.2.1.11]	269	241	32.5	30.3	0.45	1.12	NA	NA	NA
Aminoacid biosynthesis	RNA	K01689	eno	enolase [EC:4.2.1.11]	1300	1260	322	642	0.623	1.04	NA	NA	NA
Aminoacid biosynthesis	Transcripts/gene	NA	eno	enolase [EC:4.2.1.11]	4.86	5.08	1.25	2.12	0.731	1.04	NA	NA	NA
Aminoacid biosynthesis	DNA	K01693	hisB	imidazoleglycerol-phosphate dehydratase [EC:4.2.1.19]	68.7	66.8	11.8	15.7	1	1.03	NA	NA	NA

Aminoacid biosynthesis	RNA	K01693	hisB	imidazoleglycerol-phosphate dehydratase [EC:4.2.1.19]	36.5	30.4	5.61	4.69	0.138	1.2	NA	NA	NA
Aminoacid biosynthesis	Transcripts/gene	NA	hisB	imidazoleglycerol-phosphate dehydratase [EC:4.2.1.19]	0.658	0.541	0.11	0.0459	0.136	1.22	NA	NA	NA
Aminoacid biosynthesis	DNA	K01089	hisB	imidazoleglycerol-phosphate dehydratase / histidinol-phosphatase [EC:4.2.1.19 3.1.3.15]	87.6	90.2	10.9	16.4	1	1.03	NA	NA	NA
Aminoacid biosynthesis	RNA	K01089	hisB	imidazoleglycerol-phosphate dehydratase / histidinol-phosphatase [EC:4.2.1.19 3.1.3.15]	65.2	54.6	9.95	12.8	0.253	1.19	NA	NA	NA
Aminoacid biosynthesis	DNA	K01694	TRP	tryptophan synthase [EC:4.2.1.20]	0.149	0.21	0.0432	0.0848	0.455	1.41	NA	NA	NA
Aminoacid biosynthesis	RNA	K01694	TRP	tryptophan synthase [EC:4.2.1.20]	0.35	0.349	0.248	0.21	0.944	1	NA	NA	NA
Aminoacid biosynthesis	DNA	K01695	trpA	tryptophan synthase alpha chain [EC:4.2.1.20]	132	126	15	8.2	0.938	1.05	NA	NA	NA
Aminoacid biosynthesis	RNA	K01695	trpA	tryptophan synthase alpha chain [EC:4.2.1.20]	87.7	88.1	27.1	23.5	1	1	NA	NA	NA
Aminoacid biosynthesis	DNA	K01696	trpB	tryptophan synthase beta chain [EC:4.2.1.20]	218	213	19.7	16.2	1	1.02	NA	NA	NA
Aminoacid biosynthesis	RNA	K01696	trpB	tryptophan synthase beta chain [EC:4.2.1.20]	177	181	48	55.1	1	1.02	NA	NA	NA
Aminoacid biosynthesis	DNA	K06001	trpB	tryptophan synthase beta chain [EC:4.2.1.20]	253	293	17.9	19.3	0.0717	1.16	NA	NA	NA
Aminoacid biosynthesis	RNA	K06001	trpB	tryptophan synthase beta chain [EC:4.2.1.20]	565	432	201	91.1	0.344	1.31	NA	NA	NA
Aminoacid biosynthesis	Transcripts/gene	NA	trpB	tryptophan synthase beta chain [EC:4.2.1.20]	1.4	1.11	0.475	0.233	0.426	1.26	NA	NA	NA
Aminoacid biosynthesis	DNA	K01697	CBS	cystathionine beta-synthase [EC:4.2.1.22]	5.17	8.92	1.55	2.78	0.299	1.73	NA	NA	NA
Aminoacid biosynthesis	RNA	K01697	CBS	cystathionine beta-synthase [EC:4.2.1.22]	4.21	5.05	1.66	1.13	0.424	1.2	NA	NA	NA
Aminoacid biosynthesis	Transcripts/gene	NA	CBS	cystathionine beta-synthase [EC:4.2.1.22]	0.881	0.611	0.452	0.226	0.426	1.44	NA	NA	NA
Aminoacid biosynthesis	DNA	K01681	acnA	aconitate hydratase [EC:4.2.1.3]	282	283	42.1	65.7	1	1	NA	NA	NA
Aminoacid biosynthesis	RNA	K01681	acnA	aconitate hydratase [EC:4.2.1.3]	240	196	52.8	40.3	0.254	1.22	NA	NA	NA
Aminoacid biosynthesis	Transcripts/gene	NA	acnA	aconitate hydratase 2 / 2-methylisocitrate dehydratase [EC:4.2.1.3 4.2.1.99]	0.88	0.724	0.28	0.187	0.426	1.21	NA	NA	NA
Aminoacid biosynthesis	DNA	K01682	acnB	aconitate hydratase 2 / 2-methylisocitrate dehydratase [EC:4.2.1.3 4.2.1.99]	1.06	1.18	0.432	0.592	1	1.12	NA	NA	NA
Aminoacid biosynthesis	RNA	K01682	acnB	aconitate hydratase 2 / 2-methylisocitrate dehydratase [EC:4.2.1.3 4.2.1.99]	1.68	1.07	0.563	0.78	0.311	1.57	NA	NA	NA
Aminoacid biosynthesis	DNA	K01702	LEU1	3-isopropylmalate dehydratase [EC:4.2.1.33]	0.0412	0.0192	0.0228	0.0158	0.252	2.15	NA	NA	NA
Aminoacid biosynthesis	RNA	K01702	LEU1	3-isopropylmalate dehydratase [EC:4.2.1.33]	0.0739	0.0209	0.072	0.0382	0.151	3.54	NA	NA	NA
Aminoacid biosynthesis	Transcripts/gene	NA	leuD	3-isopropylmalate/(R)-2-methylmalate dehydratase small subunit [EC:4.2.1.33 4.2.1.35]	1.11	0.962	0.272	0.216	0.426	1.15	NA	NA	NA
Aminoacid biosynthesis	DNA	K01703	leuC	3-isopropylmalate/(R)-2-methylmalate dehydratase large subunit [EC:4.2.1.33 4.2.1.35]	264	236	26.3	21.9	0.346	1.12	NA	NA	NA
Aminoacid biosynthesis	RNA	K01703	leuC	3-isopropylmalate/(R)-2-methylmalate dehydratase large subunit [EC:4.2.1.33 4.2.1.35]	295	227	67.7	49.2	0.207	1.3	NA	NA	NA
Aminoacid biosynthesis	DNA	K01704	leuD	3-isopropylmalate/(R)-2-methylmalate dehydratase small subunit [EC:4.2.1.33 4.2.1.35]	104	92.6	10.2	5.78	0.255	1.12	NA	NA	NA
Aminoacid biosynthesis	RNA	K01704	leuD	3-isopropylmalate/(R)-2-methylmalate dehydratase small subunit [EC:4.2.1.33 4.2.1.35]	108	87.7	24.2	20.1	0.283	1.23	NA	NA	NA
Aminoacid biosynthesis	Transcripts/gene	NA	leuD	3-isopropylmalate/(R)-2-methylmalate dehydratase small subunit [EC:4.2.1.33 4.2.1.35]	1.11	0.962	0.274	0.216	0.426	1.15	NA	NA	NA
Aminoacid biosynthesis	DNA	K01705	LYS4	homoaconitate hydratase [EC:4.2.1.36]	0.462	0.389	0.103	0.177	0.911	1.19	NA	NA	NA
Aminoacid biosynthesis	RNA	K01705	LYS4	homoaconitate hydratase [EC:4.2.1.36]	0.177	0.293	0.116	0.287	0.572	1.65	NA	NA	NA
Aminoacid biosynthesis	Transcripts/gene	NA	LYS4	homoaconitate hydratase [EC:4.2.1.36]	0.44	0.853	0.382	1.07	0.671	1.94	NA	NA	NA
Aminoacid biosynthesis	DNA	K04518	pheA2	prephenate dehydratase [EC:4.2.1.51]	109	109	9.98	8.78	1	1	NA	NA	NA
Aminoacid biosynthesis	RNA	K04518	pheA2	prephenate dehydratase [EC:4.2.1.51]	99.7	55.9	35	22.7	0.113	1.78	NA	NA	NA
Aminoacid biosynthesis	Transcripts/gene	NA	pheA2	chorismate mutase / prephenate dehydratase [EC:5.4.99.5 4.2.1.51]	0.607	0.367	0.172	0.0883	0.097	1.65	NA	NA	NA
Aminoacid biosynthesis	DNA	K01713	pheC	cyclohexadienyl dehydratase [EC:4.2.1.51 4.2.1.91]	72.3	73.5	13	14.3	1	1.02	NA	NA	NA
Aminoacid biosynthesis	RNA	K01713	pheC	cyclohexadienyl dehydratase [EC:4.2.1.51 4.2.1.91]	24.3	20.8	9.35	5.31	0.843	1.17	NA	NA	NA
Aminoacid biosynthesis	DNA	K01687	ilvD	dihydroxy-acid dehydratase [EC:4.2.1.9]	364	332	46.8	28.3	0.779	1.1	NA	NA	NA
Aminoacid biosynthesis	RNA	K01687	ilvD	dihydroxy-acid dehydratase [EC:4.2.1.9]	453	304	81.9	29.2	0.0269	1.49	-0.0221	-0.0056	-0.0114
Aminoacid biosynthesis	Transcripts/gene	NA	ilvD	dihydroxy-acid dehydratase [EC:4.2.1.9]	1.27	0.92	0.294	0.12	0.097	1.38	NA	NA	NA
Aminoacid biosynthesis	Transcripts/gene	NA	pheC	cyclohexadienyl dehydratase [EC:4.2.1.51 4.2.1.91]	0.344	0.283	0.139	0.0515	0.636	1.22	NA	NA	NA
Aminoacid biosynthesis	Transcripts/gene	NA	NA	aconitate hydratase 2 / 2-methylisocitrate dehydratase [EC:4.2.1.3 4.2.1.99]	1.92	0.898	1.22	0.44	0.177	2.14	NA	NA	NA
Aminoacid biosynthesis	DNA	K01733	thrC	threonine synthase [EC:4.2.3.1]	302	308	33.6	26.5	1	1.02	NA	NA	NA
Aminoacid biosynthesis	RNA	K01733	thrC	threonine synthase [EC:4.2.3.1]	269	221	39.8	30.7	0.167	1.22	NA	NA	NA
Aminoacid biosynthesis	Transcripts/gene	NA	thrC	threonine synthase [EC:4.2.3.1]	0.901	0.721	0.183	0.102	0.136	1.25	NA	NA	NA
Aminoacid biosynthesis	DNA	K01735	aroB	3-dehydroquinate synthase [EC:4.2.3.4]	201	195	19.4	8.83	0.875	1.03	NA	NA	NA
Aminoacid biosynthesis	RNA	K01735	aroB	3-dehydroquinate synthase [EC:4.2.3.4]	118	95.9	12.8	13.1	0.0466	1.23	NA	NA	NA
Aminoacid biosynthesis	Transcripts/gene	NA	aroB	pentafunctional AROM polypeptide [EC:4.2.3.4 4.2.1.10 1.1.1.25 2.7.1.71 2.5.1.19]	0.577	0.481	0.0738	0.0552	0.097	1.2	NA	NA	NA
Aminoacid biosynthesis	DNA	K13830	ARO1	pentafunctional AROM polypeptide [EC:4.2.3.4 4.2.1.10 1.1.1.25 2.7.1.71 2.5.1.19]	0.625	1.02	0.203	0.177	0.0425	1.63	NA	NA	NA
Aminoacid biosynthesis	RNA	K13830	ARO1	pentafunctional AROM polypeptide [EC:4.2.3.4 4.2.1.10 1.1.1.25 2.7.1.71 2.5.1.19]	0.45	0.36	0.229	0.0671	0.73	1.25	NA	NA	NA
Aminoacid biosynthesis	DNA	K01736	aroC	chorismate synthase [EC:4.2.3.5]	247	243	23.9	19.2	1	1.02	NA	NA	NA
Aminoacid biosynthesis	RNA	K01736	aroC	chorismate synthase [EC:4.2.3.5]	152	113	21.3	17.8	0.0297	1.35	NA	NA	NA
Aminoacid biosynthesis	Transcripts/gene	NA	aroC	chorismate synthase [EC:4.2.3.5]	0.623	0.466	0.128	0.0826	0.125	1.34	NA	NA	NA
Aminoacid biosynthesis	DNA	K01750	ocd	ornithine cyclodeaminase [EC:4.3.1.12]	48.7	29.3	10.5	5.7	0.0408	1.66	-0.0209	-0.00336	-0.0128
Aminoacid biosynthesis	RNA	K01750	ocd	ornithine cyclodeaminase [EC:4.3.1.12]	15.1	13.8	6.9	5.37	0.788	1.09	NA	NA	NA
Aminoacid biosynthesis	Transcripts/gene	NA	ocd	ornithine cyclodeaminase [EC:4.3.1.12]	0.313	0.47	0.12	0.122	0.155	1.5	NA	NA	NA
Aminoacid biosynthesis	DNA	K01752	sdaA	L-serine dehydratase [EC:4.3.1.17]	129	125	34.8	15.3	1	1.03	NA	NA	NA
Aminoacid biosynthesis	RNA	K01752	sdaA	L-serine dehydratase [EC:4.3.1.17]	39.6	34.9	8.39	3.75	0.471	1.14	NA	NA	NA
Aminoacid biosynthesis	Transcripts/gene	NA	sdaA	L-serine dehydratase [EC:4.3.1.17]	0.317	0.282	0.067	0.0386	0.554	1.12	NA	NA	NA
Aminoacid biosynthesis	DNA	K01754	ilvA, tdcB	threonine dehydratase [EC:4.3.1.19]	109	85.7	22.2	21.4	0.563	1.27	NA	NA	NA
Aminoacid biosynthesis	RNA	K01754	ilvA, tdcB	threonine dehydratase [EC:4.3.1.19]	67.9	51.7	13	8.9	0.158	1.31	NA	NA	NA
Aminoacid biosynthesis	Transcripts/gene	NA	ilvA, tdcB	threonine dehydratase [EC:4.3.1.19]	0.634	0.627	0.11	0.154	0.731	1.01	NA	NA	NA
Aminoacid biosynthesis	DNA	K01755	argH	argininosuccinate lyase [EC:4.3.2.1]	314	307	37	21.4	1	1.02	NA	NA	NA
Aminoacid biosynthesis	RNA	K01755	argH	argininosuccinate lyase [EC:4.3.2.1]	226	195	22	18	0.101	1.16	NA	NA	NA
Aminoacid biosynthesis	Transcripts/gene	NA	argH	argininosuccinate lyase / amino-acid N-acetyltransferase [EC:4.3.2.1 2.3.1.1]	0.73	0.637	0.126	0.0491	0.234	1.15	NA	NA	NA
Aminoacid biosynthesis	DNA	K14681	argHA	argininosuccinate lyase / amino-acid N-acetyltransferase [EC:4.3.2.1 2.3.1.1]	0.129	0.146	0.0998	0.0579	0.968	1.13	NA	NA	NA
Aminoacid biosynthesis	RNA	K14681	argHA	argininosuccinate lyase / amino-acid N-acetyltransferase [EC:4.3.2.1 2.3.1.1]	0.0537	0.0326	0.0356	0.0352	0.517	1.65	NA	NA	NA
Aminoacid biosynthesis	DNA	K01714	dapA	4-hydroxy-tetrahydrodipicolinate synthase [EC:4.3.3.7]	300	302	35.3	28.6	1	1.01	NA	NA	NA
Aminoacid biosynthesis	RNA	K01714	dapA	4-hydroxy-tetrahydrodipicolinate synthase [EC:4.3.3.7]	305	255	46.2	33.9	0.207	1.19	NA	NA	NA
Aminoacid biosynthesis	Transcripts/gene	NA	dapA	4-hydroxy-tetrahydrodipicolinate synthase [EC:4.3.3.7]	1.03	0.85	0.174	0.129	0.155	1.21	NA	NA	NA
Aminoacid biosynthesis	DNA	K01758	CTH	cystathionine gamma-lyase [EC:4.4.1.1]	74.9	80.5	8.38	15.8	0.731	1.07	NA	NA	NA
Aminoacid biosynthesis	RNA	K01758	CTH	cystathionine gamma-lyase [EC:4.4.1.1]	18.4	17.2	4.08	3.45	0.788	1.07	NA	NA	NA
Aminoacid biosynthesis	Transcripts/gene	NA	CTH	cystathionine gamma-lyase [EC:4.4.1.1]	0.245	0.215	0.0492	0.0251	0.426	1.14	NA	NA	NA
Aminoacid biosynthesis	DNA	K01761	NA	methionine-gamma-lyase [EC:4.4.1.11]	27.4	27.2	5.8	7.82	1	1.01	NA	NA	NA
Aminoacid biosynthesis	RNA	K01761	NA	methionine-gamma-lyase [EC:4.4.1.11]	31	23.3	7.28	4.29	0.152	1.33	NA	NA	NA
Aminoacid biosynthesis	Transcripts/gene	NA	NA	methionine-gamma-lyase [EC:4.4.1.11]	1.2	0.909	0.492	0.251	0.486	1.32	NA	NA	NA
Aminoacid biosynthesis	DNA	K01762	ACS	1-aminocyclopropane-1-carboxylate synthase [EC:4.4.1.14]	0.00858	0.0089	0.00462	0.0069	1	1.03	NA	NA	NA
Aminoacid biosynthesis	RNA	K01762	ACS	1-aminocyclopropane-1-carboxylate synthase [EC:4.4.1.14]	0.0585	0.042	0.0555	0.0628	0.73	1.39	NA	NA	NA
Aminoacid biosynthesis	Transcripts/gene	NA	ACS	1-aminocyclopropane-1-carboxylate synthase [EC:4.4.1.14]	7.8	4.79	11	6.89	0.751	1.63	NA	NA	NA
Aminoacid biosynthesis	DNA	K05396	dcyD	D-cysteine desulfhydrase [EC:4.4.1.15]	13.9	17.5	3.38	5.54	0.563	1.26	NA	NA	NA
Aminoacid biosynthesis	RNA	K05396	dcyD	D-cysteine desulfhydrase [EC:4.4.1.15]	5.94	5	2.86	1.29	0.788	1.19	NA	NA	NA
Aminoacid biosynthesis	Transcripts/gene	NA	dcyD	D-cysteine desulfhydrase [EC:4.4.1.15]	0.424	0.306	0.137	0.0924	0.344	1.39	NA	NA	NA
Aminoacid biosynthesis	DNA	K07173	luxS	S-ribosylhomocysteine lyase [EC:4.4.1.21]	88.5	82.2	10.1	6.64	0.504	1.08	NA	NA	NA
Aminoacid biosynthesis	RNA	K07173	luxS	S-ribosylhomocysteine lyase [EC:4.4.1.21]	49	44.5	5.87	7.67	0.471	1.1	NA	NA	NA

Aminoacid biosynthesis	Transcripts/gene	NA	luxS	S-ribosylhomocysteine lyase [EC:4.4.1.21]	0.557	0.54	0.0682	0.0659	0.731	1.03	NA	NA	NA
Aminoacid biosynthesis	DNA	K01760	metC	cystathionine beta-lyase [EC:4.4.1.8]	36.4	32.2	11.4	6.73	1	1.13	NA	NA	NA
Aminoacid biosynthesis	RNA	K01760	metC	cystathionine beta-lyase [EC:4.4.1.8]	43.3	6.04	39.2	2.66	0.00669	7.17	NA	NA	NA
Aminoacid biosynthesis	DNA	K14155	patB, malY	cystathione beta-lyase [EC:4.4.1.8]	331	282	30.6	10.9	0.033	1.17	-0.0154	-0.00544	-0.0107
Aminoacid biosynthesis	RNA	K14155	patB, malY	cystathione beta-lyase [EC:4.4.1.8]	150	96.3	31.3	11.7	0.00669	1.56	NA	NA	NA
Aminoacid biosynthesis	Transcripts/gene	NA	patB, malY	cystathione beta-lyase [EC:4.4.1.8]	0.535	0.327	0.193	0.0417	0.097	1.64	NA	NA	NA
Aminoacid biosynthesis	DNA	K01778	dapF	diaminopimelate epimerase [EC:5.1.1.7]	169	162	15.3	12.3	0.875	1.04	NA	NA	NA
Aminoacid biosynthesis	RNA	K01778	dapF	diaminopimelate epimerase [EC:5.1.1.7]	50.1	37.4	13.8	12.5	0.253	1.34	NA	NA	NA
Aminoacid biosynthesis	Transcripts/gene	NA	dapF	diaminopimelate epimerase [EC:5.1.1.7]	0.295	0.227	0.0675	0.061	0.177	1.3	NA	NA	NA
Aminoacid biosynthesis	DNA	K01783	rpe	ribulose-phosphate 3-epimerase [EC:5.1.3.1]	178	183	17.1	11.5	0.968	1.03	NA	NA	NA
Aminoacid biosynthesis	RNA	K01783	rpe	ribulose-phosphate 3-epimerase [EC:5.1.3.1]	146	136	17.1	13.6	0.384	1.08	NA	NA	NA
Aminoacid biosynthesis	Transcripts/gene	NA	rpe	ribulose-phosphate 3-epimerase [EC:5.1.3.1]	0.833	0.747	0.165	0.0753	0.554	1.12	NA	NA	NA
Aminoacid biosynthesis	DNA	K01803	tpiA	triosephosphate isomerase (TIM) [EC:5.3.1.1]	213	214	19.4	9.76	1	1	NA	NA	NA
Aminoacid biosynthesis	RNA	K01803	tpiA	triosephosphate isomerase (TIM) [EC:5.3.1.1]	880	800	126	119	0.572	1.1	NA	NA	NA
Aminoacid biosynthesis	Transcripts/gene	NA	tpiA	triosephosphate isomerase (TIM) [EC:5.3.1.1]	4.16	3.75	0.701	0.558	0.408	1.11	NA	NA	NA
Aminoacid biosynthesis	DNA	K01814	hisA	phosphoribosylformimino-5-aminoimidazole carboxamide ribotide isomerase [EC:5.3.1.16]	138	135	16.1	12.2	1	1.02	NA	NA	NA
Aminoacid biosynthesis	RNA	K01814	hisA	phosphoribosylformimino-5-aminoimidazole carboxamide ribotide isomerase [EC:5.3.1.16]	71.6	54.5	15.8	6.9	0.122	1.31	NA	NA	NA
Aminoacid biosynthesis	Transcripts/gene	NA	hisA	phosphoribosylformimino-5-aminoimidazole carboxamide ribotide isomerase [EC:5.3.1.16]	0.532	0.407	0.172	0.0688	0.234	1.31	NA	NA	NA
Aminoacid biosynthesis	DNA	K08963	mtnA	methylthioribose-1-phosphate isomerase [EC:5.3.1.23]	52.9	56.9	7.89	14.8	0.875	1.07	NA	NA	NA
Aminoacid biosynthesis	RNA	K08963	mtnA	methylthioribose-1-phosphate isomerase [EC:5.3.1.23]	20.7	19.9	6.12	4.78	0.895	1.04	NA	NA	NA
Aminoacid biosynthesis	Transcripts/gene	NA	mtnA	methylthioribose-1-phosphate isomerase [EC:5.3.1.23]	0.401	0.369	0.131	0.12	0.902	1.09	NA	NA	NA
Aminoacid biosynthesis	DNA	K01817	trpF	phosphoribosylanthranilate isomerase [EC:5.3.1.24]	87.8	85.9	9.52	4.22	1	1.02	NA	NA	NA
Aminoacid biosynthesis	RNA	K01817	trpF	phosphoribosylanthranilate isomerase [EC:5.3.1.24]	34.7	30.5	10.7	10.1	0.623	1.14	NA	NA	NA
Aminoacid biosynthesis	Transcripts/gene	NA	trpF	anthranilate synthase / indole-3-glycerol phosphate synthase / phosphoribosylanthranilate isomerase [EC:4.1.3.27 4.1.1.48 5.3.1.24]	0.387	0.349	0.14	0.0942	0.671	1.11	NA	NA	NA
Aminoacid biosynthesis	DNA	K08094	hxlB	6-phospho-3-hexuloisomerase [EC:5.3.1.27]	10.5	8.01	3.05	2.74	0.779	1.31	NA	NA	NA
Aminoacid biosynthesis	RNA	K08094	hxlB	6-phospho-3-hexuloisomerase [EC:5.3.1.27]	1.91	1.93	0.779	0.632	1	1.01	NA	NA	NA
Aminoacid biosynthesis	Transcripts/gene	NA	hxlB	3-hexulose-6-phosphate synthase / 6-phospho-3-hexuloisomerase [EC:4.1.2.43 5.3.1.27]	0.188	0.23	0.0822	0.103	0.671	1.22	NA	NA	NA
Aminoacid biosynthesis	DNA	K01807	rpiA	ribose 5-phosphate isomerase A [EC:5.3.1.6]	11.8	9.61	3.49	2.1	0.676	1.23	NA	NA	NA
Aminoacid biosynthesis	RNA	K01807	rpiA	ribose 5-phosphate isomerase A [EC:5.3.1.6]	3.39	2.33	1.6	0.518	0.424	1.45	NA	NA	NA
Aminoacid biosynthesis	DNA	K01808	rpiB	ribose 5-phosphate isomerase B [EC:5.3.1.6]	141	137	13	11.9	0.911	1.03	NA	NA	NA
Aminoacid biosynthesis	RNA	K01808	rpiB	ribose 5-phosphate isomerase B [EC:5.3.1.6]	198	222	16.6	56.7	0.471	1.12	NA	NA	NA
Aminoacid biosynthesis	Transcripts/gene	NA	rpiB	ribose 5-phosphate isomerase B [EC:5.3.1.6]	1.32	1.53	0.135	0.365	0.354	1.16	NA	NA	NA
Aminoacid biosynthesis	Transcripts/gene	NA	tal-pgi	transaldolase / glucose-6-phosphate isomerase [EC:2.2.1.2 5.3.1.9]	2.16	2.12	0.38	0.317	0.785	1.02	NA	NA	NA
Aminoacid biosynthesis	DNA	K08965	mtnW	2,3-diketo-5-methylthiopentyl-1-phosphate enolase [EC:5.3.2.5]	2.19	1.05	0.768	0.425	0.0752	2.09	-0.0132	-0.00388	-0.00848
Aminoacid biosynthesis	RNA	K08965	mtnW	2,3-diketo-5-methylthiopentyl-1-phosphate enolase [EC:5.3.2.5]	4.49	0.441	9.01	0.263	0.11	10.2	NA	NA	NA
Aminoacid biosynthesis	Transcripts/gene	NA	mtnW	2,3-diketo-5-methylthiopentyl-1-phosphate enolase [EC:5.3.2.5]	1.54	0.447	2.49	0.224	0.382	3.45	NA	NA	NA
Aminoacid biosynthesis	DNA	K01834	gpmA	2,3-bisphosphoglycerate-dependent phosphoglycerate mutase [EC:5.4.2.11]	728	703	54.8	24.6	0.875	1.03	NA	NA	NA
Aminoacid biosynthesis	RNA	K01834	gpmA	2,3-bisphosphoglycerate-dependent phosphoglycerate mutase [EC:5.4.2.11]	1050	886	103	84.4	0.0881	1.19	NA	NA	NA
Aminoacid biosynthesis	Transcripts/gene	NA	gpmA	2,3-bisphosphoglycerate-dependent phosphoglycerate mutase [EC:5.4.2.11]	1.45	1.26	0.177	0.0855	0.125	1.15	NA	NA	NA
Aminoacid biosynthesis	DNA	K01850	NA	chorismate mutase [EC:5.4.99.5]	0.708	0.779	0.249	0.513	1	1.1	NA	NA	NA
Aminoacid biosynthesis	RNA	K01850	NA	chorismate mutase [EC:5.4.99.5]	0.132	0.128	0.121	0.071	0.843	1.03	NA	NA	NA
Aminoacid biosynthesis	DNA	K04092	tyrA1	chorismate mutase [EC:5.4.99.5]	0.0607	0.0408	0.016	0.0111	0.174	1.49	NA	NA	NA
Aminoacid biosynthesis	DNA	K04093	pheA1	chorismate mutase [EC:5.4.99.5]	1.99	2.12	0.57	0.733	1	1.07	NA	NA	NA
Aminoacid biosynthesis	RNA	K04093	pheA1	chorismate mutase [EC:5.4.99.5]	0.502	1.05	0.484	0.518	0.0867	2.08	NA	NA	NA
Aminoacid biosynthesis	DNA	K04516	aroA	chorismate mutase [EC:5.4.99.5]	118	118	14.3	14.6	1	1	NA	NA	NA
Aminoacid biosynthesis	RNA	K04516	aroA	chorismate mutase [EC:5.4.99.5]	157	90.9	47.9	41.8	0.142	1.73	NA	NA	NA
Aminoacid biosynthesis	DNA	K06208	aroH	chorismate mutase [EC:5.4.99.5]	2.48	2.86	0.798	2.61	0.989	1.15	NA	NA	NA
Aminoacid biosynthesis	RNA	K06208	aroH	chorismate mutase [EC:5.4.99.5]	0.478	1.1	0.284	1.25	0.623	2.3	NA	NA	NA
Aminoacid biosynthesis	DNA	K06209	pheB	chorismate mutase [EC:5.4.99.5]	21.5	18.6	4.41	4.09	0.938	1.15	NA	NA	NA
Aminoacid biosynthesis	RNA	K06209	pheB	chorismate mutase [EC:5.4.99.5]	8.63	5.56	2.7	1.58	0.142	1.55	-0.0271	-0.00397	-0.0162
Aminoacid biosynthesis	Transcripts/gene	NA	tyrA	chorismate mutase / prephenate dehydrogenase [EC:5.4.99.5 1.3.1.12]	0.897	0.541	0.24	0.187	0.125	1.66	NA	NA	NA
Aminoacid biosynthesis	DNA	K14187	tyrA	chorismate mutase / prephenate dehydrogenase [EC:5.4.99.5 1.3.1.12]	0.592	0.777	0.166	0.261	0.676	1.31	NA	NA	NA
Aminoacid biosynthesis	RNA	K14187	tyrA	chorismate mutase / prephenate dehydrogenase [EC:5.4.99.5 1.3.1.12]	0.131	0.218	0.159	0.199	0.623	1.66	NA	NA	NA
Aminoacid biosynthesis	DNA	K14170	pheA	chorismate mutase / prephenate dehydratase [EC:5.4.99.5 4.2.1.51]	76.4	74	11.7	15.5	1	1.03	NA	NA	NA
Aminoacid biosynthesis	RNA	K14170	pheA	chorismate mutase / prephenate dehydratase [EC:5.4.99.5 4.2.1.51]	29.5	18.1	5.94	3.42	0.0252	1.63	-0.0314	-0.0101	-0.0218
Aminoacid biosynthesis	DNA	K01914	asnA	aspartate--ammonia ligase [EC:6.3.1.1]	204	194	11.1	6.2	0.504	1.05	NA	NA	NA
Aminoacid biosynthesis	RNA	K01914	asnA	aspartate--ammonia ligase [EC:6.3.1.1]	206	169	68	23.4	0.344	1.22	NA	NA	NA
Aminoacid biosynthesis	Transcripts/gene	NA	asnA	aspartate--ammonia ligase [EC:6.3.1.1]	1.01	0.872	0.305	0.126	0.594	1.16	NA	NA	NA
Aminoacid biosynthesis	DNA	K01915	glnA	glutamine synthetase [EC:6.3.1.2]	963	989	108	70.4	1	1.03	NA	NA	NA
Aminoacid biosynthesis	RNA	K01915	glnA	glutamine synthetase [EC:6.3.1.2]	538	459	63.9	62.1	0.207	1.17	NA	NA	NA
Aminoacid biosynthesis	Transcripts/gene	NA	glnA	glutamine synthetase [EC:6.3.1.2]	0.565	0.467	0.0939	0.0684	0.136	1.21	NA	NA	NA
Aminoacid biosynthesis	DNA	K01948	CPS1	carbamoyl-phosphate synthase (ammonia) [EC:6.3.4.16]	0.543	1.04	0.131	0.638	0.0977	1.91	NA	NA	NA
Aminoacid biosynthesis	RNA	K01948	CPS1	carbamoyl-phosphate synthase (ammonia) [EC:6.3.4.16]	0.545	0.425	0.233	0.239	0.519	1.28	NA	NA	NA
Aminoacid biosynthesis	Transcripts/gene	NA	CPS1	carbamoyl-phosphate synthase (ammonia) [EC:6.3.4.16]	1.05	0.452	0.478	0.222	0.097	2.33	NA	NA	NA
Aminoacid biosynthesis	DNA	K01940	argG	argininosuccinate synthase [EC:6.3.4.5]	321	316	33.4	18.4	1	1.02	NA	NA	NA
Aminoacid biosynthesis	RNA	K01940	argG	argininosuccinate synthase [EC:6.3.4.5]	360	258	82.9	32.3	0.125	1.39	-0.0273	-0.00326	-0.0147
Aminoacid biosynthesis	Transcripts/gene	NA	argG	argininosuccinate synthase [EC:6.3.4.5]	1.13	0.819	0.249	0.0949	0.125	1.37	NA	NA	NA
Aminoacid biosynthesis	DNA	K01958	pyc	pyruvate carboxylase [EC:6.4.1.1]	69.2	57.8	16.9	16.2	0.83	1.2	NA	NA	NA
Aminoacid biosynthesis	RNA	K01958	pyc	pyruvate carboxylase [EC:6.4.1.1]	45.2	30.6	11.3	6.65	0.0968	1.48	NA	NA	NA
Aminoacid biosynthesis	DNA	K01959	pycA	pyruvate carboxylase subunit A [EC:6.4.1.1]	9.52	9.54	2.05	3.39	1	1	NA	NA	NA
Aminoacid biosynthesis	RNA	K01959	pycA	pyruvate carboxylase subunit A [EC:6.4.1.1]	1.54	3	0.375	1.01	0.0364	1.95	NA	NA	NA
Aminoacid biosynthesis	DNA	K01960	pycB	pyruvate carboxylase subunit B [EC:6.4.1.1]	249	255	26.4	23.4	0.989	1.02	NA	NA	NA
Aminoacid biosynthesis	RNA	K01960	pycB	pyruvate carboxylase subunit B [EC:6.4.1.1]	501	455	80.2	61.6	0.382	1.1	NA	NA	NA
Aminoacid biosynthesis	Transcripts/gene	NA	pycB	pyruvate carboxylase subunit B [EC:6.4.1.1]	1.71	1.52	0.413	0.163	0.636	1.13	NA	NA	NA
Aminoacid biosynthesis	DNA	K00841	patA	aminotransferase [EC:2.6.1.-]	2.49	1.64	1.01	0.459	0.299	1.52	NA	NA	NA
Aminoacid biosynthesis	RNA	K00841	patA	aminotransferase [EC:2.6.1.-]	1.48	0.687	0.999	0.204	0.119	2.16	NA	NA	NA
Aminoacid biosynthesis	DNA	K01663	HIS7	glutamine amidotransferase / cyclase [EC:2.4.2.- 4.1.3.-]	0.425	0.529	0.105	0.174	0.779	1.25	NA	NA	NA
Aminoacid biosynthesis	RNA	K01663	HIS7	glutamine amidotransferase / cyclase [EC:2.4.2.- 4.1.3.-]	0.373	0.313	0.0684	0.0889	0.572	1.19	NA	NA	NA
Aminoacid biosynthesis	DNA	K02500	hisF	cyclase [EC:4.1.3.-]	154	153	18	14.2	1	1	NA	NA	NA
Aminoacid biosynthesis	RNA	K02500	hisF	cyclase [EC:4.1.3.-]	74.6	57.4	15.9	5.71	0.147	1.3	NA	NA	NA
Aminoacid biosynthesis	DNA	K02501	hisH	glutamine amidotransferase [EC:2.4.2.-]	119	115	13.5	9.21	1	1.03	NA	NA	NA
Aminoacid biosynthesis	RNA	K02501	hisH	glutamine amidotransferase [EC:2.4.2.-]	63.5	49.7	8.79	4.79	0.0413	1.28	NA	NA	NA
Aminoacid biosynthesis	DNA	K02502	hisZ	ATP phosphoribosyltransferase regulatory subunit	66.7	63.3	10.6	16.9	1	1.05	NA	NA	NA
Aminoacid biosynthesis	RNA	K02502	hisZ	ATP phosphoribosyltransferase regulatory subunit	19.2	17.2	3.78	4.07	0.424	1.11	NA	NA	NA

Aminoacid biosynthesis	DNA	K05825	avtA	2-aminoadipate transaminase [EC:2.6.1.-]	1.66	1.2	0.564	0.396	0.68	1.38	NA	NA	NA
Aminoacid biosynthesis	RNA	K05825	avtA	2-aminoadipate transaminase [EC:2.6.1.-]	1.09	0.725	0.541	0.23	0.344	1.51	NA	NA	NA
Aminoacid biosynthesis	DNA	K05827	lysX	alpha-aminoadipate--LysW ligase LysX [EC:6.3.2.-]	0.0294	0.0459	0.031	0.0139	0.504	1.56	NA	NA	NA
Aminoacid biosynthesis	DNA	K05831	lysK	LysW-gamma-L-lysine carboxypeptidase	0.0137	0.0092	0.0111	0.0076	0.968	1.5	NA	NA	NA
Aminoacid biosynthesis	DNA	K08969	mtnE, mtnV	aminotransferase [EC:2.6.1.-]	0.849	0.236	0.539	0.102	0.00856	3.6	NA	NA	NA
Aminoacid biosynthesis	RNA	K08969	mtnE, mtnV	aminotransferase [EC:2.6.1.-]	0.257	0.317	0.129	0.264	0.989	1.23	NA	NA	NA
Aminoacid biosynthesis	DNA	K10764	metZ	O-succinylhomoserine sulfhydrylase [EC:2.5.1.-]	0.606	0.52	0.169	0.168	0.938	1.17	NA	NA	NA
Aminoacid biosynthesis	RNA	K10764	metZ	O-succinylhomoserine sulfhydrylase [EC:2.5.1.-]	1.28	0.418	0.96	0.117	0.0285	3.06	NA	NA	NA
Bacterial secretion systems	DNA	K03224	yscN, sctN, hrcN	ATP synthase in type III secretion protein SctN [EC:3.6.3.14]	2.47	8.32	1.22	2.45	0.00856	3.37	0.00724	0.0196	0.0153
Bacterial secretion systems	RNA	K03224	yscN, sctN, hrcN	ATP synthase in type III secretion protein SctN [EC:3.6.3.14]	0.733	1.99	0.343	1.2	0.0651	2.71	NA	NA	NA
Bacterial secretion systems	Transcripts/gene	NA	yscN, sctN, hrcN	ATP synthase in type III secretion protein SctN [EC:3.6.3.14]	0.489	0.226	0.606	0.088	0.52	2.16	NA	NA	NA
Bacterial secretion systems	DNA	K03219	yscC, sctC	type III secretion protein SctC	1.22	3.71	0.595	1.03	0.0233	3.03	0.00785	0.0201	0.0159
Bacterial secretion systems	RNA	K03219	yscC, sctC	type III secretion protein SctC	0.213	0.345	0.0934	0.135	0.142	1.62	NA	NA	NA
Bacterial secretion systems	Transcripts/gene	K03219	yscC, sctC	type III secretion protein SctC	0.23	0.0906	0.172	0.0272	0.52	2.54	NA	NA	NA
Bacterial secretion systems	DNA	K03221	yscF, sctF	type III secretion protein SctF	0.0599	0.0491	0.0665	0.03	1	1.22	NA	NA	NA
Bacterial secretion systems	RNA	K03221	yscF, sctF	type III secretion protein SctF	0.0409	0.0314	0.0356	0.0457	0.843	1.3	NA	NA	NA
Bacterial secretion systems	Transcripts/gene	K03221	yscF, sctF	type III secretion protein SctF	2.32	0.796	3.35	0.991	0.685	2.91	NA	NA	NA
Bacterial secretion systems	DNA	K03222	yscJ, sctJ, hrcJ	type III secretion protein SctJ	0.805	2.52	0.444	0.742	0.0233	3.13	0.00775	0.0207	0.0164
Bacterial secretion systems	RNA	K03222	yscJ, sctJ, hrcJ	type III secretion protein SctJ	0.284	0.789	0.242	0.367	0.0857	2.78	NA	NA	NA
Bacterial secretion systems	Transcripts/gene	K03222	yscJ, sctJ, hrcJ	type III secretion protein SctJ	0.644	0.309	0.886	0.12	0.861	2.09	NA	NA	NA
Bacterial secretion systems	DNA	K03223	yscL, sctL	type III secretion protein SctL	0.462	1.31	0.227	0.377	0.00856	2.83	0.0069	0.0196	0.0153
Bacterial secretion systems	RNA	K03223	yscL, sctL	type III secretion protein SctL	0.0374	0.0712	0.0557	0.0866	0.565	1.91	NA	NA	NA
Bacterial secretion systems	Transcripts/gene	K03223	yscL, sctL	type III secretion protein SctL	0.135	0.055	0.187	0.0756	1	2.46	NA	NA	NA
Bacterial secretion systems	DNA	K03225	yscQ, sctQ, hrcQ	type III secretion protein SctQ	0.0368	0.0719	0.00801	0.0211	0.0536	1.95	0.00992	0.0209	0.0175
Bacterial secretion systems	DNA	K03226	yscR, sctR, hrcR	type III secretion protein SctR	1.23	3.78	0.523	1.21	0.00856	3.06	0.00692	0.0189	0.0141
Bacterial secretion systems	RNA	K03226	yscR, sctR, hrcR	type III secretion protein SctR	0.181	0.617	0.152	0.3	0.0429	3.41	0.0115	0.0387	0.0295
Bacterial secretion systems	Transcripts/gene	K03226	yscR, sctR, hrcR	type III secretion protein SctR	0.154	0.163	0.125	0.0659	0.774	1.06	NA	NA	NA
Bacterial secretion systems	DNA	K03227	yscS, sctS, hrcS	type III secretion protein SctS	0.462	1.54	0.225	0.442	0.00856	3.33	0.0078	0.0203	0.016
Bacterial secretion systems	RNA	K03227	yscS, sctS, hrcS	type III secretion protein SctS	0.0787	0.239	0.0628	0.169	0.0707	3.03	NA	NA	NA
Bacterial secretion systems	Transcripts/gene	K03227	yscS, sctS, hrcS	type III secretion protein SctS	0.34	0.15	0.419	0.0828	0.685	2.27	NA	NA	NA
Bacterial secretion systems	DNA	K03228	yscT, sctT, hrcT	type III secretion protein SctT	1.11	3.57	0.557	1.1	0.00856	3.21	0.00672	0.0201	0.0149
Bacterial secretion systems	RNA	K03228	yscT, sctT, hrcT	type III secretion protein SctT	0.043	0.146	0.045	0.0941	0.115	3.38	NA	NA	NA
Bacterial secretion systems	Transcripts/gene	K03228	yscT, sctT, hrcT	type III secretion protein SctT	0.104	0.0401	0.16	0.017	0.727	2.59	NA	NA	NA
Bacterial secretion systems	DNA	K03229	yscU, sctU, hrcU	type III secretion protein SctU	1.6	5.05	0.719	1.47	0.00856	3.16	0.00737	0.0193	0.0151
Bacterial secretion systems	RNA	K03229	yscU, sctU, hrcU	type III secretion protein SctU	0.234	0.381	0.0841	0.214	0.232	1.63	NA	NA	NA
Bacterial secretion systems	Transcripts/gene	K03229	yscU, sctU, hrcU	type III secretion protein SctU	0.207	0.0749	0.166	0.0382	0.52	2.76	NA	NA	NA
Bacterial secretion systems	DNA	K03230	yscV, sctV, hrcV	type III secretion protein SctV	3.68	12.2	1.64	3.52	0.00856	3.32	0.00734	0.0195	0.0152
Bacterial secretion systems	RNA	K03230	yscV, sctV, hrcV	type III secretion protein SctV	0.983	2.42	0.325	0.974	0.0252	2.47	NA	NA	NA
Bacterial secretion systems	Transcripts/gene	K03230	yscV, sctV, hrcV	type III secretion protein SctV	0.39	0.192	0.432	0.0526	0.52	2.03	NA	NA	NA
Bacterial secretion systems	DNA	K04056	yscO, sctO	type III secretion protein SctO	0.109	0.296	0.071	0.0839	0.0536	2.72	0.0075	0.0194	0.0155
Bacterial secretion systems	DNA	K04057	yscP, sctP	type III secretion protein SctP	0.00512	0.0042	0.00292	0.0041	1	1.23	NA	NA	NA
Bacterial secretion systems	DNA	K04058	yscW, sctW	type III secretion protein SctW	0.17	0.397	0.0981	0.066	0.0484	2.33	0.00713	0.0199	0.0162
Bacterial secretion systems	RNA	K04058	yscW, sctW	type III secretion protein SctW	0.0288	0.0358	0.0362	0.0299	0.725	1.24	NA	NA	NA
Bacterial secretion systems	Transcripts/gene	K04058	yscW, sctW	type III secretion protein SctW	0.34	0.0946	0.67	0.0795	0.985	3.59	NA	NA	NA
Bacterial secretion systems	DNA	K04059	yscX, sctX	type III secretion protein SctX	0.0212	0.0406	0.00892	0.0278	0.628	1.91	NA	NA	NA
PTS and Galactose	DNA	K00011	AKR3	aldehyde reductase [EC:1.1.1.21]	20	18.2	5.73	3.38	1	1.1	NA	NA	NA
PTS and Galactose	RNA	K00011	AKR4	aldehyde reductase [EC:1.1.1.21]	8.61	15.1	6.55	7.18	0.283	1.75	NA	NA	NA
PTS and Galactose	Transcripts/gene	NA	AKR4	aldehyde reductase [EC:1.1.1.21]	0.469	0.825	0.354	0.372	0.268	1.76	NA	NA	NA
PTS and Galactose	DNA	K00094	gatD	galactitol-1-phosphate 5-dehydrogese [EC:1.1.1.251]	3.38	3.13	0.903	0.537	1	1.08	NA	NA	NA
PTS and Galactose	RNA	K00094	gatD	galactitol-1-phosphate 5-dehydrogese [EC:1.1.1.251]	1.55	1.06	0.859	0.453	0.572	1.46	NA	NA	NA
PTS and Galactose	Transcripts/gene	NA	gatD	galactitol-1-phosphate 5-dehydrogese [EC:1.1.1.251]	0.465	0.346	0.259	0.137	0.775	1.34	NA	NA	NA
PTS and Galactose	DNA	K00035	gal	D-galactose 1-dehydrogese [EC:1.1.1.48]	0.0923	0.148	0.0444	0.104	0.83	1.6	NA	NA	NA
PTS and Galactose	DNA	K04618	GAOA	galactose oxidase [EC:1.1.3.9]	0.241	0.26	0.186	0.0977	0.968	1.08	NA	NA	NA
PTS and Galactose	RNA	K04618	GAOA	galactose oxidase [EC:1.1.3.9]	0.0596	0.129	0.0587	0.0669	0.253	2.16	NA	NA	NA
PTS and Galactose	Transcripts/gene	NA	GAOA	galactose oxidase [EC:1.1.3.9]	0.363	0.546	0.554	0.35	0.483	1.5	NA	NA	NA
PTS and Galactose	DNA	K06611	NA	stachyose synthetase [EC:2.4.1.67]	1.96	2.82	0.558	1.2	0.62	1.44	NA	NA	NA
PTS and Galactose	RNA	K06611	NA	stachyose synthetase [EC:2.4.1.67]	0.0791	0.144	0.0569	0.0829	0.312	1.81	NA	NA	NA
PTS and Galactose	Transcripts/gene	NA	NA	stachyose synthetase [EC:2.4.1.67]	0.0478	0.0585	0.0449	0.0391	0.775	1.22	NA	NA	NA
PTS and Galactose	DNA	K00844	HK	hexokise [EC:2.7.1.1]	19	21	6.77	10.7	1	1.11	NA	NA	NA
PTS and Galactose	RNA	K00844	HK	hexokise [EC:2.7.1.1]	9.35	21.9	2.44	26.9	0.73	2.34	NA	NA	NA
PTS and Galactose	Transcripts/gene	NA	HK	hexokise [EC:2.7.1.1]	0.552	0.854	0.241	0.789	0.958	1.55	NA	NA	NA
PTS and Galactose	DNA	K00850	pfkA	6-phosphofructokise 1 [EC:2.7.1.11]	453	429	49.9	55.8	0.83	1.06	NA	NA	NA
PTS and Galactose	RNA	K00850	pfkA	6-phosphofructokise 1 [EC:2.7.1.11]	807	861	194	263	0.97	1.07	NA	NA	NA
PTS and Galactose	Transcripts/gene	NA	pfkA	6-phosphofructokise 1 [EC:2.7.1.11]	1.81	1.99	0.515	0.458	0.775	1.1	NA	NA	NA
PTS and Galactose	DNA	K00917	lacC	tagatose 6-phosphate kise [EC:2.7.1.144]	31.1	13	9.91	2.83	0.00856	2.4	-0.0109	-0.00432	-0.00698
PTS and Galactose	RNA	K00917	lacC	tagatose 6-phosphate kise [EC:2.7.1.144]	21	5.25	17.5	1.75	0.0212	4	NA	NA	NA
PTS and Galactose	Transcripts/gene	NA	lacC	tagatose 6-phosphate kise [EC:2.7.1.144]	0.643	0.433	0.479	0.209	0.686	1.48	NA	NA	NA
PTS and Galactose	DNA	K00845	glk	glucokise [EC:2.7.1.2]	373	375	70.9	27.9	1	1.01	NA	NA	NA
PTS and Galactose	RNA	K00845	glk	glucokise [EC:2.7.1.2]	410	407	58.7	83.3	0.989	1.01	NA	NA	NA
PTS and Galactose	DNA	K12407	GCK	glucokise [EC:2.7.1.2]	0.00688	0.0066	0.00744	0.0087	1	1.05	NA	NA	NA
PTS and Galactose	Transcripts/gene	NA	glk	glucokise [EC:2.7.1.2]	1.13	1.08	0.232	0.178	0.775	1.05	NA	NA	NA
PTS and Galactose	DNA	K00883	dgoK	2-dehydro-3-deoxygalactonokise [EC:2.7.1.58]	1.37	0.456	0.96	0.203	0.109	3.01	-0.0136	-0.00293	-0.00772
PTS and Galactose	RNA	K00883	dgoK	2-dehydro-3-deoxygalactonokise [EC:2.7.1.58]	0.537	0.095	0.344	0.091	0.0212	5.65	-0.0219	-0.00742	-0.0147
PTS and Galactose	Transcripts/gene	NA	dgoK	2-dehydro-3-deoxygalactonokise [EC:2.7.1.58]	0.445	0.201	0.33	0.145	0.286	2.21	NA	NA	NA
PTS and Galactose	DNA	K00849	galK	galactokise [EC:2.7.1.6]	376	392	37	30	0.989	1.04	NA	NA	NA
PTS and Galactose	RNA	K00849	galK	galactokise [EC:2.7.1.6]	680	636	137	145	0.895	1.07	NA	NA	NA
PTS and Galactose	Transcripts/gene	NA	galK	galactokise [EC:2.7.1.6]	1.83	1.62	0.435	0.369	0.701	1.13	NA	NA	NA
PTS and Galactose	DNA	K02744	agaF	PTS system, N-acetylgalactosamine-specific IIA component [EC:2.7.1.69]	18.7	7.54	6.72	3.41	0.0359	2.48	-0.0103	-0.000158	-0.00461
PTS and Galactose	RNA	K02744	agaF	PTS system, N-acetylgalactosamine-specific IIA component [EC:2.7.1.69]	46.9	38.7	21.9	23	0.623	1.21	NA	NA	NA
PTS and Galactose	DNA	K02745	agaV	PTS system, N-acetylgalactosamine-specific IIB component [EC:2.7.1.69]	8.49	0.588	4.51	0.304	0.00856	14.4	-0.0112	-0.00411	-0.00716
PTS and Galactose	RNA	K02745	agaV	PTS system, N-acetylgalactosamine-specific IIB component [EC:2.7.1.69]	59.4	2.8	47.8	3.04	0.00669	21.2	NA	NA	NA
PTS and Galactose	DNA	K02749	glvB	PTS system, arbutin-like IIB component [EC:2.7.1.69]	14.5	3.26	11.1	2.13	0.124	4.46	-0.0175	-0.00237	-0.0103
PTS and Galactose	RNA	K02749	glvB	PTS system, arbutin-like IIB component [EC:2.7.1.69]	1.56	2.9	0.913	4.04	0.73	1.85	NA	NA	NA
PTS and Galactose	DNA	K02752	ascF	PTS system, arbutin-, cellobiose-, and salicin-specific IIB component [EC:2.7.1.69]	1.88	1.13	0.868	0.62	0.457	1.66	NA	NA	NA

PTS and Galactose	RNA	K02752	ascF	PTS system, arbutin-, cellobiose-, and salicin-specific IIB component [EC:2.7.1.69]	0.42	0.329	0.387	0.21	1	1.28	NA	NA	NA
PTS and Galactose	DNA	K02755	bglF	PTS system, beta-glucosides-specific IIA component [EC:2.7.1.69]	136	83.2	34.7	38.6	0.203	1.64	NA	NA	NA
PTS and Galactose	RNA	K02755	bglF	PTS system, beta-glucosides-specific IIA component [EC:2.7.1.69]	84.1	98.6	40.9	55.2	0.843	1.17	NA	NA	NA
PTS and Galactose	DNA	K02756	bglF	PTS system, beta-glucosides-specific IIB component [EC:2.7.1.69]	21.1	7.58	9.8	3.67	0.129	2.78	-0.0121	-0.00165	-0.00486
PTS and Galactose	RNA	K02756	bglF	PTS system, beta-glucosides-specific IIB component [EC:2.7.1.69]	1.79	0.728	0.729	0.371	0.0413	2.46	-0.0249	-0.00645	-0.0154
PTS and Galactose	DNA	K02759	celC	PTS system, cellobiose-specific IIA component [EC:2.7.1.69]	14.6	2.96	5.57	0.905	0.00856	4.94	-0.0126	-0.00723	-0.0102
PTS and Galactose	RNA	K02759	celC	PTS system, cellobiose-specific IIA component [EC:2.7.1.69]	44	12.6	23	6.8	0.047	3.49	NA	NA	NA
PTS and Galactose	DNA	K02760	celA	PTS system, cellobiose-specific IIB component [EC:2.7.1.69]	34.9	6.18	15.3	1.99	0.00856	5.64	-0.0115	-0.00446	-0.00738
PTS and Galactose	RNA	K02760	celA	PTS system, cellobiose-specific IIB component [EC:2.7.1.69]	132	29.9	74.8	15.7	0.00669	4.42	-0.0251	-0.00431	-0.0109
PTS and Galactose	DNA	K02763	gamP	PTS system, D-glucosamine-specific IIA component [EC:2.7.1.69]	38.2	24.3	15.6	17.1	0.301	1.57	NA	NA	NA
PTS and Galactose	RNA	K02763	gamP	PTS system, D-glucosamine-specific IIA component [EC:2.7.1.69]	31.1	82.4	21.5	93.6	0.344	2.65	NA	NA	NA
PTS and Galactose	DNA	K02768	fruB	PTS system, fructose-specific IIA component [EC:2.7.1.69]	118	97.3	29.4	33.9	0.875	1.21	NA	NA	NA
PTS and Galactose	RNA	K02768	fruB	PTS system, fructose-specific IIA component [EC:2.7.1.69]	198	266	78	133	0.572	1.35	NA	NA	NA
PTS and Galactose	DNA	K02769	fruA	PTS system, fructose-specific IIB component [EC:2.7.1.69]	7.48	1.5	3.67	0.583	0.033	4.98	-0.0125	-0.00528	-0.00884
PTS and Galactose	RNA	K02769	fruA	PTS system, fructose-specific IIB component [EC:2.7.1.69]	10.5	6.43	2.44	2.1	0.0736	1.63	NA	NA	NA
PTS and Galactose	DNA	K02773	gatA	PTS system, galactitol-specific IIA component [EC:2.7.1.69]	4.28	1.56	2.61	0.795	0.136	2.73	NA	NA	NA
PTS and Galactose	RNA	K02773	gatA	PTS system, galactitol-specific IIA component [EC:2.7.1.69]	1.15	0.441	0.625	0.289	0.075	2.6	NA	NA	NA
PTS and Galactose	DNA	K02774	gatB	PTS system, galactitol-specific IIB component [EC:2.7.1.69]	3.04	1.08	1.61	0.617	0.11	2.82	NA	NA	NA
PTS and Galactose	RNA	K02774	gatB	PTS system, galactitol-specific IIB component [EC:2.7.1.69]	1.9	0.516	0.797	0.234	0.00669	3.68	-0.0259	-0.0105	-0.0205
PTS and Galactose	DNA	K02777	crr	PTS system, glucose-specific IIA component [EC:2.7.1.69]	28.9	7.75	13.1	2.58	0.00856	3.73	-0.0106	-0.0037	-0.00644
PTS and Galactose	RNA	K02777	crr	PTS system, glucose-specific IIA component [EC:2.7.1.69]	24.3	13.7	7.02	6.14	0.0822	1.78	NA	NA	NA
PTS and Galactose	DNA	K02778	ptsG	PTS system, glucose-specific IIB component [EC:2.7.1.69]	19.7	2.96	9.75	1.49	0.00856	6.66	-0.0111	-0.00237	-0.00667
PTS and Galactose	RNA	K02778	ptsG	PTS system, glucose-specific IIB component [EC:2.7.1.69]	10.9	1.61	6.81	0.997	0.00669	6.74	NA	NA	NA
PTS and Galactose	DNA	K02781	srlB	PTS system, glucitol/sorbitol-specific IIA component [EC:2.7.1.69]	6.68	8.63	2.24	7.63	1	1.29	NA	NA	NA
PTS and Galactose	RNA	K02781	srlB	PTS system, glucitol/sorbitol-specific IIA component [EC:2.7.1.69]	7.19	17.7	5.79	25.4	0.97	2.45	NA	NA	NA
PTS and Galactose	DNA	K02782	srlE	PTS system, glucitol/sorbitol-specific IIB component [EC:2.7.1.69]	21.5	31.7	10.4	21.5	0.83	1.48	NA	NA	NA
PTS and Galactose	RNA	K02782	srlE	PTS system, glucitol/sorbitol-specific IIB component [EC:2.7.1.69]	1.31	2.06	0.951	2.09	0.73	1.57	NA	NA	NA
PTS and Galactose	DNA	K02786	lacF	PTS system, lactose-specific IIA component [EC:2.7.1.69]	5.64	0.919	2.52	0.262	0.00856	6.14	NA	NA	NA
PTS and Galactose	DNA	K02786	lacF	PTS system, lactose-specific IIA component [EC:2.7.1.69]	5.64	0.919	2.52	0.262	0.00856	6.14	-0.0115	-0.00482	-0.00757
PTS and Galactose	RNA	K02786	lacF	PTS system, lactose-specific IIA component [EC:2.7.1.69]	0.546	0.107	0.282	0.0923	0.0285	5.11	NA	NA	NA
PTS and Galactose	DNA	K02787	lacE	PTS system, lactose-specific IIB component [EC:2.7.1.69]	29.3	4.7	12.9	1.42	0.00856	6.24	NA	NA	NA
PTS and Galactose	DNA	K02787	lacE	PTS system, lactose-specific IIB component [EC:2.7.1.69]	29.3	4.7	12.9	1.42	0.00856	6.24	-0.0118	-0.00503	-0.00798
PTS and Galactose	RNA	K02787	lacE	PTS system, lactose-specific IIB component [EC:2.7.1.69]	1.75	0.235	1.48	0.169	0.00669	7.44	NA	NA	NA
PTS and Galactose	DNA	K02790	malX	PTS system, maltose and glucose-specific IIB component [EC:2.7.1.69]	26.3	8.75	11.2	11.4	0.132	3	-0.0124	-0.00292	-0.00736
PTS and Galactose	RNA	K02790	malX	PTS system, maltose and glucose-specific IIB component [EC:2.7.1.69]	6.18	31.3	6.7	46	0.204	5.06	NA	NA	NA
PTS and Galactose	DNA	K02793	manX	PTS system, mannose-specific IIA component [EC:2.7.1.69]	35.7	20	9.97	8.57	0.171	1.78	-0.0106	-0.00279	-0.00595
PTS and Galactose	RNA	K02793	manX	PTS system, mannose-specific IIA component [EC:2.7.1.69]	484	213	205	92.2	0.0164	2.28	-0.0315	-0.00783	-0.0186
PTS and Galactose	DNA	K02794	manX	PTS system, mannose-specific IIB component [EC:2.7.1.69]	28	15.1	6.32	7.06	0.0696	1.86	-0.0117	-0.00257	-0.00653
PTS and Galactose	RNA	K02794	manX	PTS system, mannose-specific IIB component [EC:2.7.1.69]	149	152	91.7	81.7	0.97	1.02	NA	NA	NA
PTS and Galactose	DNA	K02798	mtlA	PTS system, mannitol-specific IIA component [EC:2.7.1.69]	26.5	32.4	5.23	17.2	0.968	1.22	NA	NA	NA
PTS and Galactose	RNA	K02798	mtlA	PTS system, mannitol-specific IIA component [EC:2.7.1.69]	5.47	5.81	2.08	3.55	0.989	1.06	NA	NA	NA
PTS and Galactose	DNA	K02799	mtlA	PTS system, mannitol-specific IIB component [EC:2.7.1.69]	9.09	10.7	2.28	7.83	1	1.18	NA	NA	NA
PTS and Galactose	RNA	K02799	mtlA	PTS system, mannitol-specific IIB component [EC:2.7.1.69]	2.53	2.55	0.651	2.37	0.424	1.01	NA	NA	NA
PTS and Galactose	DNA	K02802	nagE	PTS system, N-acetylglucosamine-specific IIA component [EC:2.7.1.69]	2.57	1.4	1.02	0.669	0.298	1.83	NA	NA	NA
PTS and Galactose	RNA	K02802	nagE	PTS system, N-acetylglucosamine-specific IIA component [EC:2.7.1.69]	8.89	4.62	4.14	2.47	0.164	1.92	NA	NA	NA
PTS and Galactose	DNA	K02803	nagE	PTS system, N-acetylglucosamine-specific IIB component [EC:2.7.1.69]	10.9	11.4	2.47	7.61	0.938	1.05	NA	NA	NA
PTS and Galactose	RNA	K02803	nagE	PTS system, N-acetylglucosamine-specific IIB component [EC:2.7.1.69]	8.46	6.27	3.88	4.93	0.312	1.35	NA	NA	NA
PTS and Galactose	DNA	K02806	ptsN	PTS system, nitrogen regulatory IIA component [EC:2.7.1.69]	7.57	8.43	1.8	3.13	1	1.11	NA	NA	NA
PTS and Galactose	RNA	K02806	ptsN	PTS system, nitrogen regulatory IIA component [EC:2.7.1.69]	12.8	11.6	6.2	8.14	0.73	1.1	NA	NA	NA
PTS and Galactose	DNA	K02808	scrA	PTS system, sucrose-specific IIA component [EC:2.7.1.69]	22	15.4	5.81	7.54	0.504	1.43	NA	NA	NA
PTS and Galactose	RNA	K02808	scrA	PTS system, sucrose-specific IIA component [EC:2.7.1.69]	60.2	53.9	25.2	27.4	0.843	1.12	NA	NA	NA
PTS and Galactose	DNA	K02809	scrA	PTS system, sucrose-specific IIB component [EC:2.7.1.69]	97.6	64.6	23.4	34	0.256	1.51	NA	NA	NA
PTS and Galactose	RNA	K02809	scrA	PTS system, sucrose-specific IIB component [EC:2.7.1.69]	246	240	124	143	0.944	1.03	NA	NA	NA
PTS and Galactose	DNA	K02812	sorF	PTS system, sorbose-specific IIA component [EC:2.7.1.69]	0.0075	0.0028	0.011	0.0041	0.911	2.7	NA	NA	NA
PTS and Galactose	DNA	K02813	sorB	PTS system, sorbose-specific IIB component [EC:2.7.1.69]	0.0869	0.0126	0.0662	0.0154	0.0954	6.88	-0.0167	-0.0031	-0.0122
PTS and Galactose	RNA	K02813	sorB	PTS system, sorbose-specific IIB component [EC:2.7.1.69]	0.0448	0.132	0.0474	0.194	0.843	2.95	NA	NA	NA
PTS and Galactose	DNA	K02817	treP	PTS system, trehalose-specific IIA component [EC:2.7.1.69]	4.66	1.45	2.55	0.573	0.0752	3.21	NA	NA	NA
PTS and Galactose	RNA	K02817	treP	PTS system, trehalose-specific IIA component [EC:2.7.1.69]	10.5	4.05	7.28	2.12	0.109	2.59	NA	NA	NA
PTS and Galactose	DNA	K02818	treB	PTS system, trehalose-specific IIB component [EC:2.7.1.69]	31.9	19.4	10.7	9.67	0.298	1.65	NA	NA	NA
PTS and Galactose	RNA	K02818	treB	PTS system, trehalose-specific IIB component [EC:2.7.1.69]	18.6	15	13	6.37	1	1.24	NA	NA	NA
PTS and Galactose	DNA	K02821	sgaA	PTS system, ascorbate-specific IIA component [EC:2.7.1.69]	8.18	6.17	1.91	5.01	0.504	1.33	NA	NA	NA
PTS and Galactose	RNA	K02821	sgaA	PTS system, ascorbate-specific IIA component [EC:2.7.1.69]	7.75	6.95	3.46	3.21	0.788	1.11	NA	NA	NA
PTS and Galactose	DNA	K02822	sgaB	PTS system, ascorbate-specific IIB component [EC:2.7.1.69]	5.57	4.47	1.38	3.54	0.563	1.25	NA	NA	NA
PTS and Galactose	RNA	K02822	sgaB	PTS system, ascorbate-specific IIB component [EC:2.7.1.69]	3.36	8.21	0.794	9.18	0.572	2.44	NA	NA	NA
PTS and Galactose	DNA	K10984	agaB	PTS system, galactosamine-specific IIB component [EC:2.7.1.69]	0.929	0.574	0.624	0.361	0.731	1.62	NA	NA	NA
PTS and Galactose	RNA	K10984	agaB	PTS system, galactosamine-specific IIB component [EC:2.7.1.69]	11.6	7.52	11.3	8.97	0.623	1.55	NA	NA	NA
PTS and Galactose	DNA	K11191	murP	PTS system, N-acetylmuramic acid-specific IIB component [EC:2.7.1.69]	0.397	0.642	0.154	0.265	0.394	1.62	NA	NA	NA
PTS and Galactose	RNA	K11191	murP	PTS system, N-acetylmuramic acid-specific IIB component [EC:2.7.1.69]	0.0893	0.0871	0.0454	0.0618	0.731	1.03	NA	NA	NA
PTS and Galactose	DNA	K11198	mngA, hrsA	PTS system, 2-O-A-mannosyl-D-glycerate-specific IIA component [EC:2.7.1.69]	0.106	0.035	0.0715	0.0278	0.162	3.04	NA	NA	NA
PTS and Galactose	RNA	K11198	mngA, hrsA	PTS system, 2-O-A-mannosyl-D-glycerate-specific IIA component [EC:2.7.1.69]	0.231	0.0206	0.268	0.031	0.144	11.2	NA	NA	NA
PTS and Galactose	Transcripts/gene	NA	mngA, hrsA	PTS system, 2-O-A-mannosyl-D-glycerate-specific IIA component [EC:2.7.1.69]	2.2	2.81	1.17	1.19	0.686	1.28	NA	NA	NA
PTS and Galactose	DNA	K08483	ptsI	phosphotransferase system, enzyme I, PtsI [EC:2.7.3.9]	159	142	31.2	35	0.989	1.12	NA	NA	NA
PTS and Galactose	RNA	K08483	ptsI	phosphotransferase system, enzyme I, PtsI [EC:2.7.3.9]	544	454	189	190	0.424	1.2	NA	NA	NA
PTS and Galactose	DNA	K08484	ptsP	phosphotransferase system, enzyme I, PtsP [EC:2.7.3.9]	1.22	2.24	0.456	0.741	0.127	1.84	NA	NA	NA
PTS and Galactose	RNA	K08484	ptsP	phosphotransferase system, enzyme I, PtsP [EC:2.7.3.9]	0.512	1.98	0.376	2.55	0.283	3.87	NA	NA	NA
PTS and Galactose	DNA	K00965	galT	UDPglucose--hexose-1-phosphate uridylyltransferase [EC:2.7.7.12]	113	98.8	19.9	24.1	0.968	1.15	NA	NA	NA
PTS and Galactose	RNA	K00965	galT	UDPglucose--hexose-1-phosphate uridylyltransferase [EC:2.7.7.12]	50.8	46.3	10.2	12.9	0.519	1.1	NA	NA	NA
PTS and Galactose	Transcripts/gene	NA	galT	UDPglucose--hexose-1-phosphate uridylyltransferase [EC:2.7.7.12]	0.461	0.5	0.128	0.189	0.958	1.08	NA	NA	NA
PTS and Galactose	DNA	K00963	galU, galF	UTP--glucose-1-phosphate uridylyltransferase [EC:2.7.7.9]	54.4	56.8	14.5	12.2	1	1.05	NA	NA	NA
PTS and Galactose	RNA	K00963	galU, galF	UTP--glucose-1-phosphate uridylyltransferase [EC:2.7.7.9]	31.1	30.4	11.9	10.8	0.989	1.02	NA	NA	NA
PTS and Galactose	Transcripts/gene	NA	galU, galF	UTP--glucose-1-phosphate uridylyltransferase [EC:2.7.7.9]	0.577	0.531	0.154	0.12	0.775	1.09	NA	NA	NA
PTS and Galactose	DNA	K01182	NA	oligo-1,6-glucosidase [EC:3.2.1.10]	93.6	101	19.7	36.1	0.938	1.08	NA	NA	NA
PTS and Galactose	RNA	K01182	NA	oligo-1,6-glucosidase [EC:3.2.1.10]	30.6	26.6	3.74	5.23	0.312	1.15	NA	NA	NA
PTS and Galactose	Transcripts/gene	NA	SI	sucrase-isomaltase / oligo-1,6-glucosidase [EC:3.2.1.48 3.2.1.10]	0.335	0.301	0.0478	0.137	0.268	1.12	NA	NA	NA

PTS and Galactose	DNA	K01203	SI	sucrase-isomaltase / oligo-1,6-glucosidase [EC:3.2.1.48 3.2.1.10]	1.14	0.886	0.819	0.232	1	1.29	NA	NA	NA
PTS and Galactose	Transcripts/gene	NA	LCT	lactase-phlorizin hydrolase [EC:3.2.1.108 3.2.1.62]	24.5	19.9	16.2	17.8	0.775	1.23	NA	NA	NA
PTS and Galactose	DNA	K01229	LCT	lactase-phlorizin hydrolase [EC:3.2.1.108 3.2.1.62]	0.154	0.271	0.0387	0.108	0.345	1.76	NA	NA	NA
PTS and Galactose	RNA	K01229	LCT	lactase-phlorizin hydrolase [EC:3.2.1.108 3.2.1.62]	3.82	4.15	2.8	1.57	0.843	1.09	NA	NA	NA
PTS and Galactose	DNA	K01187	malZ	alpha-glucosidase [EC:3.2.1.20]	733	780	110	47.7	0.938	1.06	NA	NA	NA
PTS and Galactose	RNA	K01187	malZ	alpha-glucosidase [EC:3.2.1.20]	360	348	42.1	55.7	0.843	1.03	NA	NA	NA
PTS and Galactose	DNA	K12316	GAA	lysosomal alpha-glucosidase [EC:3.2.1.20]	8.6	7.91	4.82	1.96	1	1.09	NA	NA	NA
PTS and Galactose	RNA	K12316	GAA	lysosomal alpha-glucosidase [EC:3.2.1.20]	1.26	1.89	1.06	0.868	0.283	1.49	NA	NA	NA
PTS and Galactose	DNA	K12317	GANC	neutral alpha-glucosidase C [EC:3.2.1.20]	1.51	1.61	0.934	0.697	0.911	1.07	NA	NA	NA
PTS and Galactose	RNA	K12317	GANC	neutral alpha-glucosidase C [EC:3.2.1.20]	0.137	0.195	0.155	0.165	0.563	1.42	NA	NA	NA
PTS and Galactose	Transcripts/gene	NA	GANC	neutral alpha-glucosidase C [EC:3.2.1.20]	0.495	0.445	0.11	0.0794	0.84	1.11	NA	NA	NA
PTS and Galactose	DNA	K12047	MGAM	maltase-glucoamylase [EC:3.2.1.20 3.2.1.3]	2.86	2.36	1.92	0.574	1	1.21	NA	NA	NA
PTS and Galactose	RNA	K12047	MGAM	maltase-glucoamylase [EC:3.2.1.20 3.2.1.3]	0.563	0.865	0.421	0.366	0.312	1.54	NA	NA	NA
PTS and Galactose	DNA	K01189	GLA	alpha-galactosidase [EC:3.2.1.22]	0.0584	0.107	0.0307	0.0507	0.507	1.83	NA	NA	NA
PTS and Galactose	RNA	K01189	GLA	alpha-galactosidase [EC:3.2.1.22]	0.331	0.562	0.368	0.209	0.188	1.69	NA	NA	NA
PTS and Galactose	DNA	K07406	melA	alpha-galactosidase [EC:3.2.1.22]	34.8	47	7.87	17.3	0.675	1.35	NA	NA	NA
PTS and Galactose	RNA	K07406	melA	alpha-galactosidase [EC:3.2.1.22]	44.4	35.7	32.7	9.01	0.944	1.25	NA	NA	NA
PTS and Galactose	DNA	K07407	galA, rafA	alpha-galactosidase [EC:3.2.1.22]	600	627	59.4	50.9	0.83	1.04	NA	NA	NA
PTS and Galactose	RNA	K07407	galA, rafA	alpha-galactosidase [EC:3.2.1.22]	274	282	59.1	57.3	1	1.03	NA	NA	NA
PTS and Galactose	Transcripts/gene	NA	galA, rafA	alpha-galactosidase [EC:3.2.1.22]	0.509	0.479	0.147	0.105	0.958	1.06	NA	NA	NA
PTS and Galactose	DNA	K01190	lacZ	beta-galactosidase [EC:3.2.1.23]	1910	1970	163	153	0.989	1.03	NA	NA	NA
PTS and Galactose	RNA	K01190	lacZ	beta-galactosidase [EC:3.2.1.23]	868	790	124	152	0.519	1.1	NA	NA	NA
PTS and Galactose	DNA	K12111	ebgA	evolved beta-galactosidase subunit alpha [EC:3.2.1.23]	10.7	8.55	2.11	0.972	0.563	1.25	NA	NA	NA
PTS and Galactose	RNA	K12111	ebgA	evolved beta-galactosidase subunit alpha [EC:3.2.1.23]	2.45	1.86	0.538	0.267	0.141	1.32	NA	NA	NA
PTS and Galactose	DNA	K12308	bgaB, lacA	beta-galactosidase [EC:3.2.1.23]	170	171	27.8	30.6	1	1	NA	NA	NA
PTS and Galactose	RNA	K12308	bgaB, lacA	beta-galactosidase [EC:3.2.1.23]	109	92.2	25	23.3	0.424	1.18	NA	NA	NA
PTS and Galactose	DNA	K12309	GLB1	beta-galactosidase [EC:3.2.1.23]	0.55	0.542	0.204	0.128	1	1.02	NA	NA	NA
PTS and Galactose	RNA	K12309	GLB1	beta-galactosidase [EC:3.2.1.23]	0.308	0.341	0.136	0.171	0.843	1.11	NA	NA	NA
PTS and Galactose	Transcripts/gene	NA	lacZ, GLB1	beta-galactosidase [EC:3.2.1.23]	0.472	0.414	0.0932	0.0894	0.573	1.14	NA	NA	NA
PTS and Galactose	DNA	K01193	sacA	beta-fructofuranosidase [EC:3.2.1.26]	191	201	23.4	90.5	1	1.05	NA	NA	NA
PTS and Galactose	RNA	K01193	sacA	beta-fructofuranosidase [EC:3.2.1.26]	58.9	137	20.4	155	0.676	2.34	NA	NA	NA
PTS and Galactose	Transcripts/gene	NA	sacA	beta-fructofuranosidase [EC:3.2.1.26]	0.313	0.599	0.118	0.488	0.625	1.91	NA	NA	NA
PTS and Galactose	Transcripts/gene	NA	MGAM	maltase-glucoamylase [EC:3.2.1.20 3.2.1.3]	0.324	0.424	0.245	0.199	0.775	1.31	NA	NA	NA
PTS and Galactose	Transcripts/gene	NA	SI	sucrase-isomaltase / oligo-1,6-glucosidase [EC:3.2.1.48 3.2.1.10]	0.873	0.732	0.929	0.406	0.958	1.19	NA	NA	NA
PTS and Galactose	RNA	K01203	SI	sucrase-isomaltase / oligo-1,6-glucosidase [EC:3.2.1.48 3.2.1.10]	0.473	0.607	0.428	0.281	0.623	1.28	NA	NA	NA
PTS and Galactose	Transcripts/gene	NA	LCT	lactase-phlorizin hydrolase [EC:3.2.1.108 3.2.1.62]	24.5	19.9	16.2	17.8	0.775	1.23	NA	NA	NA
PTS and Galactose	DNA	K01220	lacG	6-phospho-beta-galactosidase [EC:3.2.1.85]	26.7	4.25	11.6	1.23	0.00856	6.29	-0.0118	-0.0052	-0.008
PTS and Galactose	RNA	K01220	lacG	6-phospho-beta-galactosidase [EC:3.2.1.85]	2.08	0.325	1.42	0.112	0.00669	6.4	-0.0239	-0.00789	-0.0151
PTS and Galactose	Transcripts/gene	NA	lacG	6-phospho-beta-galactosidase [EC:3.2.1.85]	0.0723	0.0783	0.0257	0.0222	0.701	1.08	NA	NA	NA
PTS and Galactose	DNA	K02079	agaA	N-acetylgalactosamine-6-phosphate deacetylase [EC:3.5.1.25]	0.359	0.334	0.157	0.144	1	1.07	NA	NA	NA
PTS and Galactose	RNA	K02079	agaA	N-acetylgalactosamine-6-phosphate deacetylase [EC:3.5.1.25]	0.157	0.114	0.0938	0.0919	0.669	1.37	NA	NA	NA
PTS and Galactose	Transcripts/gene	NA	agaA	N-acetylgalactosamine-6-phosphate deacetylase [EC:3.5.1.25]	0.534	0.316	0.113	0.0625	0.0321	1.69	NA	NA	NA
PTS and Galactose	DNA	K01631	dgoA	2-dehydro-3-deoxyphosphogalactote aldolase [EC:4.1.2.21]	0.489	0.781	0.159	0.0973	0.00856	1.6	0.00568	0.0192	0.0142
PTS and Galactose	RNA	K01631	dgoA	2-dehydro-3-deoxyphosphogalactote aldolase [EC:4.1.2.21]	0.198	0.188	0.112	0.087	1	1.05	NA	NA	NA
PTS and Galactose	Transcripts/gene	NA	dgoA	2-dehydro-3-deoxyphosphogalactote aldolase [EC:4.1.2.21]	0.419	0.246	0.261	0.123	0.573	1.71	NA	NA	NA
PTS and Galactose	DNA	K01635	lacD	tagatose 1,6-diphosphate aldolase [EC:4.1.2.40]	4.39	2.15	1.67	0.767	0.0767	2.04	NA	NA	NA
PTS and Galactose	RNA	K01635	lacD	tagatose 1,6-diphosphate aldolase [EC:4.1.2.40]	0.324	0.228	0.235	0.196	0.519	1.42	NA	NA	NA
PTS and Galactose	DNA	K08302	gatY-kbaY	tagatose 1,6-diphosphate aldolase GatY/KbaY [EC:4.1.2.40]	12.6	9.64	2.28	2.2	0.504	1.3	NA	NA	NA
PTS and Galactose	RNA	K08302	gatY-kbaY	tagatose 1,6-diphosphate aldolase GatY/KbaY [EC:4.1.2.40]	6.66	3.84	1.35	0.741	0.00669	1.74	-0.0333	-0.0103	-0.0218
PTS and Galactose	Transcripts/gene	NA	gatY-kbaY	tagatose 1,6-diphosphate aldolase GatY/KbaY [EC:4.1.2.40]	0.429	0.365	0.149	0.119	0.686	1.18	NA	NA	NA
PTS and Galactose	DNA	K01684	dgoD	galactote dehydratase [EC:4.2.1.6]	15.5	21.3	3.69	3	0.204	1.37	NA	NA	NA
PTS and Galactose	RNA	K01684	dgoD	galactote dehydratase [EC:4.2.1.6]	5.2	4.12	1.24	1.06	0.283	1.26	NA	NA	NA
PTS and Galactose	Transcripts/gene	NA	dgoD	galactote dehydratase [EC:4.2.1.6]	0.35	0.193	0.107	0.0378	0.0321	1.81	NA	NA	NA
PTS and Galactose	DNA	K01784	galE	UDP-glucose 4-epimerase [EC:5.1.3.2]	558	571	49.8	19.9	1	1.02	NA	NA	NA
PTS and Galactose	RNA	K01784	galE	UDP-glucose 4-epimerase [EC:5.1.3.2]	509	458	47.1	48.1	0.23	1.11	NA	NA	NA
PTS and Galactose	Transcripts/gene	NA	galE	UDP-glucose 4-epimerase [EC:5.1.3.2]	0.917	0.802	0.111	0.0764	0.268	1.14	NA	NA	NA
PTS and Galactose	DNA	K01819	lacA, lacB	galactose-6-phosphate isomerase [EC:5.3.1.26]	18.6	18.8	3.11	4.57	1	1.01	NA	NA	NA
PTS and Galactose	RNA	K01819	lacA, lacB	galactose-6-phosphate isomerase [EC:5.3.1.26]	30.7	30.4	6.67	8.11	0.97	1.01	NA	NA	NA
PTS and Galactose	Transcripts/gene	NA	lacA, lacB	galactose-6-phosphate isomerase [EC:5.3.1.26]	1.68	1.73	0.376	0.734	0.958	1.03	NA	NA	NA
PTS and Galactose	DNA	K01835	pgm	phosphoglucomutase [EC:5.4.2.2]	133	135	19	36.3	0.989	1.01	NA	NA	NA
PTS and Galactose	RNA	K01835	pgm	phosphoglucomutase [EC:5.4.2.2]	280	328	84.9	207	0.895	1.17	NA	NA	NA
PTS and Galactose	Transcripts/gene	NA	pgm	phosphoglucomutase [EC:5.4.2.2]	2.1	2.36	0.587	1.07	0.905	1.12	NA	NA	NA
PTS and Galactose	DNA	K02080	agaI	galactosamine-6-phosphate isomerase [EC:5.3.1.-]	0.0895	0.0551	0.0298	0.0297	0.258	1.62	NA	NA	NA
PTS and Galactose	DNA	K02746	agaW	PTS system, N-acetylgalactosamine-specific IIC component	10.2	0.769	4.97	0.387	0.00856	13.3	-0.0117	-0.00506	-0.0079
PTS and Galactose	DNA	K02746	agaW	PTS system, N-acetylgalactosamine-specific IIC component	10.2	0.769	4.97	0.387	0.00856	13.3	-0.0117	-0.00506	-0.0079
PTS and Galactose	RNA	K02746	agaW	PTS system, N-acetylgalactosamine-specific IIC component	43.5	2.28	23.4	2.46	0.00669	19.1	-0.0302	-0.0148	-0.0239
PTS and Galactose	RNA	K02746	agaW	PTS system, N-acetylgalactosamine-specific IIC component	43.5	2.28	23.4	2.46	0.00669	19.1	-0.0302	-0.0148	-0.0239
PTS and Galactose	DNA	K02747	agaE	PTS system, N-acetylgalactosamine-specific IID component	12.1	0.846	6.02	0.401	0.00856	14.3	-0.0116	-0.00537	-0.00787
PTS and Galactose	DNA	K02747	agaE	PTS system, N-acetylgalactosamine-specific IID component	12.1	0.846	6.02	0.401	0.00856	14.3	-0.0116	-0.00537	-0.00787
PTS and Galactose	RNA	K02747	agaE	PTS system, N-acetylgalactosamine-specific IID component	53.7	2.93	38.6	3.44	0.00669	18.3	-0.0275	-0.0115	-0.0198
PTS and Galactose	RNA	K02747	agaE	PTS system, N-acetylgalactosamine-specific IID component	53.7	2.93	38.6	3.44	0.00669	18.3	-0.0275	-0.0115	-0.0198
PTS and Galactose	DNA	K02761	celB	PTS system, cellobiose-specific IIC component	97.8	27.8	35.2	8.85	0.0145	3.52	-0.0114	-0.00563	-0.00804
PTS and Galactose	RNA	K02761	celB	PTS system, cellobiose-specific IIC component	269	72.8	124	33.7	0.0191	3.7	-0.0232	-0.00335	-0.00978
PTS and Galactose	DNA	K02770	fruA	PTS system, fructose-specific IIC component	13.4	1.44	8.24	0.795	0.0145	9.31	-0.0109	-0.0038	-0.0072
PTS and Galactose	RNA	K02770	fruA	PTS system, fructose-specific IIC component	9.27	4.46	4.27	2.04	0.0819	2.08	-0.0242	-0.0057	-0.0157
PTS and Galactose	DNA	K02771	levG	PTS system, fructose-specific IID component	27.4	15.2	8.38	7.19	0.3	1.8	NA	NA	NA
PTS and Galactose	RNA	K02771	levG	PTS system, fructose-specific IID component	192	167	107	96.4	0.895	1.15	NA	NA	NA
PTS and Galactose	DNA	K02775	gatC	PTS system, galactitol-specific IIC component	120	113	29.2	25.6	0.989	1.06	NA	NA	NA
PTS and Galactose	DNA	K02775	gatC	PTS system, galactitol-specific IIC component	120	113	29.2	25.6	0.989	1.06	NA	NA	NA
PTS and Galactose	RNA	K02775	gatC	PTS system, galactitol-specific IIC component	163	141	44.1	37.5	0.623	1.15	NA	NA	NA
PTS and Galactose	RNA	K02775	gatC	PTS system, galactitol-specific IIC component	163	141	44.1	37.5	0.623	1.15	NA	NA	NA
PTS and Galactose	DNA	K02783	srlA	PTS system, glucitol/sorbitol-specific IIC component	11.7	15	6.16	9.47	0.968	1.28	NA	NA	NA
PTS and Galactose	RNA	K02783	srlA	PTS system, glucitol/sorbitol-specific IIC component	0.647	1.19	0.359	1.21	0.519	1.84	NA	NA	NA
PTS and Galactose	DNA	K02784	ptsH	phosphocarrier protein HPr	0.244	0.187	0.148	0.0745	1	1.3	NA	NA	NA

PTS and Galactose	RNA	K02784	ptsH	phosphocarrier protein HPr	0.536	0.405	0.526	0.225	0.97	1.32	NA	NA	NA
PTS and Galactose	DNA	K02788	lacE	PTS system, lactose-specific IIC component	0.0757	0.0424	0.0438	0.0316	0.62	1.78	NA	NA	NA
PTS and Galactose	DNA	K02788	lacE	PTS system, lactose-specific IIC component	0.0757	0.0424	0.0438	0.0316	0.62	1.78	NA	NA	NA
PTS and Galactose	RNA	K02788	lacE	PTS system, lactose-specific IIC component	0.0694	0.0297	0.106	0.0264	0.666	2.34	NA	NA	NA
PTS and Galactose	RNA	K02788	lacE	PTS system, lactose-specific IIC component	0.0694	0.0297	0.106	0.0264	0.666	2.34	NA	NA	NA
PTS and Galactose	DNA	K02795	manY	PTS system, mannose-specific IIC component	71	37.8	18.2	17.1	0.0993	1.88	-0.0108	-0.0024	-0.00598
PTS and Galactose	RNA	K02795	manY	PTS system, mannose-specific IIC component	595	314	207	149	0.102	1.9	-0.0239	-0.00343	-0.0128
PTS and Galactose	DNA	K02796	manZ	PTS system, mannose-specific IID component	50.7	25.3	13.1	12.1	0.0608	2.01	-0.0121	-0.0057	-0.00875
PTS and Galactose	RNA	K02796	manZ	PTS system, mannose-specific IID component	535	275	203	191	0.105	1.94	NA	NA	NA
PTS and Galactose	DNA	K02800	mtlA	PTS system, mannitol-specific IIC component	0.0203	0.0189	0.0152	0.0148	1	1.07	NA	NA	NA
PTS and Galactose	DNA	K02804	nagE	PTS system, N-acetylglucosamine-specific IIC component	0.217	0.4	0.154	0.628	1	1.84	NA	NA	NA
PTS and Galactose	RNA	K02804	nagE	PTS system, N-acetylglucosamine-specific IIC component	0.0814	0.515	0.0767	0.659	0.186	6.33	NA	NA	NA
PTS and Galactose	DNA	K02810	scrA	PTS system, sucrose-specific IIC component	0.00282	0.0035	0.00466	0.0033	0.989	1.24	NA	NA	NA
PTS and Galactose	DNA	K02814	sorA	PTS system, sorbose-specific IIC component	0.468	0.198	0.357	0.152	0.504	2.37	NA	NA	NA
PTS and Galactose	RNA	K02814	sorA	PTS system, sorbose-specific IIC component	2.64	1.34	2.09	1.04	0.471	1.97	NA	NA	NA
PTS and Galactose	DNA	K02815	sorM	PTS system, sorbose-specific IID component	0.16	0.0416	0.0985	0.0272	0.0408	3.85	-0.0172	-0.00317	-0.0116
PTS and Galactose	RNA	K02815	sorM	PTS system, sorbose-specific IID component	0.221	0.177	0.195	0.169	0.981	1.25	NA	NA	NA
PTS and Galactose	DNA	K03475	ulaA, sgaT	PTS system, ascorbate-specific IIC component	25	21.4	6.23	19.4	0.675	1.17	NA	NA	NA
PTS and Galactose	RNA	K03475	ulaA, sgaT	PTS system, ascorbate-specific IIC component	60.5	52.6	41.6	16.6	0.895	1.15	NA	NA	NA
PTS and Galactose	DNA	K08485	ptsO, npr	phosphocarrier protein NPr	0.0143	0.0171	0.00672	0.0133	1	1.2	NA	NA	NA
PTS and Galactose	DNA	K10985	agaC	PTS system, galactosamine-specific IIC component	1.51	0.787	1.09	0.516	0.675	1.92	NA	NA	NA
PTS and Galactose	DNA	K10985	agaC	PTS system, galactosamine-specific IIC component	1.51	0.787	1.09	0.516	0.675	1.92	NA	NA	NA
PTS and Galactose	RNA	K10985	agaC	PTS system, galactosamine-specific IIC component	12.6	6.57	11.5	8.49	0.31	1.92	NA	NA	NA
PTS and Galactose	RNA	K10985	agaC	PTS system, galactosamine-specific IIC component	12.6	6.57	11.5	8.49	0.31	1.92	NA	NA	NA
PTS and Galactose	DNA	K10986	agaD	PTS system, galactosamine-specific IID component	1.48	0.741	1.09	0.487	0.45	2	NA	NA	NA
PTS and Galactose	DNA	K10986	agaD	PTS system, galactosamine-specific IID component	1.48	0.741	1.09	0.487	0.45	2	NA	NA	NA
PTS and Galactose	RNA	K10986	agaD	PTS system, galactosamine-specific IID component	21.1	13.8	20.7	16.7	0.519	1.53	NA	NA	NA
PTS and Galactose	RNA	K10986	agaD	PTS system, galactosamine-specific IID component	21.1	13.8	20.7	16.7	0.519	1.53	NA	NA	NA
PTS and Galactose	DNA	K11196	levF	PTS system, fructose-specific IIC component	0.00398	0.0024	0.00287	0.0026	1	1.65	NA	NA	NA
PTS and Galactose	DNA	K12112	ebgC	evolved beta-galactosidase subunit beta	0.266	0.0065	0.257	0.0058	0.179	40.8	NA	NA	NA
Short chain fatty acid meta	Transcripts/gene	NA	adhE	acetaldehyde dehydrogenase / alcohol dehydrogenase [EC:1.2.1.10 1.1.1.1]	0.973	0.885	0.15	0.135	0.338	1.1	NA	NA	NA
Short chain fatty acid meta	DNA	K00074	paaH, hbd, fadB, mr	3-hydroxybutyryl-CoA dehydrogenase [EC:1.1.1.157]	95.1	68.8	20.7	17.1	0.131	1.38	NA	NA	NA
Short chain fatty acid meta	RNA	K00074	paaH, hbd, fadB, mr	3-hydroxybutyryl-CoA dehydrogenase [EC:1.1.1.157]	325	139	46.1	44.6	0.00669	2.33	-0.0288	-0.015	-0.024
Short chain fatty acid meta	Transcripts/gene	NA	paaH, hbd, fadB, mr	3-hydroxybutyryl-CoA dehydrogenase [EC:1.1.1.157]	3.56	2.06	0.933	0.496	0.0291	1.73	NA	NA	NA
Short chain fatty acid meta	DNA	K00011	AKR1	aldehyde reductase [EC:1.1.1.21]	20	18.2	5.73	3.38	1	1.1	NA	NA	NA
Short chain fatty acid meta	RNA	K00011	AKR2	aldehyde reductase [EC:1.1.1.21]	8.61	15.1	6.55	7.18	0.283	1.75	NA	NA	NA
Short chain fatty acid meta	Transcripts/gene	NA	AKR4	aldehyde reductase [EC:1.1.1.21]	0.469	0.825	0.354	0.372	0.183	1.76	NA	NA	NA
Short chain fatty acid meta	Transcripts/gene	NA	HADHA	enoyl-CoA hydratase / long-chain 3-hydroxyacyl-CoA dehydrogenase [EC:4.2.1.17 1.1.1.211]	8.31	7.99	1.28	6.24	0.5	1.04	NA	NA	NA
Short chain fatty acid meta	Transcripts/gene	NA	HADHA	enoyl-CoA hydratase / long-chain 3-hydroxyacyl-CoA dehydrogenase [EC:4.2.1.17 1.1.1.211]	8.31	7.99	1.28	6.24	0.577	1.04	NA	NA	NA
Short chain fatty acid meta	DNA	K00016	ldh	L-lactate dehydrogenase [EC:1.1.1.27]	92.8	62.6	21.2	13.4	0.0359	1.48	-0.0102	-0.00375	-0.00691
Short chain fatty acid meta	RNA	K00016	ldh	L-lactate dehydrogenase [EC:1.1.1.27]	76.6	193	14.9	259	0.519	2.52	NA	NA	NA
Short chain fatty acid meta	Transcripts/gene	NA	ldh	L-lactate dehydrogenase [EC:1.1.1.27]	0.842	2.71	0.162	3.15	0.249	3.23	NA	NA	NA
Short chain fatty acid meta	DNA	K03777	dld	D-lactate dehydrogenase [EC:1.1.1.28]	0.2	0.148	0.152	0.0673	1	1.35	NA	NA	NA
Short chain fatty acid meta	RNA	K03777	dld	D-lactate dehydrogenase [EC:1.1.1.28]	0.167	0.105	0.176	0.106	0.736	1.58	NA	NA	NA
Short chain fatty acid meta	DNA	K03778	ldhA	D-lactate dehydrogenase [EC:1.1.1.28]	73.1	63.3	12.9	8.7	0.675	1.15	NA	NA	NA
Short chain fatty acid meta	RNA	K03778	ldhA	D-lactate dehydrogenase [EC:1.1.1.28]	266	180	104	83.7	0.254	1.48	NA	NA	NA
Short chain fatty acid meta	Transcripts/gene	NA	ldhA	D-lactate dehydrogenase [EC:1.1.1.28]	3.68	2.82	1.5	1.24	0.409	1.3	NA	NA	NA
Short chain fatty acid meta	Transcripts/gene	NA	mcr	malonyl-CoA reductase / 3-hydroxypropionate dehydrogenase (NADP+) [EC:1.2.1.75 1.1.1.298]	0.281	0.0914	0.132	0.0888	0.114	3.07	NA	NA	NA
Short chain fatty acid meta	DNA	K00019	bdh	3-hydroxybutyrate dehydrogenase [EC:1.1.1.30]	6.87	5.67	1.58	0.428	0.127	1.21	-0.0139	-0.00723	-0.0106
Short chain fatty acid meta	RNA	K00019	bdh	3-hydroxybutyrate dehydrogenase [EC:1.1.1.30]	2.84	2.81	0.736	0.677	0.895	1.01	NA	NA	NA
Short chain fatty acid meta	Transcripts/gene	NA	bdh	3-hydroxybutyrate dehydrogenase [EC:1.1.1.30]	0.435	0.494	0.159	0.111	0.625	1.14	NA	NA	NA
Short chain fatty acid meta	DNA	K00004	butB	(R,R)-butanediol dehydrogenase / meso-butanediol dehydrogenase / diacetyl reductase [EC:1.1.1.4 1.1.1.- 1.1.1.303]	1.47	1.28	0.338	0.454	0.938	1.15	NA	NA	NA
Short chain fatty acid meta	DNA	K03366	butA, budC	meso-butanediol dehydrogenase / (S,S)-butanediol dehydrogenase / diacetyl reductase [EC:1.1.1.- 1.1.1.76 1.1.1.304]	1.83	2.11	0.508	0.552	0.875	1.15	NA	NA	NA
Short chain fatty acid meta	Transcripts/gene	NA	butA, budC	meso-butanediol dehydrogenase / (S,S)-butanediol dehydrogenase / diacetyl reductase [EC:1.1.1.- 1.1.1.76 1.1.1.304]	1.22	0.447	0.617	0.0847	0.0211	2.73	NA	NA	NA
Short chain fatty acid meta	DNA	K00022	HADH	3-hydroxyacyl-CoA dehydrogenase [EC:1.1.1.35]	0.291	0.256	0.172	0.207	0.968	1.14	NA	NA	NA
Short chain fatty acid meta	RNA	K00022	HADH	3-hydroxyacyl-CoA dehydrogenase [EC:1.1.1.35]	0.205	0.185	0.0625	0.0672	0.574	1.11	NA	NA	NA
Short chain fatty acid meta	Transcripts/gene	NA	HADH	enoyl-CoA hydratase / 3-hydroxyacyl-CoA dehydrogenase / 3,2-trans-enoyl-CoA isomerase [EC:4.2.1.17 1.1.1.35 5.3.3.8]	0.533	0.489	0.147	0.237	0.43	1.09	NA	NA	NA
Short chain fatty acid meta	Transcripts/gene	NA	HADH	enoyl-CoA hydratase / 3-hydroxyacyl-CoA dehydrogenase / 3,2-trans-enoyl-CoA isomerase [EC:4.2.1.17 1.1.1.35 5.3.3.8]	0.533	0.489	0.147	0.237	0.45	1.09	NA	NA	NA
Short chain fatty acid meta	DNA	K01782	fadJ	3-hydroxyacyl-CoA dehydrogenase / enoyl-CoA hydratase / 3-hydroxybutyryl-CoA epimerase [EC:1.1.1.35 4.2.1.17 5.1.2.3]	0.388	0.283	0.219	0.0651	0.989	1.37	NA	NA	NA
Short chain fatty acid meta	RNA	K01782	fadJ	3-hydroxyacyl-CoA dehydrogenase / enoyl-CoA hydratase / 3-hydroxybutyryl-CoA epimerase [EC:1.1.1.35 4.2.1.17 5.1.2.3]	0.411	0.322	0.106	0.206	0.205	1.28	NA	NA	NA
Short chain fatty acid meta	DNA	K01825	fadB	3-hydroxyacyl-CoA dehydrogenase / enoyl-CoA hydratase / 3-hydroxybutyryl-CoA epimerase / enoyl-CoA isomerase [EC:1.1.1.35 4.2.1.17 5.1.2.3 5.3.3.	0.194	0.084	0.198	0.0354	0.779	2.31	NA	NA	NA
Short chain fatty acid meta	RNA	K01825	fadB	3-hydroxyacyl-CoA dehydrogenase / enoyl-CoA hydratase / 3-hydroxybutyryl-CoA epimerase / enoyl-CoA isomerase [EC:1.1.1.35 4.2.1.17 5.1.2.3 5.3.3.	0.487	0.304	0.287	0.219	0.381	1.6	NA	NA	NA
Short chain fatty acid meta	DNA	K00023	phbB	acetoacetyl-CoA reductase [EC:1.1.1.36]	3.34	3.18	0.818	0.546	1	1.05	NA	NA	NA
Short chain fatty acid meta	RNA	K00023	phbB	acetoacetyl-CoA reductase [EC:1.1.1.36]	1.61	1.09	0.764	0.18	0.254	1.47	NA	NA	NA
Short chain fatty acid meta	Transcripts/gene	NA	phbB	(R,R)-butanediol dehydrogenase / meso-butanediol dehydrogenase / diacetyl reductase [EC:1.1.1.4 1.1.1.- 1.1.1.303]	0.516	0.349	0.288	0.0628	0.45	1.48	NA	NA	NA
Short chain fatty acid meta	DNA	K00024	mdh	malate dehydrogenase [EC:1.1.1.37]	163	152	12.1	14	0.504	1.07	NA	NA	NA
Short chain fatty acid meta	RNA	K00024	mdh	malate dehydrogenase [EC:1.1.1.37]	813	806	87.1	152	0.944	1.01	NA	NA	NA
Short chain fatty acid meta	DNA	K00025	MDH1	malate dehydrogenase [EC:1.1.1.37]	0.757	0.761	0.461	0.262	1	1.01	NA	NA	NA
Short chain fatty acid meta	RNA	K00025	MDH1	malate dehydrogenase [EC:1.1.1.37]	2.54	1.23	3.81	0.844	0.623	2.07	NA	NA	NA
Short chain fatty acid meta	DNA	K00026	MDH2	malate dehydrogenase [EC:1.1.1.37]	0.0299	0.0724	0.0195	0.0834	0.779	2.42	NA	NA	NA
Short chain fatty acid meta	RNA	K00026	MDH2	malate dehydrogenase [EC:1.1.1.37]	2.7	1.99	0.918	0.968	0.382	1.36	NA	NA	NA
Short chain fatty acid meta	Transcripts/gene	NA	MDH2	malate dehydrogenase [EC:1.1.1.37]	5.02	5.28	0.665	0.918	0.597	1.05	NA	NA	NA
Short chain fatty acid meta	DNA	K00027	sfcA, maeA	malate dehydrogenase (oxaloacetate-decarboxylating) [EC:1.1.1.38]	166	165	22.2	32.8	1	1.01	NA	NA	NA
Short chain fatty acid meta	RNA	K00027	sfcA, maeA	malate dehydrogenase (oxaloacetate-decarboxylating) [EC:1.1.1.38]	220	267	83.5	114	0.519	1.21	NA	NA	NA
Short chain fatty acid meta	Transcripts/gene	NA	sfcA, maeA	malate dehydrogenase (oxaloacetate-decarboxylating) [EC:1.1.1.38]	1.35	1.62	0.547	0.546	0.558	1.2	NA	NA	NA
Short chain fatty acid meta	DNA	K00028	NA	malate dehydrogenase (decarboxylating) [EC:1.1.1.39]	0.465	0.52	0.23	0.33	1	1.12	NA	NA	NA
Short chain fatty acid meta	RNA	K00028	NA	malate dehydrogenase (decarboxylating) [EC:1.1.1.39]	1.21	1.65	0.589	0.502	0.256	1.36	NA	NA	NA
Short chain fatty acid meta	Transcripts/gene	NA	NA	malate dehydrogenase (decarboxylating) [EC:1.1.1.39]	3.35	3.88	2.2	1.88	0.786	1.16	NA	NA	NA
Short chain fatty acid meta	RNA	K00004	butB	(R,R)-butanediol dehydrogenase / meso-butanediol dehydrogenase / diacetyl reductase [EC:1.1.1.4 1.1.1.- 1.1.1.303]	2.72	0.934	1.45	0.395	0.0326	2.91	-0.0247	-0.00766	-0.0158
Short chain fatty acid meta	DNA	K03366	butA, budC	meso-butanediol dehydrogenase / (S,S)-butanediol dehydrogenase / diacetyl reductase [EC:1.1.1.- 1.1.1.76 1.1.1.304]	1.83	2.11	0.508	0.552	0.875	1.15	NA	NA	NA
Short chain fatty acid meta	RNA	K03366	butA, budC	meso-butanediol dehydrogenase / (S,S)-butanediol dehydrogenase / diacetyl reductase [EC:1.1.1.- 1.1.1.76 1.1.1.304]	1.59	0.575	2.03	0.215	0.519	2.76	NA	NA	NA
Short chain fatty acid meta	Transcripts/gene	NA	butA, budC	meso-butanediol dehydrogenase / (S,S)-butanediol dehydrogenase / diacetyl reductase [EC:1.1.1.- 1.1.1.76 1.1.1.304]	1.22	0.447	0.617	0.0847	0.0211	2.73	NA	NA	NA
Short chain fatty acid meta	DNA	K00029	maeB	malate dehydrogenase (oxaloacetate-decarboxylating)(NADP+) [EC:1.1.1.40]	175	177	31.3	33.3	1	1.01	NA	NA	NA
Short chain fatty acid meta	RNA	K00029	maeB	malate dehydrogenase (oxaloacetate-decarboxylating)(NADP+) [EC:1.1.1.40]	268	203	39.1	35.2	0.0743	1.32	NA	NA	NA

Short chain fatty acid meta Transcripts/gene	NA	maeB	malate dehydrogenase (oxaloacetate-decarboxylating)(NADP+) [EC:1.1.1.40]	1.59	1.16	0.403	0.152	0.183	1.38	NA	NA	NA
Short chain fatty acid meta DNA	K00043	gbd	4-hydroxybutyrate dehydrogenase [EC:1.1.1.61]	10.4	5.21	5.31	1.52	0.133	1.99	NA	NA	NA
Short chain fatty acid meta RNA	K00043	gbd	4-hydroxybutyrate dehydrogenase [EC:1.1.1.61]	6.03	1.4	4.82	0.742	0.0326	4.3	NA	NA	NA
Short chain fatty acid meta Transcripts/gene	NA	gbd	4-hydroxybutyrate dehydrogenase [EC:1.1.1.61]	0.623	0.257	0.44	0.0809	0.175	2.43	NA	NA	NA
Short chain fatty acid meta DNA	K00048	fucO	lactaldehyde reductase [EC:1.1.1.77]	102	101	12.8	20.8	1	1.02	NA	NA	NA
Short chain fatty acid meta RNA	K00048	fucO	lactaldehyde reductase [EC:1.1.1.77]	310	274	79.3	36.3	0.623	1.13	NA	NA	NA
Short chain fatty acid meta Transcripts/gene	NA	fucO	lactaldehyde reductase [EC:1.1.1.77]	3.04	2.77	0.714	0.328	0.708	1.09	NA	NA	NA
Short chain fatty acid meta Transcripts/gene	NA	ghrA	glyoxylate/hydroxypyruvate reductase [EC:1.1.1.79 1.1.1.81]	0.289	0.186	0.112	0.0529	0.183	1.56	NA	NA	NA
Short chain fatty acid meta DNA	K00049	GRHPR	glyoxylate/hydroxypyruvate reductase [EC:1.1.1.79 1.1.1.81]	0.0787	0.0436	0.0335	0.0134	0.298	1.8	NA	NA	NA
Short chain fatty acid meta DNA	K12972	ghrA	gyoxylate/hydroxypyruvate reductase A [EC:1.1.1.79 1.1.1.81]	6.24	5.8	1.35	2.12	0.968	1.08	NA	NA	NA
Short chain fatty acid meta RNA	K12972	ghrA	gyoxylate/hydroxypyruvate reductase A [EC:1.1.1.79 1.1.1.81]	1.73	1.01	0.476	2.12	0.0252	1.72	NA	NA	NA
Short chain fatty acid meta Transcripts/gene	NA	ghrA	gyoxylate/hydroxypyruvate reductase A [EC:1.1.1.79 1.1.1.81]	0.976	0.391	0.798	0.128	0.163	2.5	NA	NA	NA
Short chain fatty acid meta DNA	K00051	NA	malate dehydrogenase (NADP+) [EC:1.1.1.82]	0.192	0.23	0.111	0.095	0.675	1.2	NA	NA	NA
Short chain fatty acid meta RNA	K00051	NA	malate dehydrogenase (NADP+) [EC:1.1.1.82]	0.0455	0.0746	0.0494	0.0725	0.676	1.64	NA	NA	NA
Short chain fatty acid meta Transcripts/gene	NA	NA	malate dehydrogenase (NADP+) [EC:1.1.1.82]	0.292	0.337	0.284	0.302	0.786	1.15	NA	NA	NA
Short chain fatty acid meta Transcripts/gene	NA	NA	tartrate dehydrogenase/decarboxylase / D-malate dehydrogenase [EC:1.1.1.93 4.1.1.73 1.1.1.83]	0.3	0.203	0.134	0.1	0.355	1.47	NA	NA	NA
Short chain fatty acid meta DNA	K00101	lldD	L-lactate dehydrogenase (cytochrome) [EC:1.1.2.3]	4.5	4.3	0.866	1.21	1	1.05	NA	NA	NA
Short chain fatty acid meta RNA	K00101	lldD	L-lactate dehydrogenase (cytochrome) [EC:1.1.2.3]	2.39	1.68	0.738	0.593	0.204	1.43	NA	NA	NA
Short chain fatty acid meta Transcripts/gene	NA	lldD	L-lactate dehydrogenase (cytochrome) [EC:1.1.2.3]	0.557	0.4	0.208	0.138	0.249	1.39	NA	NA	NA
Short chain fatty acid meta DNA	K00102	dld, LDHD	D-lactate dehydrogenase (cytochrome) [EC:1.1.2.4]	25	27.4	11.2	3.71	0.911	1.1	NA	NA	NA
Short chain fatty acid meta RNA	K00102	dld, LDHD	D-lactate dehydrogenase (cytochrome) [EC:1.1.2.4]	10.6	10.2	4.51	2.31	0.944	1.03	NA	NA	NA
Short chain fatty acid meta Transcripts/gene	NA	dld, LDHD	D-lactate dehydrogenase (cytochrome) [EC:1.1.2.4]	0.509	0.372	0.429	0.0617	0.908	1.37	NA	NA	NA
Short chain fatty acid meta DNA	K00114	exaA	alcohol dehydrogenase (cytochrome c) [EC:1.1.2.8]	0.0617	0.0341	0.0273	0.0245	0.394	1.81	NA	NA	NA
Short chain fatty acid meta DNA	K00116	mqo	malate dehydrogenase (quinone) [EC:1.1.5.4]	0.226	0.148	0.165	0.0504	0.99	1.53	NA	NA	NA
Short chain fatty acid meta RNA	K00116	mqo	malate dehydrogenase (quinone) [EC:1.1.5.4]	0.196	0.0776	0.199	0.0283	0.229	2.53	NA	NA	NA
Short chain fatty acid meta Transcripts/gene	NA	mqo	malate dehydrogenase (quinone) [EC:1.1.5.4]	0.872	0.556	0.591	0.19	0.379	1.57	NA	NA	NA
Short chain fatty acid meta DNA	K00109	L2HDGD	2-hydroxyglutarate dehydrogenase [EC:1.1.99.2]	0.261	0.123	0.117	0.0393	0.11	2.13	NA	NA	NA
Short chain fatty acid meta RNA	K00109	L2HDGD	2-hydroxyglutarate dehydrogenase [EC:1.1.99.2]	0.0749	0.0668	0.113	0.0527	0.676	1.12	NA	NA	NA
Short chain fatty acid meta Transcripts/gene	NA	L2HDGD	2-hydroxyglutarate dehydrogenase [EC:1.1.99.2]	0.439	0.517	0.668	0.248	0.355	1.18	NA	NA	NA
Short chain fatty acid meta DNA	K00467	NA	lactate 2-monooxygenase [EC:1.13.12.4]	1.27	0.184	0.951	0.0774	0.00856	6.89	NA	NA	NA
Short chain fatty acid meta RNA	K00467	NA	lactate 2-monooxygenase [EC:1.13.12.4]	0.0549	0.051	0.0566	0.0538	0.901	1.08	NA	NA	NA
Short chain fatty acid meta Transcripts/gene	NA	NA	lactate 2-monooxygenase [EC:1.13.12.4]	0.0678	0.376	0.0938	0.515	0.543	5.54	NA	NA	NA
Short chain fatty acid meta DNA	K00132	NA	acetaldehyde dehydrogenase (acetylating) [EC:1.2.1.10]	6.69	5.34	1.14	1.79	0.62	1.25	NA	NA	NA
Short chain fatty acid meta RNA	K00132	NA	acetaldehyde dehydrogenase (acetylating) [EC:1.2.1.10]	10.1	8.28	3.72	3.06	0.572	1.23	NA	NA	NA
Short chain fatty acid meta DNA	K04073	mhpF	acetaldehyde dehydrogenase [EC:1.2.1.10]	0.782	0.901	0.243	0.25	0.968	1.15	NA	NA	NA
Short chain fatty acid meta RNA	K04073	mhpF	acetaldehyde dehydrogenase [EC:1.2.1.10]	0.487	0.567	0.463	0.294	0.518	1.16	NA	NA	NA
Short chain fatty acid meta Transcripts/gene	NA	mhpF	acetaldehyde dehydrogenase [EC:1.2.1.10]	1.2	0.925	0.138	0.191	0.163	1.3	NA	NA	NA
Short chain fatty acid meta DNA	K04072	adhE	acetaldehyde dehydrogenase / alcohol dehydrogenase [EC:1.2.1.10 1.1.1.1]	194	192	21.4	32.8	1	1.01	NA	NA	NA
Short chain fatty acid meta RNA	K04072	adhE	acetaldehyde dehydrogenase / alcohol dehydrogenase [EC:1.2.1.10 1.1.1.1]	232	172	50.1	32.8	0.162	1.35	NA	NA	NA
Short chain fatty acid meta DNA	K00135	gabD	succinate-semialdehyde dehydrogenase / glutarate-semialdehyde dehydrogenase [EC:1.2.1.16 1.2.1.79 1.2.1.20]	5.49	5.92	1.08	1.43	1	1.08	NA	NA	NA
Short chain fatty acid meta RNA	K00135	gabD	succinate-semialdehyde dehydrogenase / glutarate-semialdehyde dehydrogenase [EC:1.2.1.16 1.2.1.79 1.2.1.20]	2.44	2.62	0.753	0.888	0.944	1.08	NA	NA	NA
Short chain fatty acid meta Transcripts/gene	NA	gabD	succinate-semialdehyde dehydrogenase / glutarate-semialdehyde dehydrogenase [EC:1.2.1.16 1.2.1.79 1.2.1.20]	0.446	0.45	0.116	0.125	0.958	1.01	NA	NA	NA
Short chain fatty acid meta Transcripts/gene	NA	mmsA, iolA, ALDH	malonate-semialdehyde dehydrogenase (acetylating) / methylmalonate-semialdehyde dehydrogenase [EC:1.2.1.18 1.2.1.27]	2.03	1.25	0.95	0.284	0.336	1.63	NA	NA	NA
Short chain fatty acid meta DNA	K00140	mmsA, iolA, ALDH	malonate-semialdehyde dehydrogenase (acetylating) / methylmalonate-semialdehyde dehydrogenase [EC:1.2.1.18 1.2.1.27]	11	6.93	4.15	1.81	0.185	1.58	NA	NA	NA
Short chain fatty acid meta RNA	K00140	mmsA, iolA, ALDH	malonate-semialdehyde dehydrogenase (acetylating) / methylmalonate-semialdehyde dehydrogenase [EC:1.2.1.18 1.2.1.27]	19.8	8.43	5.95	2.18	0.00669	2.34	NA	NA	NA
Short chain fatty acid meta Transcripts/gene	NA	aldA	lactaldehyde dehydrogenase / glycolaldehyde dehydrogenase [EC:1.2.1.22 1.2.1.21]	3.19	2.9	2.33	0.663	0.597	1.1	NA	NA	NA
Short chain fatty acid meta Transcripts/gene	NA	aldA	lactaldehyde dehydrogenase / glycolaldehyde dehydrogenase [EC:1.2.1.22 1.2.1.21]	3.19	2.9	2.33	0.663	0.597	1.1	NA	NA	NA
Short chain fatty acid meta DNA	K07248	aldA	lactaldehyde dehydrogenase / glycolaldehyde dehydrogenase [EC:1.2.1.22 1.2.1.21]	70.4	79.2	30.1	21.2	1	1.12	NA	NA	NA
Short chain fatty acid meta RNA	K07248	aldA	lactaldehyde dehydrogenase / glycolaldehyde dehydrogenase [EC:1.2.1.22 1.2.1.21]	192	234	76.5	21.2	0.676	1.22	NA	NA	NA
Short chain fatty acid meta DNA	K00139	ALDH5A1	succinate-semialdehyde dehydrogenase [EC:1.2.1.24]	0.0298	0.0257	0.0254	0.0059	0.989	1.16	NA	NA	NA
Short chain fatty acid meta RNA	K00139	ALDH5A1	succinate-semialdehyde dehydrogenase [EC:1.2.1.24]	0.0356	0.0335	0.0483	0.0314	0.944	1.06	NA	NA	NA
Short chain fatty acid meta Transcripts/gene	NA	ALDH5A1	succinate-semialdehyde dehydrogenase [EC:1.2.1.24]	1.85	1.37	3.23	1.33	0.895	1.35	NA	NA	NA
Short chain fatty acid meta Transcripts/gene	NA	mmsA, iolA, ALDH	malonate-semialdehyde dehydrogenase (acetylating) / methylmalonate-semialdehyde dehydrogenase [EC:1.2.1.18 1.2.1.27]	2.03	1.25	0.95	0.284	0.336	1.63	NA	NA	NA
Short chain fatty acid meta DNA	K00128	ALDH3A2	aldehyde dehydrogenase (NAD+) [EC:1.2.1.3]	109	74.5	17.3	16.1	0.0484	1.46	-0.0118	-0.0026	-0.00727
Short chain fatty acid meta RNA	K00128	ALDH3A2	aldehyde dehydrogenase (NAD+) [EC:1.2.1.3]	70.7	40.3	38.4	13.3	0.205	1.75	NA	NA	NA
Short chain fatty acid meta Transcripts/gene	NA	ALDH3A2	aldehyde dehydrogenase (NAD+) [EC:1.2.1.3]	0.671	0.548	0.391	0.147	0.84	1.22	NA	NA	NA
Short chain fatty acid meta Transcripts/gene	NA	ALDH7A1	aldehyde dehydrogenase family 7 member A1 [EC:1.2.1.31 1.2.1.8 1.2.1.3]	0.229	0.34	0.143	0.147	0.323	1.48	NA	NA	NA
Short chain fatty acid meta RNA	K14085	ALDH7A1	aldehyde dehydrogenase family 7 member A1 [EC:1.2.1.31 1.2.1.8 1.2.1.3]	0.0592	0.0875	0.0591	0.0726	0.853	1.48	NA	NA	NA
Short chain fatty acid meta Transcripts/gene	NA	ALDH9A1	aldehyde dehydrogenase family 9 member A1 [EC:1.2.1.47 1.2.1.3]	2.73	0.575	2.25	0.518	0.184	4.76	NA	NA	NA
Short chain fatty acid meta DNA	K00149	ALDH9A1	aldehyde dehydrogenase family 9 member A1 [EC:1.2.1.47 1.2.1.3]	0.0422	0.031	0.0341	0.0156	1	1.36	NA	NA	NA
Short chain fatty acid meta RNA	K00149	ALDH9A1	aldehyde dehydrogenase family 9 member A1 [EC:1.2.1.47 1.2.1.3]	0.0812	0.0203	0.0444	0.0156	0.107	4	NA	NA	NA
Short chain fatty acid meta DNA	K14468	mcr	malonyl-CoA reductase / 3-hydroxypropionate dehydrogenase (NADP+) [EC:1.2.1.75 1.1.1.298]	0.0235	0.0283	0.0123	0.0135	0.989	1.21	NA	NA	NA
Short chain fatty acid meta Transcripts/gene	NA	ALDH7A1	aldehyde dehydrogenase family 7 member A1 [EC:1.2.1.31 1.2.1.8 1.2.1.3]	1.76	1.46	1.86	0.498	0.786	1.2	NA	NA	NA
Short chain fatty acid meta Transcripts/gene	NA	ALDH7A1	aldehyde dehydrogenase family 7 member A1 [EC:1.2.1.31 1.2.1.8 1.2.1.3]	1.76	1.46	1.86	0.498	0.832	1.2	NA	NA	NA
Short chain fatty acid meta DNA	K00158	poxL	pyruvate oxidase [EC:1.2.3.3]	5.55	3.14	1.68	0.746	0.107	1.77	-0.0102	-0.00272	-0.00564
Short chain fatty acid meta RNA	K00158	poxL	pyruvate oxidase [EC:1.2.3.3]	24.9	4.44	14	1.84	0.00669	5.6	-0.0197	-0.0101	-0.0143
Short chain fatty acid meta Transcripts/gene	NA	poxL	pyruvate oxidase [EC:1.2.3.3]	4.52	1.46	1.88	0.587	0.105	3.1	NA	NA	NA
Short chain fatty acid meta DNA	K00161	pdhA	pyruvate dehydrogenase E1 component subunit alpha [EC:1.2.4.1]	7.9	8.66	2.73	1.97	0.911	1.1	NA	NA	NA
Short chain fatty acid meta RNA	K00161	pdhA	pyruvate dehydrogenase E1 component subunit alpha [EC:1.2.4.1]	19.7	24.6	13	11.2	0.676	1.25	NA	NA	NA
Short chain fatty acid meta DNA	K00162	pdhB	pyruvate dehydrogenase E1 component subunit beta [EC:1.2.4.1]	9.54	9.86	2.55	2.38	1	1.03	NA	NA	NA
Short chain fatty acid meta RNA	K00162	pdhB	pyruvate dehydrogenase E1 component subunit beta [EC:1.2.4.1]	23.3	27.7	13.9	14.2	0.791	1.19	NA	NA	NA
Short chain fatty acid meta DNA	K00163	aceE	pyruvate dehydrogenase E1 component [EC:1.2.4.1]	0.415	0.311	0.329	0.0809	1	1.33	NA	NA	NA
Short chain fatty acid meta RNA	K00163	aceE	pyruvate dehydrogenase E1 component [EC:1.2.4.1]	1.58	1.02	1.62	0.91	0.789	1.55	NA	NA	NA
Short chain fatty acid meta Transcripts/gene	NA	aceE	pyruvate dehydrogenase E1 component [EC:1.2.4.1]	2.79	2.72	1.98	0.869	0.764	1.03	NA	NA	NA
Short chain fatty acid meta DNA	K00156	poxB	pyruvate dehydrogenase (quinone) [EC:1.2.5.1]	0.166	0.215	0.16	0.122	0.683	1.3	NA	NA	NA
Short chain fatty acid meta RNA	K00156	poxB	pyruvate dehydrogenase (quinone) [EC:1.2.5.1]	0.0697	0.076	0.0764	0.0654	0.895	1.09	NA	NA	NA
Short chain fatty acid meta Transcripts/gene	NA	poxB	pyruvate dehydrogenase (quinone) [EC:1.2.5.1]	0.544	0.448	0.57	0.51	0.786	1.21	NA	NA	NA
Short chain fatty acid meta DNA	K00169	porA	pyruvate ferredoxin oxidoreductase, alpha subunit [EC:1.2.7.1]	41.2	45.4	10.8	9.89	1	1.1	NA	NA	NA
Short chain fatty acid meta RNA	K00169	porA	pyruvate ferredoxin oxidoreductase, alpha subunit [EC:1.2.7.1]	32.3	37.4	13.1	8.89	0.424	1.16	NA	NA	NA
Short chain fatty acid meta DNA	K00170	porB	pyruvate ferredoxin oxidoreductase, beta subunit [EC:1.2.7.1]	34.5	37.8	8.76	8.23	0.989	1.1	NA	NA	NA
Short chain fatty acid meta RNA	K00170	porB	pyruvate ferredoxin oxidoreductase, beta subunit [EC:1.2.7.1]	28.1	34.3	13	8.94	0.519	1.22	NA	NA	NA
Short chain fatty acid meta DNA	K00171	porD	pyruvate ferredoxin oxidoreductase, delta subunit [EC:1.2.7.1]	11	11.9	2.69	2.6	1	1.08	NA	NA	NA
Short chain fatty acid meta RNA	K00171	porD	pyruvate ferredoxin oxidoreductase, delta subunit [EC:1.2.7.1]	6.73	6.97	2.33	2.04	1	1.03	NA	NA	NA
Short chain fatty acid meta DNA	K00172	porG	pyruvate ferredoxin oxidoreductase, gamma subunit [EC:1.2.7.1]	22	23.7	6.23	5.16	0.989	1.08	NA	NA	NA

Short chain fatty acid meta RNA	K00172	porG	pyruvate ferredoxin oxidoreductase, gamma subunit [EC:1.2.7.1]	14.4	19.3	8.49	4.55	0.223	1.34	NA	NA	NA
Short chain fatty acid meta Transcripts/gene	NA	porG	pyruvate ferredoxin oxidoreductase, gamma subunit [EC:1.2.7.1]	0.755	0.858	0.265	0.253	0.558	1.14	NA	NA	NA
Short chain fatty acid meta DNA	K10783	NA	NA	8.93	5.42	5.72	1.54	0.676	1.65	NA	NA	NA
Short chain fatty acid meta RNA	K10783	NA	NA	6.28	4.78	3.96	3.28	0.472	1.31	NA	NA	NA
Short chain fatty acid meta Transcripts/gene	NA	NA	NA	0.846	0.868	0.6	0.495	0.895	1.03	NA	NA	NA
Short chain fatty acid meta DNA	K00248	bcd	butyryl-CoA dehydrogenase [EC:1.3.8.1]	77.6	75.7	14.6	20.1	1	1.03	NA	NA	NA
Short chain fatty acid meta RNA	K00248	bcd	butyryl-CoA dehydrogenase [EC:1.3.8.1]	347	179	87	58.7	0.0374	1.94	-0.02	-0.00457	-0.0117
Short chain fatty acid meta Transcripts/gene	NA	bcd	butyryl-CoA dehydrogenase [EC:1.3.8.1]	4.65	2.46	1.61	0.747	0.0346	1.89	NA	NA	NA
Short chain fatty acid meta DNA	K00249	acd	acyl-CoA dehydrogenase [EC:1.3.8.7]	6.35	4.75	2.52	1.24	0.675	1.34	NA	NA	NA
Short chain fatty acid meta RNA	K00249	acd	acyl-CoA dehydrogenase [EC:1.3.8.7]	23.3	10.1	7.29	3.35	0.00669	2.31	-0.029	-0.0104	-0.0239
Short chain fatty acid meta Transcripts/gene	NA	acd	acyl-CoA dehydrogenase [EC:1.3.8.7]	4.04	2.13	1.25	0.527	0.0796	1.89	NA	NA	NA
Short chain fatty acid meta DNA	K00239	sdhA, frdA	succinate dehydrogenase flavoprotein subunit [EC:1.3.5.1]	360	347	39	24.3	0.875	1.04	NA	NA	NA
Short chain fatty acid meta RNA	K00239	sdhA, frdA	succinate dehydrogenase flavoprotein subunit [EC:1.3.5.1]	1160	1060	197	189	0.623	1.1	NA	NA	NA
Short chain fatty acid meta DNA	K00240	sdhB, frdB	succinate dehydrogenase iron-sulfur subunit [EC:1.3.5.1]	139	135	15	11.7	0.989	1.03	NA	NA	NA
Short chain fatty acid meta RNA	K00240	sdhB, frdB	succinate dehydrogenase iron-sulfur subunit [EC:1.3.5.1]	419	373	65.6	62.5	0.344	1.12	NA	NA	NA
Short chain fatty acid meta DNA	K00244	frdA	fumarate reductase flavoprotein subunit [EC:1.3.5.4]	154	118	29.8	49.7	0.675	1.31	NA	NA	NA
Short chain fatty acid meta RNA	K00244	frdA	fumarate reductase flavoprotein subunit [EC:1.3.5.4]	141	90.9	49.6	21.2	0.154	1.55	NA	NA	NA
Short chain fatty acid meta DNA	K00245	frdB	fumarate reductase iron-sulfur subunit [EC:1.3.5.4]	14.9	12.8	2.36	3.49	0.779	1.16	NA	NA	NA
Short chain fatty acid meta RNA	K00245	frdB	fumarate reductase iron-sulfur subunit [EC:1.3.5.4]	4.89	4.26	1.5	1.21	0.677	1.15	NA	NA	NA
Short chain fatty acid meta Transcripts/gene	NA	frdA, frdB	fumarate reductase iron-sulfur subunit [EC:1.3.5.4]	2.62	2.51	0.521	0.456	0.868	1.04	NA	NA	NA
Short chain fatty acid meta DNA	K00382	lpd, pdhD	dihydrolipoamide dehydrogenase [EC:1.8.1.4]	183	183	23.2	11.3	1	1	NA	NA	NA
Short chain fatty acid meta RNA	K00382	lpd, pdhD	dihydrolipoamide dehydrogenase [EC:1.8.1.4]	121	118	26.5	18.1	0.944	1.03	NA	NA	NA
Short chain fatty acid meta Transcripts/gene	NA	lpd, pdhD	dihydrolipoamide dehydrogenase [EC:1.8.1.4]	0.669	0.647	0.144	0.126	0.764	1.03	NA	NA	NA
Short chain fatty acid meta DNA	K03416	NA	methylmalonyl-CoA carboxyltransferase 5S subunit [EC:2.1.3.1]	0.0116	0.0213	0.0098	0.0176	0.504	1.84	NA	NA	NA
Short chain fatty acid meta DNA	K01652	ilvB, ilvG, ilvI	acetolactate synthase I/II/III large subunit [EC:2.2.1.6]	454	445	58.7	51.5	1	1.02	NA	NA	NA
Short chain fatty acid meta RNA	K01652	ilvB, ilvG, ilvI	acetolactate synthase I/II/III large subunit [EC:2.2.1.6]	394	304	53.6	35.6	0.0526	1.3	NA	NA	NA
Short chain fatty acid meta DNA	K01653	ilvH, ilvN	acetolactate synthase I/III small subunit [EC:2.2.1.6]	116	109	11.5	8.93	0.779	1.07	NA	NA	NA
Short chain fatty acid meta RNA	K01653	ilvH, ilvN	acetolactate synthase I/III small subunit [EC:2.2.1.6]	101	83.3	19.7	8.28	0.233	1.21	NA	NA	NA
Short chain fatty acid meta Transcripts/gene	NA	ilvH, ilvN	acetolactate synthase I/III small subunit [EC:2.2.1.6]	0.88	0.706	0.164	0.102	0.114	1.25	NA	NA	NA
Short chain fatty acid meta DNA	K00627	aceF, pdhC	pyruvate dehydrogenase E2 component (dihydrolipoamide acetyltransferase) [EC:2.3.1.12]	14.2	17.1	3.11	4.52	0.675	1.2	NA	NA	NA
Short chain fatty acid meta RNA	K00627	aceF, pdhC	pyruvate dehydrogenase E2 component (dihydrolipoamide acetyltransferase) [EC:2.3.1.12]	17.9	21.8	6.77	6.03	0.424	1.22	NA	NA	NA
Short chain fatty acid meta Transcripts/gene	NA	aceF, pdhC	pyruvate dehydrogenase E2 component (dihydrolipoamide acetyltransferase) [EC:2.3.1.12]	1.36	1.27	0.646	0.0789	1	1.07	NA	NA	NA
Short chain fatty acid meta DNA	K00634	ptb	phosphate butyryltransferase [EC:2.3.1.19]	114	128	8.87	7.96	0.113	1.12	NA	NA	NA
Short chain fatty acid meta RNA	K00634	ptb	phosphate butyryltransferase [EC:2.3.1.19]	101	85.3	20.5	9.35	0.204	1.18	NA	NA	NA
Short chain fatty acid meta Transcripts/gene	NA	ptb	phosphate butyryltransferase [EC:2.3.1.19]	0.883	0.667	0.178	0.0811	0.0291	1.32	NA	NA	NA
Short chain fatty acid meta DNA	K13923	pduL	phosphate propanoyltransferase [EC:2.3.1.222]	1.4	1.34	0.297	0.287	1	1.04	NA	NA	NA
Short chain fatty acid meta RNA	K13923	pduL	phosphate propanoyltransferase [EC:2.3.1.222]	1.21	1.29	0.401	0.492	0.944	1.07	NA	NA	NA
Short chain fatty acid meta Transcripts/gene	NA	pduL	phosphate propanoyltransferase [EC:2.3.1.222]	0.884	0.941	0.285	0.197	0.832	1.07	NA	NA	NA
Short chain fatty acid meta DNA	K00656	pflD	formate C-acetyltransferase [EC:2.3.1.54]	425	335	76.8	42.8	0.0738	1.27	NA	NA	NA
Short chain fatty acid meta RNA	K00656	pflD	formate C-acetyltransferase [EC:2.3.1.54]	753	648	192	268	0.623	1.16	NA	NA	NA
Short chain fatty acid meta Transcripts/gene	NA	pflD	formate C-acetyltransferase [EC:2.3.1.54]	1.8	1.97	0.5	0.86	0.832	1.1	NA	NA	NA
Short chain fatty acid meta DNA	K00625	pta	phosphate acetyltransferase [EC:2.3.1.8]	244	256	19.7	16.3	0.779	1.05	NA	NA	NA
Short chain fatty acid meta RNA	K00625	pta	phosphate acetyltransferase [EC:2.3.1.8]	652	545	131	61	0.312	1.19	NA	NA	NA
Short chain fatty acid meta DNA	K13788	pta	phosphate acetyltransferase [EC:2.3.1.8]	11.4	8.19	7.07	1.84	0.968	1.4	NA	NA	NA
Short chain fatty acid meta RNA	K13788	pta	phosphate acetyltransferase [EC:2.3.1.8]	10.2	7.36	4.02	1.5	0.344	1.38	NA	NA	NA
Short chain fatty acid meta DNA	K15024	pduL	putative phosphotransacetylase [EC:2.3.1.8]	22.2	17.9	5.4	4.7	0.513	1.24	NA	NA	NA
Short chain fatty acid meta RNA	K15024	pduL	putative phosphotransacetylase [EC:2.3.1.8]	46.3	40.3	9.41	13.2	0.62	1.15	NA	NA	NA
Short chain fatty acid meta Transcripts/gene	NA	pduL	putative phosphotransacetylase [EC:2.3.1.8]	2.6	2.11	0.72	0.211	0.409	1.24	NA	NA	NA
Short chain fatty acid meta DNA	K00626	atoB	acetyl-CoA C-acetyltransferase [EC:2.3.1.9]	109	107	19.6	22.1	1	1.01	NA	NA	NA
Short chain fatty acid meta RNA	K00626	atoB	acetyl-CoA C-acetyltransferase [EC:2.3.1.9]	461	146	255	40.7	0.0191	3.17	-0.0253	-0.00446	-0.018
Short chain fatty acid meta Transcripts/gene	NA	atoB	acetyl-CoA C-acetyltransferase [EC:2.3.1.9]	4.21	1.41	1.93	0.448	0.0419	2.99	NA	NA	NA
Short chain fatty acid meta DNA	K01641	HMGCS1, HMGCS	hydroxymethylglutaryl-CoA synthase [EC:2.3.3.10]	2.53	2.38	1.38	0.379	1	1.06	NA	NA	NA
Short chain fatty acid meta RNA	K01641	HMGCS1, HMGCS	hydroxymethylglutaryl-CoA synthase [EC:2.3.3.10]	0.169	0.418	0.0794	0.174	0.102	2.47	NA	NA	NA
Short chain fatty acid meta Transcripts/gene	NA	HMGCS1, HMGCS	hydroxymethylglutaryl-CoA synthase [EC:2.3.3.10]	0.0889	0.174	0.0703	0.0676	0.101	1.96	NA	NA	NA
Short chain fatty acid meta DNA	K01649	leuA	2-isopropylmalate synthase [EC:2.3.3.13]	329	326	38.2	34.1	1	1.01	NA	NA	NA
Short chain fatty acid meta RNA	K01649	leuA	2-isopropylmalate synthase [EC:2.3.3.13]	304	249	58	48.9	0.382	1.22	NA	NA	NA
Short chain fatty acid meta Transcripts/gene	NA	leuA	2-isopropylmalate synthase [EC:2.3.3.13]	0.933	0.779	0.209	0.206	0.338	1.2	NA	NA	NA
Short chain fatty acid meta DNA	K01655	LYS20, LYS21	homocitrate synthase [EC:2.3.3.14]	5.92	8.01	2.44	1.71	0.504	1.35	NA	NA	NA
Short chain fatty acid meta RNA	K01655	LYS20, LYS21	homocitrate synthase [EC:2.3.3.14]	1.06	1.13	0.27	0.244	0.788	1.06	NA	NA	NA
Short chain fatty acid meta DNA	K10977	aksA	methanogen homocitrate synthase [EC:2.3.3.14 2.3.3.-]	6.15	7.03	1.46	2.68	0.83	1.14	NA	NA	NA
Short chain fatty acid meta RNA	K10977	aksA	methanogen homocitrate synthase [EC:2.3.3.14 2.3.3.-]	0.28	0.884	0.151	0.593	0.121	3.16	NA	NA	NA
Short chain fatty acid meta Transcripts/gene	NA	aksA	methanogen homocitrate synthase [EC:2.3.3.14 2.3.3.-]	0.112	0.155	0.0191	0.105	0.652	1.38	NA	NA	NA
Short chain fatty acid meta DNA	K01659	prpC	2-methylcitrate synthase [EC:2.3.3.5]	1.95	1.81	0.998	0.438	1	1.08	NA	NA	NA
Short chain fatty acid meta RNA	K01659	prpC	2-methylcitrate synthase [EC:2.3.3.5]	1.96	1.63	0.669	0.579	0.572	1.2	0.000745	0.0259	0.0191
Short chain fatty acid meta Transcripts/gene	NA	prpC	2-methylcitrate synthase [EC:2.3.3.5]	1.26	0.935	0.914	0.357	0.832	1.35	NA	NA	NA
Short chain fatty acid meta DNA	K01638	aceB, glcB	malate synthase [EC:2.3.3.9]	0.324	0.147	0.17	0.043	0.0954	2.2	NA	NA	NA
Short chain fatty acid meta RNA	K01638	aceB, glcB	malate synthase [EC:2.3.3.9]	0.407	0.13	0.221	0.131	0.0727	3.14	NA	NA	NA
Short chain fatty acid meta Transcripts/gene	NA	aceB, glcB	malate synthase [EC:2.3.3.9]	1.65	0.918	1.24	1.01	0.409	1.8	NA	NA	NA
Short chain fatty acid meta DNA	K00822	NA	beta-alanine--pyruvate transaminase [EC:2.6.1.18]	0.052	0.0837	0.024	0.0426	0.62	1.61	NA	NA	NA
Short chain fatty acid meta RNA	K00822	NA	beta-alanine--pyruvate transaminase [EC:2.6.1.18]	0.0241	0.0178	0.0233	0.0153	0.73	1.35	NA	NA	NA
Short chain fatty acid meta Transcripts/gene	NA	NA	beta-alanine--pyruvate transaminase [EC:2.6.1.18]	0.6	0.252	0.602	0.227	0.492	2.38	NA	NA	NA
Short chain fatty acid meta DNA	K00823	puuE	4-aminobutyrate aminotransferase [EC:2.6.1.19]	12.5	11.9	3.82	11.6	0.563	1.05	NA	NA	NA
Short chain fatty acid meta RNA	K00823	puuE	4-aminobutyrate aminotransferase [EC:2.6.1.19]	9.59	12.4	3.55	5.44	0.519	1.29	NA	NA	NA
Short chain fatty acid meta DNA	K07250	gabT	4-aminobutyrate aminotransferase / (S)-3-amino-2-methylpropionate transaminase [EC:2.6.1.19 2.6.1.22]	8.56	3.77	3.85	0.937	0.0608	2.27	-0.0169	-0.00192	-0.0093
Short chain fatty acid meta Transcripts/gene	NA	gabT	4-aminobutyrate aminotransferase / (S)-3-amino-2-methylpropionate transaminase [EC:2.6.1.19 2.6.1.22]	0.588	1.01	0.349	0.448	0.147	1.71	NA	NA	NA
Short chain fatty acid meta Transcripts/gene	NA	gabT	4-aminobutyrate aminotransferase / (S)-3-amino-2-methylpropionate transaminase [EC:2.6.1.19 2.6.1.22]	0.588	1.01	0.349	0.448	0.0829	1.71	NA	NA	NA
Short chain fatty acid meta RNA	K07250	gabT	4-aminobutyrate aminotransferase / (S)-3-amino-2-methylpropionate transaminase [EC:2.6.1.19 2.6.1.22]	1.42	1.11	0.792	0.332	0.623	1.29	NA	NA	NA
Short chain fatty acid meta DNA	K13524	ABAT	4-aminobutyrate aminotransferase / (S)-3-amino-2-methylpropionate transaminase [EC:2.6.1.19 2.6.1.22]	0.00339	0.0035	0.00399	0.0022	1	1.04	NA	NA	NA
Short chain fatty acid meta DNA	K07250	gabT	4-aminobutyrate aminotransferase / (S)-3-amino-2-methylpropionate transaminase [EC:2.6.1.19 2.6.1.22]	8.56	3.77	3.85	0.937	0.0608	2.27	-0.0169	-0.00192	-0.0093
Short chain fatty acid meta Transcripts/gene	NA	gabT	4-aminobutyrate aminotransferase / (S)-3-amino-2-methylpropionate transaminase [EC:2.6.1.19 2.6.1.22]	0.182	0.302	0.0858	0.0736	0.147	1.66	NA	NA	NA
Short chain fatty acid meta Transcripts/gene	NA	gabT	4-aminobutyrate aminotransferase / (S)-3-amino-2-methylpropionate transaminase [EC:2.6.1.19 2.6.1.22]	0.182	0.302	0.0858	0.0736	0.0829	1.66	NA	NA	NA
Short chain fatty acid meta DNA	K00873	pyk	pyruvate kinase [EC:2.7.1.40]	214	208	31.7	31.1	1	1.03	NA	NA	NA
Short chain fatty acid meta RNA	K00873	pyk	pyruvate kinase [EC:2.7.1.40]	234	170	69.4	42.9	0.139	1.38	NA	NA	NA
Short chain fatty acid meta DNA	K12406	PKLR	pyruvate kinase isozymes R/L [EC:2.7.1.40]	0.0851	0.0891	0.0491	0.0503	1	1.05	NA	NA	NA

Short chain fatty acid meta Transcripts/gene	NA	pyk	pyruvate kinase [EC:2.7.1.40]	1.09	0.813	0.203	0.141	0.163	1.33	NA	NA	NA
Short chain fatty acid meta DNA	K00925	ackA	acetate kinase [EC:2.7.2.1]	352	335	30.7	17.2	0.878	1.05	NA	NA	NA
Short chain fatty acid meta RNA	K00925	ackA	acetate kinase [EC:2.7.2.1]	847	719	141	112	0.231	1.18	NA	NA	NA
Short chain fatty acid meta Transcripts/gene	NA	ackA	acetate kinase [EC:2.7.2.1]	2.43	2.14	0.524	0.254	0.37	1.14	NA	NA	NA
Short chain fatty acid meta DNA	K00932	tdcD, pduW	propionate kinase [EC:2.7.2.15]	0.0288	0.0358	0.015	0.0125	0.911	1.24	NA	NA	NA
Short chain fatty acid meta RNA	K00932	tdcD, pduW	propionate kinase [EC:2.7.2.15]	0.416	0.313	0.206	0.18	0.573	1.33	NA	NA	NA
Short chain fatty acid meta Transcripts/gene	NA	tdcD, pduW	propionate kinase [EC:2.7.2.15]	16.8	10.7	8.61	7.79	0.43	1.57	NA	NA	NA
Short chain fatty acid meta DNA	K00929	buk	butyrate kinase [EC:2.7.2.7]	145	166	21.8	12.1	0.302	1.15	NA	NA	NA
Short chain fatty acid meta RNA	K00929	buk	butyrate kinase [EC:2.7.2.7]	105	78.4	22.2	10.5	0.0715	1.34	NA	NA	NA
Short chain fatty acid meta Transcripts/gene	NA	buk	butyrate kinase [EC:2.7.2.7]	0.745	0.476	0.234	0.0905	0.0611	1.56	NA	NA	NA
Short chain fatty acid meta DNA	K01006	ppdK	pyruvate, orthophosphate dikinase [EC:2.7.9.1]	665	700	36.6	29.7	0.565	1.05	NA	NA	NA
Short chain fatty acid meta RNA	K01006	ppdK	pyruvate, orthophosphate dikinase [EC:2.7.9.1]	4850	4680	494	532	0.47	1.04	NA	NA	NA
Short chain fatty acid meta Transcripts/gene	NA	ppdK	pyruvate, orthophosphate dikinase [EC:2.7.9.1]	7.31	6.69	0.87	0.709	0.314	1.09	NA	NA	NA
Short chain fatty acid meta DNA	K01007	pps, ppsA	pyruvate, water dikinase [EC:2.7.9.2]	83.6	88.7	13.6	18.6	0.989	1.06	NA	NA	NA
Short chain fatty acid meta RNA	K01007	pps, ppsA	pyruvate, water dikinase [EC:2.7.9.2]	75	45	43.7	2.95	0.0984	1.67	NA	NA	NA
Short chain fatty acid meta Transcripts/gene	NA	pps, ppsA	pyruvate, water dikinase [EC:2.7.9.2]	0.886	0.53	0.444	0.128	0.183	1.67	NA	NA	NA
Short chain fatty acid meta DNA	K01026	pct	propionate CoA-transferase [EC:2.8.3.1]	34.5	23.4	10.8	8.29	0.563	1.48	NA	NA	NA
Short chain fatty acid meta RNA	K01026	pct	propionate CoA-transferase [EC:2.8.3.1]	40.3	16.4	14.6	6.65	0.0326	2.45	-0.0226	-0.00555	-0.0146
Short chain fatty acid meta Transcripts/gene	NA	pct	propionate CoA-transferase [EC:2.8.3.1]	1.32	0.732	0.77	0.269	0.223	1.8	NA	NA	NA
Short chain fatty acid meta DNA	K01039	gctA	glutaconate CoA-transferase, subunit A [EC:2.8.3.12]	3.03	3.36	0.351	1.27	0.731	1.11	NA	NA	NA
Short chain fatty acid meta RNA	K01039	gctA	glutaconate CoA-transferase, subunit A [EC:2.8.3.12]	17.5	16	7.63	5.78	0.972	1.09	NA	NA	NA
Short chain fatty acid meta DNA	K01040	gctB	glutaconate CoA-transferase, subunit B [EC:2.8.3.12]	2.61	2.93	0.322	1.07	0.779	1.12	NA	NA	NA
Short chain fatty acid meta RNA	K01040	gctB	glutaconate CoA-transferase, subunit B [EC:2.8.3.12]	13.2	13.2	4.51	4.66	0.971	1	NA	NA	NA
Short chain fatty acid meta Transcripts/gene	NA	gctB	glutaconate CoA-transferase, subunit B [EC:2.8.3.12]	5.62	4.76	2.66	0.932	0.895	1.18	NA	NA	NA
Short chain fatty acid meta DNA	K01027	OXCT	3-oxoacid CoA-transferase [EC:2.8.3.5]	0.179	0.327	0.0492	0.138	0.0993	1.83	NA	NA	NA
Short chain fatty acid meta RNA	K01027	OXCT	3-oxoacid CoA-transferase [EC:2.8.3.5]	0.636	0.732	0.312	0.434	0.989	1.15	NA	NA	NA
Short chain fatty acid meta DNA	K01028	scoA	3-oxoacid CoA-transferase subunit A [EC:2.8.3.5]	9.1	10.9	2.4	2.3	0.83	1.2	NA	NA	NA
Short chain fatty acid meta RNA	K01028	scoA	3-oxoacid CoA-transferase subunit A [EC:2.8.3.5]	1.72	1.56	0.629	0.427	0.895	1.1	NA	NA	NA
Short chain fatty acid meta DNA	K01029	scoB	3-oxoacid CoA-transferase subunit B [EC:2.8.3.5]	6.56	9.9	1.62	3	0.208	1.51	NA	NA	NA
Short chain fatty acid meta RNA	K01029	scoB	3-oxoacid CoA-transferase subunit B [EC:2.8.3.5]	16.9	20.6	4.23	5.19	0.283	1.22	NA	NA	NA
Short chain fatty acid meta Transcripts/gene	NA	scoA/scoB	3-oxoacid CoA-transferase subunit B [EC:2.8.3.5]	1.24	1.1	0.345	0.217	0.625	1.13	NA	NA	NA
Short chain fatty acid meta DNA	K01034	atoD	acetate CoA/acetoacetate CoA-transferase alpha subunit [EC:2.8.3.8 2.8.3.9]	7.32	10.6	1.87	3.29	0.252	1.45	NA	NA	NA
Short chain fatty acid meta RNA	K01034	atoD	acetate CoA/acetoacetate CoA-transferase alpha subunit [EC:2.8.3.8 2.8.3.9]	29.7	20.4	14	4.59	0.519	1.46	NA	NA	NA
Short chain fatty acid meta DNA	K01035	atoA	acetate CoA/acetoacetate CoA-transferase beta subunit [EC:2.8.3.8 2.8.3.9]	1.17	1.91	0.153	0.551	0.176	1.64	NA	NA	NA
Short chain fatty acid meta RNA	K01035	atoA	acetate CoA/acetoacetate CoA-transferase beta subunit [EC:2.8.3.8 2.8.3.9]	4.27	4.74	1.12	1.71	0.843	1.11	NA	NA	NA
Short chain fatty acid meta Transcripts/gene	NA	atoA	acetate CoA/acetoacetate CoA-transferase beta subunit [EC:2.8.3.8 2.8.3.9]	3.94	2.05	0.957	0.357	0.0419	1.92	NA	NA	NA
Short chain fatty acid meta DNA	K07518	NA	hydroxybutyrate-dimer hydrolase [EC:3.1.1.22]	0.00675	0.003	0.00717	0.0052	0.641	2.28	NA	NA	NA
Short chain fatty acid meta DNA	K05973	phaZ	poly(3-hydroxybutyrate) depolymerase [EC:3.1.1.75]	0.103	0.161	0.0954	0.0703	0.45	1.56	NA	NA	NA
Short chain fatty acid meta DNA	K01067	ACH1	acetyl-CoA hydrolase [EC:3.1.2.1]	87.7	84	15.1	18.5	0.989	1.04	NA	NA	NA
Short chain fatty acid meta RNA	K01067	ACH1	acetyl-CoA hydrolase [EC:3.1.2.1]	236	181	40.2	43.6	0.188	1.3	NA	NA	NA
Short chain fatty acid meta Transcripts/gene	NA	ACH1	acetyl-CoA hydrolase [EC:3.1.2.1]	2.75	2.2	0.671	0.535	0.338	1.25	NA	NA	NA
Short chain fatty acid meta DNA	K05605	HIBCH	3-hydroxyisobutyryl-CoA hydrolase [EC:3.1.2.4]	0.0076	0.0306	0.00806	0.0199	0.11	4.02	NA	NA	NA
Short chain fatty acid meta DNA	K01069	gloB	hydroxyacylglutathione hydrolase [EC:3.1.2.6]	95.7	97	10.9	8.1	0.938	1.01	NA	NA	NA
Short chain fatty acid meta RNA	K01069	gloB	hydroxyacylglutathione hydrolase [EC:3.1.2.6]	18.5	16.5	3.01	1.73	0.283	1.12	NA	NA	NA
Short chain fatty acid meta Transcripts/gene	NA	gloB	hydroxyacylglutathione hydrolase [EC:3.1.2.6]	0.195	0.171	0.04	0.0164	0.537	1.15	NA	NA	NA
Short chain fatty acid meta DNA	K01505	NA	1-aminocyclopropane-1-carboxylate deaminase [EC:3.5.99.7]	0.709	1.01	0.286	0.412	0.731	1.43	NA	NA	NA
Short chain fatty acid meta RNA	K01505	NA	1-aminocyclopropane-1-carboxylate deaminase [EC:3.5.99.7]	0.299	0.39	0.145	0.189	0.623	1.31	NA	NA	NA
Short chain fatty acid meta Transcripts/gene	NA	NA	1-aminocyclopropane-1-carboxylate deaminase [EC:3.5.99.7]	0.473	0.393	0.275	0.16	0.832	1.2	NA	NA	NA
Short chain fatty acid meta DNA	K01512	acyP	acylphosphatase [EC:3.6.1.7]	18.5	18.3	1.97	6.02	1	1.01	NA	NA	NA
Short chain fatty acid meta RNA	K01512	acyP	acylphosphatase [EC:3.6.1.7]	5.02	4	1.67	1.78	0.382	1.25	NA	NA	NA
Short chain fatty acid meta Transcripts/gene	NA	acyP	acylphosphatase [EC:3.6.1.7]	0.276	0.222	0.107	0.0614	0.558	1.24	NA	NA	NA
Short chain fatty acid meta DNA	K01580	gadB, gadA, GAD	glutamate decarboxylase [EC:4.1.1.15]	1.34	2.04	0.34	0.497	0.132	1.52	0.0105	0.0258	0.021
Short chain fatty acid meta RNA	K01580	gadB, gadA, GAD	glutamate decarboxylase [EC:4.1.1.15]	0.0958	0.256	0.0863	0.0885	0.0959	2.67	NA	NA	NA
Short chain fatty acid meta Transcripts/gene	NA	gadB, gadA, GAD	glutamate decarboxylase [EC:4.1.1.15]	0.0741	0.13	0.0647	0.051	0.114	1.76	NA	NA	NA
Short chain fatty acid meta DNA	K01571	oadA	oxaloacetate decarboxylase, alpha subunit [EC:4.1.1.3]	114	141	13.6	33	0.298	1.23	NA	NA	NA
Short chain fatty acid meta RNA	K01571	oadA	oxaloacetate decarboxylase, alpha subunit [EC:4.1.1.3]	381	333	75.2	76.4	0.471	1.14	NA	NA	NA
Short chain fatty acid meta DNA	K01572	oadB	oxaloacetate decarboxylase, beta subunit [EC:4.1.1.3]	241	263	23.2	12.5	0.394	1.09	NA	NA	NA
Short chain fatty acid meta RNA	K01572	oadB	oxaloacetate decarboxylase, beta subunit [EC:4.1.1.3]	557	399	105	42.2	0.0938	1.4	NA	NA	NA
Short chain fatty acid meta DNA	K01573	oadG	oxaloacetate decarboxylase, gamma subunit [EC:4.1.1.3]	1.56	1.55	0.697	0.657	1	1.01	NA	NA	NA
Short chain fatty acid meta RNA	K01573	oadG	oxaloacetate decarboxylase, gamma subunit [EC:4.1.1.3]	1.54	0.95	1.1	0.451	0.572	1.62	NA	NA	NA
Short chain fatty acid meta Transcripts/gene	NA	oadG	oxaloacetate decarboxylase, gamma subunit [EC:4.1.1.3]	2.66	1.81	0.607	0.14	0.163	1.48	NA	NA	NA
Short chain fatty acid meta DNA	K01595	ppc	phosphoenolpyruvate carboxylase [EC:4.1.1.31]	14	4.43	8.29	2.29	0.0268	3.16	NA	NA	NA
Short chain fatty acid meta RNA	K01595	ppc	phosphoenolpyruvate carboxylase [EC:4.1.1.31]	3.85	0.874	2.36	0.238	0.00669	4.41	NA	NA	NA
Short chain fatty acid meta Transcripts/gene	NA	ppc	phosphoenolpyruvate carboxylase [EC:4.1.1.31]	0.288	0.33	0.0779	0.351	0.708	1.15	NA	NA	NA
Short chain fatty acid meta DNA	K01596	pckA, PEPCK	phosphoenolpyruvate carboxykinase (GTP) [EC:4.1.1.32]	29.8	39.1	8.76	7.07	0.252	1.31	NA	NA	NA
Short chain fatty acid meta RNA	K01596	pckA, PEPCK	phosphoenolpyruvate carboxykinase (GTP) [EC:4.1.1.32]	214	189	73.8	75.3	0.52	1.13	NA	NA	NA
Short chain fatty acid meta Transcripts/gene	NA	pckA, PEPCK	phosphoenolpyruvate carboxykinase (GTP) [EC:4.1.1.32]	7.47	4.77	2.85	1.37	0.187	1.57	NA	NA	NA
Short chain fatty acid meta DNA	K01574	adc	acetoacetate decarboxylase [EC:4.1.1.4]	0.436	0.713	0.188	0.457	0.83	1.64	NA	NA	NA
Short chain fatty acid meta DNA	K11264	mmcD	methylmalonyl-CoA decarboxylase [EC:4.1.1.41]	4.71	9.82	8.68	3.18	0.176	2.08	NA	NA	NA
Short chain fatty acid meta RNA	K11264	mmcD	methylmalonyl-CoA decarboxylase [EC:4.1.1.41]	1.25	5.24	1.61	2.81	0.0494	4.2	NA	NA	NA
Short chain fatty acid meta Transcripts/gene	NA	mmcD	methylmalonyl-CoA decarboxylase [EC:4.1.1.41]	0.428	0.517	0.25	0.166	0.561	1.21	NA	NA	NA
Short chain fatty acid meta DNA	K01610	pckA	phosphoenolpyruvate carboxykinase (ATP) [EC:4.1.1.49]	392	374	30.6	13.4	0.301	1.05	NA	NA	NA
Short chain fatty acid meta RNA	K01610	pckA	phosphoenolpyruvate carboxykinase (ATP) [EC:4.1.1.49]	2130	1690	414	185	0.144	1.26	NA	NA	NA
Short chain fatty acid meta Transcripts/gene	NA	pckA	phosphoenolpyruvate carboxykinase (ATP) [EC:4.1.1.49]	5.47	4.53	1.12	0.471	0.183	1.21	NA	NA	NA
Short chain fatty acid meta DNA	K01575	alsD, budA, aldC	acetolactate decarboxylase [EC:4.1.1.5]	20.1	24.2	8.41	6.18	0.731	1.2	NA	NA	NA
Short chain fatty acid meta RNA	K01575	alsD, budA, aldC	acetolactate decarboxylase [EC:4.1.1.5]	8.85	12.2	4.46	8.35	0.73	1.38	NA	NA	NA
Short chain fatty acid meta Transcripts/gene	NA	alsD, budA, aldC	acetolactate decarboxylase [EC:4.1.1.5]	0.444	0.482	0.102	0.239	0.87	1.08	NA	NA	NA
Short chain fatty acid meta DNA	K01615	gcdA	glutaconyl-CoA decarboxylase [EC:4.1.1.70]	28.7	30.6	6.56	6.46	1	1.06	NA	NA	NA
Short chain fatty acid meta RNA	K01615	gcdA	glutaconyl-CoA decarboxylase [EC:4.1.1.70]	198	133	45	24.3	0.0394	1.5	-0.0223	-0.00841	-0.0158
Short chain fatty acid meta Transcripts/gene	NA	gcdA	glutaconyl-CoA decarboxylase [EC:4.1.1.70]	7.13	4.38	1.78	0.578	0.037	1.63	NA	NA	NA
Short chain fatty acid meta DNA	K07246	ttuC, dmlA	tartrate dehydrogenase/decarboxylase / D-malate dehydrogenase [EC:1.1.1.93 4.1.1.73 1.1.1.83]	17.3	19.7	3.46	7.97	1	1.14	NA	NA	NA
Short chain fatty acid meta RNA	K07246	ttuC, dmlA	tartrate dehydrogenase/decarboxylase / D-malate dehydrogenase [EC:1.1.1.93 4.1.1.73 1.1.1.83]	4.9	3.62	1.79	1.32	0.344	1.35	NA	NA	NA
Short chain fatty acid meta Transcripts/gene	NA	ttuC, dmlA	tartrate dehydrogenase/decarboxylase / D-malate dehydrogenase [EC:1.1.1.93 4.1.1.73 1.1.1.83]	0.3	0.203	0.134	0.1	0.355	1.47	NA	NA	NA
Short chain fatty acid meta DNA	K01578	MLYCD	malonyl-CoA decarboxylase [EC:4.1.1.9]	0.0179	0.0141	0.0242	0.0103	0.989	1.27	NA	NA	NA

Short chain fatty acid meta DNA	K03417	prpB	methylisocitrate lyase [EC:4.1.3.30]	1.06	1.01	0.214	0.181	0.968	1.05	NA	NA	NA
Short chain fatty acid meta RNA	K03417	prpB	methylisocitrate lyase [EC:4.1.3.30]	0.636	0.519	0.224	0.178	0.424	1.23	NA	NA	NA
Short chain fatty acid meta Transcripts/gene	NA	prpB	methylisocitrate lyase [EC:4.1.3.30]	0.65	0.529	0.366	0.208	0.958	1.23	NA	NA	NA
Short chain fatty acid meta DNA	K01640	hmgL	hydroxymethylglutaryl-CoA lyase [EC:4.1.3.4]	5.11	4.38	1.41	1.86	0.911	1.17	NA	NA	NA
Short chain fatty acid meta RNA	K01640	hmgL	hydroxymethylglutaryl-CoA lyase [EC:4.1.3.4]	2.72	1.62	1.24	0.519	0.179	1.68	NA	NA	NA
Short chain fatty acid meta Transcripts/gene	NA	hmgL	hydroxymethylglutaryl-CoA lyase [EC:4.1.3.4]	0.566	0.441	0.254	0.212	0.45	1.28	NA	NA	NA
Short chain fatty acid meta Transcripts/gene	NA	abfD	4-hydroxybutyryl-CoA dehydratase / vinylacetyl-CoA-Delta-isomerase [EC:4.2.1.120 5.3.3.3]	1.65	1.7	0.773	0.449	0.87	1.03	NA	NA	NA
Short chain fatty acid meta DNA	K01692	paaF, echA	enoyl-CoA hydratase [EC:4.2.1.17]	13.9	9.04	3.87	2.5	0.198	1.54	NA	NA	NA
Short chain fatty acid meta RNA	K01692	paaF, echA	enoyl-CoA hydratase [EC:4.2.1.17]	40.4	8.53	42.2	2.35	0.204	4.73	NA	NA	NA
Short chain fatty acid meta RNA	K07511	ECHS1	enoyl-CoA hydratase [EC:4.2.1.17]	0.103	0.0399	0.0514	0.0527	0.147	2.57	NA	NA	NA
Short chain fatty acid meta Transcripts/gene	NA	NA	enoyl-CoA hydratase / long-chain 3-hydroxyacyl-CoA dehydrogenase [EC:4.2.1.17 1.1.1.211]	2.46	1.04	1.97	0.218	0.37	2.37	NA	NA	NA
Short chain fatty acid meta DNA	K07511	ECHS1	enoyl-CoA hydratase [EC:4.2.1.17]	0.0538	0.0553	0.031	0.0491	0.989	1.03	NA	NA	NA
Short chain fatty acid meta DNA	K07515	HADHA	enoyl-CoA hydratase / long-chain 3-hydroxyacyl-CoA dehydrogenase [EC:4.2.1.17 1.1.1.211]	0.0298	0.0326	0.0212	0.0239	1	1.09	NA	NA	NA
Short chain fatty acid meta RNA	K07515	HADHA	enoyl-CoA hydratase / long-chain 3-hydroxyacyl-CoA dehydrogenase [EC:4.2.1.17 1.1.1.211]	0.155	0.133	0.127	0.0792	0.989	1.17	NA	NA	NA
Short chain fatty acid meta DNA	K07514	EHHADH	enoyl-CoA hydratase / 3-hydroxyacyl-CoA dehydrogenase / 3,2-trans-enoyl-CoA isomerase [EC:4.2.1.17 1.1.1.35 5.3.3.8]	0.0211	0.0123	0.0139	0.0097	0.786	1.72	NA	NA	NA
Short chain fatty acid meta RNA	K07514	EHHADH	enoyl-CoA hydratase / 3-hydroxyacyl-CoA dehydrogenase / 3,2-trans-enoyl-CoA isomerase [EC:4.2.1.17 1.1.1.35 5.3.3.8]	0.0427	0.011	0.0332	0.0217	0.188	3.89	NA	NA	NA
Short chain fatty acid meta Transcripts/gene	NA	acnA	aconitate hydratase 2 / 2-methylisocitrate dehydratase [EC:4.2.1.3 4.2.1.99]	0.88	0.724	0.28	0.187	0.43	1.21	NA	NA	NA
Short chain fatty acid meta RNA	K01682	acnB	aconitate hydratase 2 / 2-methylisocitrate dehydratase [EC:4.2.1.3 4.2.1.99]	1.68	1.07	0.563	0.78	0.311	1.57	NA	NA	NA
Short chain fatty acid meta DNA	K01682	acnB	aconitate hydratase 2 / 2-methylisocitrate dehydratase [EC:4.2.1.3 4.2.1.99]	1.06	1.18	0.432	0.592	1	1.12	NA	NA	NA
Short chain fatty acid meta DNA	K01715	crt	enoyl-CoA hydratase [EC:4.2.1.17]	47.2	48.4	5.21	8.7	1	1.03	NA	NA	NA
Short chain fatty acid meta RNA	K01715	crt	3-hydroxybutyryl-CoA dehydratase [EC:4.2.1.55]	161	89.6	50.2	25.7	0.0698	1.79	-0.0328	-0.00798	-0.0258
Short chain fatty acid meta Transcripts/gene	NA	crt	3-hydroxybutyryl-CoA dehydratase [EC:4.2.1.55]	3.28	1.86	0.992	0.477	0.037	1.76	NA	NA	NA
Short chain fatty acid meta DNA	K01720	prpD	2-methylcitrate dehydratase [EC:4.2.1.79]	0.537	0.854	0.195	0.267	0.172	1.59	NA	NA	NA
Short chain fatty acid meta RNA	K01720	prpD	2-methylcitrate dehydratase [EC:4.2.1.79]	0.0127	0.113	0.0181	0.0979	0.139	8.86	NA	NA	NA
Short chain fatty acid meta Transcripts/gene	NA	prpD	2-methylcitrate dehydratase [EC:4.2.1.79]	0.0332	0.129	0.051	0.0852	0.155	3.89	NA	NA	NA
Short chain fatty acid meta Transcripts/gene	NA	NA	aconitate hydratase 2 / 2-methylisocitrate dehydratase [EC:4.2.1.3 4.2.1.99]	1.92	0.898	1.22	0.44	0.203	2.14	NA	NA	NA
Short chain fatty acid meta DNA	K01734	mgsA	methylglyoxal synthase [EC:4.2.3.3]	30	35.4	3.53	5.91	0.17	1.18	NA	NA	NA
Short chain fatty acid meta RNA	K01734	mgsA	methylglyoxal synthase [EC:4.2.3.3]	48.3	43.5	9.59	10.7	0.572	1.11	NA	NA	NA
Short chain fatty acid meta Transcripts/gene	NA	mgsA	methylglyoxal synthase [EC:4.2.3.3]	1.63	1.26	0.34	0.347	0.294	1.29	NA	NA	NA
Short chain fatty acid meta DNA	K01759	gloA	lactoylglutathione lyase [EC:4.4.1.5]	33.9	29.7	7.39	13	0.779	1.14	NA	NA	NA
Short chain fatty acid meta RNA	K01759	gloA	lactoylglutathione lyase [EC:4.4.1.5]	12.7	19.2	8.27	24.9	0.676	1.52	NA	NA	NA
Short chain fatty acid meta Transcripts/gene	NA	gloA	lactoylglutathione lyase [EC:4.4.1.5]	0.361	0.537	0.181	0.522	1	1.49	NA	NA	NA
Short chain fatty acid meta Transcripts/gene	NA	fadJ	3-hydroxyacyl-CoA dehydrogenase / enoyl-CoA hydratase / 3-hydroxybutyryl-CoA epimerase / enoyl-CoA isomerase [EC:1.1.1.35 4.2.1.17 5.1.2.3 5.3.3.	1.94	1.76	1.2	0.856	0.832	1.1	NA	NA	NA
Short chain fatty acid meta DNA	K05606	epi	methylmalonyl-CoA/ethylmalonyl-CoA epimerase [EC:5.1.99.1]	47.4	48	5.44	6.07	1	1.01	NA	NA	NA
Short chain fatty acid meta RNA	K05606	epi	methylmalonyl-CoA/ethylmalonyl-CoA epimerase [EC:5.1.99.1]	169	139	20.9	22.2	0.186	1.21	NA	NA	NA
Short chain fatty acid meta Transcripts/gene	NA	epi	methylmalonyl-CoA/ethylmalonyl-CoA epimerase [EC:5.1.99.1]	3.61	2.91	0.621	0.408	0.155	1.24	NA	NA	NA
Short chain fatty acid meta DNA	K01799	nicE, maiA	maleate isomerase [EC:5.2.1.1]	0.232	0.237	0.066	0.0728	1	1.02	NA	NA	NA
Short chain fatty acid meta RNA	K01799	nicE, maiA	maleate isomerase [EC:5.2.1.1]	0.0238	0.0238	0.0371	0.025	0.895	1	NA	NA	NA
Short chain fatty acid meta Transcripts/gene	NA	nicE, maiA	maleate isomerase [EC:5.2.1.1]	0.0947	0.098	0.137	0.0946	0.87	1.03	NA	NA	NA
Short chain fatty acid meta DNA	K14534	abfD	4-hydroxybutyryl-CoA dehydratase / vinylacetyl-CoA-Delta-isomerase [EC:4.2.1.120 5.3.3.3]	19	12.7	4.26	4.13	0.174	1.49	-0.0131	-0.00533	-0.00934
Short chain fatty acid meta RNA	K14534	abfD	4-hydroxybutyryl-CoA dehydratase / vinylacetyl-CoA-Delta-isomerase [EC:4.2.1.120 5.3.3.3]	29.4	21.7	9.8	8.35	0.424	1.36	NA	NA	NA
Short chain fatty acid meta Transcripts/gene	NA	abfD	4-hydroxybutyryl-CoA dehydratase / vinylacetyl-CoA-Delta-isomerase [EC:4.2.1.120 5.3.3.3]	1.65	1.7	0.773	0.449	0.87	1.03	NA	NA	NA
Short chain fatty acid meta Transcripts/gene	NA	EHHADH	enoyl-CoA hydratase / 3-hydroxyacyl-CoA dehydrogenase / 3,2-trans-enoyl-CoA isomerase [EC:4.2.1.17 1.1.1.35 5.3.3.8]	3.71	3.34	3.59	2.13	0.832	1.11	NA	NA	NA
Short chain fatty acid meta DNA	K01847	MUT	methylmalonyl-CoA mutase [EC:5.4.99.2]	410	414	55.7	64	1	1.01	NA	NA	NA
Short chain fatty acid meta RNA	K01847	MUT	methylmalonyl-CoA mutase [EC:5.4.99.2]	1320	1270	307	237	0.73	1.04	NA	NA	NA
Short chain fatty acid meta DNA	K01848	mcmA1	methylmalonyl-CoA mutase, N-terminal domain [EC:5.4.99.2]	13.4	10.9	4.18	2.42	0.779	1.23	NA	NA	NA
Short chain fatty acid meta RNA	K01848	mcmA1	methylmalonyl-CoA mutase, N-terminal domain [EC:5.4.99.2]	435	250	92.3	60.7	0.0164	1.74	-0.0262	-0.0127	-0.0202
Short chain fatty acid meta DNA	K01849	mcmA2	methylmalonyl-CoA mutase, C-terminal domain [EC:5.4.99.2]	3.9	3.29	1.22	0.726	0.938	1.18	NA	NA	NA
Short chain fatty acid meta RNA	K01849	mcmA2	methylmalonyl-CoA mutase, C-terminal domain [EC:5.4.99.2]	90	52.8	23.5	10.4	0.0164	1.71	-0.024	-0.0113	-0.0185
Short chain fatty acid meta DNA	K11942	NA	methylmalonyl-CoA mutase [EC:5.4.99.2]	0.0325	0.0316	0.0221	0.0128	0.968	1.03	NA	NA	NA
Short chain fatty acid meta Transcripts/gene	NA	mcmA2	methylmalonyl-CoA mutase, C-terminal domain [EC:5.4.99.2]	4.39	3.69	0.936	0.57	0.336	1.19	NA	NA	NA
Short chain fatty acid meta DNA	K01895	acs	acetyl-CoA synthetase [EC:6.2.1.1]	264	319	29.4	22.4	0.0906	1.21	NA	NA	NA
Short chain fatty acid meta RNA	K01895	acs	acetyl-CoA synthetase [EC:6.2.1.1]	491	458	133	167	0.895	1.07	NA	NA	NA
Short chain fatty acid meta Transcripts/gene	NA	acs	acetyl-CoA synthetase [EC:6.2.1.1]	1.9	1.44	0.616	0.503	0.471	1.32	NA	NA	NA
Short chain fatty acid meta DNA	K01905	NA	acetyl-CoA synthetase (ADP-forming) [EC:6.2.1.13]	2.45	3.82	0.709	1.38	0.45	1.56	NA	NA	NA
Short chain fatty acid meta RNA	K01905	NA	acetyl-CoA synthetase (ADP-forming) [EC:6.2.1.13]	1.77	3.54	0.638	0.783	0.0269	2	NA	NA	NA
Short chain fatty acid meta Transcripts/gene	NA	NA	acetyl-CoA synthetase (ADP-forming) [EC:6.2.1.13]	0.752	0.977	0.296	0.216	0.186	1.3	NA	NA	NA
Short chain fatty acid meta DNA	K01907	acsA	acetoacetyl-CoA synthetase [EC:6.2.1.16]	1.18	2.06	0.377	0.757	0.25	1.74	NA	NA	NA
Short chain fatty acid meta RNA	K01907	acsA	acetoacetyl-CoA synthetase [EC:6.2.1.16]	0.555	0.966	0.437	0.61	0.253	1.74	NA	NA	NA
Short chain fatty acid meta Transcripts/gene	NA	acsA	acetoacetyl-CoA synthetase [EC:6.2.1.16]	0.443	0.461	0.273	0.183	0.87	1.04	NA	NA	NA
Short chain fatty acid meta DNA	K01908	prpE	propionyl-CoA synthetase [EC:6.2.1.17]	5.34	3.86	2.95	0.646	0.99	1.38	NA	NA	NA
Short chain fatty acid meta RNA	K01908	prpE	propionyl-CoA synthetase [EC:6.2.1.17]	0.315	0.402	0.171	0.266	0.73	1.27	NA	NA	NA
Short chain fatty acid meta Transcripts/gene	NA	prpE	propionyl-CoA synthetase [EC:6.2.1.17]	0.0769	0.0999	0.0656	0.0472	0.489	1.3	NA	NA	NA
Short chain fatty acid meta DNA	K01896	ACSM	medium-chain acyl-CoA synthetase [EC:6.2.1.2]	1.06	1.48	0.394	0.438	0.62	1.39	NA	NA	NA
Short chain fatty acid meta RNA	K01896	ACSM	medium-chain acyl-CoA synthetase [EC:6.2.1.2]	1.8	1.81	0.535	0.85	0.895	1	NA	NA	NA
Short chain fatty acid meta Transcripts/gene	NA	ACSM	medium-chain acyl-CoA synthetase [EC:6.2.1.2]	1.96	1.25	1.3	0.498	0.355	1.58	NA	NA	NA
Short chain fatty acid meta Transcripts/gene	NA	LSC2	succinyl-CoA synthetase beta subunit [EC:6.2.1.4 6.2.1.5]	25.8	19.2	14.3	10.1	0.5	1.35	NA	NA	NA
Short chain fatty acid meta RNA	K01900	LSC2	succinyl-CoA synthetase beta subunit [EC:6.2.1.4 6.2.1.5]	4.78	3.78	3.46	1.82	0.989	1.27	NA	NA	NA
Short chain fatty acid meta DNA	K01899	LSC1	succinyl-CoA synthetase alpha subunit [EC:6.2.1.4 6.2.1.5]	0.0962	0.136	0.0626	0.092	0.911	1.42	NA	NA	NA
Short chain fatty acid meta RNA	K01899	LSC1	succinyl-CoA synthetase alpha subunit [EC:6.2.1.4 6.2.1.5]	3.4	2.69	2.6	1.27	1	1.27	NA	NA	NA
Short chain fatty acid meta DNA	K01900	LSC2	succinyl-CoA synthetase beta subunit [EC:6.2.1.4 6.2.1.5]	0.209	0.229	0.0404	0.0503	0.875	1.1	NA	NA	NA
Short chain fatty acid meta DNA	K01902	sucD	succinyl-CoA synthetase alpha subunit [EC:6.2.1.5]	13.7	16	3.33	8.69	1	1.17	NA	NA	NA
Short chain fatty acid meta RNA	K01902	sucD	succinyl-CoA synthetase alpha subunit [EC:6.2.1.5]	19.4	33.8	8.77	31.9	1	1.75	NA	NA	NA
Short chain fatty acid meta DNA	K01903	sucC	succinyl-CoA synthetase beta subunit [EC:6.2.1.5]	18.7	21.5	3.77	10.9	1	1.15	NA	NA	NA
Short chain fatty acid meta RNA	K01903	sucC	succinyl-CoA synthetase beta subunit [EC:6.2.1.5]	23.4	40.9	13.6	39.7	0.944	1.75	NA	NA	NA
Short chain fatty acid meta Transcripts/gene	NA	sucC	succinyl-CoA synthetase beta subunit [EC:6.2.1.5]	1.63	2.1	0.923	1.72	0.896	1.29	NA	NA	NA
Short chain fatty acid meta Transcripts/gene	NA	ACACA	acetyl-CoA carboxylase / biotin carboxylase [EC:6.4.1.2 6.3.4.14]	0.637	0.824	0.174	0.192	0.323	1.29	NA	NA	NA
Short chain fatty acid meta DNA	K01958	pyc	pyruvate carboxylase [EC:6.4.1.1]	69.2	57.8	16.9	16.2	0.83	1.2	NA	NA	NA
Short chain fatty acid meta RNA	K01958	pyc	pyruvate carboxylase [EC:6.4.1.1]	45.2	30.6	11.3	6.65	0.0968	1.48	NA	NA	NA
Short chain fatty acid meta DNA	K01959	pycA	pyruvate carboxylase subunit A [EC:6.4.1.1]	9.52	9.54	2.05	3.39	1	1	NA	NA	NA
Short chain fatty acid meta RNA	K01959	pycA	pyruvate carboxylase subunit A [EC:6.4.1.1]	1.54	3	0.375	1.01	0.0364	1.95	NA	NA	NA
Short chain fatty acid meta DNA	K01960	pycB	pyruvate carboxylase subunit B [EC:6.4.1.1]	249	255	26.4	23.4	0.989	1.02	NA	NA	NA
Short chain fatty acid meta RNA	K01960	pycB	pyruvate carboxylase subunit B [EC:6.4.1.1]	501	455	80.2	61.6	0.382	1.1	NA	NA	NA
Short chain fatty acid meta Transcripts/gene	NA	pycB	pyruvate carboxylase subunit B [EC:6.4.1.1]	1.71	1.52	0.413	0.163	0.652	1.13	NA	NA	NA

Short chain fatty acid meta	DNA	K01962	accA	acetyl-CoA carboxylase carboxyl transferase subunit alpha [EC:6.4.1.2]	44	32.8	8.67	6.6	0.134	1.34	NA	NA	NA
Short chain fatty acid meta	RNA	K01962	accA	acetyl-CoA carboxylase carboxyl transferase subunit alpha [EC:6.4.1.2]	14	16.5	3.72	4.73	0.73	1.17	NA	NA	NA
Short chain fatty acid meta	DNA	K01963	accD	acetyl-CoA carboxylase carboxyl transferase subunit beta [EC:6.4.1.2]	39.8	28.2	8.47	4.28	0.0993	1.41	NA	NA	NA
Short chain fatty acid meta	RNA	K01963	accD	acetyl-CoA carboxylase carboxyl transferase subunit beta [EC:6.4.1.2]	16.7	18.1	3.22	4.4	0.788	1.08	NA	NA	NA
Short chain fatty acid meta	Transcripts/gene	NA	accD	acetyl-CoA/propionyl-CoA carboxylase [EC:6.4.1.2 6.4.1.3]	0.487	0.668	0.163	0.128	0.174	1.37	NA	NA	NA
Short chain fatty acid meta	DNA	K01961	accC	acetyl-CoA carboxylase, biotin carboxylase subunit [EC:6.4.1.2 6.3.4.14]	76.9	56.7	19	11.9	0.346	1.36	NA	NA	NA
Short chain fatty acid meta	RNA	K01961	accC	acetyl-CoA carboxylase, biotin carboxylase subunit [EC:6.4.1.2 6.3.4.14]	36.6	39.9	9.39	11.9	0.79	1.09	NA	NA	NA
Short chain fatty acid meta	DNA	K11262	ACACA	acetyl-CoA carboxylase / biotin carboxylase [EC:6.4.1.2 6.3.4.14]	0.151	0.108	0.0849	0.0254	0.911	1.4	NA	NA	NA
Short chain fatty acid meta	RNA	K11262	ACACA	acetyl-CoA carboxylase / biotin carboxylase [EC:6.4.1.2 6.3.4.14]	0.264	0.308	0.177	0.0254	0.788	1.17	NA	NA	NA
Short chain fatty acid meta	DNA	K01964	accC	acetyl-CoA/propionyl-CoA carboxylase [EC:6.4.1.2 6.4.1.3]	0.0454	0.0694	0.0259	0.0161	0.246	1.53	NA	NA	NA
Short chain fatty acid meta	RNA	K01964	accC	acetyl-CoA/propionyl-CoA carboxylase [EC:6.4.1.2 6.4.1.3]	0.0421	0.0704	0.043	0.0824	0.843	1.67	NA	NA	NA
Short chain fatty acid meta	DNA	K15036	pccB	acetyl-CoA/propionyl-CoA carboxylase [EC:6.4.1.2 6.4.1.3]	1.42	1.59	0.369	0.529	0.875	1.11	NA	NA	NA
Short chain fatty acid meta	RNA	K15036	pccB	acetyl-CoA/propionyl-CoA carboxylase [EC:6.4.1.2 6.4.1.3]	7.53	5.46	1.74	1.14	0.102	1.38	NA	NA	NA
Short chain fatty acid meta	DNA	K01965	pccA	propionyl-CoA carboxylase alpha chain [EC:6.4.1.3]	3.99	3.86	1.15	0.812	1	1.03	NA	NA	NA
Short chain fatty acid meta	RNA	K01965	pccA	propionyl-CoA carboxylase alpha chain [EC:6.4.1.3]	9.99	6.81	2.91	1.52	0.144	1.47	NA	NA	NA
Short chain fatty acid meta	DNA	K01966	pccB	propionyl-CoA carboxylase beta chain [EC:6.4.1.3]	241	254	26.6	11.4	0.968	1.05	NA	NA	NA
Short chain fatty acid meta	RNA	K01966	pccB	propionyl-CoA carboxylase beta chain [EC:6.4.1.3]	1070	799	142	115	0.0212	1.34	NA	NA	NA
Short chain fatty acid meta	Transcripts/gene	NA	pccA/pccB	acetyl-CoA/propionyl-CoA carboxylase [EC:6.4.1.2 6.4.1.3]	4.49	3.11	0.865	0.369	0.0772	1.44	NA	NA	NA
Short chain fatty acid meta	DNA	K00100	NA	NA	913	882	75.1	58.6	0.968	1.03	NA	NA	NA
Short chain fatty acid meta	RNA	K00100	NA	NA	529	409	49	38.5	0.0164	1.29	-0.0385	-0.0101	-0.0276
Short chain fatty acid meta	DNA	K00241	sdhC, frdC	succinate dehydrogenase cytochrome b556 subunit	110	99.4	11.7	13.3	0.675	1.11	NA	NA	NA
Short chain fatty acid meta	RNA	K00241	sdhC, frdC	succinate dehydrogenase cytochrome b556 subunit	273	267	33.1	40.6	0.989	1.02	NA	NA	NA
Short chain fatty acid meta	DNA	K00242	sdhD, frdD	succinate dehydrogenase membrane anchor subunit	0.0469	0.0374	0.0345	0.0134	1	1.25	NA	NA	NA
Short chain fatty acid meta	RNA	K00242	sdhD, frdD	succinate dehydrogenase membrane anchor subunit	0.0741	0.0526	0.0763	0.0694	0.571	1.41	NA	NA	NA
Short chain fatty acid meta	DNA	K00246	frdC	fumarate reductase subunit C	0.159	0.17	0.038	0.0701	1	1.07	NA	NA	NA
Short chain fatty acid meta	RNA	K00246	frdC	fumarate reductase subunit C	0.913	0.241	0.974	0.134	0.623	3.78	NA	NA	NA
Short chain fatty acid meta	DNA	K00247	frdD	fumarate reductase subunit D	0.042	0.0701	0.0269	0.0768	1	1.67	NA	NA	NA
Short chain fatty acid meta	RNA	K00247	frdD	fumarate reductase subunit D	0.0376	0.118	0.0368	0.111	0.247	3.15	NA	NA	NA
Short chain fatty acid meta	DNA	K01726	NA	NA	203	204	36.2	12.4	0.875	1.01	NA	NA	NA
Short chain fatty acid meta	RNA	K01726	NA	NA	113	103	17.7	7.05	0.519	1.09	NA	NA	NA
Short chain fatty acid meta	DNA	K02160	accB, bccP	acetyl-CoA carboxylase biotin carboxyl carrier protein	18.1	14.6	2.89	1.73	0.0962	1.24	NA	NA	NA
Short chain fatty acid meta	RNA	K02160	accB, bccP	acetyl-CoA carboxylase biotin carboxyl carrier protein	11.3	9.88	3.01	1.47	0.731	1.14	NA	NA	NA
Short chain fatty acid meta	DNA	K03821	phbC, phaC	polyhydroxyalkanoate synthase [EC:2.3.1.-]	0.66	0.626	0.197	0.153	1	1.05	NA	NA	NA
Short chain fatty acid meta	RNA	K03821	phbC, phaC	polyhydroxyalkanoate synthase [EC:2.3.1.-]	1.8	2.05	0.801	0.673	0.676	1.14	NA	NA	NA
Short chain fatty acid meta	DNA	K04020	eutD	phosphotransacetylase	0.283	0.305	0.064	0.154	1	1.08	NA	NA	NA
Short chain fatty acid meta	RNA	K04020	eutD	phosphotransacetylase	0.093	0.164	0.0661	0.0848	0.312	1.76	NA	NA	NA
Short chain fatty acid meta	DNA	K04021	eutE	aldehyde dehydrogenase	0.586	0.438	0.178	0.145	0.675	1.34	NA	NA	NA
Short chain fatty acid meta	RNA	K04021	eutE	aldehyde dehydrogenase	1.52	1.13	0.411	0.334	0.311	1.34	NA	NA	NA
Short chain fatty acid meta	DNA	K13997	PDHX	dihydrolipoamide dehydrogenase-binding protein of pyruvate dehydrogenase complex	0.0147	0.0111	0.00776	0.0062	0.875	1.33	NA	NA	NA
Short chain fatty acid meta	DNA	K15037	NA	biotin carboxyl carrier protein	0.147	0.119	0.0584	0.0306	0.938	1.24	NA	NA	NA
Short chain fatty acid meta	RNA	K15037	NA	biotin carboxyl carrier protein	0.0592	0.0414	0.0507	0.047	0.652	1.43	NA	NA	NA
