# Supplementary material for: Rumen metagenome and metatranscriptome analyses of low methane yield sheep reveals a Sharpea-enriched microbiome characterised by lactic acid formation and utilisation
Source: Microbiome. 2016 Oct 19;4:56. doi: 10.1186/s40168-016-0201-2 (PMC5069950; doi:10.1186/s40168-016-0201-2)
Supplement: Additional file 10: Table S5. — Read mapping statistics of metagenome and metatranscriptome data for lcdA genes. (PDF 83 kb) [file 40168_2016_201_MOESM10_ESM.pdf]

Table S5. Read mapping statistics of metagenome and metatranscriptome data for lcdA genes.

	Low methane yield		High methane yield		Fold change	*P*-value
	Mean read counts (RPM)	Standard deviation	Mean read counts (RPM)	Standard deviation		
Metagenome	6.757	8.176	0.370	0.190	18.26	0.024
Metatranscriptome	0.549	0.369	0.453	0.143	1.21	0.958
