# Supplementary material for: Rumen metagenome and metatranscriptome analyses of low methane yield sheep reveals a Sharpea-enriched microbiome characterised by lactic acid formation and utilisation
Source: Microbiome. 2016 Oct 19;4:56. doi: 10.1186/s40168-016-0201-2 (PMC5069950; doi:10.1186/s40168-016-0201-2)
Supplement: Additional file 14: Table S6. — Differentially expressed genes of Sharpea azabuensis DSM20406 in low methane yield sheep according to metatranscriptome read mapping. (DOCX 25 kb) [file 40168_2016_201_MOESM14_ESM.docx]

Table S6. Highly and significant differentially expressed genes of Sharpea azabuensis DSM20406 in LMY sheep according to metatranscriptome read mapping.

| IMG gene ID | *P* value | Mean LMY | Mean HMY | log_2_ fold change | Product | KO | EC |
| --- | --- | --- | --- | --- | --- | --- | --- |
| 2608610835 | < 0.01 | 1084.38 | 75.70 | 3.84 | glyceraldehyde-3-phosphate dehydrogenase, type I | K00134 | EC:1.2.1.12 |
| 2608609366 | < 0.01 | 722.67 | 54.71 | 3.72 | Bacterial RNase P class B |  |  |
| 2608611229 | < 0.01 | 631.83 | 48.09 | 3.72 | fructose-1,6-bisphosphate aldolase, class II | K01624 | EC:4.1.2.13 |
| 2608610255 | < 0.01 | 391.03 | 39.06 | 3.32 | transfer-messenger RNA |  |  |
| 2608610332 | < 0.01 | 269.99 | 22.15 | 3.61 | glycogen/starch synthase, ADP-glucose type | K00703 | EC:2.4.1.21 |
| 2608609759 | < 0.01 | 241.90 | 18.74 | 3.69 | PTS system, lactose/cellobiose family IIC component |  |  |
| 2608610334 | < 0.01 | 241.49 | 15.16 | 3.99 | glucose-1-phosphate adenylyltransferase | K00975 | EC:2.7.7.27 |
| 2608610333 | < 0.01 | 215.60 | 16.73 | 3.69 | glucose-1-phosphate adenylyltransferase, GlgD subunit | K00975 | EC:2.7.7.27 |
| 2608610176 | < 0.01 | 180.04 | 12.43 | 3.86 | GGGtGRT protein |  |  |
| 2608610388 | < 0.01 | 167.49 | 11.79 | 3.83 | pyruvate:ferredoxin (flavodoxin) oxidoreductase, homodimeric | K03737 | EC:1.2.7.- |
| 2608610560 | < 0.01 | 159.05 | 8.78 | 4.18 | PTS system, mannose/fructose/N-acetylgalactosamine-specific IIB | K02794 | EC:2.7.1.69 |
| 2608610727 | < 0.01 | 153.30 | 10.45 | 3.87 | Response regulator receiver domain/Histidine kinase-, DNA gyrase B-, and HSP90-like ATPase |  |  |
| 2608609554 | < 0.01 | 144.40 | 12.02 | 3.59 | D-lactate dehydrogenase [EC:1.1.1.28] | K03778 | EC:1.1.1.28 |
| 2608610312 | < 0.01 | 139.80 | 12.37 | 3.50 | translation elongation factor TU | K02358 |  |
| 2608610562 | < 0.01 | 139.46 | 7.73 | 4.17 | PTS system, mannose/fructose/sorbose family, IID component | K02796 |  |
| 2608609003 | < 0.01 | 127.70 | 9.36 | 3.77 | Basic membrane lipoprotein Med, periplasmic binding protein (PBP1-ABC) superfamily | K07335 |  |
| 2608610522 | < 0.01 | 122.45 | 11.80 | 3.38 | PTS system, glucose subfamily, IIA component | K02810 |  |
| 2608611032 | < 0.01 | 121.48 | 10.23 | 3.57 | Phosphoenolpyruvate-protein kinase (PTS system EI component) | K08483 | EC:2.7.3.9 |
| 2608610561 | < 0.01 | 111.44 | 5.41 | 4.36 | PTS, mannose/fructose/N-acetylgalactosamine-specific component IIC | K02795 |  |
| 2608609292 | < 0.01 | 97.50 | 8.82 | 3.47 | phosphoglycerate kinase (EC 2.7.2.3) | K00927 | EC:2.7.2.3 |
| 2608611184 | < 0.01 | 85.14 | 6.21 | 3.78 | Phosphomannomutase | K01835 | EC:5.4.2.2 |
| 2608609622 | < 0.01 | 84.38 | 9.17 | 3.20 | pyruvate phosphate dikinase (EC 2.7.9.1) | K01006 | EC:2.7.9.1 |
| 2608609981 | < 0.01 | 82.40 | 4.74 | 4.12 | transcriptional regulator, LacI family | K02529 |  |
| 2608609291 | < 0.01 | 77.35 | 6.28 | 3.62 | triosephosphate isomerase (EC 5.3.1.1) | K01803 | EC:5.3.1.1 |
| 2608609750 | < 0.01 | 69.92 | 4.49 | 3.96 | small GTP-binding protein domain | K02355 |  |
| 2608609216 | < 0.01 | 64.84 | 3.18 | 4.35 | Beta-glucosidase/6-phospho-beta-glucosidase/beta-galactosidase | K01223 | EC:3.2.1.86 |
| 2608609423 | < 0.01 | 62.84 | 6.13 | 3.36 | ribosomal subunit interface protein | K05808 |  |
| 2608609885 | < 0.01 | 56.87 | 4.92 | 3.53 | glycogen/starch/alpha-glucan phosphorylases | K00688 | EC:2.4.1.1 |
| 2608610747 | < 0.01 | 54.23 | 3.26 | 4.06 | Protein kinase, ubiquinone biosynthesis, AarF/ABC1/UbiB family | K03688 |  |
| 2608610726 | < 0.01 | 52.14 | 4.20 | 3.63 | desulfoferrodoxin ferrous iron-binding domain | K05919 | EC:1.15.1.2 |
| 2608609289 | < 0.01 | 50.49 | 4.26 | 3.57 | Alcohol dehydrogenase, class IV | K04072 | EC:1.1.1.1 |
| 2608610116 | < 0.01 | 49.81 | 4.61 | 3.43 | phosphoribosylformylglycinamidine synthase, clade II | K01952 | EC:6.3.5.3 |
| 2608610524 | < 0.01 | 46.33 | 2.45 | 4.24 | Glycosidase | K05341 | EC:2.4.1.4 |
| 2608610563 | < 0.01 | 44.45 | 2.81 | 3.98 | Uncharacterized protein |  |  |
| 2608610775 | < 0.01 | 44.30 | 4.00 | 3.47 | pyruvate kinase | K00873 | EC:2.7.1.40 |
| 2608608981 | < 0.01 | 42.36 | 3.24 | 3.71 | Phosphotransferase system cellobiose-specific component IIB | K02760 | EC:2.7.1.69 |
| 2608610049 | < 0.01 | 41.09 | 3.33 | 3.63 | MacB-like periplasmic core domain/FtsX-like permease family | K02004 |  |
| 2608610591 | < 0.01 | 38.82 | 0.52 | 6.21 | hypothetical protein |  |  |
| 2608610313 | < 0.01 | 37.08 | 3.70 | 3.33 | translation elongation factor EF-G | K02355 |  |
| 2608611092 | < 0.01 | 37.07 | 2.02 | 4.20 | Glutamine phosphoribosylpyrophosphate amidotransferase | K00764 | EC:2.4.2.14 |
| 2608609886 | < 0.01 | 36.86 | 2.99 | 3.63 | alpha-1,4-glucan:alpha-1,4-glucan 6-glycosyltransferase | K00700 | EC:2.4.1.18 |
| 2608609432 | < 0.01 | 36.80 | 2.45 | 3.91 | glucose-6-phosphate isomerase | K01810 | EC:5.3.1.9 |
| 2608610317 | < 0.01 | 35.05 | 4.71 | 2.89 | DNA-directed RNA polymerase, beta' subunit, predominant form | K03046 | EC:2.7.7.6 |
| 2608609315 | < 0.01 | 34.74 | 3.31 | 3.39 | phosphoglycerate mutase | K15633 | EC:5.4.2.12 |
| 2608610776 | < 0.01 | 32.54 | 3.02 | 3.43 | 6-phosphofructokinase | K00850 | EC:2.7.1.11 |
| 2608611255 | < 0.01 | 32.15 | 2.02 | 3.99 | NADPH-dependent glutamate synthase beta chain/related oxidoreductase |  |  |
| 2608609283 | < 0.01 | 31.57 | 2.73 | 3.53 | sulfide dehydrogenase (flavoprotein) subunit SudA | K00266 | EC 1.8.1.- |
| 2608610008 | < 0.01 | 30.60 | 2.70 | 3.50 | 3-hydroxyacyl-CoA dehydrogenase | K00074 | EC:1.1.1.157 |
| 2608610087 | < 0.01 | 30.57 | 2.11 | 3.86 | D-alanyl-lipoteichoic acid acyltransferase DltB, MBOAT superfamily |  |  |
| 2608609543 | < 0.01 | 27.99 | 3.08 | 3.19 | PTS system, fructose-specific, IIB / IIA /IIC components | K02770 |  |
| 2608609336 | < 0.01 | 3.36 | 0.21 | 4.02 | 3-dehydroquinate dehydratase | K03785 | EC:4.2.1.10 |
| 2608609641 | < 0.01 | 2.38 | 0.33 | 2.85 | 3-deoxy-D-arabinoheptulosonate-7-phosphate synthase | K01626 | EC:2.5.1.54 |
| 2608610786 | < 0.01 | 1.18 | 0.15 | 2.99 | 3-phosphoshikimate 1-carboxyvinyltransferase | K00800 | EC:2.5.1.19 |
| 2608609054 | < 0.01 | 1.08 | 0.10 | 3.51 | shikimate dehydrogenase | K00014 | EC:1.1.1.25 |
| 2608610784 | < 0.01 | 1.16 | 0.18 | 2.69 | chorismate mutase | K14170 | EC 5.4.99.5 |
| 2608610785 | < 0.01 | 0.85 | 0.08 | 3.45 | chorismate synthase | K01736 | EC:4.2.3.5 |
| 2608610783 | < 0.01 | 0.34 | 0.01 | 5.08 | shikimate kinase | K00891 | EC:2.7.1.71 |
| 2608610787 | < 0.01 | 0.56 | 0.06 | 3.24 | 3-dehydroquinate synthase | K01735 | EC:4.2.3.4 |
| 2608609927 | < 0.01 | 3.81 | 0.42 | 3.17 | 2,3,4,5-tetrahydropyridine-2,6-dicarboxylate N-acetyltransferase | K00674 | EC 2.3.1.89 |
| 2608611247 | < 0.01 | 0.69 | 0.04 | 4.02 | pyrroline-5-carboxylate reductase | K00286 | EC:1.5.1.2 |
| 2608609296 | < 0.01 | 11.61 | 1.21 | 3.26 | dihydroxy-acid dehydratase | K01687 | EC:4.2.1.9 |
