# Supplementary material for: Rumen metagenome and metatranscriptome analyses of low methane yield sheep reveals a Sharpea-enriched microbiome characterised by lactic acid formation and utilisation
Source: Microbiome. 2016 Oct 19;4:56. doi: 10.1186/s40168-016-0201-2 (PMC5069950; doi:10.1186/s40168-016-0201-2)
Supplement: Additional file 15: Table S7. — Top 50 most highly and significant differentially expressed genes of Megasphaera elsdenii J1 in low methane yield sheep according to metatranscriptome read mapping. (DOCX 17 kb) [file 40168_2016_201_MOESM15_ESM.docx]

Table S7: Top 50 most highly and significant differentially expressed genes of Megasphaera elsdenii J1 in low methane yield sheep according to metatranscriptome read mapping.

| IMG gene ID | *P* value | mean low CH4 | mean high CH4 | log2 fold change | Product | KO | EC |
| --- | --- | --- | --- | --- | --- | --- | --- |
| 2628044249 | 0.04 | 566.50 | 0.06 | 13.30 | Cupin domain-containing protein |  |  |
| 2628045750 | 0.04 | 80.22 | 0.02 | 12.08 | acetyl-CoA C-acetyltransferase | K00626 | EC:2.3.1.9 |
| 2628046119 | 0.00 | 71.39 | 0.00 | 6.16 | pyruvate-ferredoxin/flavodoxin oxidoreductase | K03737 | EC:1.2.7.- |
| 2628044021 | 0.03 | 45.74 | 0.00 | 13.56 | basic amino acid/polyamine antiporter, APA family | K03294 |  |
| 2628044979 | 0.01 | 37.19 | 0.00 | 5.22 | glutamate dehydrogenase (NADP+) | K00262 | EC:1.4.1.4 |
| 2628045112 | 0.03 | 33.90 | 0.01 | 12.12 | glycolate oxidase | K00104 | EC:1.1.3.15 |
| 2628044483 | 0.01 | 21.72 | 0.00 | 4.44 | Acyl-CoA dehydrogenase |  |  |
| 2628044636 | 0.04 | 21.27 | 0.01 | 11.45 | Nucleotide-binding universal stress protein, UspA family |  |  |
| 2628044759 | 0.00 | 18.33 | 0.00 | 4.20 | Enoyl-CoA hydratase | K18474 | EC:5.3.3.14 |
| 2628046110 | 0.03 | 17.96 | 0.00 | 12.45 | Coiled stalk of trimeric autotransporter adhesin |  |  |
| 2628045528 | 0.01 | 17.81 | 0.00 | 4.15 | fructose-1,6-bisphosphatase II | K02446 | EC:3.1.3.11 |
| 2628045373 | 0.03 | 17.48 | 0.00 | 4.13 | branched-chain amino acid aminotransferase | K00826 | EC:2.6.1.42 |
| 2628044482 | 0.01 | 12.82 | 0.00 | 3.68 | electron transfer flavoprotein beta subunit | K03521 |  |
| 2628046231 | 0.01 | 12.37 | 0.00 | 3.63 | GMP synthase (glutamine-hydrolysing) | K01951 | EC:6.3.5.2 |
| 2628044763 | 0.03 | 12.28 | 0.00 | 3.62 | phosphoenolpyruvate synthase | K01007 | EC:2.7.9.2 |
| 2628044234 | 0.00 | 12.24 | 0.00 | 3.61 | Rubrerythrin |  |  |
| 2628045655 | 0.03 | 12.10 | 0.00 | 3.60 | electron transfer flavoprotein-quinone oxidoreductase | K00313 | EC:1.5.5.- |
| 2628044481 | 0.03 | 11.78 | 0.00 | 3.56 | electron transfer flavoprotein alpha subunit apoprotein | K03522 |  |
| 2628045111 | 0.03 | 11.64 | 0.00 | 3.54 | lactate permease, LutC, contrinas LUD domain | K03303 |  |
| 2628045659 | 0.01 | 10.15 | 0.00 | 3.34 | fused gene of L-lactate utilization protein LutB (4..461) and Fe-s oxidoredutase (477..713) | K18929 |  |
| 2628045311 | 0.03 | 9.12 | 0.00 | 3.19 | phosphotransferase system, enzyme I, PtsI | K08483 | EC:2.7.3.9 |
| 2628045641 | 0.00 | 8.39 | 0.00 | 3.07 | carbon starvation protein | K06200 |  |
| 2628044737 | 0.01 | 7.62 | 0.00 | 2.93 | Acyl-CoA dehydrogenase |  |  |
| 2628044263 | 0.03 | 7.38 | 0.00 | 2.88 | superoxide reductase | K05919 | EC:1.15.1.2 |
| 2628044438 | 0.03 | 7.33 | 0.00 | 2.87 | putative sigma-54 modulation protein | K05808 |  |
| 2628045615 | 0.03 | 7.14 | 0.00 | 2.84 | pyruvate-ferredoxin/flavodoxin oxidoreductase | K03737 | EC:1.2.7.- |
| 2628044628 | 0.01 | 6.60 | 0.00 | 2.72 | CO or xanthine dehydrogenase, Mo-binding subunit |  |  |
| 2628045573 | 0.03 | 6.42 | 0.00 | 2.68 | 4-hydroxy-3-methylbut-2-enyl diphosphate reductase | K03527 | EC:1.17.1.2 |
| 2628045955 | 0.03 | 6.30 | 0.00 | 2.66 | 4-alpha-glucanotransferase | K00705 | EC:2.4.1.25 |
| 2628045936 | 0.03 | 5.85 | 0.00 | 2.55 | electron transfer flavoprotein beta subunit | K03521 |  |
| 2628045654 | 0.03 | 5.34 | 0.00 | 2.42 | ferredoxin like protein | K03855 |  |
| 2628046036 | 0.01 | 5.33 | 0.00 | 2.41 | lysine-N-methylase | K18475 | EC:2.1.1.- |
| 2628043994 | 0.03 | 5.28 | 0.00 | 2.40 | DNA primase | K02316 | EC:2.7.7.- |
| 2628044680 | 0.03 | 5.21 | 0.00 | 2.38 | Uncharacterized membrane protein YdjX, TVP38/TMEM64 family, SNARE-associated domain |  |  |
| 2628044738 | 0.03 | 4.97 | 0.00 | 2.31 | NagD protein | K02566 |  |
| 2628045656 | 0.03 | 4.76 | 0.00 | 2.25 | demethylmenaquinone methyltransferase / 2-methoxy-6-polyprenyl-1,4-benzoquinol methylase | K03183 | EC:2.1.1.201 |
| 2628045673 | 0.03 | 4.68 | 0.00 | 2.23 | hydroxylamine reductase | K05601 | EC:1.7.99.1 |
| 2628045952 | 0.03 | 4.50 | 0.00 | 2.17 | starch phosphorylase | K00688 | EC:2.4.1.1 |
| 2628045995 | 0.03 | 4.47 | 0.00 | 2.16 | enoyl-CoA hydratase | K01715 | EC:4.2.1.17 |
| 2628045131 | 0.01 | 4.11 | 0.00 | 2.04 | exonuclease SbcC | K03546 |  |
| 2628044766 | 0.03 | 3.73 | 0.00 | 1.90 | Nicotinic acid mononucleotide adenylyltransferase |  |  |
| 2628045953 | 0.03 | 3.63 | 0.00 | 1.86 | 1,4-alpha-glucan branching enzyme | K00700 | EC:2.4.1.18 |
| 2628044729 | 0.03 | 3.30 | 0.00 | 1.72 | 3-oxoacyl-[acyl-carrier-protein] reductase | K00059 | EC:1.1.1.100 |
| 2628045649 | 0.03 | 3.18 | 0.00 | 1.67 | glycolate oxidase | K00104 | EC:1.1.3.15 |
| 2628044989 | 0.03 | 2.70 | 0.00 | 1.44 | elongation factor Tu | K02358 |  |
| 2628043992 | 0.03 | 2.65 | 0.00 | 1.41 | fructose-bisphosphate aldolase, class II | K01624 | EC:4.1.2.13 |
| 2628045274 | 0.03 | 2.22 | 0.00 | 1.15 | Arginine utilization protein RocB |  |  |
| 2628046311 | 0.03 | 2.21 | 0.00 | 1.14 | L-lactate dehydrogenase | K00016 | EC:1.1.1.27 |
| 2628045699 | 0.03 | 2.06 | 0.00 | 1.04 | selenium-dependent xanthine dehydrogenase |  |  |
| 2628046118 | 0.03 | 2.01 | 0.00 | 1.00 | DNA-binding transcriptional regulator, LysR family |  |  |
